# Derivatives of Amodiaquine as Potent Human Cholinesterases Inhibitors: Implication for Treatment of Alzheimer’s Disease

**DOI:** 10.3390/molecules29225357

**Published:** 2024-11-14

**Authors:** Ana Matošević, Dejan M. Opsenica, Marija Bartolić, Nikola Maraković, Andriana Stoilković, Katarina Komatović, Antonio Zandona, Suzana Žunec, Anita Bosak

**Affiliations:** 1 Institute for Medical Research and Occupational Health, Ksaverska Cesta 2, 10001 Zagreb, Croatia; amatosevic@imi.hr (A.M.); mbartolic@imi.hr (M.B.); nmarakovic@imi.hr (N.M.); azandona@imi.hr (A.Z.); suzana@imi.hr (S.Ž.); 2 Institute of Chemistry Technology and Metallurgy, University of Belgrade, Njegoševa 12, 11000 Beograd, Serbia; andriana.stoilkovic@ihtm.bg.ac.rs; 3 Centre of Excellence in Environmental Chemistry and Engineering, Njegoševa 12, 11000 Belgrade, Serbia; 4 Faculty of Chemistry, University of Belgrade, Studentski trg 12-16, 11158 Belgrade, Serbia; katarinabogojevic@chem.bg.ac.rs

**Keywords:** acetylcholinesterase, butyrylcholinesterase, chelation, cytotoxicity, 4-aminoquinoline derivatives

## Abstract

As some previously reported studies have proven that amodiaquine, in addition to its primary antimalarial activity, also has potential for new applications such as the inhibition of cholinesterases, in our study we focused on the evaluation of the influence of different substituents in the aminoquinoline part of the amodiaquine structure on the inhibition of human acetylcholinesterase and butyrylcholinesterase to investigate the possibility for their use as drugs for the treatment of AD. We synthesized a series of amodiaquine derivatives bearing H-, F-, CF_3_-, NO_2_-, CN-, CO_2_H- or CH_3_O- groups on the aminoquinoline ring, and determined that all of the tested derivatives were very potent inhibitors of both cholinesterases, with inhibition constants (*K*_i_) in the nM and low μM range and with prominent selectivity (up to 300 times) for the inhibition of acetylcholinesterase. All compounds displayed an ability to chelate biometal ions Fe^2+^, Zn^2+^ and Cu^2+^ and an antioxidant power comparable to that of standard antioxidants. Most of the compounds were estimated to be able to cross the blood–brain barrier by passive transport and were nontoxic toward cells that represent the models of individual organs. Considering all these beneficial features, our study has singled out compound **5**, the most potent AChE inhibitor with a CH_3_O- on C(7) position, followed by **6** and **14**, compounds without substituent or hydroxyl groups in the C(17) position, respectively, as the most promising compounds from the series which could be considered as potential multi-target drugs for the treatment of AD.

## 1. Introduction

Amodiaquine (AMQ) is a 4-aminoquinoline used as a therapy for malaria. Its use has been linked to significant side-effects, including agranulocytosis and hepatotoxicity [1,2,3], for which reason it is recommended to be used only as treatment and not as prophylaxis against malaria. However, its activity against some chloroquine-resistant strains of *Plasmodium falciparum* has led to a revival of its use as part of combination therapy with artesunate. AMQ was dropped from malaria control programs in the European Union and USA by the WHO in 1990 [4], but it remains in use in Africa and Oceania [5]. Following the modern concept of drug repurposing, with the aim of shortening the process of the development of new medication, various research groups have used AMQ or its derivatives to find new, more potent drugs for treatment of malaria or new potential application of AMQ derivatives such as the treatment of *Leishmania infantum* [6], breast cancer [7], invasive mycosis caused by resistant *Cryptococcosis strains* [8], thrombocytopenia syndrome caused by *Dabie bandavirus* [9] or severe viral infection caused by SARS-CoV-2 [10,11,12]. It has been known that AMQ exerts anti-inflammatory properties by inhibiting histamine N-methyltransferase (HNMT) [13]. Furthermore, AMQ is a highly selective orphan nuclear receptor Nurr1 agonist, and it was reported that in the presence of AMQ the deposition of amyloid-β plaques decreases and neuronal protection increases in a mice model of Alzheimer’s disease (AD) [14]. What is more, AMQ was reported to have beneficial effects on behavioral deficits in a 6-hydroxydopamine-induced (6-OHDA) Parkinson’s disease mice model, in which it upregulated tyrosine hydroxylase expression via phosphorylated p38 MAPK [15]. During a thorough quest for a new drug in the treatment of multiple sclerosis, using the new network-medicine-based algorithm for drug repurposing called SAveRUNNER, AMQ was found to be the most promising one [16].

Due to the fact that the 4-aminoquinoline system in combination with 4-*N*-aminophenol (Mannich phenol) reversibly inhibited electric eel acetylcholynesterase (*ee*AChE) and horse serum butyrylcholinesterase (*eq*BChE) [17], the structure of AMQ became of interest as a structural core for the development of drugs whose mode of action is the modulation of cholinesterases’ activities. In addition, AMQ was evaluated as an alternative to oximes for recovering AChE previously covalently inhibited by organophosphate compounds (OPCs) [18,19]. Although AMQ exhibits modest potency as an antidote for OPC poisoning, reported results have demonstrated AMQ’s capacity for interaction with amino acid residues of the AChE active site and, consequently, the potency of AMQ to act as a reversible inhibitor of AChE [20,21], a feature highly appreciated in the development of drugs for the symptomatic treatment of AD. AD is a complex neurological age-related disorder whose etiology is associated with clinical hallmarks such as a decline in neurotransmitter acetylcholine levels, amyloid-β (Aβ) peptide deposits, oxidative stress, dyshomeostasis of biometals and tau protein hyperphosphorylation and accumulation [22,23,24]. Although great efforts have been made over the past several years to develop drugs for AD treatment, they still cannot meet clinical needs. Today, AD treatment is mainly aimed at increasing the level of the neurotransmitter acetylcholine by inhibiting acetylcholinesterase (AChE) and butyrylcholinesterase (BChE), the enzymes responsible for acetylcholine’s hydrolysis. In this approach, AChE represents the main target due to the fact that the hydrolysis of acetylcholine is its physiological role, but with the progression of AD, the ratio of AChE and BChE levels change in favor of BChE, so the inhibition of BChE represents a promising strategy for the treatment of AD in advanced stages of the disease [22,25,26]. The drawback of this approach is that it is limited mostly to alleviating symptoms and improving the patient’s quality of life, but cannot stop the disease onset or progression. More recently, new drugs have been introduced to the market: monoclonal antibodies aiming to reduce the accumulation of amyloid β-plaques in the brain, thus allowing direct action on the very causes of the disease [27]. Recently, a highly promising idea has emerged to use compounds that can simultaneously act on at least two mechanisms involved in the development and/or progression of the disease. Recently, our group evaluated two series of 4-aminoquinoline derivatives as such multi-target compounds whose primary mode of action was the inhibition of human AChE and/or BChE with the ability to inhibit beta-secretase 1 (BACE1), chelate biometal ions and penetrate the blood–brain barrier (BBB) [28,29]. In line with that, AMQ derivatives with a modified quinoline ring and different 4-aminophenyl substituents exhibited additional activities important to AD’s etiology besides anti-cholinesterase activity, such as antioxidative activity and the ability to chelate Cu^2+^, Fe^2+^, Al^3+^ and Zn^2+^ ions, suggesting the potential use of AMQ-based molecules as multi-target-directed ligands (MTDLs) [19,20,30].

In this study, we synthesized amodiaquine derivatives bearing H-, F-, CF_3_-, NO_2_-, CN-, CO_2_H or CH_3_O- groups on the aminoquinoline ring of AMQ to determine their ability to moderate the activity of AChE and BChE, the main targets in the symptomatic treatment of AD. The obtained kinetic results were analyzed using molecular modelling. In addition, metal chelating properties and antioxidative potential were also determined to evaluate the possibility of those derivatives to act as multi-targeting agents for the treatment of AD. Drug-likeness of compounds was estimated by evaluation of their ability to cross the blood–brain barrier (BBB) by passive transport and their human intestinal absorption.

## 2. Results

### 2.1. Synthesis

**Figure 1:**
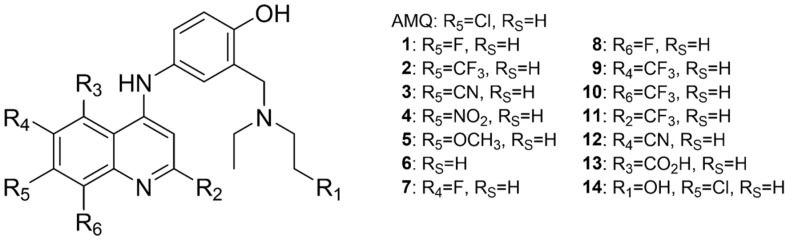
The general structure of amodiaquine derivatives. Numbers in brackets refer to the numeration of carbon atoms in the molecule.

In this study, AMQ and 14 derivatives of AMQ were synthesized (Figure 1), according to the previously described synthetic protocols [31,32]. Synthetized compounds (**1**–**13**) differ in the type and disposition of substituents on the quinoline ring (H-, Cl-, F-, CF_3_-, NO_2_-, CN- or CH_3_O- groups). A derivative with a hydroxyl group on C(17) was synthesized as well (**14**) (Figure 1).

**Scheme 1:**
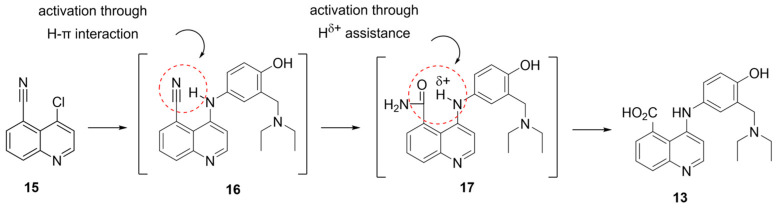
Hypothesized mechanism of the transformation of CN to CO_2_H in the quinoline system under mild conditions.

**Scheme 2:**
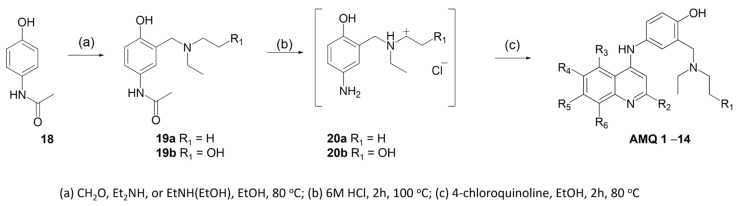
Synthesis of amodiaquine derivatives **1**–**14**.

During the synthesis of derivative from 4-chloroquinoline-5-carbonitrile (**15**, Scheme 1) and corresponding hydrochloric salt **20a** (Scheme 2), an unexpected product **13** (R_3_ = CO_2_H) was obtained. Analysis of IR and NMR spectra (SI document) of the obtained product revealed the presence of a CO_2_H group on the C(5) position instead of the expected CN. Such an outcome was not observed when 4-chloroquinoline-7-carbonitrile (R_5_ = CN,) or 4-chloroquinoline-6-carbonitrile (R_4_ = CN) were used; the 7-CN and 6-CN groups were preserved and the expected products (**3** and **12**) were obtained. We hypothesize that the transformation of the nitrile group occurred due to hydrolysis under acidic conditions, shortly after the introduction of Mannich aminophenol at position C(4). 5-CN underwent hydrolysis to 5-CO_2_H when an amino-aryl moiety was already present in the molecule and with the assistance of the neighboring amino group at position C(4) (Scheme 1). Hydrogen from the 4-amino group is close to the 5-CN substituent and more acidic than in the aliphatic amino group, which made it available to participate in additional activation of the nitrile group for hydrolysis through hydrogen–π interaction (the intermediate **17**, Scheme 1). Also, we assume that the intermediate amide group in **16** is further hydrolyzed through the similar neighboring amino-aryl group’s assistance to produce the final carboxyl group. A similar example of the transformation of CN- to CH_3_O- was found in the synthesis of 2-aminonicotinic acid derivative, which started from corresponding 2-chloronicotinonitrile by the action of aqueous ammonia in the autoclave at 170–175 °C [33]. To the best of our knowledge, this is the first time that this type of transformation was observed in the quinoline system under mild conditions. However, additional confirmation of our hypothesis is needed.

The structures of the obtained compounds were verified via IR and NMR spectral analysis, and their purity was determined by HPLC analysis (spectral analyses and purity are given in the Supplementary Information). The HPLC purity of all tested derivatives was >95%, verified by two different HPLC conditions. 

### 2.2. Inhibition of AChE and BChE

All compounds were tested for their ability to inhibit the activity of AChE and BChE, and for all of them, a reversible mode of inhibition was determined with inhibition potency expressed with the dissociation constants of the enzyme–ligand complex (*K*_i_) (Table 1). The *K*_i_ constants determined for AChE were in the range 0.025–7.6 µM (Table 1). Concerning the substituents on the C(7) position on the quinoline ring, it seems that the determining factor for inhibition potency towards AChE are the electronegativity of the substituents and their size. Namely, the replacement of a chlorine atom with stronger electron-withdrawing substituents like CF_3_-, CN- or NO_2_- only slightly diminished the inhibition potency compared to AMQ. On the other hand, the replacement of chlorine with smaller atoms like fluorine or hydrogen, as in compounds **1** and **6**, respectively, decreased the inhibition potency by about four times compared to AMQ.

**Figure 2:**
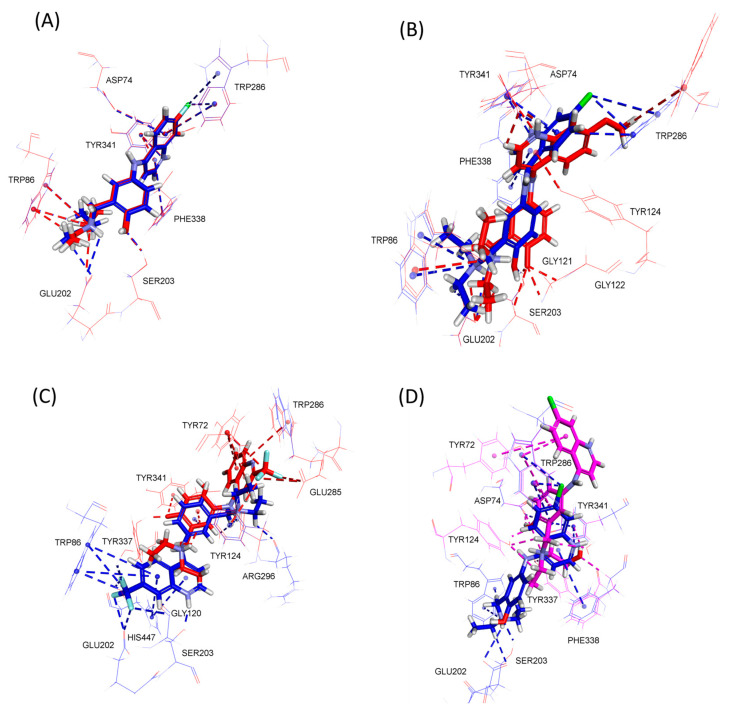
Superposition of modelled complexes of AChE and AMQ (carbons and interactions in blue) and compound **1** (carbons and interactions in red) (**A**), AMQ (carbons and interactions in blue) and compound **5** (carbons and interactions in red) (**B**), compound **2** (carbons and interactions in blue) and compound **11** (carbons and interactions in red) (**C**), and AMQ (carbons and interactions in blue) and compound **14** (carbons and interactions in magenta) (**D**).

Superposition of model complexes between AChE and AMQ and AChE and **1** (Figure 2A), the derivative with fluorine on the C(7) position, obtained by molecular docking studies, reveals almost identical binding geometry between two compounds. However, the decrease in inhibition potency of derivative **1** can be attributed to the stronger interaction of AMQ with Trp286, a distinct amino acid from the peripheral anionic site of AChE which plays a key role in the initial step of substrate trafficking inside the AChE active site gorge through hydrophobic Cl–π interaction via its chlorine substituent. The most potent inhibitor of AChE is compound **5** with an electron-donating methoxy group on C(7), which demonstrated a two times higher inhibition potency compared to AMQ. In elucidating the underlying ligand–receptor interaction pattern that dictates the degree of inhibition potency of compounds of interest, one should keep in mind that it is important to look beyond the number and type of non-bonding interactions. Such is the case of compound **5**; superposition of model complexes between AChE and AMQ and AChE and **5** (Figure 2B) predicts not only a higher number of non-bonding interactions of a strong type, i.e., attractive charge interactions (two vs. one) and conventional hydrogen bonds (three vs. one), in favor of compound **5**, but also points to unusual conformation of distinct peripheral anionic site residue Trp286. In this unique conformation, the indole ring of Trp286 “flips” out of the active site gorge core and becomes solvent-accessible. This distinct conformation of Trp286 was first reported by Bourne et al. in the case of the complex between high-affinity regioisomer of tacrine–phenanthridinium cycloaddition product and AChE and was recognized as a feature of a complex with high-potency inhibitors [35].

Additionally, it seems that the presence of a strong electron-withdrawing group close to the nitrogen atom in the pyridine moiety (positions C(2) and C(8)) lowers the capacity for AChE inhibition due to their high electronic influence, demonstrated by a 100 times lower inhibition potency of compound **11** with CF_3_ substituent on C(2) position compared to **2**. The results of the molecular docking studies, visualized with the superposition of model complexes between AChE and **2** and AChE and **11** (Figure 2C), revealed that the CF_3_ position governs different orientation of compounds **2** and **11**. Indeed, with CF_3_ substituent on the C(7) position, compound **2** is oriented with the quinoline part of the molecule in the bottom of the active site gorge, directing the CF_3_ substituent into the region outlined by the choline binding site and oxyanion hole. On the other hand, compound **11** with a CF_3_ substituent on the C(2) position is predicted to bind with the quinoline part of the molecule oriented towards the entrance of the active site gorge, lacking any interaction with residues from the catalytic triad. Overall, compound **2** is predicted to form half a dozen more interactions with residues outlining the active site gorge than compound **11**. Introduction of the hydroxyl group into the side chain of AMQ in **14** ends up with an about two times lower inhibition potency compared to AMQ. A plausible explanation for the decrease in the inhibition potency of **14** may lie in the more restricted access to the bottom of the active site gorge because of the additional hydroxyl group. Namely, the elongation of the conformationally loose diethylamino group with the OH- group with a high hydrogen bonding potential diminishes the rate of passage of compound **14** to the bottom of the gorge by its engagement in non-bonding interactions with residues outlining the active site gorge via its hydroxyl group, also directing its second hydroxyl group to interact via hydrogen bonding with the hydroxyl group of Tyr124, one of the two amino acids constituting active site’s bottleneck. One such possible transit structure between AChE and **14** is shown in Figure 2D; the superposition of model complexes between AChE and AMQ and AChE and **14** reveals differences in the extent of accommodation of the compounds inside the active site gorge: while AMQ positions itself towards the bottom of the active site gorge, the less potent **14** is locked into the upper part of the active site.

The value of the *K*_i_ constant for AMQ is about 15 times lower compared to the IC_50_ reported in Bienerisch et al., which could be ascribed to the fact that the reported IC_50_ was determined at only one concentration of acetylthiocholine, used as a substrate (ATCh; 0.71 mM), which was higher than the concentration range used in our experiments [19]. If compared to tacrine, an anti-AD drug with a similar structure discontinued from therapeutic use in 2013 [35,36,37], AMQ and **7** are equally potent inhibitors of AChE, while **5** is even two times more potent. Four more compounds (**2**, **3**, **4** and **9**) are about two times less potent than tacrine. All of those six compounds could be considered as potential anti-AD drugs considering their ability to inhibit the action of AChE.

**Figure 3:**
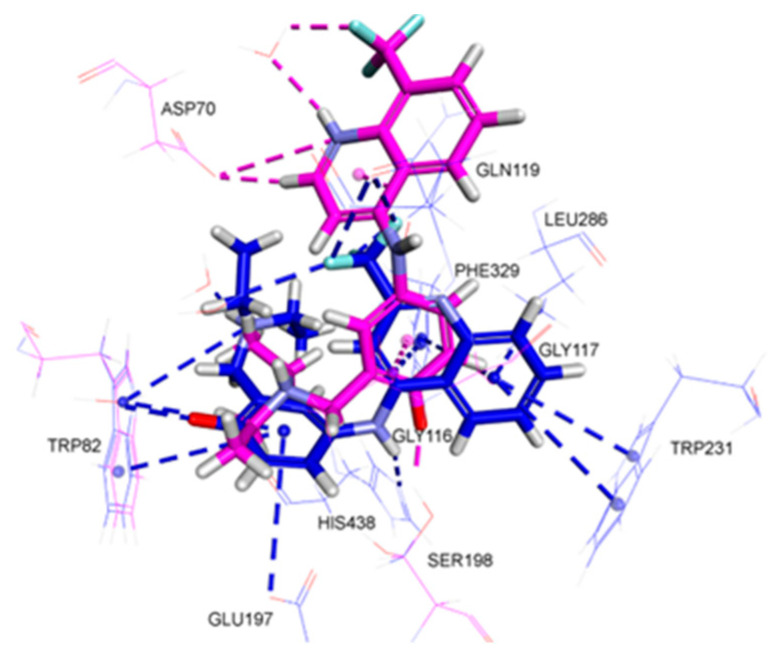
Superposition of modelled complexes of BChE and **10** (carbons and interactions in magenta) and compound **11** (carbos and interactions in blue).

Same as with AChE, all tested compounds reversibly inhibited BChE activity, but with generally lower inhibition potency and corresponding *K*_i_ constants, ranging from 1.7 to 21 µM. The most potent BChE inhibitor is compound **11** with a CF_3_- group on the C(2) position on the quinoline ring. The presence of the CF_3_- group in **10** and **11** at positions C(8) and C(2), respectively, has quite a different effect on BChE compared to that of AChE; while it significantly attenuates the inhibition of AChE in both positions, **11** is the most active against BChE, while compound **10**, with its CF_3_- group on C(8) with *K*_i_ = 20 µM, is one of the least active within the series. While the introduction of the CH_3_O- group as a strong electron-donating group increased inhibition of AChE compared with AMQ, in the case of BChE, its influence is negligible. Next to **10**, the least active derivatives were **12** and **3** with CN-groups at the C(6) or C(7) position, respectively, with a *K*_i_ around 20 µM. Our kinetic results are in agreement with molecular docking studies. The superposition of model complexes between BChE and **11** and **10** (Figure 3) suggests that the origin of the said difference in inhibition potency arises from different orientations between the two compounds. Compound **11** occupies the bottom of the active site gorge, oriented perpendicularly to the axis connecting the gorge entrance and its bottom and slightly bent so that hydroxyphenylamino part of the molecule engages in T-shaped aromatic π–π interactions with Trp82, and the hydroxyphenylamino part of the molecule establishes the same type of interactions with Trp231 on the opposite side of the active site gorge—an orientation which directs both the diethylamino group and the CF_3_- group to the center of the active site. On the contrary, compound **10** binds in an elongated conformation along said axis with the quinoline part located at the gorge entrance and the hydroxyphenylamino part directed towards the bottom of the active site. However, it seems the CF_3_- group on the C(8) position of compound **10** would make it impossible, on account of its steric hindrance, to adopt the same conformation as compound **11**. As a consequence, the number of non-bonding interactions established with neighboring residues and water molecules is reduced from 17 (**11**) to 7 (**10**).

As for BChE, the *K*_i_ constant for AMQ is about 9 times lower compared to the IC_50_ reported in Bienerisch et al., who reported an IC_50_ at 1.58 mM ATCh, which was three times higher than the highest concentration used in our experiments [19]. Compared to tacrine, the tested compounds were at least 300 times less potent inhibitors of BChE [35].

Generally, all compounds showed higher affinity for AChE compared to BChE with the selectivity index in the range of 11–304. The most selective was compound **5**, which could be ascribed mostly to its very high-potency inhibition of AChE. Six more compounds were more than 100 times more selective: AMQ, **3**, **4, 7**, **9** and **12**. The only exception is derivative **11**, the most potent BChE inhibitor with a CF_3_- group on C(2) of the quinoline ring, which was about five times more selective to BChE.

### 2.3. In Vitro Antioxidative Potential

**Figure 4:**
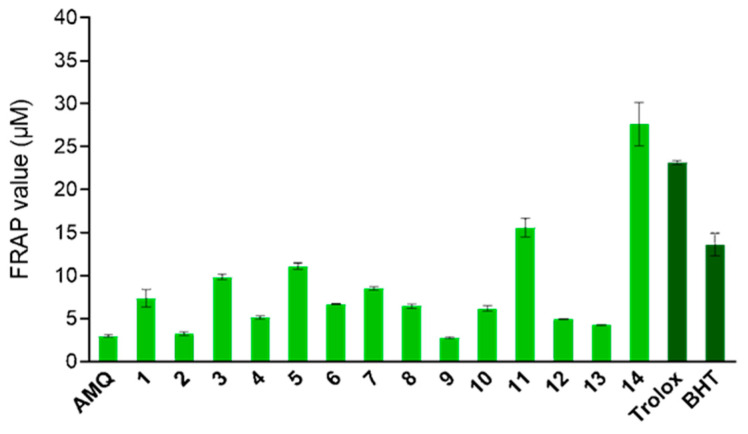
Determined FRAP values. Columns represent the FRAP values for a 10 μM concentration of each amodiaquine derivative.

Oxidative stress is recognized as one of the contributors to the development and progression of various neurodegenerative disorders, particularly AD. Therefore, in this study we used the ferric reducing antioxidant power (FRAP) assay to measure the antioxidant capacity of amodiaquine derivatives and their ability to reduce an oxidant in the form of a ferric salt [38]. The measurements were conducted using a concentration of 10 µM for the tested compounds. This concentration was selected based on the *K*_i_ values for these compounds with respect to AChE and BChE. The FRAP values determined for amodiaquine derivatives and standard antioxidants Trolox and BHT are presented in Figure 4. Overall, all the tested compounds exhibited reducing power at a concentration of 10 μM. Notably, the compound **14** stood out, demonstrating even greater potency than both Trolox and BHT. Additionally, the FRAP values determined for five other compounds (**1**, **3**, **5**, **7** and **11**) were not more than 50% lower than those of either Trolox or BHT. The observed good ferric reducing power of the tested derivatives is likely attributed to the presence of the *p*-aminophenol moiety in their structures, which readily donates electrons [39,40]. Additionally, amodiaquine is known to be susceptible to oxidation, with its redox chemistry characterized by an oxidation reaction that involves the transfer of two electrons [41]. Our results (Figure 4) corroborate previous reports of very good antioxidant activity of tacrine–melatonin, tacrine–8-hydroxyquinoline, and tacrine–caffeic acid hybrids [42,43,44]. Based on their FRAP values, the tested amodiaquine derivatives can be considered as highly promising anti-AD drugs in terms of multitarget drugs targeting cholinesterase as their main activity and attenuating adverse effects of oxidative stress associated with AD as an additional beneficial activity.

### 2.4. Metal Chelation Study

**Figure 5:**
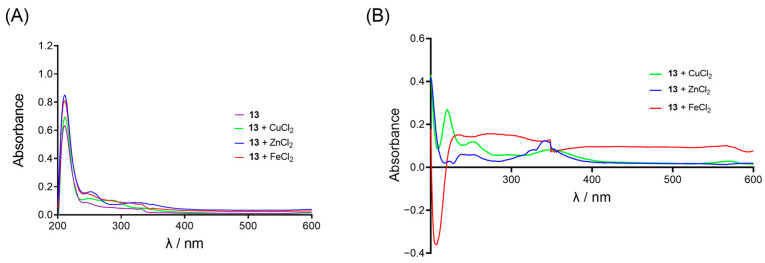
(**A**) The UV-VIS spectra of compound **13** (purple) and after mixing with Cu^2+^ (green), Zn^2+^ (blue) and Fe^2+^ (red). (**B**) The differential spectra of compound **13**–metal complex for Cu^2+^ (green), Zn^2+^ (blue) and Fe^2+^ complexes (red).

The ability of compounds to chelate biometal ions Fe^2+^, Zn^2+^ and Cu^2+^ was tested to evaluate their potential to be used in the treatment of AD as metal chelators with the aim of reducing reactive oxygens species (ROS) production and formation of toxic metal–Aβ plaques [45,46]. The absorbance spectra of compound and biometal mixtures were recorded at 1, 60 and 120 min after mixing. For all of the biometal–compound mixtures, changes in absorbance intensity, as the evidence of the formation of a metal–compound complex (Figure 5), were observed when compared to the spectra of compounds and metals alone [47]. Since after 60 min of incubation, the spectra of the compound–metal mixtures did not change with time, spectra recorded after 60 min were chosen for differential spectra analysis. Differential spectra confirmed that all tested compounds have the ability to chelate all tested metals (Figures S9–S11), indicating their possible use as modulators of biometal homeostasis. Of particular interest is the ability to chelate Fe^2+^ due to its involvement in the mechanism of regulated cell death associated with an increase in oxidative stress generated by free radicals formed via the Fenton reaction, ferroptosis [48,49].

### 2.5. Cytotoxicity

The cytotoxicity of compounds was evaluated on human cell lines HepG2, HEK293 and SH-SY5Y over a 24 h exposure period. The concentration range (0.001–1000 µM) was selected based on the values of *K*_i_ constants determined for AChE and BChE, and to allow calculation of the IC_50_ values (Table 2). According to their IC_50_ values, the most toxic compounds were **11** on HEK293 and **4** on HepG2 and SH-SY5Y. Compounds **2**, **9** and **14** were not toxic to any tested cell type. In general, almost all compounds were non-toxic in concentrations 100 times higher than values of *K*_i_ determined for AChE. The exceptions were compounds **10**, **11** and **13**. Moreover, compounds **3**, **6**, **7**, **9**, **13** and **14** have IC_50_ values ≥ 100 µM, a value that is 10 times higher than the concentration of compounds used for the FRAP assay.

### 2.6. In Silico Prediction of Blood–Brain Barrier (BBB) Penetration

**Figure 6:**
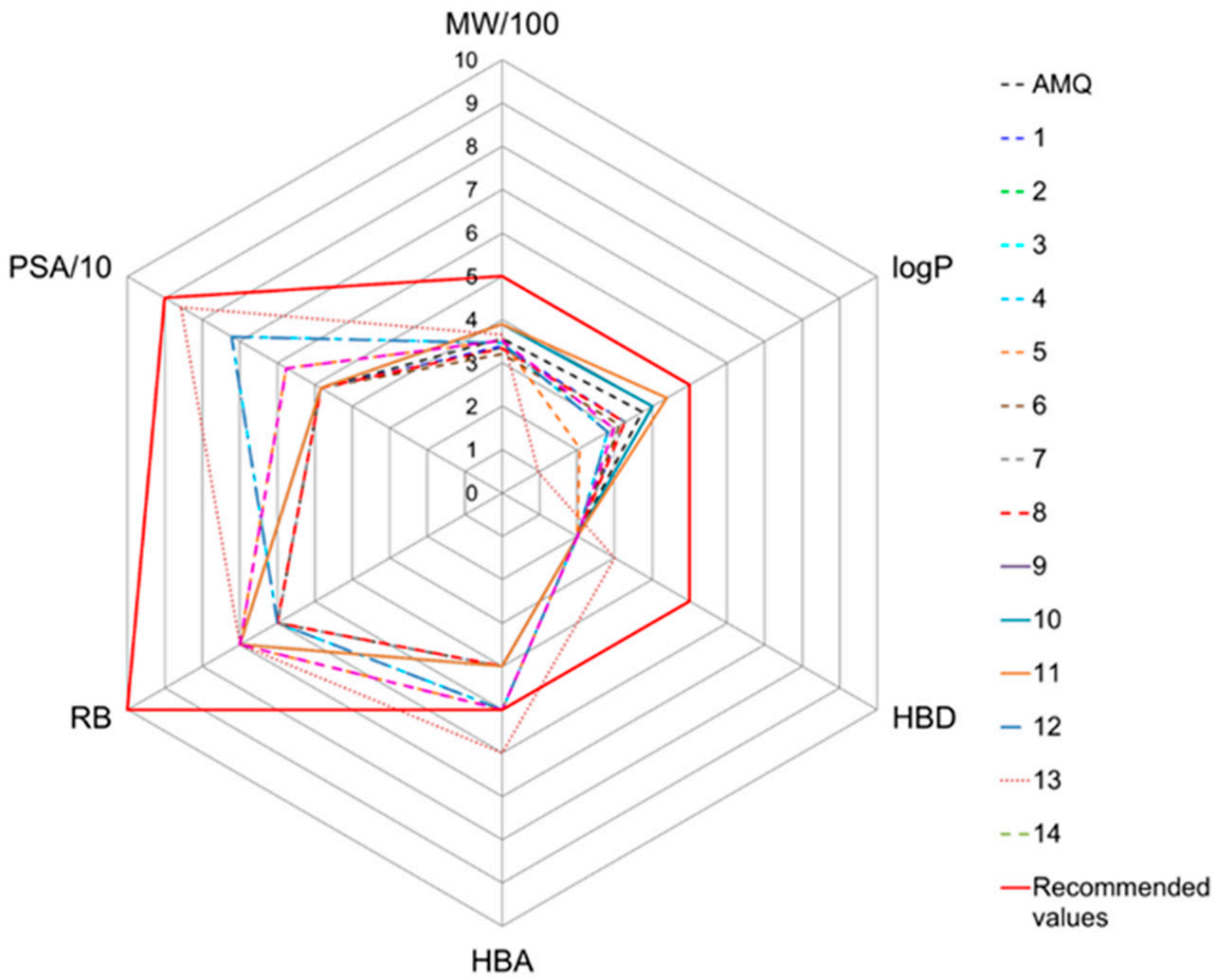
Radar plot of physicochemical properties of amodiaquine derivatives. M_w_ stands for molecular weight, logP for hydrophobicity, HBD for number of hydrogen bond donors, HBA for hydrogen bond acceptors, RB for number of rotatable bonds and PSA for polar surface area.

The ability of compounds to cross the BBB was estimated by comparing the calculated values of molecular weight (Mw); hydrophobicity (logP); the number of hydrogen bond donors (HBDs), hydrogen bond acceptors (HBAs) and rotatable bonds (RBs); and the polar surface area (PSA), physicochemical properties important for passive transport of compounds to the central nervous system (CNS), to the recommended values based on the physicochemical properties of known drugs active in the CNS [50,51,52]. Generally, all CNS-active drugs have a Mw < 500 g moL^−1^, logP < 5, less than five HBDs and HBAs, less than ten RBs and a PSA < 90 Å^2^. According to the calculated values of the physicochemical properties (Figure 6), all of the tested compounds should have the ability to penetrate the BBB after oral administration. The only exception was compound **13**, which had a higher number of HBAs than recommended and therefore should have a moderate possibility for passing the BBB. The values of the tested physicochemical properties can be found in Table S1.

### 2.7. Human Intestinal Absorption

The percentage of amodiaquine derivatives that would be absorbed through the human intestine was estimated in silico using the Deep-PK prediction model. According to the model, with the absorption percentage in the range between 92.8 and 99.7 (Table S2), all of the compounds would likely be very well absorbed in the human intestines.

## 3. Discussion

Alzheimer’s disease is a progressive neurodegenerative disease. As an age-related disease, its prevalence is increasing, and currently available therapies still cannot meet the clinical needs. In this situation, the possibility of decreasing the developmental cost and time to reach the market of new treatments requires the use of the drug repurposing concept over standard drug discovery [53]. As some previously reported studies proved that AMQ, in addition to its primary action as an antimalarial drug, also has potential for new applications such as the inhibition of cholinesterases, in this study we focused on the exploration of AMQ as a structural scaffold for the development of AD drugs [21,30].

As the primary goal of this study was to explore the influence of different substituents in the aminoquinoline part of AMQ molecule on the inhibition of human AChE and BChE to evaluate the possibility of their use as drugs for symptomatic treatment of different stages of AD, we synthesized fourteen amodiaquine derivatives bearing H-, F-, CF_3_-, NO_2_-, CN-, CO_2_H- or CH_3_O- groups on the aminoquinoline ring. All of the tested derivatives were very potent inhibitors of both cholinesterses, with inhibition constants (*K*_i_) in the nM and low μM range with prominent selectivity (up to 300 times) for the inhibition of AChE. This inhibition selectivity pattern suggests that these compounds could be effective across various stages of the disease. Furthermore, according to our kinetic results and confirmed by docking, most of the compounds are dual-site-binding inhibitors of AChE, indicating those compounds as class of potential new anti-Alzheimer agents able to inhibit the formation of toxic AChE–amyloid complexes and consequently positively modify the course of AD. The structure–activity analysis revealed that AChE prefers compounds with larger electron-donating groups, since the most potent AChE inhibitor is compound **5** with a CH_3_O- group on the quinoline ring. Among halogens, AChE has higher affinity for compounds with atoms larger than fluorine, where interactions with amino acids from the AChE peripheral anionic site, preferably with Trp286, are important. The addition of a bulky CF_3_- substituent in the positions close to the nitrogen atom in the pyridine moiety (i.e., positions C(2) and C(8)) lowers the capacity for AChE inhibition due to their high electronic influence. BChE, on the other hand, prefers binding to bulkier molecules, regardless of the nature of the substituents on the quinoline ring, as the most potent BChE inhibitor was compound **11** with a CF_3_- group on the C(2) position on the quinoline ring, while the least active derivatives were **12** and **3** with CN- groups at the C(6) or C(7) position.

As the multifaced nature of the AD calls for the development of multitarget-directed ligands able to simultaneously act on at least two mechanisms involved in onset and progression of the disease, compounds were also tested as chelators of the biometal ions Fe^2+^, Zn^2+^ and Cu^2+^, and their antioxidant capacity was measured. The results demonstrated that all tested compounds were capable of chelating the biometals and exhibited antioxidant power comparable to standard antioxidants. Additionally, most of the compounds were predicted to cross the BBB through passive transport, a necessary prerequisite for activity within the central nervous system. Moreover, all compounds were nontoxic toward cells that represent models of main target organs (neural cells) and organs responsible for their metabolism and/or excretion (liver and kidney) in concentrations at which they exert their biological activity.

**Figure 7:**
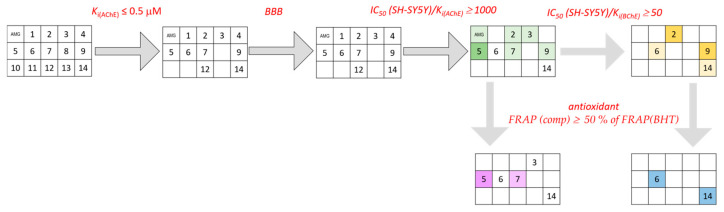
Schematic presentation of comprehensive analysis of the results.

Considering all these beneficial features, our study has singled out several compounds with potential to be used in the treatment of AD, targeting different targets. The selection of compounds was based on a priority list of activities which we consider important regarding their potential targets. As a first and most important cut off, we chose *K*_i(AChE)_ ≤ 0.5 µM, which is a concentration about 12 times higher compared to that of tacrine and 20 times than that of donepezil, the most used drugs for the symptomatic treatment of AD [54]. As the second criterion, we took their ability to cross the BBB, which is a prerequisite for compounds to be used in CNS. As the third cut off, the toxicity of compounds towards neuronal cells was set 1000 times lower than their *K*_i(AChE)_ (IC_50_ (SH-SY5Y)/*K*_i(AChE)_ ≥ 1000) regarding the role of AChE in neurotransmission of the neurotransmitter acetylcholine in the brain. As a result of selection according to these three requirements, six compounds (Figure 7, green) stood out as promising candidates for the symptomatic treatment of AD, where compound **5** is the most promising one. Furthermore, as inhibition potency towards BChE is also a desirable property, the compounds with a ratio of toxicity towards neuronal cells and inhibition potency towards BChE higher than 50 (IC_50_ (SH-SY5Y)/*K*_i(BChE)_ ≥ 50) were distinguished (compounds **2**, **6**, **9** and **14)**, among which compounds **2** and **9** were those with higher BChE selectivities. In addition to their anticholinesterase activity, the majority of the compounds also possess the ability to be used as disease modifiers affecting mechanisms involved in the onset of the disease. In line with that, we looked for the compounds which, in addition to the inhibition of AChE and/or BChE, also have FRAP values higher than 50% of the FRAP value for BHT. According to that selection criterion, three compounds were pointed out as potential multitarget compounds, **5**, **6** and **14**, of which compounds **6** and **14** possess all desired activities.

## 4. Materials and Methods

### 4.1. Synthesis and Isolation of Compounds

All of the chemicals and reagents for the synthesis of amodiaquine derivatives were purchased from commercial sources and were used without additional purification. Quinolines 4,7-dichloroquinoline, 4-chloro-2-(trifluoromethyl)quinoline, 4-chloro-6-(trifluoromethyl)quinoline, 4-chloro-8-(trifluoromethyl)quinoline, 4-chloro-8-fluoroquinoline, 4-chloro-7-metoxyquinoline and 4-chloroquinoline were purchased from a commercial source (Aldrich, St. Louis, MO, USA), while the other 4-chloroquinolines were prepared according to Schemes S1 and S2 (see Supplementary Information for details). All solvents used for synthesis and purification were prepared according to procedures described in the literature [55]. The solvents used for HPLC analysis were all HPLC-grade and of high purity and were used without additional purification. The purity of the tested compounds was determined by HPLC analysis, and all compounds had >95% purity. Other experimental details of synthetic and isolation procedures, spectral data and HPLC chromatograms are given in the Supplementary Information.

All amodiaquine derivatives, including AMQ itself, were synthesized following the procedures described in the literature (Scheme 2) [31,32]. Briefly, starting with Mannich reaction of *N*-(4-hydroxyphenyl)acetamide (18) and secondary amines diethylamine or 2-(ethylamino)ethan-1-ol in ethanol at 80 °C, the key intermediates **19a** and **19b** were obtained (Scheme 2). Deacetylation of the aniline amino group with 6 M HCl at 100 °C provided corresponding hydrochloric salts (20a or 20b), which were used in the next step without additional purification. By the addition of 4-chloroquinolines bearing electron-withdrawing groups F-, CF_3_-, NO_2_- or CN- at different positions on the quinoline core or electron donor CH_3_O- group at the C(7) position, compounds 1–14 were synthesized. Characterization of derivatives, their 1H and 13C NMR spectra and their HPLC analyses are presented in the Supplementary Information.

### 4.2. Inhibition Study

Acetylthiocholine (ATCh) and 5,5’-dithiobis(2-nitrobenzoic acid) (DTNB) were purchased from Sigma Chemical Co., St. Louis, MO, USA. ATCh was dissolved in water and DTNB in 0.1 M sodium phosphate buffer (pH 7.4). Stock solutions of amodiaquine derivatives were dissolved in DMSO, and all further dilutions were made in water. Purified human BChE and recombinant human AChE were kindly provided by Dr Florian Nachon (Département de Toxicologie, Armed Forces Biomedical Research Institute, Brétigny-sur-Orge, France). The enzymes were diluted in a phosphate sodium buffer 0.1 M (pH 7.4) containing 0.01% BSA.

Cholinesterase activity measurements, evaluation of *K*_i_ (dissociation constants of enzyme−inhibitor complex) and *K*_s_ constants (dissociation constants of enzyme−substrate complex) were performed following procedures described previously [56,57]. Briefly, enzyme activity was measured spectrophotometrically using a slightly modified Ellman method [56]. Activities of AChE and BChE were measured at different ATCh concentrations (final concentrations 0.050−0.50 mM) in the absence and presence of an amodiaquine derivative (final concentrations 0.020−50 µM, depending on the compound) selected to inhibit the enzymes by 20−80%. At least three concentrations of inhibitors for each substrate concentration were used in at least three experiments.

The apparent inhibition constant (*K*_i,app_) was calculated using the Hunter–Downs equation and linear regression analysis [57]:

*K*_i,app_ = *v*_i_·*i*/(*v*_0_ − *v*_i_) = *K*_i_ + *K*_i_/*K*_S_ · *s*, (1)

where *s* stands for final substrate concentration, *v*_0_ for the activity of enzyme measured in the absence and *v*_i_ in the presence of a given amodiaquine concentration (*i*). The y-intercept determines the enzyme–inhibitor dissociation constants (*K*_i_), while the x-intercept determines the enzyme–substrate dissociation constant, *K*_S_ [57].

### 4.3. Molecular Docking

All details of the docking procedure were described previously [28] using crystal structures of free AChE (PDB ID: 4EY4) and BChE (PDB ID: 1P0I) [58,59]. Briefly, the flexible docking protocol was used with specified residues selected as flexible, as described earlier [34,58,59]. Before starting the molecular docking protocol, ligands were created, minimized and prepared with regard to possible different protonation states, isomers and tautomers at pH 7.4 utilizing corresponding protocols implemented in Biovia Discovery Studio Client v21. (Dassault Systèmes, Vélizy-Villacoublay, France). The representative pose of each of the docked ligands was chosen based on the highest consensus score across a range of values predicted by the scoring functions estimating binding affinities, as implemented in the Biovia Discovery Studio Client v21. Score Ligand Poses protocol.

### 4.4. Antioxidant Activity

The in vitro antioxidant activity of tested compounds was evaluated using the FRAP assay following a procedure described earlier [38]. The reducing capacity of amodiaquine derivatives was determined for 10 µM compound concentrations selected according to the inhibition constants (*K*_i_) determined for AChE and BChE. Trolox and butylated hydroxytoluene (BHT) were used as standard antioxidants. The absorbance was recorded at 593 nm using a microplate reader (SpectraMax iD3, Molecular Devices, San Jose, CA, USA) against a blank. All measurements were taken in three independent triplicates in at least three independent experiments. FRAP values, denominating the reduction of ferric-tripyridyltriazine (Fe^3+^-TPTZ) to ferrous tripyridyltriazine (Fe^2+^-TPTZ) by the tested compounds, were calculated on the basis of a standard curve obtained using Fe_2_SO_4_x7H_2_O.

### 4.5. Metal Chelation

The capability of compounds to chelate biometals Fe^2+^, Cu^2+^ and Zn^2+^ was tested using metal salts ZnCl_2_, CuCl_2_x2H_2_O and FeCl_2_x4H_2_O. All compounds were dissolved in DMSO as 100 mM, and then diluted in methanol to a 5 µM concentration. All metal salts were dissolved in methanol. The absorption spectra of a fixed number of compounds (5 µM), metal salt (10 µM) and of the mixture of compounds (5 µM) and metal salt (10 µM) were recorded (200 to 600 nm at three time points (1, 60 and 120 min)). The baseline (methanol) was recorded and subtracted from every spectrum. Changes in the spectra of compounds, compared to that of the compound–metal mixture, indicated the formation of the compound–biometal complex, which was additionally confirmed by differential UV/VIS spectra obtained by the numerical subtraction of the spectra of the metal and the compound from the spectra of the compound–biometal mixture [60]. All spectra recordings were performed in a 1 cm quartz cuvette (final volume 1 mL) at 25 °C using a UV-Vis spectrophotometer (Cary 300 spectrophotometer Varian, Inc., Belrose, Australia). Differential spectra and all presentations of the spectra were conducted in GraphPad Prism, GraphPad Software v9.4.1.

### 4.6. Cytotoxicity Assay

The human Caucasian hepatocyte carcinoma HepG2 (ECACC 85011430), human embryo kidney HEK293 (ECACC 85120602) and human neuroblastoma SH-SY5Y (ECACC 94030304) cell lines were obtained from the certified cell-bank European Collection of Authenticated Cell Cultures (ECACC) through Sigma-Aldrich (Steinheim, Germany). HepG2 and HEK293 cells were grown in EMEM and SH-SY5Y in DMEM-F12, as described previously [61], and cell cultivation and maintenance were conducted according to standard protocol [62]. All media and supplements were purchased from Sigma-Aldrich, Steinheim, Germany.

The cytotoxic profiles of tested compounds were determined by measuring the succinate dehydrogenase mitochondrial activity of exposed cells. The commercially available MTS detection reagent assay (CellTiter 96^®^ AQueous One Solution Cell Proliferation Assay, Promega, Madison, WI, USA) was used. The procedure followed a previously described protocol [63]. Cells were exposed to compounds in a concentration range of 0.01–1000 µM for 24 h. DMSO (20%) was used as positive control to validate the method. After desired time of incubation at 37 °C in a 5% CO_2_ atmosphere, cells were washed with PBS buffer and 100 µL of EMEM or DMEM medium and 20 µL of MTS were added in each well, incubated up to 1 h after which the absorbance was read at 492 nm on an Infinite M200PRO plate reader (Tecan Austria GmbH, Salzburg, Austria). Data are presented as percentages of the inhibited cells to control untreated cells, i.e., the percentage of cytotoxicity.

### 4.7. Prediction of Ability to Pass the Blood–Brain Barrier (BBB)

The capability of compounds to cross the BBB was estimated by determining their physicochemical properties important for passive transport: lipophilicity (logP), molecular weight (MW), polar surface area (PSA), numbers of H-bond donors (HBDs) and H-bond acceptors (HBAs) and the number of rotatable bonds (RBs) using the Chemicalize protocol [64,65,66] and comparing them with the recommended values obtained for known central nervous system active drugs [49,50,51].

### 4.8. In Silico Evaluation of Human Intestine Absorption

The prediction of human intestinal absorption of the compounds was estimated with the Deep-PK online platform [67], which discriminates between intestinally well absorbed and poorly absorbed molecules. This prediction is based on two parameters: the lipophilicity of chemicals, evaluated from the partition coefficient (LogP) value calculated according to the Wildman–Crippen method (WLogP), and their polarity, determined by a calculated topological polar surface area (tPSA) value [67].

## Figures and Tables

**Table 1:** Inhibition of human AChE and BChE by amodiaquine derivatives. All measurements were performed in duplicates, and dissociation constants of enzyme–inhibitor complexes with standard error values (*K*_i_ ± SE) were determined from at least three experiments.

Compound	Structure	*K*_i_/µM		SI
		AChE	BChE	
AMQ	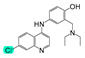	0.046 ± 0.001	9.1 ± 0.7	200
**1**	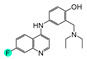	0.17 ± 0.01	13 ± 1	77
**2**	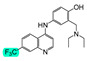	0.075± 0.003	7.3 ± 0.8	97
**3**	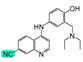	0.078 ± 0.004	19 ± 1	240
**4**	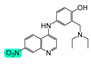	0.084 ± 0.003	14 ± 1	170
**5**	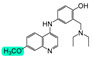	0.025 ± 0.001	7.6 ± 0.4	300
**6**	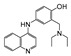	0.23 ± 0.01	5.9 ± 0.8	26
**7**	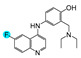	0.051 ± 0.002	11 ± 1	220
**8**	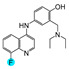	0.51 ± 0.03	5.4 ± 0.5	11
**9**	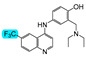	0.093 ± 0.003	14 ± 1	150
**10**	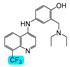	1.2 ± 0.1	20 ±1	17
**11**	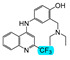	7.6 ± 0.3	1.7 ± 0.1	0.22
**12**	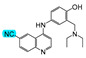	0.19 ± 0.01	21 ± 1	111
**13**	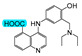	1.3 ± 0.1	16 ± 1	12
**14**	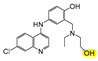	0.11 ± 0.00	6.8 ± 0.3	62
Tacrine [34]		0.040 ± 0.006	0.0063 ± 0.0001	0.16

SI = *K*_i(BChE)_/*K*_i(AChE)._

**Table 2:** The cytotoxicity of tested compounds evaluated by MTS assay after 24 h cell exposure expressed as IC_50_ values ± SE (µM).

Compound	HEK293	HepG2	SH-SY5Y
AMQ	≥1 mM	≥1 mM	134 ± 36
**1**	90 ± 22	219 ± 28	90 ± 10
**2**	≥1 mM	≥1 mM	≥1 mM
**3**	103 ± 3	471 ± 19	98 ± 3
**4**	33 ± 3	39 ± 6	26 ± 4
**5**	37 ± 11	100 ± 10	36 ± 5
**6**	350 ± 40	≥1 mM	453 ± 12
**7**	159 ± 41	529 ± 29	181 ± 41
**8**	134 ± 47	≥1 mM	85 ± 5
**9**	≥1 mM	≥1 mM	≥1 mM
**10**	87 ± 33	≥1 mM	88 ± 8
**11**	25 ± 4	232 ± 68	35 ± 5
**12**	88 ± 31	276 ± 46	92 ± 17
**13**	103 ± 8	546 ± 46	148 ± 17
**14**	≥1 mM	≥1 mM	≥1 mM

## Data Availability

The data presented in this study are available on request from the corresponding author.

## Supplementary Materials

The following supporting information can be downloaded at: https://www.mdpi.com/article/10.3390/molecules29225357/s1, general information about synthesis (synthetic procedures and spectral data), list of interactions between tested compounds and cholinesterases, differential UV/VIS spectra of the compounds–biometal mixtures, physiochemical properties of compounds and prediction of absorption through the human intestine. Table S1: Calculated physiochemical properties; Table S2: In silico estimated % of compounds that will be absorbed through the human intestine; Scheme S1: Synthesis of noncommercial 4-chloroquinolines **30**, **31**, **32**, **15**, **33** and **34**. Percentages denotes the yield; Scheme S2: Synthesis of noncommercial 4-chloroquinolines **34** and **35**; Scheme S3: Synthesis of amodiaquine derivatives **1**–**14**; Figures S1–S8: UV/Vis spectra of compounds, metals and compounds–biometal mixtures; Figures S9–S11: Differential UV/VIS spectra of the compounds-biometal mixtures; Reference [68] is cited in Supplementary Materials.
