# Supplementary material for: Derivatives of Amodiaquine as Potent Human Cholinesterases Inhibitors: Implication for Treatment of Alzheimer’s Disease
Source: Molecules. 2024 Nov 14;29(22):5357. doi: 10.3390/molecules29225357 (PMC11596630; doi:10.3390/molecules29225357)
Supplement: Supplementary file 1 [file molecules-29-05357-s001.zip › HPLC chromatograms_DO.pdf]

Sample Name: AMQ

Method A

90

Method : ACN.M (Sequence Method)

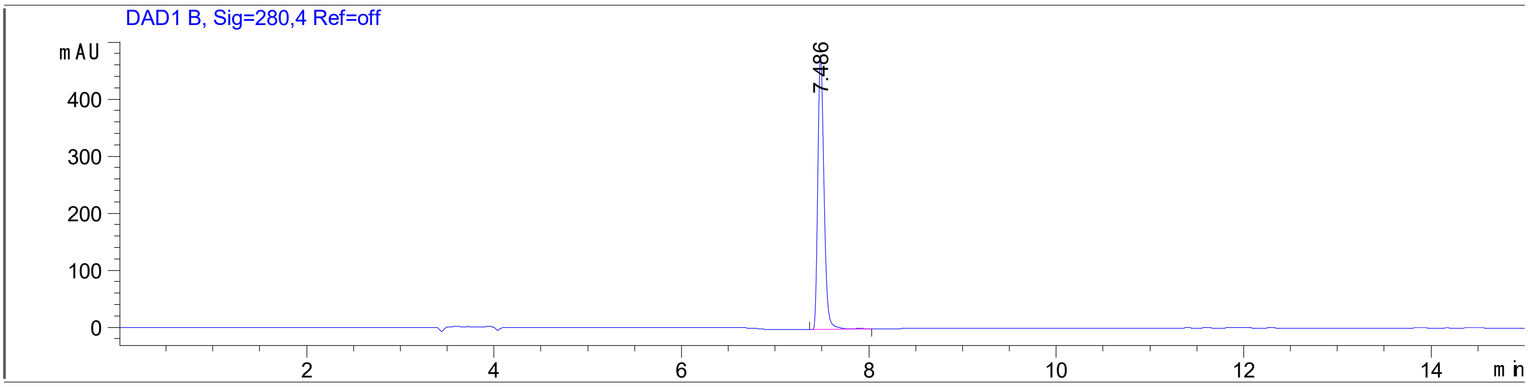

===

Area Percent Report

===

Signal 2: DAD1 B, Sig=280,4 Ref=off

Peak #	RetTime [min]	Type	Width [min]	Area [mAU*s]	Height [mAU]	Area %
1	7.486	BV R	0.0703	2157.53149	481.33957	100.0000

Totals : 2157.53149 481.33957

===

*** End of Report ***

Sample Name: AMQ

Method B

91

Method : MeOH.M (Sequence Method)

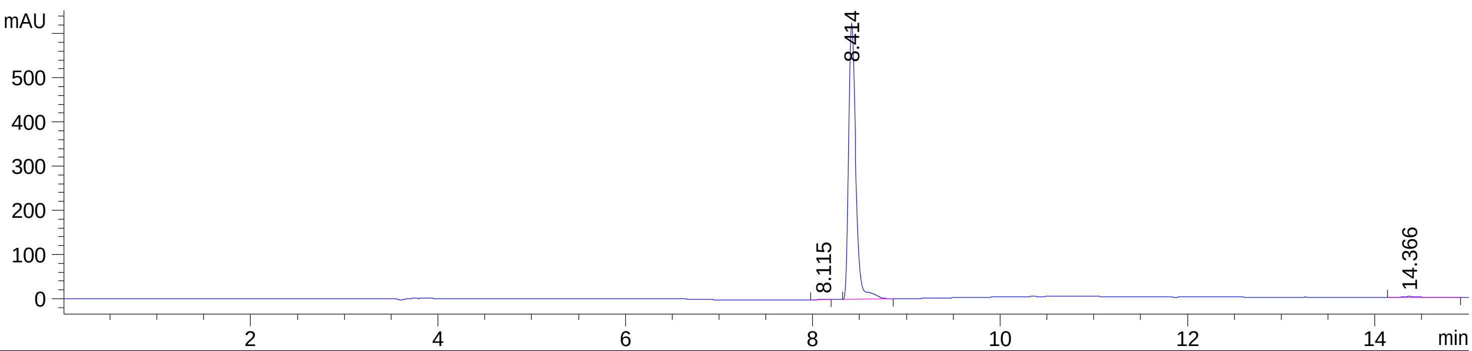

===

Area Percent Report

===

Signal 2: DAD1 B, Sig=280,4 Ref=off

Peak #	RetTime [min]	Type	Width [min]	Area [mAU*s]	Height [mAU]	Area %
1	8.115	BV	0.0880	6.40800	1.20492	0.1875
2	8.414	BB	0.0834	3394.65771	624.27399	99.3538
3	14.366	BB	0.1242	15.67020	1.82504	0.4586
Totals :				3416.73591	627.30394	

===

*** End of Report ***

Sample Name: 1

Method A

92

Method : ACN.M (Sequence Method)

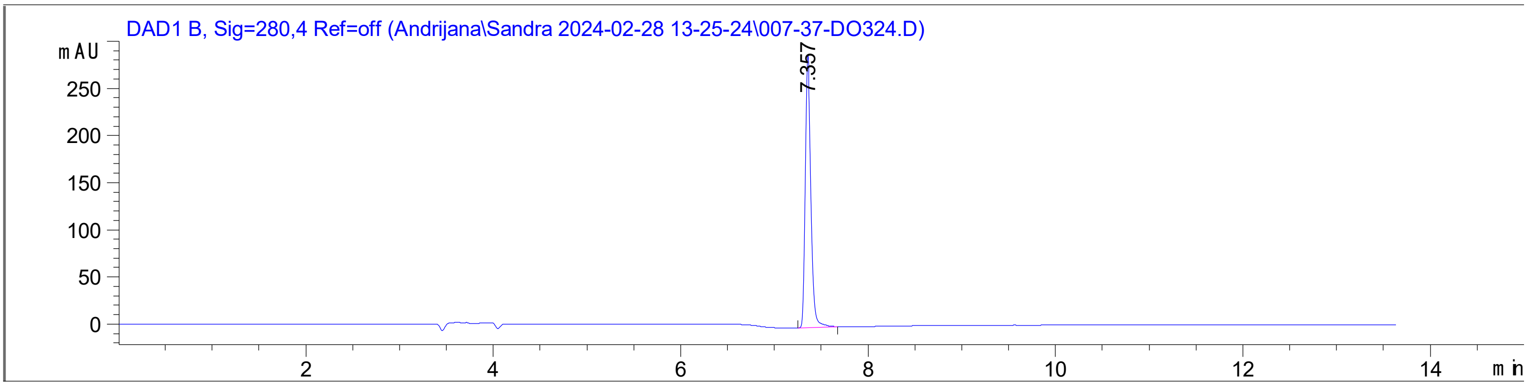

===

Area Percent Report

===

Signal 2: DAD1 B, Sig=280,4 Ref=off

Peak #	RetTime [min]	Type	Width [min]	Area [mAU*s]	Height [mAU]	Area %
1	7.357	BB	0.0667	1258.69543	289.60513	100.0000

Totals : 1258.69543 289.60513

===

*** End of Report ***

Sample Name: 1

Method B

93

Method : MeOH.M (Sequence Method)

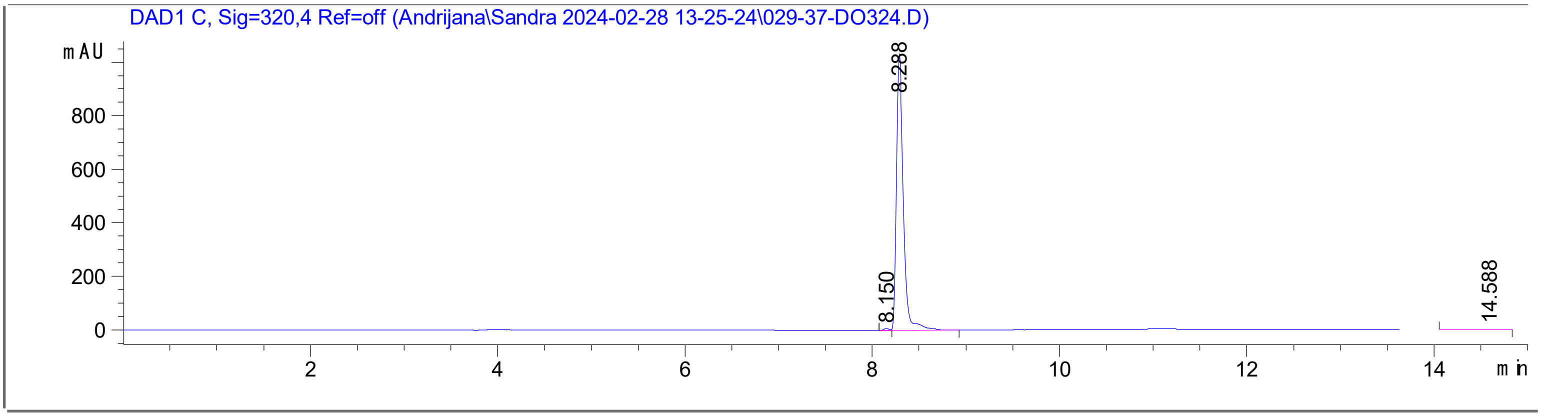

===
Area Percent Report
===

Signal 3: DAD1 C, Sig=320,4 Ref=off

Peak #	RetTime [min]	Type	Width [min]	Area [mAU*s]	Height [mAU]	Area %
1	8.150	BV E	0.0581	30.00755	7.94614	0.5451
2	8.288	VV R	0.0798	5460.11670	1030.77783	99.1805
3	14.588	BB	0.1187	15.10774	1.90171	0.2744

Totals : 5505.23198 1040.62568

===
*** End of Report ***

Sample Name: 2

Method A

94

Method : ACN.M (Sequence Method) A

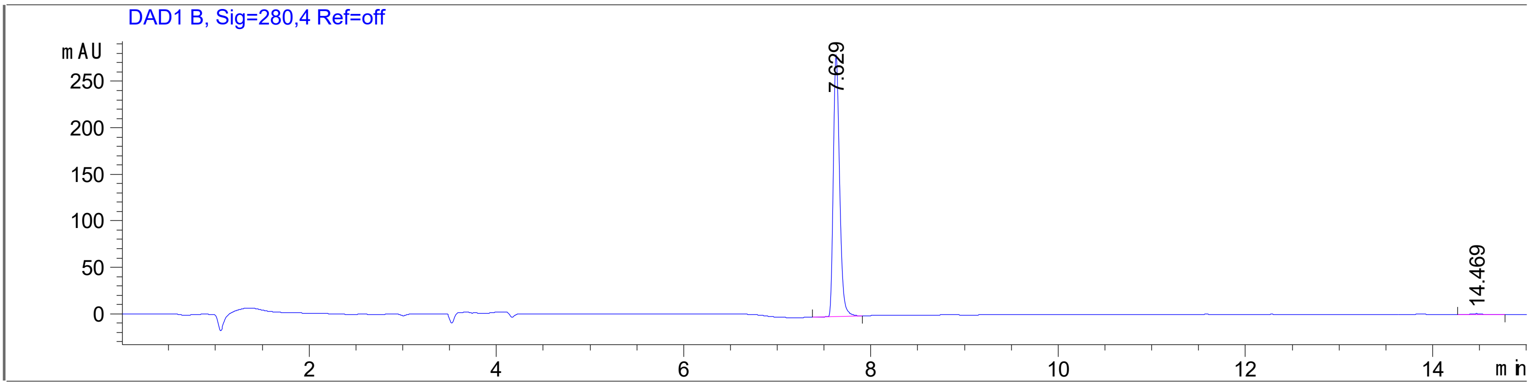

===

Area Percent Report

===

Signal 2: DAD1 B, Sig=280,4 Ref=off

Peak #	RetTime [min]	Type	Width [min]	Area [mAU*s]	Height [mAU]	Area %
1	7.629	BB	0.0710	1278.58997	281.56354	99.4315
2	14.469	BB	0.1089	7.31039	1.00470	0.5685

Totals : 1285.90036 282.56824

===

*** End of Report ***

Sample Name: 2

Method B

95

Method : MeOH.M (Sequence Method)

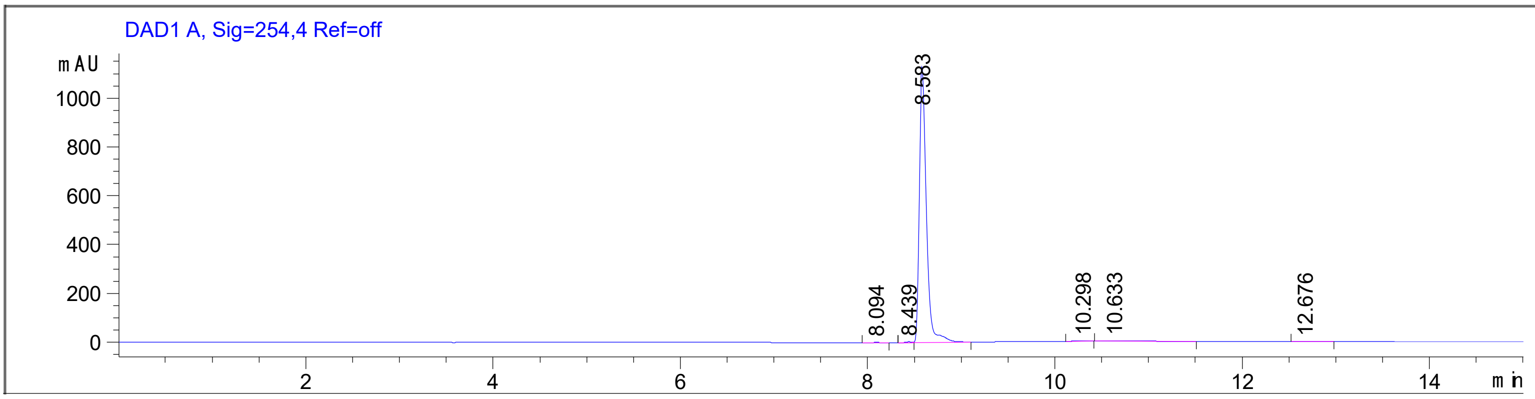

===

Area Percent Report

===

Signal 1: DAD1 A, Sig=254,4 Ref=off

Peak #	RetTime [min]	Type	Width [min]	Area [mAU*s]	Height [mAU]	Area %
1	8.094	BB	0.0984	8.65870	1.35849	0.1394
2	8.439	BV E	0.0620	12.36164	3.13572	0.1990
3	8.583	VB R	0.0832	6129.42871	1131.10828	98.6524
4	10.298	BB	0.1598	10.12848	1.03871	0.1630
5	10.633	BB	0.3023	35.84919	1.45050	0.5770
6	12.676	BB	0.1550	16.73088	1.46764	0.2693

Totals : 6213.15760 1139.55934

===

*** End of Report ***

Sample Name: 3

Method A

96

Method : ACN.M (Sequence Method)

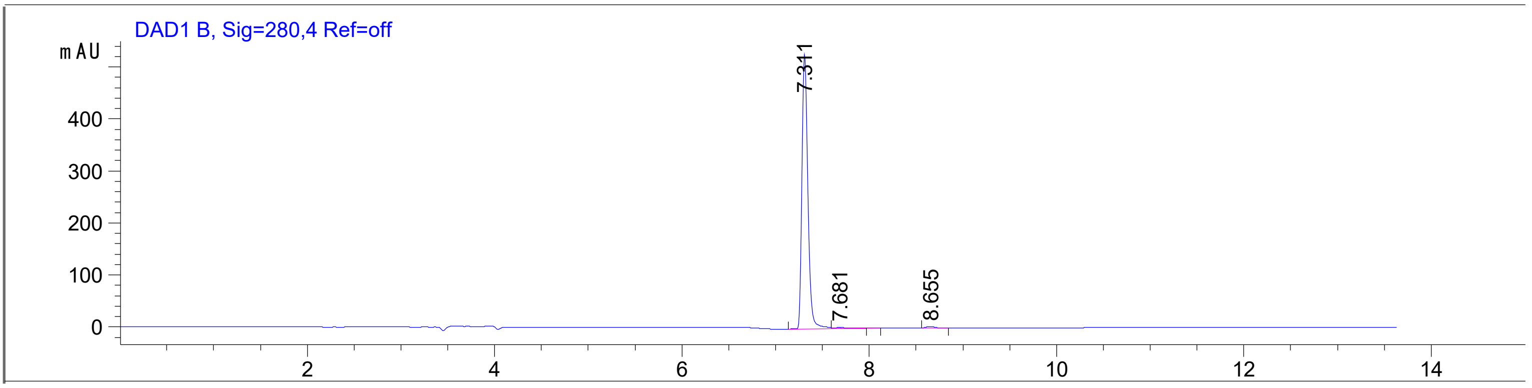

===

Area Percent Report

===

Signal 2: DAD1 B, Sig=280,4 Ref=off

Peak #	RetTime [min]	Type	Width [min]	Area [mAU*s]	Height [mAU]	Area %
1	7.311	VV R	0.0665	2285.81421	528.16888	98.5703
2	7.681	VV E	0.1406	17.28128	1.64398	0.7452
3	8.655	BB	0.0899	15.87219	2.80902	0.6845

Totals : 2318.96768 532.62188

===

*** End of Report ***

Sample Name: 3

Method B

97

Method : MeOH.M (Sequence Method)

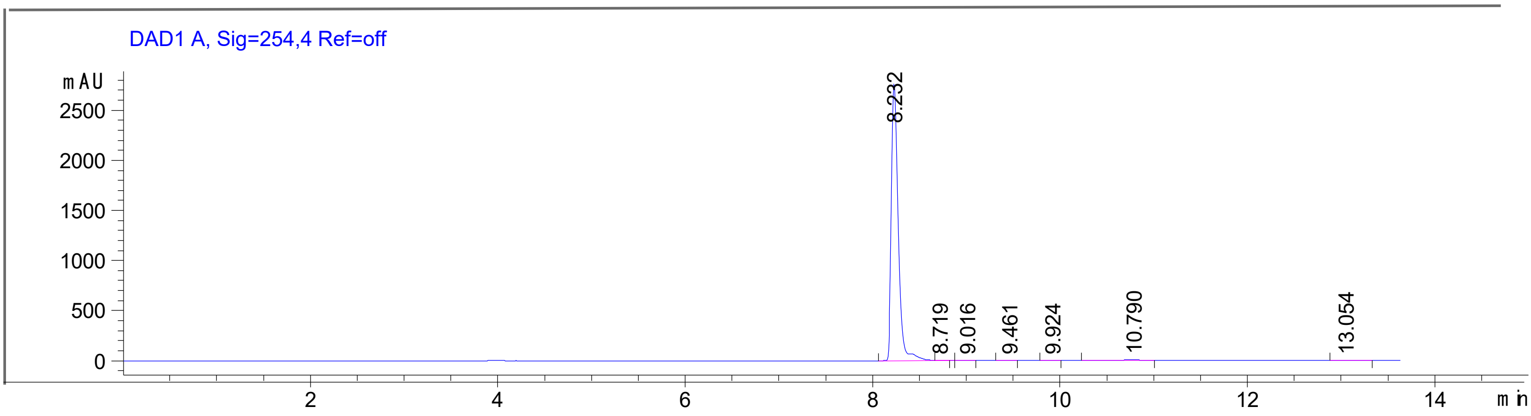

===

Area Percent Report

===

Signal 1: DAD1 A, Sig=254,4 Ref=off

Peak #	RetTime [min]	Type	Width [min]	Area [mAU*s]	Height [mAU]	Area %
1	8.232	BV R	0.0800	1.42204e4	2764.06592	99.1694
2	8.719	VB X	0.0759	16.40003	3.30611	0.1144
3	9.016	VB R	0.0649	10.54720	2.51744	0.0736
4	9.461	BV	0.0922	22.86393	3.69440	0.1594
5	9.924	BB	0.1081	7.08773	1.03297	0.0494
6	10.790	BB	0.1336	45.57909	4.60102	0.3179
7	13.054	BB	0.1544	16.63151	1.48848	0.1160

Totals : 1.43395e4 2780.70634

===

*** End of Report ***

Sample Name: 4

Method A

98

Method : ACN.M (Sequence Method)

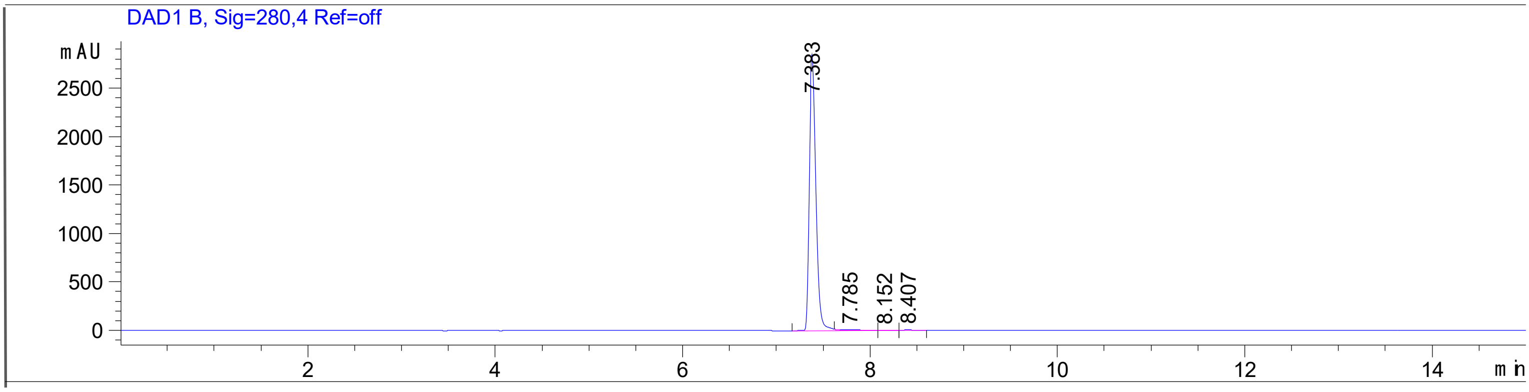

===

Area Percent Report

===

Signal 2: DAD1 B, Sig=280,4 Ref=off

Peak #	RetTime [min]	Type	Width [min]	Area [mAU*s]	Height [mAU]	Area %
1	7.383	BV R	0.0788	1.43533e4	2846.95288	98.7821
2	7.785	VV E	0.2224	121.74216	7.70243	0.8378
3	8.152	VB E	0.1110	16.70663	2.19085	0.1150
4	8.407	BB	0.0930	38.51042	6.70521	0.2650

Totals : 1.45303e4 2863.55137

===

*** End of Report ***

Sample Name: 4

Method B

99

Method : MeOH (Sequence Method)

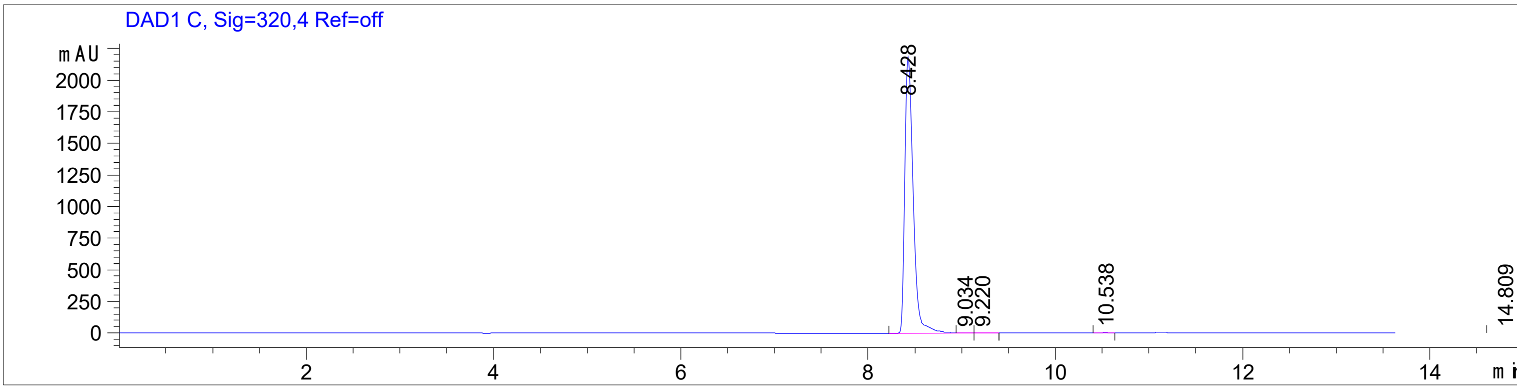

===

Area Percent Report

===

Signal 3: DAD1 C, Sig=320,4 Ref=off

Peak #	RetTime [min]	Type	Width [min]	Area [mAU*s]	Height [mAU]	Area %
1	8.428	BV R	0.0955	1.33865e4	2183.30103	99.6591
2	9.034	VV E	0.0777	6.22506	1.17620	0.0463
3	9.220	VB E	0.0759	17.91619	3.49055	0.1334
4	10.538	BB	0.0869	8.70708	1.43290	0.0648
5	14.809	BBA	0.1070	12.94117	1.81938	0.0963

Totals : 1.34323e4 2191.22007

===

*** End of Report ***

Sample Name: 5

Method A

100

Method : .ACN.M (Sequence Method)

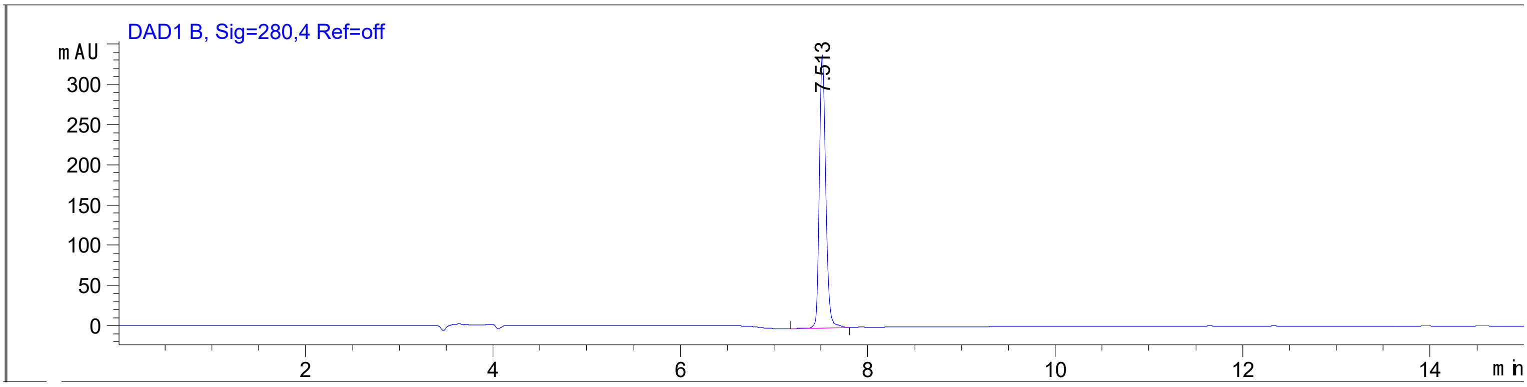

==

Area Percent Report

==

Signal 2: DAD1 B, Sig=280,4 Ref=off

Peak #	RetTime [min]	Type	Width [min]	Area [mAU*s]	Height [mAU]	Area %
1	7.513	BB	0.0720	1575.07678	340.23230	100.0000

otals : 1575.07678 340.23230

==

*** End of Report ***

Sample Name: 5

Method B

101

Method MeOH.M (Sequence Method)

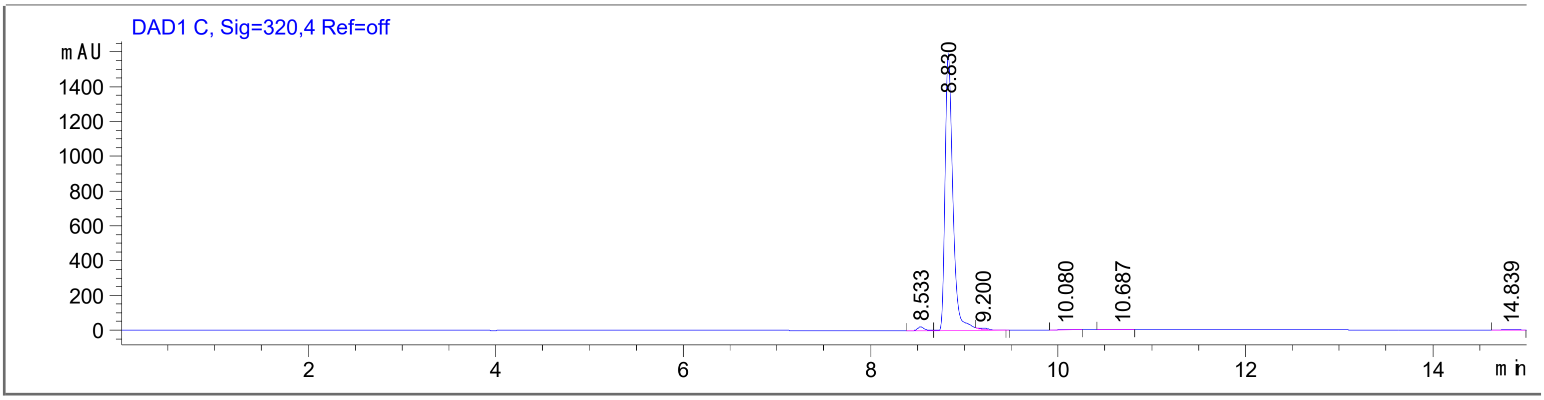

===

Area Percent Report

===

Signal 3: DAD1 C, Sig=320,4 Ref=off

Peak #	RetTime [min]	Type	Width [min]	Area [mAU*s]	Height [mAU]	Area %
1	8.533	BV	0.0709	98.59688	20.94725	1.0742
2	8.830	VV R	0.0862	8989.28613	1584.87781	97.9337
3	9.200	VB E	0.0991	45.37307	6.86552	0.4943
4	10.080	BB	0.1079	24.22400	3.45020	0.2639
5	10.687	BB	0.0957	9.09399	1.33036	0.0991
6	14.839	BBA	0.1076	12.37879	1.77035	0.1349

Totals : 9178.95285 1619.24149

===

*** End of Report ***

Sample Name: 6

Method A

102

Method : ACN.M (Sequence Method)

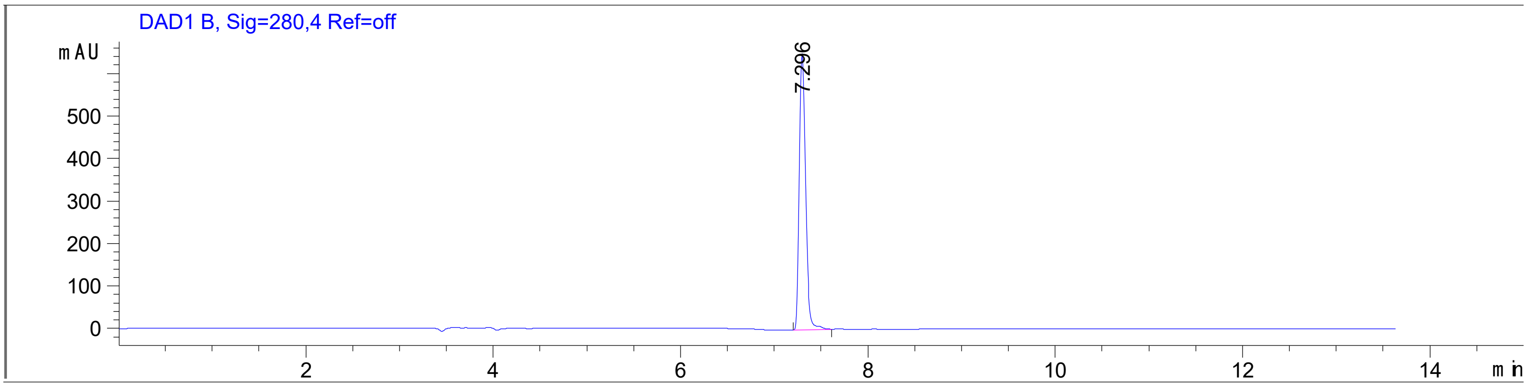

===

Area Percent Report

===

Signal 2: DAD1 B, Sig=280,4 Ref=off

Peak #	RetTime [min]	Type	Width [min]	Area [mAU*s]	Height [mAU]	Area %
1	7.296	BB	0.0730	3048.08691	646.96820	100.0000

Totals : 3048.08691 646.96820

===

*** End of Report ***

Sample Name: 6

Method B

103

Method : MeOH.M (Sequence Method)

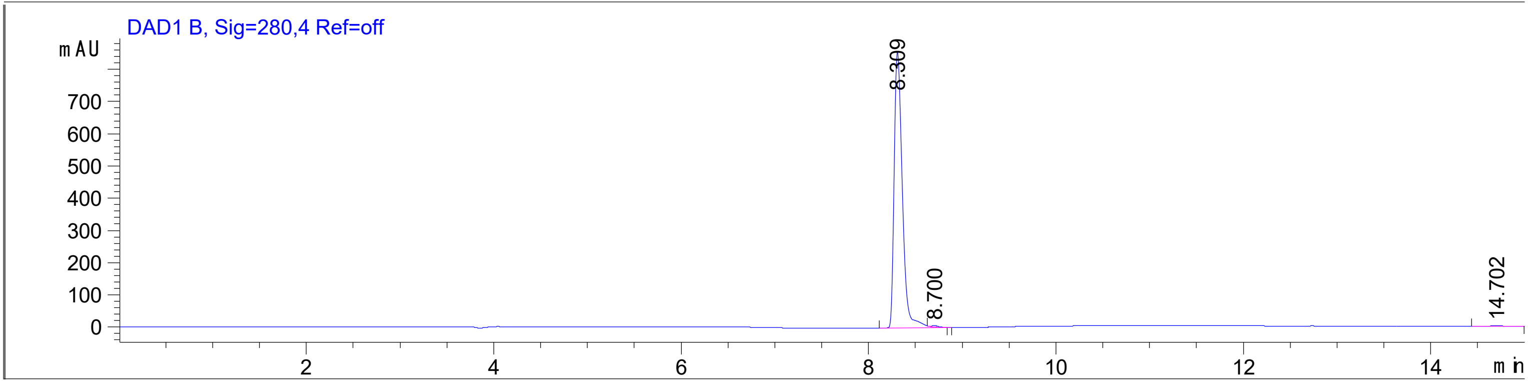

===

Area Percent Report

===

Signal 2: DAD1 B, Sig=280,4 Ref=off

Peak #	RetTime [min]	Type	Width [min]	Area [mAU*s]	Height [mAU]	Area %
1	8.309	BV R	0.0911	5082.34082	858.26489	99.2895
2	8.700	VB E	0.0769	23.07997	4.42484	0.4509
3	14.702	BBA	0.1155	13.29060	1.69434	0.2596

Totals : 5118.71139 864.38407

===

*** End of Report ***

Sample Name: 7

Method A

104

Method : ACN.M (Sequence Method)

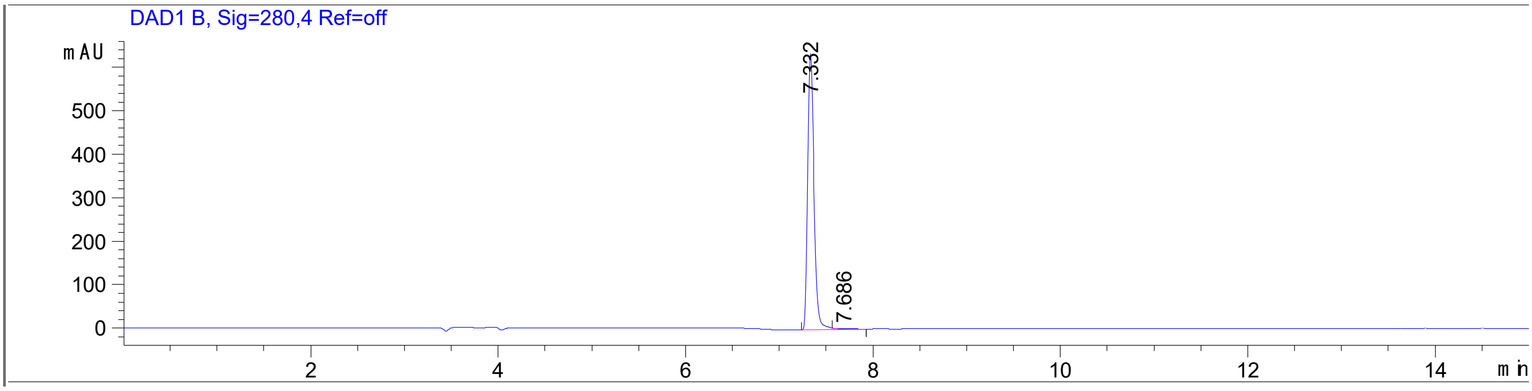

===

Area Percent Report

===

Signal 2: DAD1 B, Sig=280,4 Ref=off

Peak #	RetTime [min]	Type	Width [min]	Area [mAU*s]	Height [mAU]	Area %
1	7.332	BV R	0.0743	3053.47339	632.49554	99.5463
2	7.686	VB E	0.1346	13.91534	1.34588	0.4537

Totals : 3067.38873 633.84143

===

*** End of Report ***

Sample Name: 7

Method B

105

Method : MeOH.M (Sequence Method) A

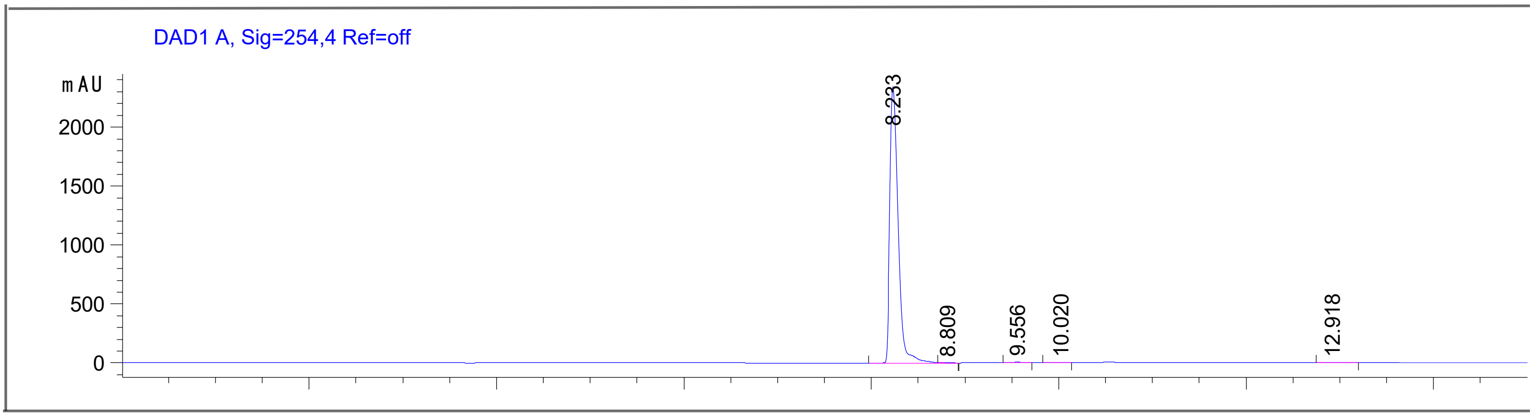

===

Area Percent Report

===

Signal 1: DAD1 A, Sig=254,4 Ref=off

Peak #	RetTime [min]	Type	Width [min]	Area [mAU*s]	Height [mAU]	Area %
1	8.233	VV R	0.0964	1.45129e4	2339.25806	99.5684
2	8.809	VB E	0.0734	9.80896	1.99526	0.0673
3	9.556	BB	0.0911	26.98314	4.42626	0.1851
4	10.020	BB	0.0955	10.00762	1.46855	0.0687
5	12.918	BB	0.1537	16.10632	1.44900	0.1105

Totals : 1.45758e4 2348.59713

===

*** End of Report ***

Sample Name: 8

Method A

106

Method : ACN.M (Sequence Method)

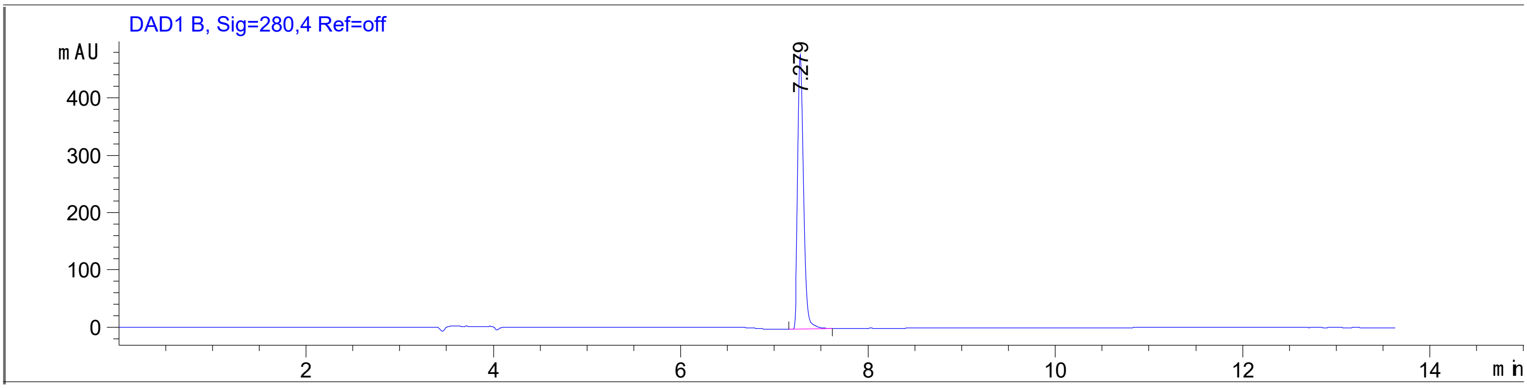

===

Area Percent Report

===

Signal 2: DAD1 B, Sig=280,4 Ref=off

Peak #	RetTime [min]	Type	Width [min]	Area [mAU*s]	Height [mAU]	Area %
1	7.279	BB	0.0670	2092.74414	479.14780	100.0000

Totals : 2092.74414 479.14780

===

*** End of Report ***

Sample Name: 8

Method B

107

Method : MeOH.M (Sequence Method)

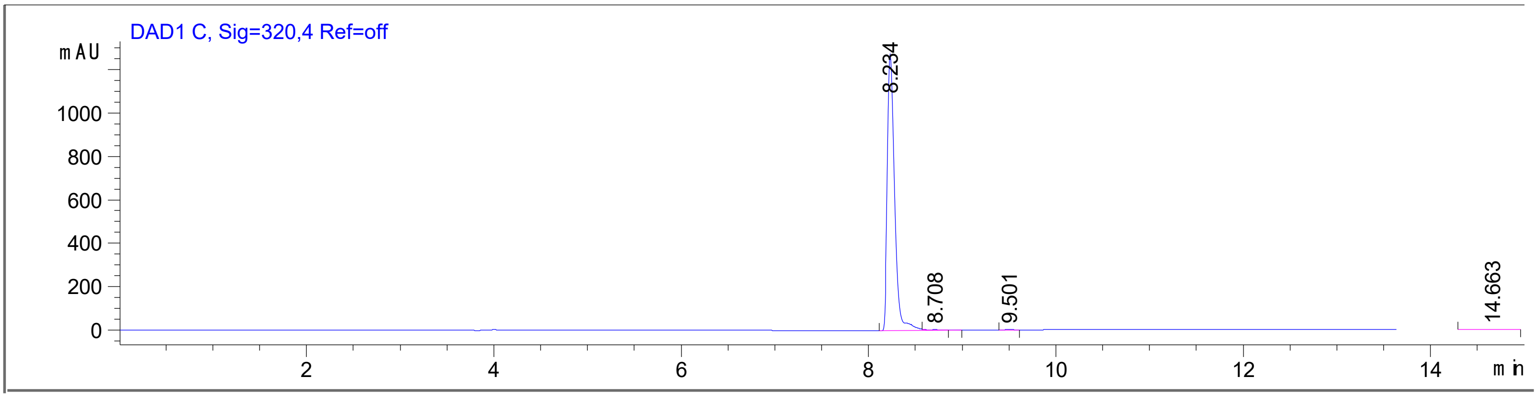

===

Area Percent Report

===

Signal 3: DAD1 C, Sig=320,4 Ref=off

Peak #	RetTime [min]	Type	Width [min]	Area [mAU*s]	Height [mAU]	Area %
1	8.234	BV R	0.0835	6912.77783	1271.03882	99.2670
2	8.708	VV E	0.0920	20.87429	3.20530	0.2998
3	9.501	BB	0.0803	15.35146	2.97103	0.2204
4	14.663	BBA	0.1139	14.82143	1.92342	0.2128

Totals : 6963.82502 1279.13856

===

*** End of Report ***

Sample Name: 9

Method A

108

Method : ACN.M (Sequence Method)

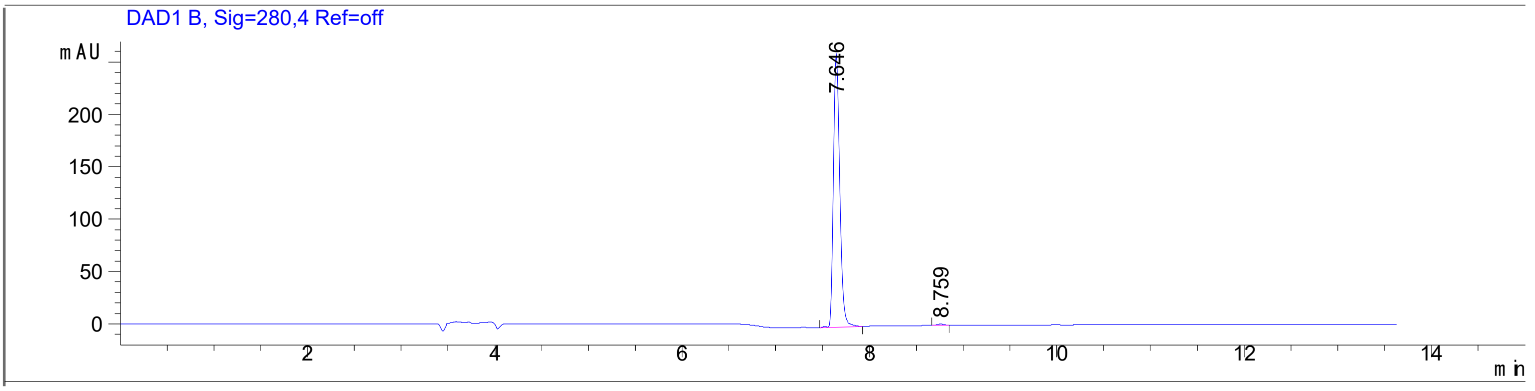

===

Area Percent Report

===

Signal 2: DAD1 B, Sig=280,4 Ref=off

Peak #	RetTime [min]	Type	Width [min]	Area [mAU*s]	Height [mAU]	Area %
1	7.646	VB R	0.0716	1195.32434	259.96664	99.5400
2	8.759	BB	0.0785	5.52332	1.14025	0.4600

Totals : 1200.84767 261.10689

===

*** End of Report ***

Sample Name: 9

Method B

109

Method : MeOH.M (Sequence Method)

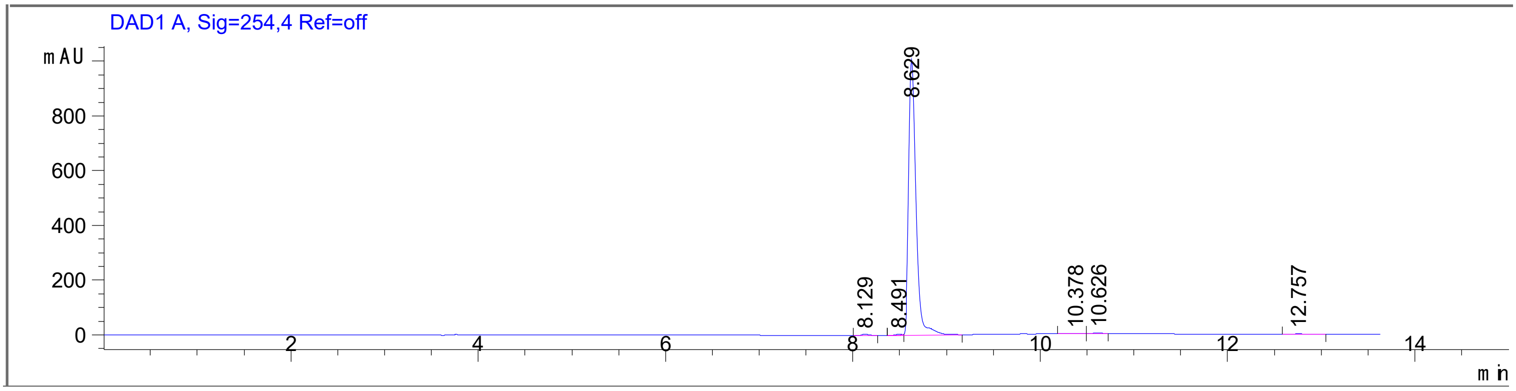

===

Area Percent Report

===

Signal 1: DAD1 A, Sig=254,4 Ref=off

Peak #	RetTime [min]	Type	Width [min]	Area [mAU*s]	Height [mAU]	Area %
1	8.129	BB	0.0674	24.87801	5.44026	0.4482
2	8.491	BV E	0.0620	17.96108	4.55456	0.3236
3	8.629	VB R	0.0833	5465.30566	1006.87225	98.4612
4	10.378	BV	0.1617	13.91286	1.37864	0.2506
5	10.626	VB	0.1020	11.03903	1.53317	0.1989
6	12.757	BB	0.1544	17.62180	1.55241	0.3175

Totals : 5550.71845 1021.33129

===

*** End of Report ***

Sample Name: 10

Method A

110

Method : ACN.M (Sequence Method)

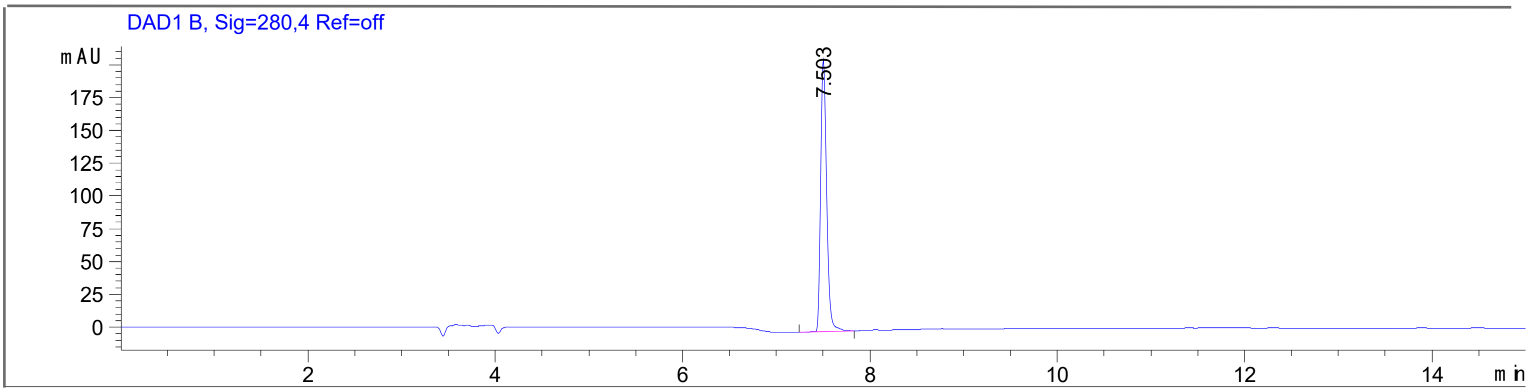

===

Area Percent Report

===

Signal 2: DAD1 B, Sig=280,4 Ref=off

Peak #	RetTime [min]	Type	Width [min]	Area [mAU*s]	Height [mAU]	Area %
1	7.503	BB	0.0667	901.14380	207.55090	100.0000

Totals : 901.14380 207.55090

===

*** End of Report ***

Sample Name: 10

Method B

111

Method : MeOH.M (Sequence Method)

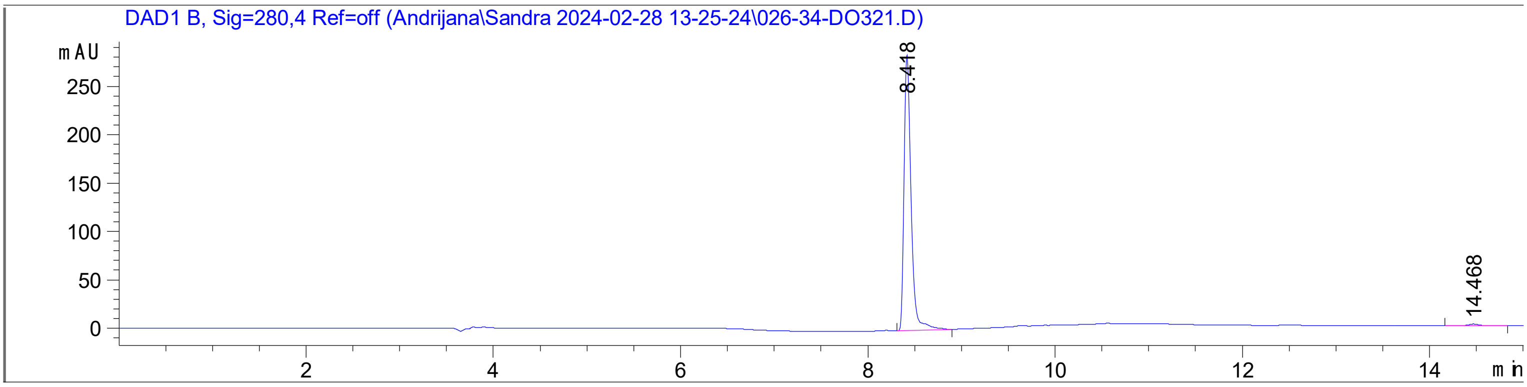

===

Area Percent Report

===

Signal 2: DAD1 B, Sig=280,4 Ref=off

Peak #	RetTime [min]	Type	Width [min]	Area [mAU*s]	Height [mAU]	Area %
1	8.418	BB	0.0773	1450.48242	285.22501	98.9577
2	14.468	BB	0.1224	15.27784	1.84834	1.0423

Totals : 1465.76027 287.07335

===

*** End of Report ***

Sample Name: 11

Method A

112

Method : ACN.M (Sequence Method)

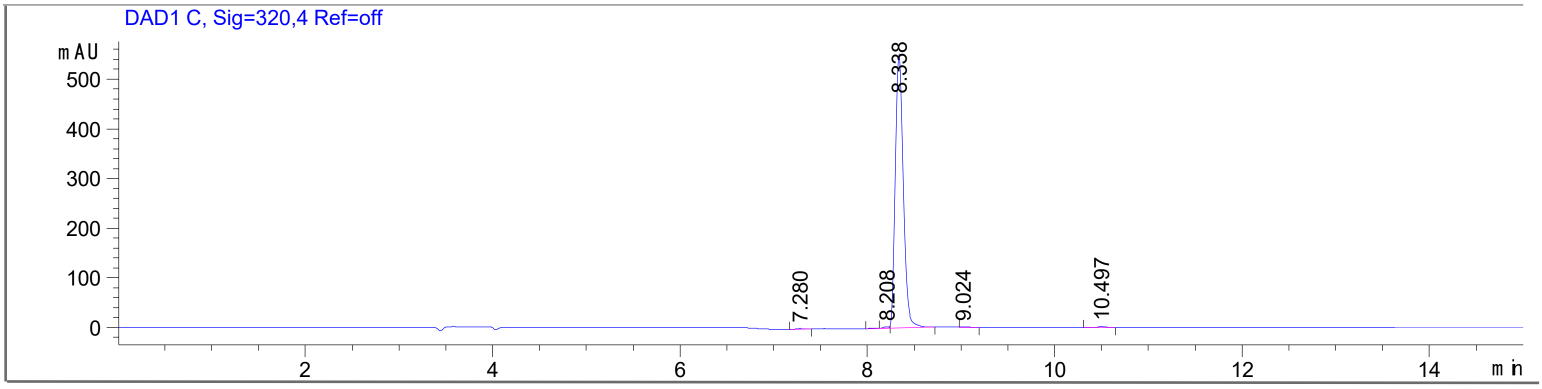

==

Area Percent Report

==

_ignal 3: DAD1 C, Sig=320,4 Ref=off

Peak #	RetTime [min]	Type	Width [min]	Area [mAU*s]	Height [mAU]	Area %
1	7.280	BB	0.0697	6.30192	1.27335	0.1935
2	8.208	VV E	0.0650	11.77019	2.92588	0.3615
3	8.338	VB R	0.0922	3218.68286	550.29974	98.8497
4	9.024	VB	0.0831	7.12713	1.23836	0.2189
5	10.497	BB	0.0832	12.25462	2.26141	0.3764

Totals : 3256.13672 557.99875

==

*** End of Report ***

Sample Name: 11

Method B

113

Method : MeOH.M (Sequence Method)

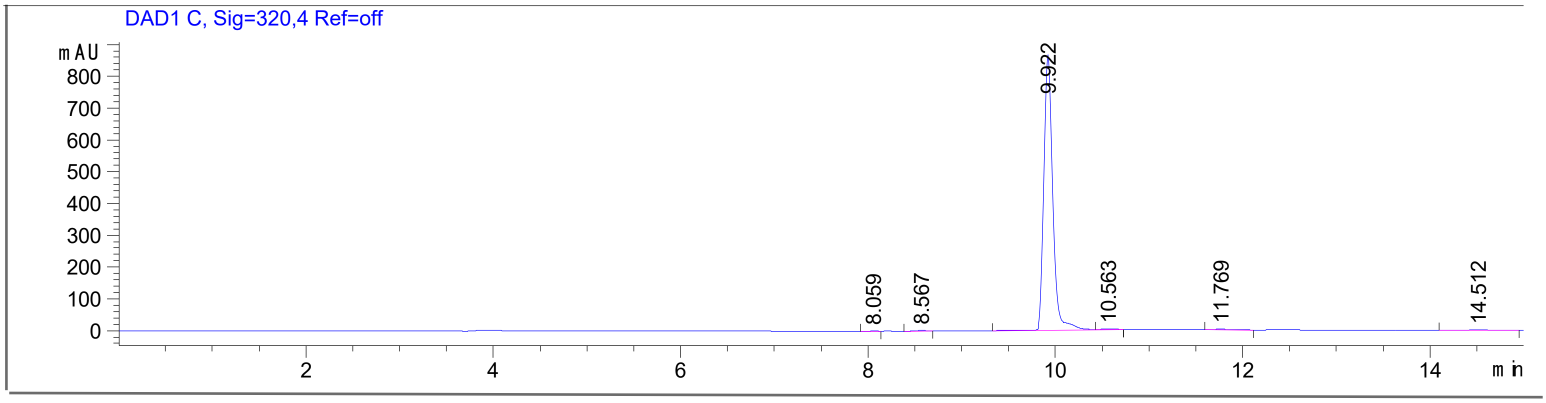

===
Area Percent Report
===

Signal 3: DAD1 C, Sig=320,4 Ref=off

Peak #	RetTime [min]	Type	Width [min]	Area [mAU*s]	Height [mAU]	Area %
1	8.059	BV	0.0662	8.54954	1.91205	0.1500
2	8.567	BB	0.0984	16.00469	2.58012	0.2809
3	9.922	BV R	0.0978	5611.38135	863.13104	98.4731
4	10.563	VB E	0.1104	20.81923	2.52056	0.3654
5	11.769	BB	0.1421	23.85035	2.57416	0.4185
6	14.512	BB	0.1280	17.78314	2.03306	0.3121

Totals : 5698.38829 874.75099

===
*** End of Report ***

Sample Name: 12

Method A

114

Method : ACN.M (Sequence Method)

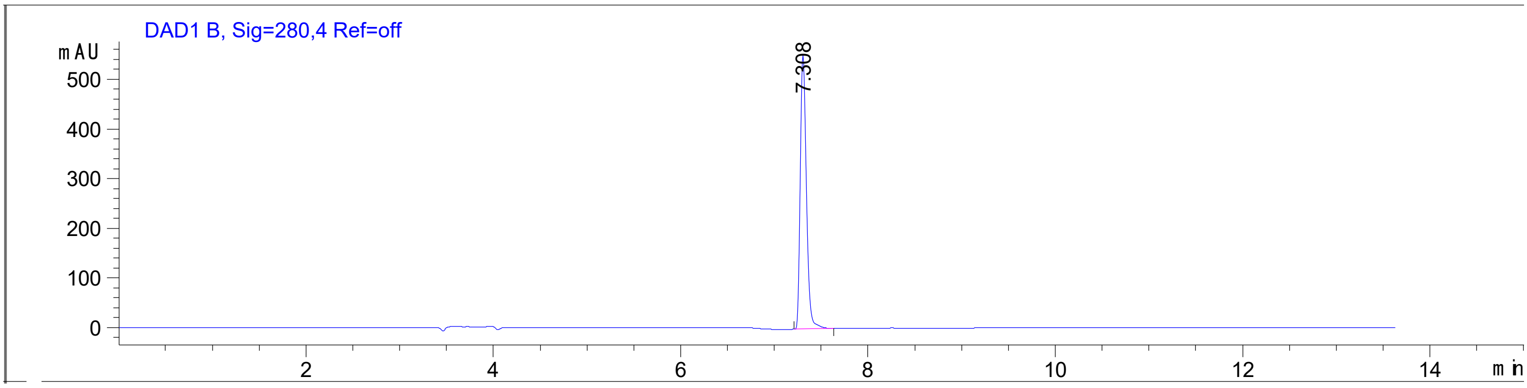

===

Area Percent Report

===

Signal 2: DAD1 B, Sig=280,4 Ref=off

Peak #	RetTime [min]	Type	Width [min]	Area [mAU*s]	Height [mAU]	Area %
1	7.308	BB	0.0699	2455.50830	552.14471	100.0000

Totals : 2455.50830 552.14471

===

*** End of Report ***

Sample Name: 12

Method B

115

Method : MeOH.M (Sequence Method)

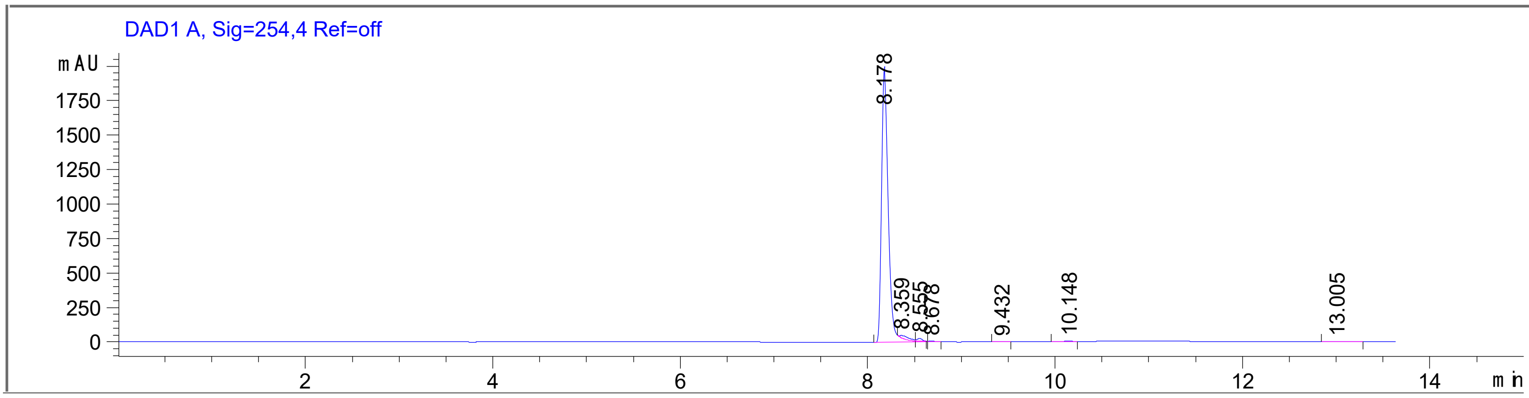

===

Area Percent Report

===

Signal 1: DAD1 A, Sig=254,4 Ref=off

Peak #	RetTime [min]	Type	Width [min]	Area [mAU*s]	Height [mAU]	Area %
1	8.178	BV R	0.0782	1.03258e4	1999.96277	97.3866
2	8.359	VV E	0.1323	156.76428	16.27980	1.4785
3	8.555	VB E	0.0605	76.11192	19.10123	0.7178
4	8.678	BB	0.0602	11.11351	2.80611	0.1048
5	9.432	BB	0.0786	10.72933	2.13486	0.1012
6	10.148	BB	0.1027	7.36071	1.11937	0.0694
7	13.005	BB	0.1507	15.01360	1.36131	0.1416

Totals : 1.06029e4 2042.76544

===

*** End of Report ***

Sample Name: 13

Method A

116

Method : ACN.M (Sequence Method)

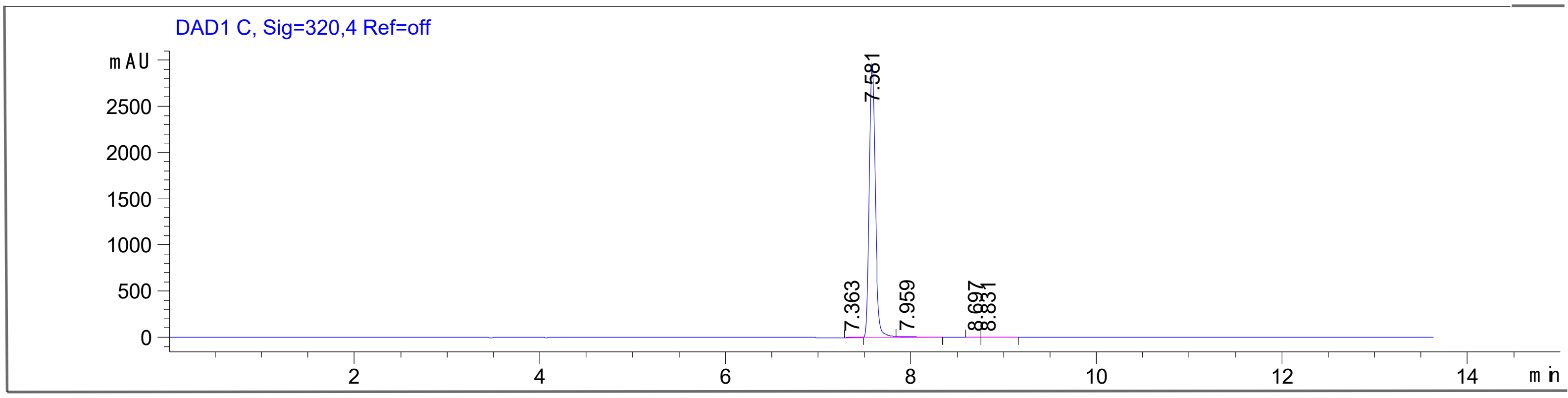

===

Area Percent Report

===

Signal 3: DAD1 C, Sig=320,4 Ref=off

Peak #	RetTime [min]	Type	Width [min]	Area [mAU*s]	Height [mAU]	Area %
1	7.363	BV E	0.0833	38.12730	6.41929	0.2778
2	7.581	VV R	0.0713	1.35540e4	2967.00317	98.7638
3	7.959	VB E	0.1521	96.90786	8.69258	0.7061
4	8.697	BV	0.0883	7.33951	1.33096	0.0535
5	8.831	VB	0.0945	27.27133	4.16165	0.1987

Totals : 1.37237e4 2987.60765

===

*** End of Report ***

Sample Name: 13

Method B

117

Method : MeOH.M (Sequence Method)

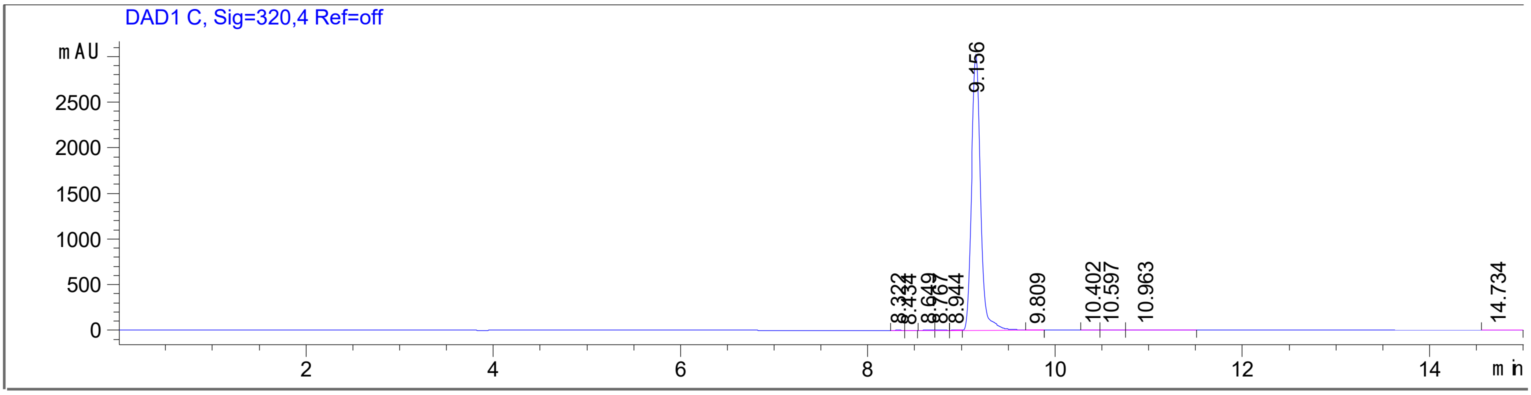

===

Area Percent Report

===

Signal 3: DAD1 C, Sig=320,4 Ref=off

Peak #	RetTime [min]	Type	Width [min]	Area [mAU*s]	Height [mAU]	Area %
1	8.322	BV	0.0668	17.21681	3.95514	0.0837
2	8.434	VB	0.0729	6.15645	1.26136	0.0299
3	8.649	BV	0.0753	23.19584	4.41521	0.1128
4	8.767	VB	0.0741	15.06913	3.02649	0.0733
5	8.944	BV E	0.0603	10.55233	2.77945	0.0513
6	9.156	VV R	0.1049	2.04207e4	3020.41089	99.3287
7	9.809	VB E	0.0922	7.76962	1.36921	0.0378
8	10.402	BV	0.1111	6.99336	1.03212	0.0340
9	10.597	VB	0.0992	10.31569	1.44668	0.0502
10	10.963	BB	0.2527	27.64759	1.53087	0.1345
11	14.734	BBA	0.1102	13.08613	1.81408	0.0637

Totals : 2.05587e4 3043.04148

===

*** End of Report ***

Sample Name: 14

Method A

118

Method : ACN.M (Sequence Method)

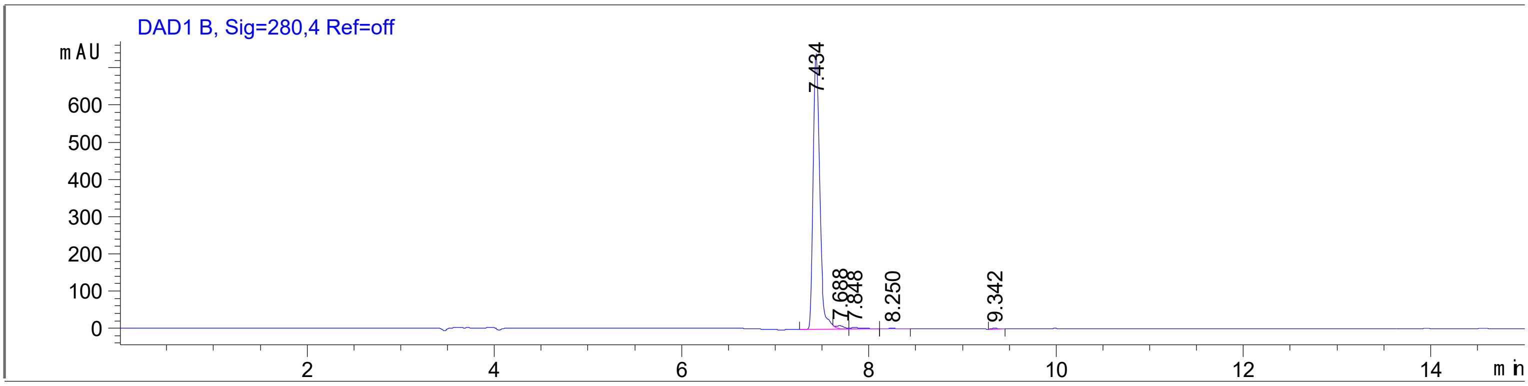

===

Area Percent Report

===

Signal 2: DAD1 B, Sig=280,4 Ref=off

Peak #	RetTime [min]	Type	Width [min]	Area [mAU*s]	Height [mAU]	Area %
1	7.434	BV R	0.0755	3639.38232	738.72607	97.4320
2	7.688	VV E	0.0829	42.61301	7.42657	1.1408
3	7.848	VB E	0.1237	36.85285	4.06752	0.9866
4	8.250	BB	0.0933	7.25574	1.25723	0.1942
5	9.342	BB	0.0764	9.20218	1.83737	0.2464

Totals : 3735.30610 753.31476

===

*** End of Report ***

Sample Name: 14

Method B

119

Method : MeOH.M (Sequence Method)

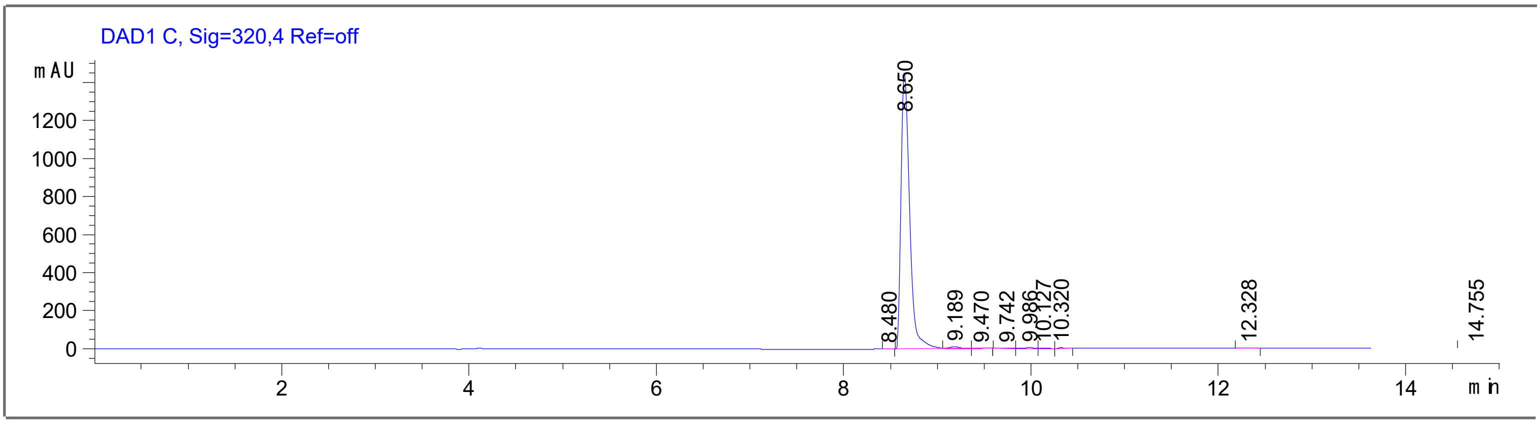

===

Area Percent Report

===

Signal 3: DAD1 C, Sig=320,4 Ref=off

Peak #	RetTime [min]	Type	Width [min]	Area [mAU*s]	Height [mAU]	Area %
1	8.480	BB	0.0575	8.35777	2.34762	0.0899
2	8.650	BV R	0.0970	9075.36426	1449.96948	97.6477
3	9.189	VV E	0.1137	73.42767	9.77071	0.7901
4	9.470	VB E	0.0977	17.75094	2.81081	0.1910
5	9.742	BV	0.1150	11.96080	1.49967	0.1287
6	9.986	VV	0.1195	41.61779	5.08463	0.4478
7	10.127	VB	0.1287	25.35429	3.26247	0.2728
8	10.320	BB	0.0747	20.64657	4.24477	0.2221
9	12.328	BB	0.0932	6.87218	1.09574	0.0739
10	14.755	BBA	0.1085	12.63123	1.78591	0.1359

Totals : 9293.98348 1481.87182

===

*** End of Report ***
