# Supplementary material for: Derivatives of Amodiaquine as Potent Human Cholinesterases Inhibitors: Implication for Treatment of Alzheimer’s Disease
Source: Molecules. 2024 Nov 14;29(22):5357. doi: 10.3390/molecules29225357 (PMC11596630; doi:10.3390/molecules29225357)
Supplement: Supplementary file 1 [file molecules-29-05357-s001.zip › molecules-3262787-supplementary.pdf]

Derivatives of amodiaquine as potent human cholinesterases inhibitors: implication for treatment of Alzheimer's disease

Ana Matošević[1], Dejan M. Opsenica[2,3,*], Marija Bartolić[1], Nikola Maraković[1], Andriana Stoilković[2], Katarina Komatović[4], Antonio Zandona[1], Suzana Žunec[1], Anita Bosak[1,*]

[1] Institute for Medical Research and Occupational Health, 10000 Zagreb, Croatia

[2] Institute of Chemistry Technology and Metallurgy, University of Belgrade, 11000 Beograd, Serbia

[3] Centre of Excellence in Environmental Chemistry and Engineering, 11000 Belgrade, Serbia

[4] Faculty of Chemistry, University of Belgrade, 11158 Belgrade, Serbia

* Corresponding author:

Dr Dejan Opsenica, University of Belgrade Institute of Chemistry, Technology and Metallurgy, Studentski trg 12-16, 11000 Beograd, Serbia; e-mail: dopsen@chem.bg.ac.rs

Dr Anita Bosak, Institute for Medical Research and Occupational Health, POBox 291, Ksaverska cesta 2, 10000 Zagreb, Croatia; e-mail: abosak@imi.hr

Table of contents

1. Synthesis S3

1.1. General information S3

1.2. Synthetic procedures and spectral data S4

1.2.1. Synthesis of starting noncommercial 4-chloroquinolines S4

Scheme S1 S4

Scheme S2 S4

1.2.2. Synthesis of AMQ and amodiaquine derivatives **1 – 14** S10

Scheme S3 S10

1.2.3. ^{1}H and ^{13}C NMR spectra S20

1.2.4. HRMS spectra of the tested compounds S90

2. List of interactions between tested compounds and cholinesterases S106

2.1. List of interactions between derivatives **1** – **14** and AChE S106

2.2. List of interactions between derivatives **1** – **14** and BChE S110

3. Metal chelation study

3.1. UV/Vis spectra of compounds, metals and compounds-biometal mixtures S114

3.2. Differential UV/VIS spectra of the compounds-biometal mixtures S122

4. *In silico* calculations S125

Table S1. Calculated physiochemical properties S125

Table S2. *In silico* estimated % of compounds that will be absorbed through the human intestine S126

References S126

1. Synthesis

1.1. General information

Melting points were recorded on an electrothermal melting point apparatus (1428 6/4 457), England. IR spectra were recorded on a Perkin-Elmer spectrophotometer FT-IR 1725X (Perkin-Elmer, Waltham, MA 02451, USA). Positions of absorbance bands are expressed in cm^{-1}, and intensity is labelled as w (weak), m (medium) or s (strong). ^{1}H and ^{13}C NMR spectra were recorded on a Varian spectrometer (Agilent Technology, Santa Clara, CA 95051, USA) (at 400 and 100 MHz for the ^{1}H and ^{13}C NMR spectra, respectively) and on a Bruker Ultrashield Advance III spectrometer (Bruker Scientific Instruments, Billerica, MA 01821, USA) (at 500 and 125 MHz for the ^{1}H and ^{13}C NMR spectra, respectively) employing indicated solvents using TMS as the internal standard. Chemical shifts are expressed in ppm (δ) values and coupling constants (J) in Hz. Mass spectra were recorded on a 4800 Plus MALDI TOF/TOF Analyzer (Applied Biosystems, US instrument, Mundelein, USA) in positive ion mode (fixed laser intensity 4280) using a CH_3CN/H_2O gradient with 0.2% HCOOH as the carrying solvent solution. Samples were dissolved in MeOH (HPLC grade purity) mixed with CHCA (α-Cyano-4-hydroxycinnamic acid) MALDI matrix (5 mg/mL in 50% acetonitrile (v/v)). Internal calibrants: Azithromycin and Azithromycin fragments (m/z 156-749).

HPLC purity: Purity of compounds was analyzed using an Agilent 1260 HPLC system equipped with Quat Pump (G1311B), InJector (G1329B) 1260 ALS, TCC 1260 (G1316A) and Detector 1260 DAD VL+ (G1315C), using 1.0 mL/min flow rate. Compounds (0.5 mg/mL) were dissolved in MeOH. The analysis was performed at the UV max of the compounds to maximize selectivity in two diverse systems:

Method A: Column Zorbax Eclipse (XDB-C18, 4.6 × 150 nm, 5.0 µm, S.N. USKBM01053) was used as the stationary phase. Eluent was made from the following solvents: 0.2% formic acid in water (A) and acetonitrile (B). Compounds were eluted using gradient protocol: 0 – 1 min 95% A, 1 − 6 min 95% A→ 5% A, 6 − 11 min 5% A, 11 – 14 min 5% A→ 95% A, 14 – 15 min 95% A.

Method B: Column Zorbax Eclipse (XDB-C18, 4.6 × 150 nm, 5.0 µm, S.N. USKBM01053) was used as the stationary phase. Eluent was made from the following solvents: 0.2% formic acid in water (A) and methanol (B). Compounds were eluted using gradient protocol: 0 – 1 min 95% A, 1 − 6 min 95% A→ 5% A, 6 − 11 min 5% A, 11 – 14 min 5% A→ 95% A, 14 – 15 min 95% A.

1.2. Synthetic procedures and spectral data

1.2.1. Synthesis of starting noncommercial 4-chloroquinolines

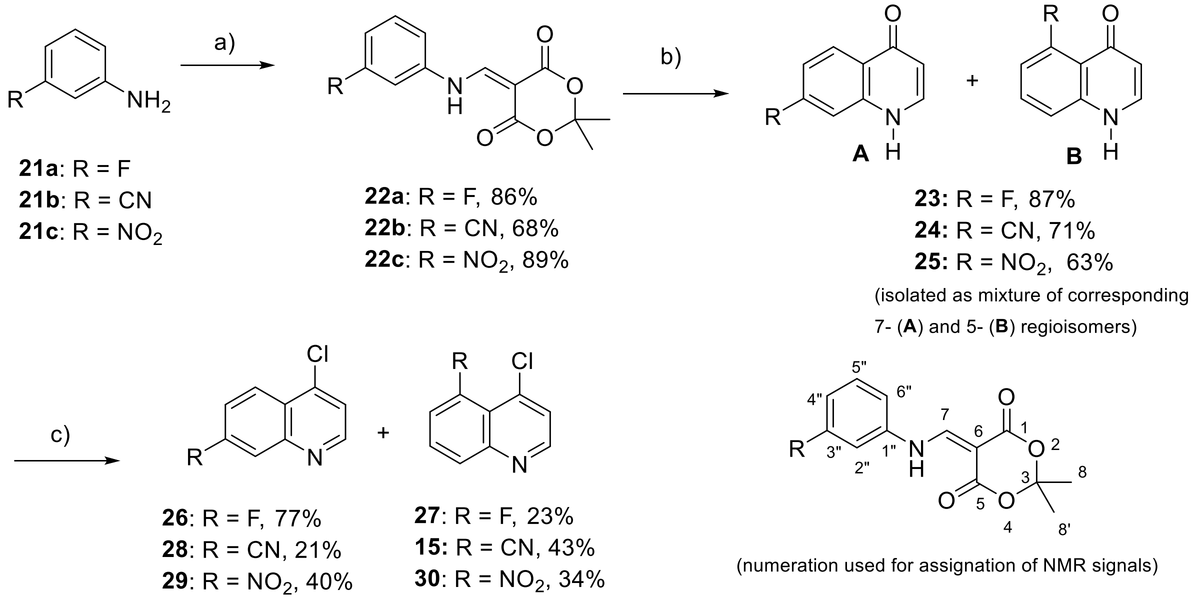

a) Meldrum's acid, orthoformate, EtOH dry, 80 °C (reflux), 2.5h; b) Ph_2O, 250 °C, 30 min.;
c) $POCl_3$, 145 °C, 45 min.;

Scheme S1. Synthesis of noncommercial 4-chloroquinolines **30**, **31**, **32**, **15**, **33** and **34**. Percentages denote the yield.

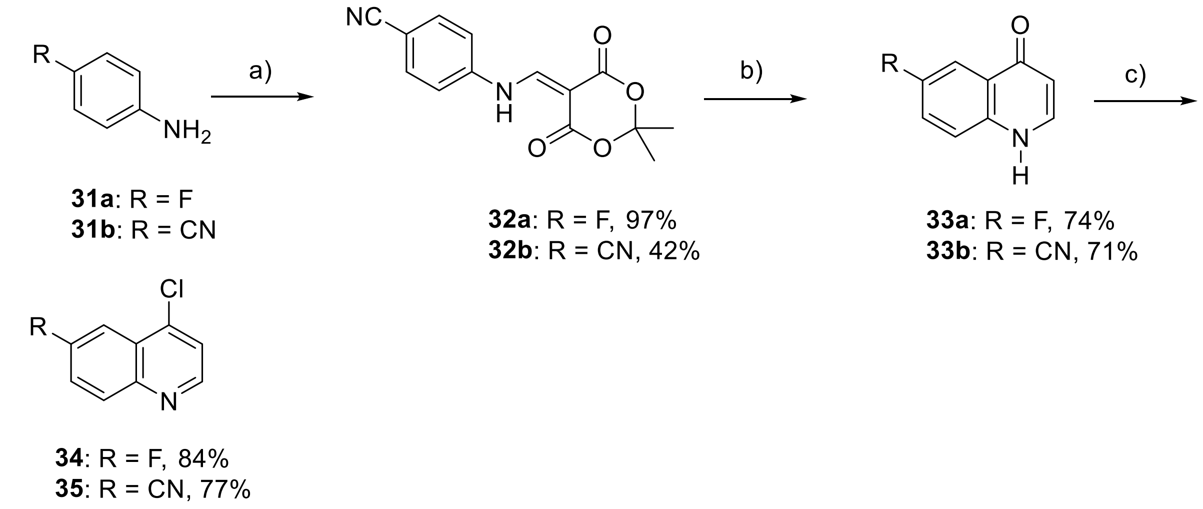

a) Meldrum's acid, orthoformate, EtOH dry, 80 °C (reflux), 2.5h; b) Ph_2O, 250 °C, 30 min.;
c) $POCl_3$, 145 °C, 45 min.;

Scheme S2. Synthesis of noncommercial 4-chloroquinolines **34** and **35**.

Starting non-commercial 4-chloro quinolines **26** **27**, **28**, **15**, **29** and **30** (Scheme S1) and **34** and **35** (Scheme S2), including corresponding intermediates, were synthetized according to the procedure described in the literature [1]. Obtained NMR spectra are in accordance with previously published data. [1]

Schemes S1 and S2: Briefly, the Meldrum's acid and triethyl-orthoformate were added into the intensively stirred solution of corresponding aniline (**21a-c** or **31a-b**; 1.5 mmol) in absolute ethanol (1.5 mL), and the reaction mixture was stirred at reflux for 2.5 hours. The enamine (**22a-c** or **32a-b**) separated as precipitate was filtered, rinsed with cold absolute EtOH, dried under reduced pressure and used without additional purification. In the next reaction, the corresponding enamine (1.13 mmol) was added slowly in small portions into the boiling Ph_2O (6 mL), and after 0.5 hours, the reaction mixture was cooled to room temperature, and the corresponding substituted 4-quinolones **23-25** or **33** separated as the precipitate were filtered, rinsed with hexane and dried under reduced pressure and used in the next reaction without additional purification. The products **23-25** were isolated as the mixtures of corresponding 7- and 5-substituted 4-quinolone regioisomers, and as such used in the next reaction without separation of isomers. In the next step, 4-substituted quinolones (**23-25** or **33**; 3.34 mmol) were dissolved in phosphoryl chloride (6.4 mL) and stirred at 145°C for 45 min, after which they were cooled down at room temperature. Phosphoryl chloride was removed under reduced pressure. Obtained solids were dissolved in water, and 0.1 M NaOH was added until the solution become basic (pH = 11). Emulsion was extracted with dichloromethane, and combined organic layers were washed with brine and dried over anhydrous Na_2SO_4. Solvent was removed under reduced pressure, and corresponding crude substituted 4-chloro quinoline derivatives were purified by column chromatography on silica gel using hexane/ethyl acetate gradient. Regioisomers **26** and **27**, **28** and **15**, and **29** and **30**, obtained from corresponding mixtures of corresponding 7- and 5-substituted 4-quinolone regioisomers **23-25**, were separated during purification and identified from their NMR spectra. Substituted 4-chloro quinolines **34** and **35** were obtained as the only products.

As a proof of identity of compounds and corresponding intermediates, 1H and 13C are given. The assignation of NMR signals was performed in accordance with numeration given in Scheme S1.

5-(((3-fluorophenyl)amino)methylene)-2,2-dimethyl-1,3-dioxane-4,6-dione (22a)

Compound **22a** was synthesized using 3-fluoroaniline **21a** (175.0 mg, 1.48 mmol, 1 eq), Meldrum's acid (311.0 mg, 2.16 mmol, 1.5 eq) and triethyl-orthoformate. Yield: 337.9 mg (86%). ^{1}H NMR (500 MHz, DMSO-d_6, δ): 10.98 (d, *J* = 14.4 Hz, 1H, NH), 8.34 (d, *J* = 14.5 Hz, 1H, H-7), 7.28 (dt, J_1 = 10.8, J_2 = 2.2 Hz, 1H, H-6´´), 7.25 – 7.17 (m, 1H, H-5´´), 7.13 (dd, J_1 = 8.1, J_2 = 1.7 1H, H-2´´), 6.84 (td, J_1 = 8.4, J_2 = 2.3 Hz, 1H, H-4´´), 1.42 (s, 6H, H-8 and H-8´). ^{13}C NMR (125 MHz, DMSO-d_6, δ): 164.55, 163.49, 163.26 (d, *J* = 194.0), 154.14, 141.0 (d, *J* = 10.9, C-1), 140.96, 132.07 (d, *J* = 9.1, C-5), 115.75, 113.56 (d, *J* = 21, C-4), 107.19 (d, *J* = 26.3, C-2), 105.05, 87.97, 27.14.

3-(((2,2-dimethyl-4,6-dioxo-1,3-dioxan-5-ylidene)methyl)amino)benzonitrile (22b)

Compound **22b** was synthesized using 3-cyanoaniline **21b** (175.0 mg, 1.58 mmol, 1 eq), Meldrum's acid (341.6 mg, 2.37 mmol, 1.5 eq) and triethyl-orthoformate (0.36 mL, 2.37 mmol, 1.5ekv). Yield: 234.4 mg (68%). ^{1}H NMR (500 MHz, DMSO-d_6, δ): 11.27 (d, *J* = 14.3 Hz, 1H, NH), 8.61 (d, *J* = 14.4 Hz, 1H, H-7), 8.15 – 8.10 (m, 1H, H-2´´), 7.91 – 7.85 (m, 1H, H-4´´),7.70 - 7.85 (m, 1H, H-6´´), 7.62 – 7.55 (m, 1H, H-5´´), 1.65 (s, 6H, H-8 and H-8´). ^{13}C NMR (125 MHz, DMSO-d_6, δ): 163.69, 162.58, 153.79, 139.61, 130.69, 129.52, 124.23, 122.76, 118.17, 112.29, 104.27, 87.78, 26.52.

2,2-dimethyl-5-(((3-nitrophenyl)amino)methylene)-1,3-dioxane-4,6-dione (22c)

Compound **22c** was synthesized using 3-nitroaniline **21c** (175.0 mg, 1.27 mmol, 1 eq), Meldrum's acid (250.0 mg, 1.74 mmol, 1.5 eq) and triethyl-orthoformate (0.28 mL, 1.65 mmol, 1.5 eq). Yield: 328.2 mg (89%). ^{1}H NMR (500 MHz, DMSO-d_6, δ): 11.25 (s, 1H, NH), 8.52 (s, 1H, H-7), 8.36 (s, 1H, H-2´´), 7.99 – 7.92 (m, *J*= 8.0 Hz, 1H, H-4´´), 7.92 – 7.85 (m, *J* = 8.1 Hz, 1H, H-6´´), 7.57 (t, *J* = 8.2 Hz, 1H, H-5´´), 1.55 (s, 6H, H-8 and H-8´). ^{13}C NMR (125 MHz, DMSO-d_6, δ): 163.64, 162.85, 154.09, 148.51, 140.19, 130.94, 125.70, 120.57, 114.68, 104.44, 88.05, 26.63.

Mixture of 7-fluoro-and 5-fluoro-4-quinolone (23)

The mixture **23** was synthesized using enamine **22a** (300.0 mg, 1.13 mmol) and 6.0 mL Ph_2O. The obtained product was a mixture of corresponding 7-fluoro and 5-fluoro regioisomers in the ratio 2:1. Yield: 160.5 mg (87%). Although corresponding isomers were isolated as mixture, in the ^{1}H NMR spectrum, signals of isomers are well-defined, which enables assignation and determination of isomer ratio. However, that was not possible for ^{13}C NMR, and corresponding signals were given collectively.

7-fluoro isomer: ^{1}H NMR (400 MHz, CD_3OD, δ): 8.29– 8.20 (m, 1H, H-2), 7.96 – 7.88 (m, 1H, H-8), 7.26 – 7.19 (m, 1H, H-5), 7.19 – 7.11 (m, 1H, H-6), 6.29 – 6.24 (m, 1H, H-3).
5-fluoro isomer: ^{1}H NMR (400 MHz, CD_3OD, δ): 7.88 – 7.82 (m, 1H, H-2), 7.63 – 7.54 (m, 1H, H-6), 7.33 – 7.28 (m, 1H, H-8), 7.02 – 6.94 (m, 1H, H-5), 6.24 – 6.19 (m, 1H, H-3).
Mixture: ^{13}C NMR (100 MHz, CD_3OD, δ): 180.17, 167.58, 165.08, 141.91, 140.85, 133.95, 133.84, 129.60, 129.49, 123.69, 115.35, 115.30, 114.38, 114.14, 111.70, 110.95, 110.73, 110.08, 104.54, 104.29.

Mixture of 7-cyano- and 5-cyano-4-quinolone (24)

The mixture **24** was synthesized using enamine **22b** (320.0 mg, 1.15 mmol) and 6.4 mL Ph_2O. The obtained product was a mixture of corresponding 7-cyano and 5-cyano regioisomers. Yield: 139.9 mg (71%). ^{1}H NMR (400 MHz, DMSO-d_6, δ): 7.23 – 7.16 (m, 1H), 7.06 – 6.92 (m, 2H), 6.86 – 6.69 (m, 4H), 6.67 – 6.58 (m, *J* = 8.3 Hz, 1H), 5.20 – 5.10 (m, 2H). ^{13}C NMR (100 MHz, DMSO-d_6, δ): 176.24, 175.18, 140.99, 140.76, 140.12, 139.66, 132.11, 131.60, 127.89, 126.70, 125.08, 124.99, 123.74, 119.03, 118.28, 113.92, 110.18, 109.99, 108.78.

Mixture of 7-nitro-and 5-nitro-4-quinolone (25)

The mixture **25** was synthesized using enamine **22c** (100.0 mg, 0.34 mmol) and 2.0 mL Ph_2O. The obtained product was a mixture of corresponding 7-nitro and 5-nitro regioisomers. Yield: 45.3 mg (70%).^{1}H NMR (400 MHz, DMSO-d_6, δ): 8.28 (s, 1H), 8.14 (d, *J* = 8.9 Hz, 1H), 7.95 (d, *J* = 7.5 Hz, 1H), 7.87 (dd, *J* = 15.1, 8.2 Hz, 2H), 7.61 (s, 1H), 7.39 – 7.31 (m, 1H), 7.24 (t, *J* = 7.8 Hz, 1H), 6.98 (t, *J* = 7.5 Hz, 1H), 6.84 (d, *J* = 8.0 Hz, 1H), 6.05 (d, *J* = 7.5 Hz, 1H), 5.96 (d, *J* = 7.5 Hz, 1H). ^{13}C NMR (100 MHz, DMSO-d_6, δ): 149.13, 148.22, 141.51, 141.01, 140.16, 139.82, 131.88, 130.19, 128.95, 127.51, 123.59, 121.38, 118.73, 117.44, 116.87, 115.85, 114.72, 110.39, 110.26.

4-chloro-7-fluoro- and 4-chloro-5-fluoroquinolines (26 and 27)

Compounds **26** and **27** were synthesized using **23** (545.1 mg, 3.34 mmol) and 6.4 mL phosphoryl chloride. Yield: **26** 440.6 mg (77%) and **27** 131.6 mg (23%).
26: ^{1}HNMR (400 MHz, $CDCl_3$, δ): 8.75 (d, *J*= 4.7, 1H, H-2), 8.22 (dd, J_1=9.3, J_2=5.9, 1H, H-5), 7.73 (dd, J_1=9.8, J_2=2.5, 1H, H-8), 7.49-7.33 (m, 2H, H-3 and H-6). ^{13}C NMR (100 MHz, $CDCl_3$) δ 163.46 (d, *J* = 252.0, C-7), 151.04 (C-2), 150.17 (d, *J* = 12.7), 142.69 (d, *J* = 1.4), 126.55 (d, *J* = 9.9, C-5), 123.56 (d, *J* = 1.2), 120.62 (d, *J* = 2.5, C-3), 118.08 (d, *J* = 25.3, C-6), 113.46 (d, *J* = 20.6, C-8).

27: ^{1}H NMR (400 MHz, $CDCl_3$, δ): 8.73 (d, *J*= 4.7, 1H, H-2), 7.92 (d, *J*=8.5, 1H, H-8), 7.65 (td, J_1=8,2, J_2=5.2, 1H, H-7), 7.46 (d, *J*=4.7, 1H, H-3), 7.31-7.19 (m, 1H, H-6). ^{13}C NMR (100 MHz, $CDCl_3$, δ):157.52 (d, *J* = 260.9, C-5), 150.71, 150.33 (d, *J* = 2.0, C-2), 139.40, 129.72 (d, *J* = 9.5, C-7), 126.19 (d, *J* = 4.6, C-8), 123.39 (d, *J* = 1.6, C-3), 112.76 (d, *J* = 21.9, C-)

4-chloroquinoline-7-carbonitrile and 4-chloroquinoline-5-carbonitrile (28 and 29)

Compounds **28** and **29** were synthesized using **24** (348.5 mg, 2.05 mmol) and 4.6 mL phosphorylchloride. Yield: **28** 81.1 mg (21%) and **29** 166.1 mg (43%).

28: ^{1}H NMR (400 MHz, $CDCl_3$, δ): 8.90 (d, *J* = 4.6 Hz, 1H, H-2), 8.50 (s, 1H, H-8), 8.35 (d, *J* = 8.7 Hz, 1H, H-5), 7.80 (dd, *J* = 8.7, 1.0 Hz, 1H, H-6), 7.64 (d, *J* = 4.7 Hz, 1H, H-3). ^{13}C NMR (100 MHz, $CDCl_3$, δ): 151.92, 148.18, 142.98, 135.83, 128.79, 128.43, 128.41, 126.06, 123.70, 118.07, 114.29.

29: ^{1}H NMR (400 MHz, $CDCl_3$, δ): 8.86 (d, *J* = 4.6 Hz, 1H, H-2), 8.38 (dd, *J* = 8.5, 1.3 Hz, 1H, H-8), 8.12 (dd, *J* = 7.3, 1.3 Hz, 1H, H-6), 7.82 (dd, J_1 = 8.5, J_2 = 7.3 Hz, 1H, H-5), 7.62 (d, *J* = 4.6 Hz, 1H, H-3). ^{13}C NMR (100 MHz, $CDCl_3$, δ): 151.21, 149.47, 141.47, 138.01, 136.11, 129.37, 125.62, 124.79, 118.57, 108.42.

4-chloro-7-nitroquinoline and 4-chloro-5-nitroquinoline (29 and 30)

Compounds **29** and **30** were synthesized using **25** (802.1 mg, 4.21 mmol) and 10.7 mL phosphoryl chloride. Yield: **29** 341.9 mg (40%) and **30** 290.6 mg (34%).

29: ^{1}H NMR (400 MHz, $CDCl_3$, δ): 9.02 (s, 1H, H-8), 8.95 (d, *J* = 4.6 Hz, 1H, H-2), 8.42 – 8.41 (m, 2H, H-5 and H-6), 7.68 (d, *J* = 4.6 Hz, 1H, H-3). ^{13}C NMR (100 MHz, $CDCl_3$, δ): 152.39, 148.87, 148.42, 143.02, 129.85, 126.45, 126.21, 124.00, 121.12.

30:^{1}H NMR (400 MHz, $CDCl_3$, δ): 8.88 (d, *J* = 4.7 Hz, 1H, H-2), 8.33 (dd, *J* = 7.6, 2.1 Hz, 1H, H-8), 7.83 – 7.76 (m, 2H, H-6 and H-7), 7.63 (d, *J* = 4.7 Hz, 1H, H-3).^{13}C NMR (100 MHz, $CDCl_3$, δ): 151.26, 149.75, 146.87, 139.19, 134.21, 128.83, 125.14, 123.45, 118.24.

The assignation of NMR signals was performed in accordance with numeration given in Scheme S1.

5-(((4-fluorophenyl)amino)methylene)-2,2-dimethyl-1,3-dioxane-4,6-dione (32a)

Compound **32a** was synthesized using 4-fluoroaniline **21** (175.0 mg, 1.48 mmol, 1 eq), Meldrum's acid (311.0 mg, 2.16 mmol, 1.5 eq) and triethyl-orthoformate (0.36 mL, 2.37, 1.5 eq). Yield: 381.1 mg (97%). ^{1}H NMR (500 MHz, DMSO-d_6, δ): 11.00 (d, *J* = 14.6 Hz, 1H, NH), 8.25 (d, *J* = 14.6 Hz, 1H, H-7), 7.38 – 7.30 (m, 2H, H-3´´ and H-5´´), 7.06 – 6.98 (m, 2H,

H-2´´ and H-6´´), 1.42 (s, 6H, H-8 and H-8´). ^{13}C NMR (125 MHz, DMSO-d_6, δ): 164.59, 163.65, 160.92 (d, *J* = 243.6 Hz), 154.44, 135.88, 122.03 (d, 8.4 Hz), 116.98 (d, *J* = 22.9 Hz), 104.92, 87.23, 27.11.

4-(((2,2-dimethyl-4,6-dioxo-1,3-dioxan-5-ylidene)methyl)amino)benzonitrile (32b)

Compound **32b** was synthesized using the 4-cyanoaniline **22** (175 mg, 1.48 mmol, 1 eq), Meldrum's acid (311.0 mg, 2.16 mmol, 1.5 eq) and triethyl-orthoformate (0.36 mL, 2.37, 1.5 eq). Yield: 165.0 mg (42%). ^{1}H NMR (500 MHz, DMSO-d_6, δ): 8.50 (s, 1H, H-7), 7.78 – 7.64 (m, 2H, H-3´´ and H-5´´), 7.64 – 7.07 (m, 2H, H-2´´ and H-6´´), 1.54 (s, 6H, H-8 and H-8´). ^{13}C NMR (125 MHz, DMSO-d_6, δ): 153.24, 142.60, 133.91, 119.76, 118.69, 108.12, 104.60, 88.57, 26.66.

6-fluoro-4-quinolone (33a)

Compound **33a** was synthesized using Ph_2O (6.0 mL) and enamine **32a** (300.0 mg, 1.13 mmol). Yield: 179.0 mg (97%). ^{1}H NMR (400 MHz, CD_3OD, δ): 7.95 (d, *J* = 7.3 Hz, 1H, H-2), 7.84 (dd, *J* = 9.3, 2.9 Hz, 1H, H-5), 7.61 (dd, *J* = 9.1, 4.5 Hz, 1H, H-8), 7.50 (td, *J* = 8.6, 2.9 Hz, 1H, H-7), 6.29 (d, *J* = 7.2 Hz, 1H,H-3). ^{13}C NMR (100 MHz, CD_3OD, δ): 179.90, 160.77 (d, *J* = 244.3 Hz), 142.54, 141.48, 138.20, 127.94 (d, *J* = 7.2 Hz), 122.54 (d, *J* = 26.0 Hz), 122.16 (d, *J* = 8.2 Hz), 110.13 (d, *J* = 22.9 Hz), 109.14.

6-cyano-4-quinolone (33b)

Compound **33b** was synthesized using Ph_2O (6.4 mL) and enamine **32b** (320.0 mg, 1.15 mmol. Yield: 82.6 mg (41%). ^{1}H NMR (400 MHz, DMSO-d_6, δ): 11.97 (s, NH), 8.21 (d, *J* = 2.0 Hz, 1H, H-5), 7.84 – 7.67 (m, 2H, H-2 and H-7), 7.46 (d, *J* = 8.7 Hz, 1H, H-8), 5.96 (d, *J* = 7.5 Hz, 1H, H-3). ^{13}C NMR (100 MHz, DMSO-d_6, δ) δ 176.22, 142.52, 141.81, 140.85, 133.75, 131.08, 125.36, 120.18, 118.91, 110.54, 105.64.

4-chloro-6-fluoroquinoline (34)

Compound **34** was synthesized using **33a** (440.9 mg, 2.70 mmol) and Ph_2O (5.3 mL). Yield: 361.9 mg (84%). ^{1}H NMR (400 MHz, $CDCl_3$, δ): 8.75 (d, *J* = 4.7 Hz, 1H, H-2), 8.12 (dd, *J* = 9.2, 5.3 Hz, 1H, H-8), 7.84 (dd, *J* = 9.4, 2.8 Hz, 1H, H-5), 7.58 – 7.48 (m, 2H, H-3 and H-7). ^{13}C NMR (100 MHz, $CDCl_3$, δ): 161.15 (d, *J* = 249.9, C-6), 159.92, 149.09 (d, *J* = 2.8, C-2), 146.18, 141.81 (d, *J* = 5.7), 132.54 (d, *J* = 9.2, C-8), 127.47 (d, *J* = 10.3), 121.76 (C-3), 120.70 (d, *J* = 25.8, C-7), 107.87 (d, *J* = 24.4, C-5).

4-chloroquinoline-6-carbonitrile (35)

Compound **35** was synthesized using **33b** (120.8 mg, 0.71 mmol) and Ph_2O (1.6 mL). Yield: 103.1 mg (77%). ^{1}H NMR (400 MHz, $CDCl_3$, δ): 8.92 (d, *J* = 4.7 Hz, 1H, H-2), 8.65 (d, *J* = 1.5 Hz, 1H, H-5), 8.22 (d, *J* = 8.7 Hz, 1H, H-8), 7.92 (dd, *J* = 8.7, 1.7 Hz, 1H, H-7), 7.62 (d, *J* = 4.7 Hz, 1H, H-3). ^{13}C NMR (100 MHz, $CDCl_3$, δ): 152.91, 150.11, 143.26, 131.65, 131.20, 130.80, 126.37, 122.92, 118.31, 111.70.

*1.2.2. Synthesis of AMQ and amodiaquine derivatives **1 – 14***

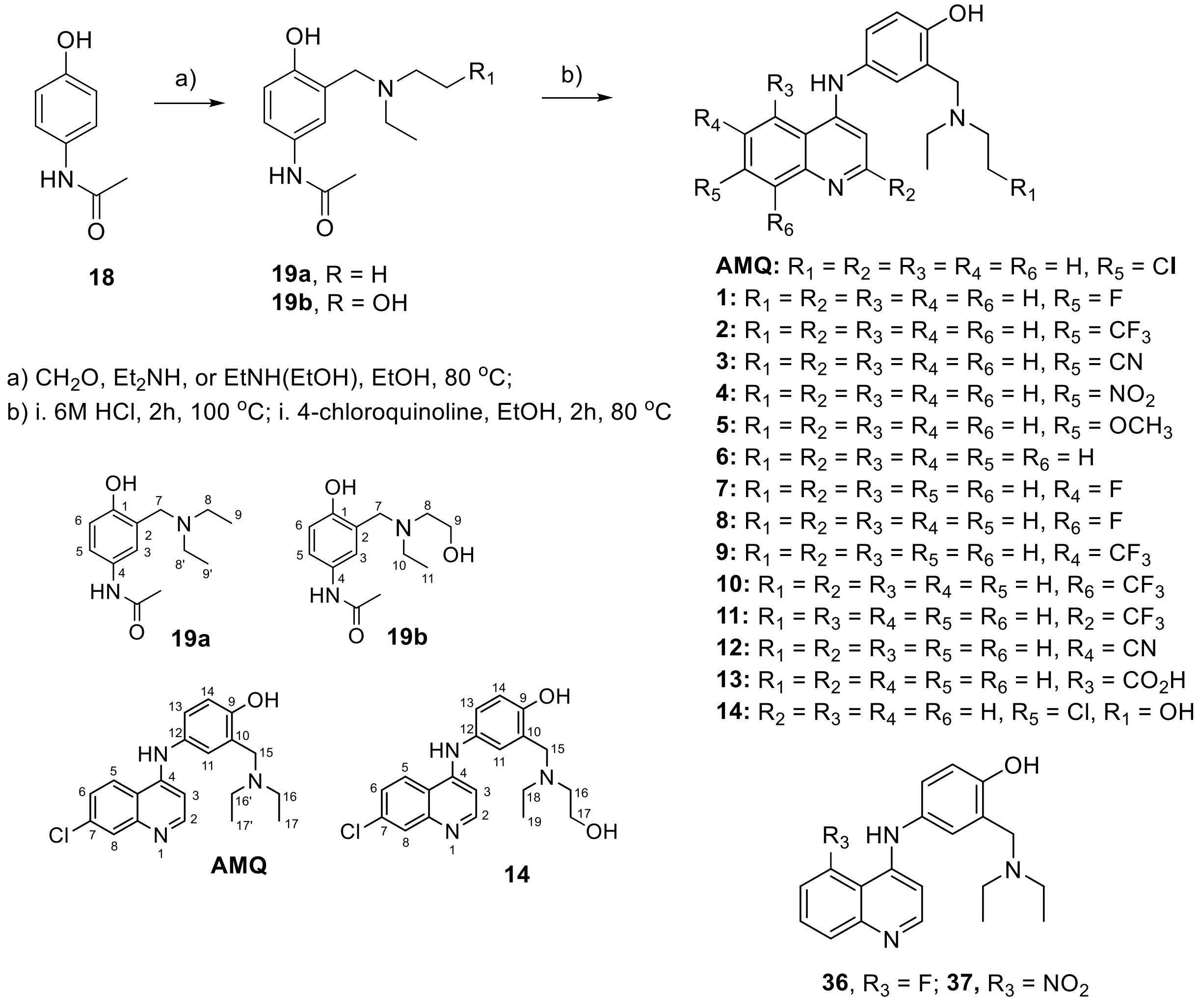

Scheme S3. Synthesis of amodiaquine derivatives **1 – 14**.

*General procedure for synthesis of compounds **19a** and **19b**:* A mixture of compound **1''**, formaldehyde and corresponding amine in EtOH was heated to reflux. After 48 hours, EtOH

was removed under reduced pressure. The residue was dissolved in dichloromethane and washed with 0.1 M HCl (2 x 120 mL). Solid NaOH (0.96 g) was added to the aqueous layer and extracted with dichloromethane (6 x 30 mL). Combined organic extracts were dried under Na_2SO_4 anhydrous. Solvent was removed under reduced pressure and product was isolated by dry-flash column chromatography on silica gel. Our attempt to synthesize amodiaquine derivatives **36** and **37** (Scheme S3), using 5-fluoro-4chloroquinoline (**27**) or 5-nitro-4-chloroquinoline (**30**), failed. During the procedure, a complex mixture was obtained from which, to the best of our knowledge, the isolation of desired compounds was not possible.

The assignation of NMR signals was performed in accordance with numeration given in Scheme S3.

4-amino-2-((diethylamino)methyl)phenol (19a)

Compound **19a** was synthesized using compound **18** (4.53 g, 30 mmol), formaldehyde (4.5 mL, 60 mmol, 2 eq) and diethylamine (6.18 mL, 60 mmol, 2 eq) in EtOH (90 mL). Yield: 2.84 g (40%). Yellow powder, Mp = 124 ºC. IR (ATR): 3278 m, 3208 w, 3160 w, 3108 m, 2972 m, 2929 w, 2853 m, 1652 s, 1623 m, 1564 s, 1492 s, 1469 m, 1333 w, 1260 s, 1195 w, 1113 w, 1013 w, 898 w, 832 m, 768 m, 654 w. ^{1}H NMR (400 MHz, $CDCl_3$, δ): 7.64 (s, 1H, H-O), 7.30 (s, 1H, H-3), 7.05 (d, *J* = 8.5 Hz, 1H, H-5), 6.70 (d, *J* = 8.6 Hz, 1H, H-6), 3.69 (s, 2H, H-7), 2.58 (q, *J* = 7.1 Hz, 4H, H-8 and H-8´), 2.09 (s, 3H, CH_3CO), 1.07 (t, *J* = 7.1 Hz, 6H, H-9 and H-9´). ^{13}C NMR (100 MHz, $CDCl_3$, δ): 168.35, 155.07, 129.48, 122.16, 121.14, 120.79, 116.00, 77.35, 77.03, 76.71, 56.80, 46.27, 24.19, 11.13.

***N*-(3-((ethyl(2-hydroxyethyl)amino)methyl)-4-hydroxyphenyl)acetamide (19b)**

Compound **19b** was synthesized using compound **18** (1.51 g, 10 mmol), formaldehyde (1.5 mL, 20 mmol, 2 eq) and 2-(ethylamino)ethan-1-ol (1.96 mL, 20 mmol, 2 eq) in EtOH (30 mL). Yield: 376.6 mg (15%). Dark yellow wax-like solid, softens at 84−100 ºC. IR (ATR): 3278 m, 3208 w, 3160 w, 3108 m, 2972 m, 2929 w, 2853 m, 1652 s, 1623 m, 1564 s, 1492 s, 1469 m, 1333 w, 1260 s, 1195 w, 1113 w, 1013 w, 898 w, 832 m, 768 m, 654 w ^{1}H NMR (400 MHz, CD_3OD, δ): 7.22 (d, *J* = 2.6 Hz, 1H, H-3), 7.17 (dd, *J* = 8.6, 2.6 Hz, 1H, H-5), 6.65 (d, *J* = 8.6 Hz, 1H, H-6), 3.76 (s, 2H, H-7), 3.67 (t, *J* = 5.9 Hz, 2H, H-9), 2.73 – 2.54 (m, 4H, H-8 and H-10), 2.05 (s, 3H, CH_3CO), 1.07 (t, *J* = 7.2 Hz, 3H, H-11). ^{13}C NMR (100 MHz, CD_3OD, δ):

171.33, 155.77, 131.41, 124.04, 122.79, 122.27, 116.73, 60.13, 58.08, 55.89, 49.65, 49.44, 49.22, 49.00, 48.80, 48.58, 48.44, 48.37, 23.52, 11.31.

*General procedure for synthesis of AMQ and compounds **1 − 14***: Compound **19a** or **19b** was heated to reflux for 2 hours in 6M HCI (0.50 mL, 3 mmol), solvent was evaporated to dryness under reduced pressure. The remaining oil was dissolved in absolute EtOH and corresponding 4-chloroquinoline was added. The reaction mixture was heated to reflux for 2 hours, solvent was removed under reduced pressure, water and 30% ammonia were added and solid was extracted with dichloromethane. Combined organic extracts were washed with brine and dried under Na_2SO_4 anhydrous. Solvent was removed under reduced pressure and product was isolated by dry-flash column chromatography on silica-gel.

4-((7-chloroquinolin-4-yl)amino)-2-((diethylamino)methyl)phenol (AMQ)

AMQ was obtained using **19a** (112.6 mg, 0.476 mmol), 4,7-dichloroquinoline (94.3 mg, 0.476 mmol) and absolute EtOH (2 mL). Yield: 109.5 mg (71.8 %). Yellow powder, Mp = 192-194 ºC. IR (ATR): 3243 m, 3102 m, 3057 m, 3026 m, 2978 s, 2936 m, 2850 m, 1613 m, 1568 s, 1542 s, 1494 s, 1420 m, 1391 m, 1215 m, 1167 m, 1106 m, 1000 w, 955 w, 880 m, 855 m, 818 m, 765 m, 676 w, 610 w. ^{1}H NMR (400 MHz, $CDCl_3$, δ): 8.48 (d, *J* = 5.4 Hz, 1H, H-2), 8.00 (s, 1H, H-8), 7.83 (d, *J* = 9.0 Hz, 1H, H-5), 7.40 (dd, *J* = 9.0, 2.2 Hz, 1H, H-6), 7.08 (dd, *J* = 8.5, 2.6 Hz, 1H, H-13), 6.93 (d, *J* = 2.6 Hz, 1H, H-11), 6.86 (d, *J* = 8.5 Hz, 1H, H-14), 6.62 (d, *J* = 5.4 Hz, 1H, H-3), 3.78 (s, 2H, H-15), 2.65 (q, *J* = 7.2 Hz, 4H, H-16 and H-16´), 1.13 (t, *J* = 7.2 Hz, 6H, H-17 and H-17´). ^{13}C NMR (100 MHz, $CDCl_3$, δ): 165.04, 164.51, 156.87, 152.06, 149.48, 135.31, 129.91, 128.92, 125.87, 125.65, 125.40, 123.46, 121.20, 117.52, 117.29, 101.45, 77.48, 77.16, 77.16, 76.84, 56.95, 46.56, 11.37. (+)ESI-HRMS (m/z): calc. for $[C_{20}H_{22}ClN_3O + H]^+$ 356.1530, found 356.1528. HPLC purity: Method A: RT 7.486, area 100.00%; method B: RT 8.414, area 99.36%.

2-((diethylamino)methyl)-4-((7-fluoroquinolin-4-yl)amino)phenol (1)

Compound **1** was obtained using **19a** (116.7 mg, 0.494 mmol), 4-chloro-7-fluoroquinoline (**26**, 89.7 mg, 0.494 mmol) and absolute EtOH (2 mL). Yield: 89.3 mg (53%). Pale yellow powder, Mp = 182-188 ºC. IR (ATR): 3189 m, 3064 m, 2977 m, 2939 m, 1630 m, 1577 s, 1544 s, 1493 s, 1426 m, 1374 m, 1262 s, 1227 m, 1159 m, 1110 w, 965 m, 870 m, 816 m, 782 w, 764 m, 648 w. ^{1}H NMR (400 MHz, $CDCl_3$, δ): 8.46 (d, *J* = 5.4 Hz, 1H, H-2), 7.91 (dd, *J* = 9.2, 5.8 Hz, 1H,

H-5), 7.61 (dd, *J* = 10.3, 2.5 Hz, 1H, H-8), 7.24 – 7.19 (m, 1H, H-6), 7.07 (dd, *J* = 8.5, 2.4 Hz, 1H, H-13), 6.91 (d, *J* = 2.1 Hz, 1H, H-11), 6.85 (d, *J* = 8.5 Hz, 1H, H-14), 6.71 (bs, 1H, H-O), 6.59 (d, *J* = 5.4 Hz, 1H, H-3), 3.76 (s, 2H, H-15), 2.64 (q, *J* = 7.2 Hz, 4H, H-16 and H-16´), 1.12 (t, *J* = 7.2 Hz, 6H, H-17 and H-17´). ^{13}C NMR (100 MHz, $CDCl_3$, δ): 163.10 (d, *J* = 249.0 Hz), 156.74, 152.15, 150.42 (d, *J* = 12.3 Hz), 149.59, 130.11, 125.52 (d, *J* = 24.7 Hz), 123.41, 121.95 (d, *J* = 10.1 Hz), 117.23, 116.09, 115.01 (d, *J* = 25.1 Hz), 113.62 (d, *J* = 19.8 Hz), 100.92, 56.94, 46.54, 11.35. (+)ESI-HRMS (m/z): calc. for $[C_{20}H_{22}FN_3O + H]^+$ 340.1825, found 340.1831. HPLC purity: Method A: RT 7.357, area 100.00%; method B: RT 8.288, area 99.18%.

2-((diethylamino)methyl)-4-((7-(trifluoromethyl)quinolin-4-yl)amino)phenol (2)

Compound **2** was obtained using **19a** (120.4 mg, 0.509 mmol), 4-chloro-7-(trifluoromethyl)quinoline (117.9 mg, 0.509 mmol) and absolute EtOH (2 mL). Yield: 123.4 mg (62.3%). Pale yellow powder, Mp = 192-194 ºC. IR (ATR): 3188 m, 3064 m, 2980 m, 2945 m, 1583 s, 1569 s, 1489 s, 1379 s, 1328 s, 1292 m, 1257 s, 1196 m, 1119 s, 1076 w, 996 w, 916 w, 830 m, 820 m, 798 w, 7390 w, 683 w. ^{1}H NMR (400 MHz, $CDCl_3$, δ): 8.54 (d, *J* = 5.2 Hz, 1H, H-2), 8.28 (s, 1H, H-8), 8.00 (d, *J* = 8.8 Hz, 1H, H-5), 7.62 (d, *J* = 8.7 Hz, 1H, H-6), 7.08 (dd, *J* = 8.5, 2.3 Hz, 1H, H-13), 6.92 (d, *J* = 2.0 Hz, 1H, H-11), 6.85 (d, *J* = 8.5 Hz, 1H, H-14), 6.72 – 6.65 (m, 2H, H-3 and H-O), 3.77 (s, 2H, H-15), 2.64 (q, *J* = 7.2 Hz, 4H, H-16 and H-16´), 1.12 (t, *J* = 7.2 Hz, 6H, H-17 and H-17´). ^{13}C NMR (100 MHz, $CDCl_3$, δ): 165.01, 157.00, 152.34, 149.35, 148.25, 131.68, 131.35, 131.04, 130.71, 129.80, 128.00, 127.95, 127.91, 127.87, 125.75, 125.48, 123.54, 122.75, 121.02, 120.78, 120.66, 120.62, 120.59, 120.56, 117.36, 102.47, 77.48, 77.16, 76.84, 56.95, 46.57, 11.36. (+)ESI-HRMS (m/z): calc. for $[C_{21}H_{22}F_3N_3O + H]^+$ 390.1793, found 390.1786. HPLC purity: Method A: RT 7.629, area 99.43%; method B: RT 8.583, area 98.65%.

4-((3-((diethylamino)methyl)-4-hydroxyphenyl)amino)quinoline-7-carbonitrile (3)

Compound **3** was obtained using **19a** (114.4 mg, 0.484 mmol), 4-chloroquinoline-7-carbonitrile (**28**, 91.3 mg, 0.484 mmol) and absolute EtOH (2 mL). Yield: 144.9 mg (86,4%). Yellow powder, Mp = 168-170 ºC. IR (ATR): 3130 m, 3087 m, 2977 m, 2931 m, 2227 m, 1582 s, 1566 s, 1531 m, 1494 s, 1454 w, 1379 s, 1328 w, 1261 m, 1251 m, 1225 m, 1166 m, 1105 m, 980 w, 915 w, 889 w, 822 w, 759 w, 656 w. ^{1}H NMR (400 MHz, $CDCl_3$, δ): 8.55 (d, *J* = 5.3 Hz, 1H, H-2), 8.33 (s, 1H, H-8), 8.01 (d, *J* = 8.7 Hz, 1H, H-5), 7.59 (d, *J* = 8.4 Hz, 1H, H-6), 7.08 (dd, *J* = 8.5, 2.3 Hz, 1H, H-13), 6.93 (d, *J* = 2.1 Hz, 1H, H-11), 6.86 (d, *J* = 8.5 Hz,

1H, H-14), 6.79 (s, 1H, H-O), 6.72 (d, *J* = 5.3 Hz, 1H, H-3), 3.78 (s, 2H, H-15), 2.65 (q, *J* = 7.1 Hz, 4H, H-16 and H-16´), 1.13 (t, *J* = 7.2 Hz, 6H, H-17 and H-17´). ^{13}C NMR (100 MHz, $CDCl_3$, δ): 157.12, 152.69, 149.44, 148.01, 135.81, 129.49, 125.76, 125.64, 125.49, 123.55, 121.53, 121.42, 118.67, 117.40, 112.85, 102.96, 77.48, 77.16, 76.84, 56.88, 46.54, 29.81, 11.33. (+)ESI-HRMS (m/z): calc. for $[C_{21}H_{22}N_4O + H]^+$ 347.1872, found 347.1871. HPLC purity: Method A: RT 7.311, area 98.57%; method B: RT 8.232, area 99.17%.

2-((diethylamino)methyl)-4-((7-nitroquinolin-4-yl)amino)phenol (4)

Compound **4** was obtained using **19a** (112.4 mg, 0.476 mmol), 4-chloro-7-nitroquinoline (**29**, 99.3 mg, 0.476 mmol) and absolute EtOH (2 mL). Yield: 148.0 mg (84.8 %). Yellow powder, Mp = 188-192 ºC. IR (ATR): 3183 w, 3096 w, 2976 m, 2940 m, 1582 s, 1535 s, 1488 s, 1422 m, 1373 s, 1350 m, 1262 s, 1196 w, 1161 w, 1107 w, 1078 w, 997 w, 908 w, 836 w, 823 m, 764 m, 741 m, 656 w. ^{1}H NMR (400 MHz, $CDCl_3$, δ): 8.84 (d, *J* = 1.9 Hz, 1H, H-8), 8.59 (d, *J* = 5.3 Hz, 1H, H-2), 8.19 (dd, *J* = 9.2, 2.0 Hz, 1H, H-6), 8.06 (d, *J* = 9.2 Hz, 1H, H-5), 7.09 (dd, *J* = 8.4, 2.1 Hz, 1H, H-13), 6.95 (d, *J* = 1.6 Hz, 1H, H-11), 6.89 – 6.81 (m, 2H, H-14 and H-O), 6.74 (d, *J* = 5.3 Hz, 1H, H-3), 3.78 (s, 2H, H-15), 2.65 (q, *J* = 7.1 Hz, 4H, H-16 and H-16´), 1.13 (t, *J* = 7.1 Hz, 6H, H-17 and H-17´). ^{13}C NMR (100 MHz, $CDCl_3$, δ): 157.19, 153.21, 149.57, 148.33, 148.09, 129.43, 126.12, 125.82, 125.55, 123.61, 122.54, 121.76, 118.17, 117.41, 103.23, 77.16, 56.90, 46.55, 11.34. (+)ESI-HRMS (m/z): calc. for $[C_{20}H_{22}N_4O_3 + H]^+$ 367.1770, found 367.1784. HPLC purity: Method A: RT 7.383, area 98.78%; method B: RT 8.428, area 99.66%.

2-((diethylamino)methyl)-4-((7-methoxyquinolin-4-yl)amino)phenol (5)

Compound **5** was obtained using **19a** (100 mg, 0.423 mmol) and 4-chloro-7-metoxyquinoline (81.9 mg, 0.423 mmol) and absolute EtOH (2 mL). Yield: 98.2 mg (66 %). Brownish powder, Mp = 178-184ºC. IR (ATR): 2972m, 2934m, 2831m, 1621m, 1577s, 1549m, 1496s, 1476s, 1436s, 1383m, 1361m, 1327w, 1308m, 1292m, 1257s, 1229s, 1198m, 1173m, 1111w, 1068w, 1025m, 1001w, 953w, 871w, 851m, 811m, 769m, 660w, 558w, 452w, cm^{-1}. ^{1}H NMR (400 MHz, $CDCl_3$, δ): 8.41 (*d*, 1H, *J* = 5.3, H-2), 7.80 (*d*, 1H, *J* = 9.2, H-5), 7.35 (*bs*, 1H, H-8), 7.15 – 7.04 (*m*, 2H, H-6 and H-13), 6.92 (*d*, 1H, *J* = 1.9, H-11), 6.85 (*d*, 1H, *J* = 8.5, H-14), 6.63 (*bs*, 1H, H-O), 6.55 (*d*, 1H, *J* = 5.4, H-3), 3.91 (*s*, 3H, CH_3-O), 3.77 (*s*, 2H, H-15), 2.65 (*q*, 4H, *J* = 7.2, H-16 and H-16`), 1.13 (*t*, 6H, *J* = 7.2, H-17 and H-17`). ^{13}C NMR (100 MHz, $CDCl_3$, δ): 160.61, 156.48, 151.20, 150.74, 149.43, 130.47, 125.45, 125.24, 123.31, 120.97, 117.66, 117.13, 113.67, 108.15, 100.14, 56.95, 55.57, 46.52, 11.36. (+)ESI-HRMS (*m/z*): calc. for

$[C_{21}H_{25}N_3O_2+ H]^+$ 352.2025, found 352.2011. HPLC purity: Method A: RT 7.513, area 100.00%; method B: RT 8.830, area 97.93%.

2-((diethylamino)methyl)-4-(quinolin-4-ylamino)phenol (6)

Compound **6** was obtained using **19a** (123.6 mg, 0.523 mmol) and 4-chloroquinoline (86.3 mg, 0.527 mmol) and absolute EtOH (2 mL). Yield: 49.3 mg (29 %). Brownish powder, Mp = 152-156 ºC. IR (ATR): 3181m, 3057m, 2978m, 2924m, 2846m, 1619w, 1578s, 1540m, 1490s, 1442m, 1389s, 1333m, 1294m, 1254s, 1218m, 1191m, 1166m, 1106w, 1049w, 1026w, 994w, 962w, 943w, 911w, 892m, 877w, 816m, 767s, 652m, 631w, 556m, 499w, 479w, 445w, 430w, cm^{-1}. ^1H NMR (400 MHz, $CDCl_3$, δ): 8.43 (*d*, 1H, *J* = 5.2, H-2), 8.01 (*d*, 1H, *J* = 8.5, H-5), 7.92 (*d*, 1H, *J* = 8.3, H-8), 7.65 (*t*, 1H, *J* = 7.6, H-6), 7.46 (*t*, 1H, *J* = 7.5, H-7), 7.09 (*d*, 1H, *J* = 8.5, H-13), 6.93 (*s*, 1H, H-11), 6.85 (*d*, 1H, *J* = 8.5, H-14), 6.76 (*bs*, 1H, H-O), 6.65 (*d*, 1H, *J* = 5.3, H-3), 3.77 (*s*, 2H, H-17), 2.65 (*q*, 4H, *J* = 7.1, H-18 and H-18`), 1.13 (*t*, 6H, *J* = 7.1, H-19 and H-19`). ^{13}C NMR (100 MHz, $CDCl_3$, δ): 156.61, 150.78, 149.48, 148.74, 130.42, 129.91, 129.42, 125.54, 125.30, 125.14, 123.39, 119.65, 119.12, 117.20, 101.12, 56.98, 46.58, 11.36. (+)ESI-HRMS (*m/z*): calc. for $[C_{20}H_{23}N_3O+ H]^+$ 322.1919, found 322.1923. HPLC purity: Method A: RT 7.296, area 100.00%; method B: RT 8.309, area 99.29%.

2-((diethylamino)methyl)-4-((6-fluoroquinolin-4-yl)amino)phenol (7)

Compound **7** was obtained using **19a** (121.8 mg, 0.515 mmol), 4-chloro-6-fluoroquinoline (**34**, 93.5 mg, 0.515 mmol) and absolute EtOH (2 mL). Yield: 130.3 mg (74.5 %). Yellow powder, Mp = 162-164 ºC. IR (ATR): 3238 m, 3040 m, 2969 m, 2935 m, 2874 m, 2829m, 1626 w, 1603 w, 1574 m, 1544 m, 1515 m, 1496 s, 1449 m, 1378 m, 1328 w, 1291 w, 1260 m, 1244 m, 1195 m, 1168 w, 1096 w, 1052 w, 996 w, 921 m, 876 m, 839 w, 829 m, 766 w, 668 w. ^1H NMR (400 MHz, $CDCl_3$, δ): 8.45 (d, *J* = 5.3 Hz, 1H, H-2), 7.98 (dd, *J* = 9.3, 5.6 Hz, 1H, H-8), 7.56 (d, *J* = 9.8 Hz, 1H, H-5), 7.40 (td, *J* = 9.3, 2.4 Hz, 1H, H-7), 7.06 (dd, *J* = 8.5, 2.6 Hz, 1H, H-15), 6.90 (d, *J* = 1.1 Hz, 1H, H-11), 6.84 (d, *J* = 8.5 Hz, 1H, H-14), 6.71 – 6.61 (m, 2H, H-3 and H-O), 3.75 (s, 2H, H-15), 2.63 (q, *J* = 7.1 Hz, 4H, H-16 and H-16´), 1.12 (t, *J* = 7.1 Hz, 6H, H-17 and H-17´). ^{13}C NMR (100 MHz, $CDCl_3$, δ): 159.89 (d, *J* = 246.0 Hz), 156.55, 150.31 (d, *J* = 2.2 Hz), 149.09 (d, *J* = 4.9 Hz), 146.04, 132.45 (d, *J* = 8.9 Hz), 130.31, 125.33 (d, *J* = 23.1 Hz), 123.37, 119.68 (d, *J* = 8.5 Hz), 119.13 (d, *J* = 25.0 Hz), 117.18, 103.99 (d, *J* = 23.0 Hz), 101.53, 56.91, 46.50, 11.32. (+)ESI-HRMS (m/z): calc. for $[C_{20}H_{22}FN_3O + H]^+$ 340.1825, found 340.1823. HPLC purity: Method A: RT 7.332, area 99.55%; method B: RT 8.233, area 99.57%.

2-((diethylamino)methyl)-4-((8-fluoroquinolin-4-yl)amino)phenol (8)

Compound **8** was obtained using **19a** (117.4 mg, 0.497 mmol), 4-chloro-8-fluoroquinoline (90.3 mg, 0.497 mmol) and absolute EtOH (2 mL). Yield: 119.4 mg (70.1%). Brownish powder, Mp = 166-168 ºC. IR (ATR): 3195 m, 3104 w, 3080 w, 2980 m, 2938 m, 2836 m, 1596 w, 1579 m, 1539 s, 1493 s, 1422 m, 1258 s, 1223 w, 1193 w, 1060 w, 964 m, 919 m, 810 m, 753 m, 741 w, 609 w. ^{1}H NMR (400 MHz, $CDCl_3$, δ): 8.51 (d, *J* = 5.2 Hz, 1H, H-2), 7.67 (d, *J* = 8.1 Hz, 1H, H-7), 7.42 – 7.29 (m, 2H, H-5 and H-6), 7.07 (d, *J* = 8.5 Hz, 1H, H-13), 6.92 (s, 1H, H-11), 6.85 (d, *J* = 8.5 Hz, 1H, H-14), 6.92 (bs, 1H, H-O), 6.85 (d, *J* = 8.5 Hz, 1H, H-3),3.76 (s, 2H, H-15), 2.64 (q, *J* = 7.1 Hz, 4H, H-16 and H-16´), 1.12 (t, *J* = 7.1 Hz, 6H, H-17 and H-17´). ^{13}C NMR (100 MHz, $CDCl_3$, δ): 163.68, 158.57 (d, *J* = 254.8 Hz), 156.64, 150.94, 149.04 (d, *J* = 3.4 Hz), 139.35 (d, *J* = 11.9 Hz), 129.80, 125.41 (d, *J* = 24.9 Hz), 124.32 (d, *J* = 8.4 Hz), 123.26, 120.72 (d, *J* = 3.0 Hz), 117.08, 115.13 (d, *J* = 4.6 Hz), 113.35 (d, *J* = 19.3 Hz), 101.6, 56.76, 46.37, 11.18. (+)ESI-HRMS (m/z): calc. for $[C_{20}H_{22}FN_3O + H]^+$ 340.1825, found 340.1828. HPLC purity: Method A: RT 7.279, area 100.00%; method B: RT 8.234, area 99.27%.

2-((diethylamino)methyl)-4-((6-(trifluoromethyl)quinolin-4-yl)amino)phenol (9)

Compound **9** was obtained using **19a** (112.4 mg, 0.476 mmol), 4-chloro-6-(trifluoromethyl)quinoline (110.2 mg, 0.476 mmol) and absolute EtOH (2 mL). Yield: 135.6 mg (73.1 %). Yellow powder, Mp = 192-196 ºC. IR (ATR): 3231 m, 3049 m, 2973 m, 2935 m, 2879 w, 2833 w, 1636 w, 1576 s, 1541 s, 1494 s, 1452 s, 1379 s, 1317 s, 1252 s, 1195 w, 1114 s, 1071 m, 996 w, 942 w, 892 w, 835 m,794 w, 628 w. ^{1}H NMR (400 MHz, $CDCl_3$, δ): 8.58 (d, *J* = 5.4 Hz, 1H, H-2), 8.23 (s, 1H, H-5), 8.12 (d, *J* = 8.9 Hz, 1H, H-8), 7.82 (dd, *J* = 8.8, 1.9 Hz, 1H, H-7), 7.11 (dd, *J* = 8.5, 2.6 Hz, 1H, H-13), 6.94 (d, *J* = 2.6 Hz, 1H, H-11), 6.87 (d, *J* = 8.5 Hz, 1H, H-14), 6.76 (bs, 1H, H-O), 6.70 (d, *J* = 5.4 Hz, 1H, H-3), 3.79 (s, 2H, H-15), 2.66 (q, *J* = 7.2 Hz, 4H, H-16 and H-16´), 1.14 (t, *J* = 7.2 Hz, 6H, H-17 and H-17´). ^{13}C NMR (100 MHz, $CDCl_3$, δ): 165.14, 157.13, 152.98, 150.24, 131.20, 131.14, 131.12, 131.08, 129.59, 126.89, 126.60, 125.82, 125.54, 125.30, 125.24, 125.21, 125.16, 123.56, 118.21, 118.13, 118.09, 118.04, 118.00, 117.39, 102.14, 77.48, 77.16, 76.84, 56.94, 46.57, 11.36. (+)ESI-HRMS (m/z): calc. for $[C_{21}H_{22}F_3N_3O + H]^+$ 390.1793, found 390.1798. HPLC purity: Method A: RT 7.646, area 99.54%; method B: RT 8.629, area 98.46%.

2-((diethylamino)methyl)-4-((8-(trifluoromethyl)quinolin-4-yl)amino)phenol (10)

Compound **10** was obtained using **19a** (112.7 mg, 0.477 mmol), 4-chloro-8-(trifluoromethyl)quinoline (110.5 mg, 0.477 mmol) and absolute EtOH (2 mL). Yield: 30.7 mg (70.4%). Yellow powder, Mp = 162-166 ºC. IR (ATR): 3235 m, 2972 m, 2934 m, 2823 m, 1584 s, 1540 s, 1423 w, 1386 m, 1313 s, 1253 s, 1150 m 1128 s, 1058 m, 987 w, 895 w, 816 w, 774 m, 715 w, 603 w. ^{1}H NMR (400 MHz, $CDCl_3$, δ): 8.61 (dd, *J* = 5.4, 1.5 Hz, 1H, H-2), 8.10 (d, *J* = 8.5 Hz, 1H, H-7), 8.01 (d, *J* = 7.3 Hz, 1H, H-6), 7.47 (t, *J* = 8.0 Hz, 1H, H-5), 7.07 (dd, *J* = 8.5, 2.6 Hz, 1H, H-13), 6.91 (d, *J* = 2.6 Hz, 1H, H-11), 6.85 (d, *J* = 8.5 Hz, 1H, H-14), 6.69 (t, *J* = 6.7 Hz, 1H, H-3), 6.65 (s, 1H, H-O), 3.77 (s, 2H, H-15), 2.64 (q, *J* = 7.1 Hz, 4H, H-16 and H-16´), 1.13 (t, *J* = 7.1 Hz, 6H, H-17 and H-17´). ^{13}C NMR (100 MHz, $CDCl_3$, δ): 156.87, 156.83, 151.76, 149.50, 145.80, 129.90, 129.88, 127.98, 127.93, 127.87, 127.81, 125.78, 125.64, 125.39, 124.24, 124.19, 123.47, 123.36, 119.78, 117.29, 101.94, 77.48, 77.16, 76.84, 56.91, 46.53, 11.33. (+)ESI-HRMS (m/z): calc. for $[C_{21}H_{22}F_3N_3O + H]^+$ 390.1793, found 390.1797. HPLC purity: Method A: RT 7.503, area 100.00%; method B: RT 8.418, area 98.96%.

2-((diethylamino)methyl)-4-((2-(trifluoromethyl)quinolin-4-yl)amino)phenol (11)

Compound **11** was obtained using **19a** (115.1 mg, 0.487 mmol), 4-chloro-2-(trifluoromethyl)quinoline (112,8 mg, 0,487 mmol) and absolute EtOH (2 mL). Yield: 79,3 mg (41,8%). Yellow powder, Mp = 112-114 ºC. IR (ATR): 3331 w, 2975 m, 2936 m, 2877 m, 2851 m, 1589 s, 1574 s, 1499 s, 1460 m, 1404 m, 1367 w, 1340 w, 1276 s, 1259 s, 1183 s, 1141 s, 1074 w, 998 w, 948 w, 844 w, 765 w, 554 w. ^{1}H NMR (400 MHz, $CDCl_3$, δ): 8.13 (d, *J* = 8.5 Hz, 1H, H-5), 7.91 (d, *J* = 8.4 Hz, 1H, H-8), 7.75 (t, *J* = 7.7 Hz, 1H, H-6), 7.58 (t, *J* = 7.6 Hz, 1H, H-7), 7.11 (dd, *J* = 8.5, 2.6 Hz, 1H, H-15), 6.95 (d, *J* = 2.6 Hz, 1H, H-11), 6.93 – 6.88 (m, 2H, H-14 and H-O), 6.75 (d, *J* = 4.6 Hz, 1H, H-3), 3.80 (s, 2H, H-15), 2.67 (q, *J* = 7.2 Hz, 4H, H-16 and H-16´), 1.14 (t, *J* = 7.2 Hz, 6H, H-17, H-17'). ^{13}C NMR (100 MHz, $CDCl_3$, δ): 157.32, 151.03, 149.11, 148.78, 148.19, 130.98, 130.45, 129.30, 126.84, 125.92, 125.66, 123.71, 119.34, 119.01, 117.54, 96.65, 96.62, 96.60, 96.57, 77.48, 77.16, 76.84, 56.85, 46.55, 11.29. (+)ESI-HRMS (m/z): calc. for $[C_{21}H_{22}F_3N_3O + H]^+$ 390.1793, found 390.1797. HPLC purity: Method A: RT 8.338, area 98.85%; method B: RT 9.922, area 98.47%.

4-((3-((diethylamino)methyl)-4-hydroxyphenyl)amino)quinoline-6-carbonitrile (12)

Compound **12** was obtained using **19a** (118.4 mg, 0.501 mmol), 4-chloroquinoline-6-carbonitrile (**35**, 94.5 mg, 0.501 mmol) and absolute EtOH (2 mL). Yield: 72.1 mg (41.5%). Yellow powder, Mp = 184-186 ºC. IR (ATR): 3233 m, 3040 m, 2975 m, 2933m, 2874 m, 2226 m, 1604 m, 1568 s, 1542 m, 1496 s, 1450 m, 1496 s, 1450 m, 1378 m, 1365 m, 1321 m, 1263 m, 1249 s, 1219 m, 1168 w, 1119 w, 1054 w, 878 m, 837 m, 796 w, 767 w, 671 w. ^{1}H NMR (400 MHz, $CDCl_3$, δ): 8.56 (d, *J* = 5.3 Hz, 1H, H-2), 8.41 (s, 1H, H-5), 8.04 (d, *J* = 8.7 Hz, 1H, H-8), 7.78 (d, *J* = 8.8 Hz, 1H, H-7), 7.10 (dd, *J* = 8.5, 2.2 Hz, 1H, H-13), 6.98 – 6.83 (m, 3H, H-11, H-14 and H-O), 6.71 (d, *J* = 5.4 Hz, 1H, H-3), 3.79 (s, 2H, H-15), 2.66 (q, *J* = 7.1 Hz, 4H, H-16 and H-16´), 1.14 (t, *J* = 7.2 Hz, 6H, H-17 and H-17´). ^{13}C NMR (100 MHz, $CDCl_3$, δ): 157.18, 153.75, 150.42, 149.82, 131.43, 130.03, 129.39, 127.08, 125.74, 125.45, 123.58, 119.35, 118.90, 117.40, 107.94, 102.44, 77.48, 77.16, 76.84, 56.95, 46.57, 11.36. (+)ESI-HRMS (m/z): calc. for $[C_{21}H_{22}N_4O + H]^+$ 347.1872, found 347.1862. HPLC purity: Method A: RT 7.308, area 100.00%; method B: RT 8.178, area 97.39%.

4-((3-((diethylamino)methyl)-4-hydroxyphenyl)amino)quinoline-5-carboxylic acid (13)

Compound **13** was obtained according to general procedure using **19a** (112.4 mg, 0.476 mmol), 4-chloroquinoline-5-carbonitrile (**15**, 89.8 mg, 0.476 mmol) and absolute EtOH (2 mL). Yield: 109.4 (62.9 %). Brown solid, Mp = 112-114ºC. IR (ATR): 2972 w, 2945 w, 2826 w, 1728 s, 1638 m, 1620 m, 1603 w, 1498 s, 1415 w, 1389 w, 1314w, 1268 m, 1072 w, 1021 m, 912 w, 835 w, 773 w, 742 w, 607 w. ^{1}H NMR (400 MHz, $CDCl_3$, δ): 8.85 (d, *J* = 4.8 Hz, 1H, H-2), 8.24 (d, *J* = 8.4 Hz, 1H, H-8), 8.11 (d, *J* = 7.0 Hz, 1H, H-6), 7.95 – 7.86 (m, 1H, H-7), 7.29 – 7.21 (m, 1H, H-13), 7.12 (d, *J* = 2.1 Hz, 1H, H-11), 6.94 (d, *J* = 8.5 Hz, 1H, H-14), 6.85 (d, *J* = 4.8 Hz, 1H, H-3), 3.84 (s, 2H, H-15), 2.67 (q, *J* = 7.1 Hz, 4H, H-16 and H-16´), 1.14 (t, *J* = 7.2 Hz, 6H, H-17 and H-17´). ^{13}C NMR (100 MHz, $CDCl_3$, δ): 167.08, 166.89, 158.49, 154.28, 147.33, 143.77, 132.85, 131.95, 126.15, 126.10, 126.08, 125.40, 125.06, 123.22, 121.85, 117.14, 101.69, 77.48, 77.16, 76.84, 56.94, 46.54, 11.34. (+)ESI-HRMS (m/z): calc. for $[C_{21}H_{23}N_3O_3 + H]^+$ 366.1818, found 366.1815. HPLC purity: Method A: RT 7.581, area 98.76%; method B: RT 9.156, area 99.33%.

4-((7-chloroquinolin-4-yl)amino)-2-((ethyl(2-hydroxyethyl)amino)methyl)phenol (14)

Compound **14** was obtained using **19b** (100 mg, 0.396 mmol) and 4,7-dichloroquinoline (78.5 mg, 0.396 mmol) and absolute EtOH (2 mL). Yield: 96.4 mg (65 %). Pale-yellow powder, Mp = 214-218 ºC. IR (ATR): 3274m, 3064m, 2974m, 2896m, 2809m, 1608m, 1572s, 1535m, 1494s, 1472m, 1446m, 1415m, 1373s, 1329m, 1304w, 1259s, 1215w, 1198w, 1182w, 1162w,

1139w, 1108w, 1078m, 1061w, 1046m, 1001w, 911m, 869m, 857m, 817w, 802m, 783m, 762m, 666w, 647w, 629w, 607w, 587w, 571w, 539m, 498w, 451w, 427w, cm^{-1}. ^{1}H NMR (400 MHz, DMSO-d_6, δ): 8.89 (*s*, 1H, H-O), 8.40 (*d*, 1H, *J* = 9.0, H-5), 8.35 (*d*, 1H, *J* = 4.3, H-2), 7.84 (*s*, 1H, H-8), 7.50 (*d*, 1H, *J* = 9.0, H-6), 7.08 – 7.06 (*m*, 2H, H-11 and H-13), 6.78 (*d*, 1H, *J* = 9.1, H-14), 6.56 (*d*, 1H, *J* = 5.3, H-3), 3.76 (*s*, 2H, H-15), 3.57 – 3.53 (*m*, 2H, H-19), 2.63 – 2.52 (*m*, 4H, H-16 and H-18), 1.01 (*t*, 3H, *J* = 7.0, H-19). ^{13}C NMR (100 MHz, DMSO-d_6, δ): 155.02, 151.91, 149.61, 149.47, 133.81, 130.41, 127.57, 125.57, 124.76, 124.63, 124.29, 117.76, 116.19, 100.54, 58.44, 55.53, 54.69, 46.90, 10.89. (+)ESI-HRMS (*m/z*): calc. for $[C_{20}H_{22}ClN_3O_2+ H]^+$ 372.1479, found 372.1486. HPLC purity: Method A: RT 7.434, area 97.43%; method B: RT 8.650, area 97.65%.

1.2.3. ^{1}H and ^{13}C NMR spectra

^{1}H NMR spectrum of 5-(((3-fluorophenyl)amino)methylene)-2,2-dimethyl-1,3-dioxane-4,6-dione (22a)

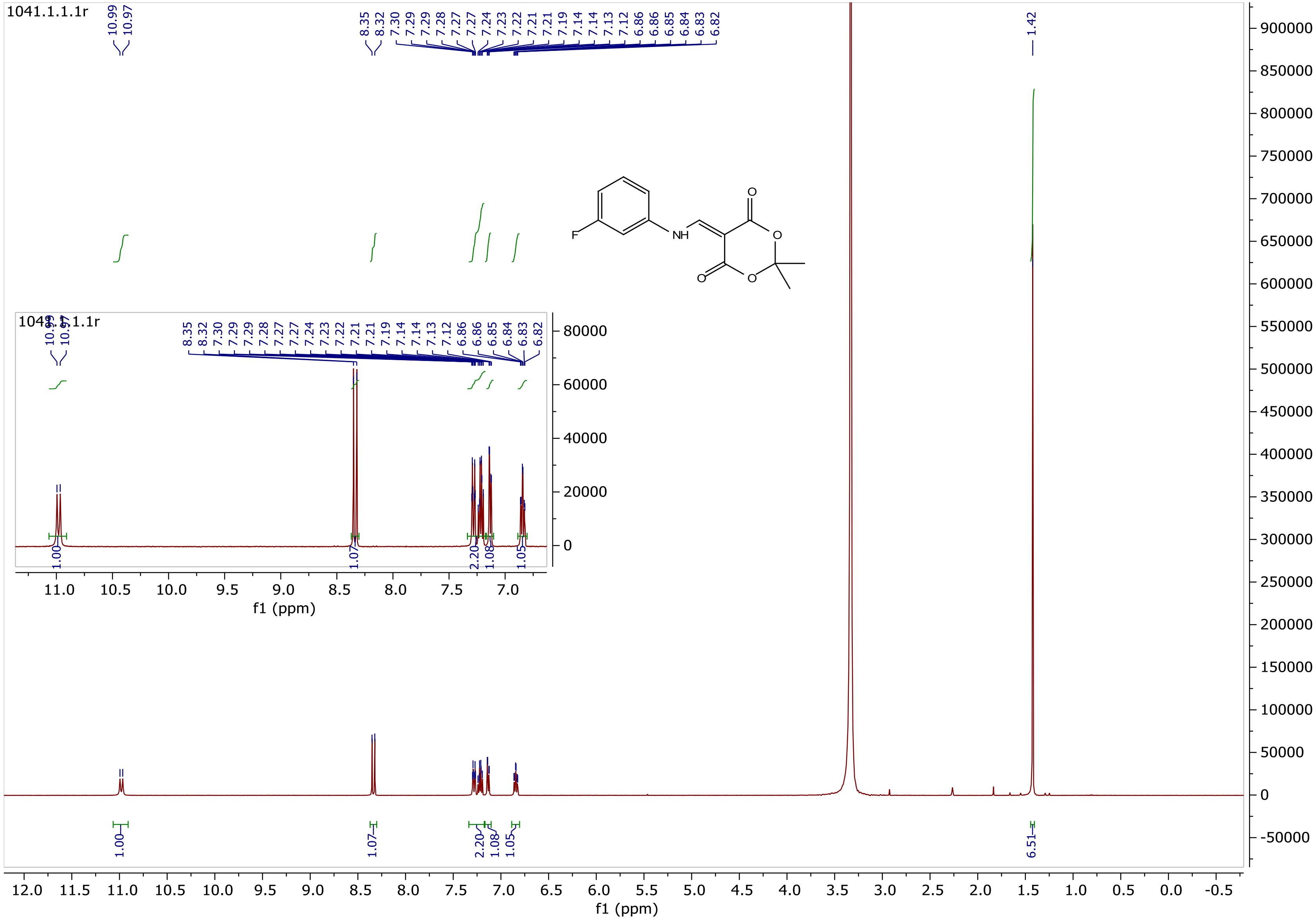

^{13}C NMR spectrum of 5-(((3-fluorophenyl)amino)methylene)-2,2-dimethyl-1,3-dioxane-4,6-dione (22a)

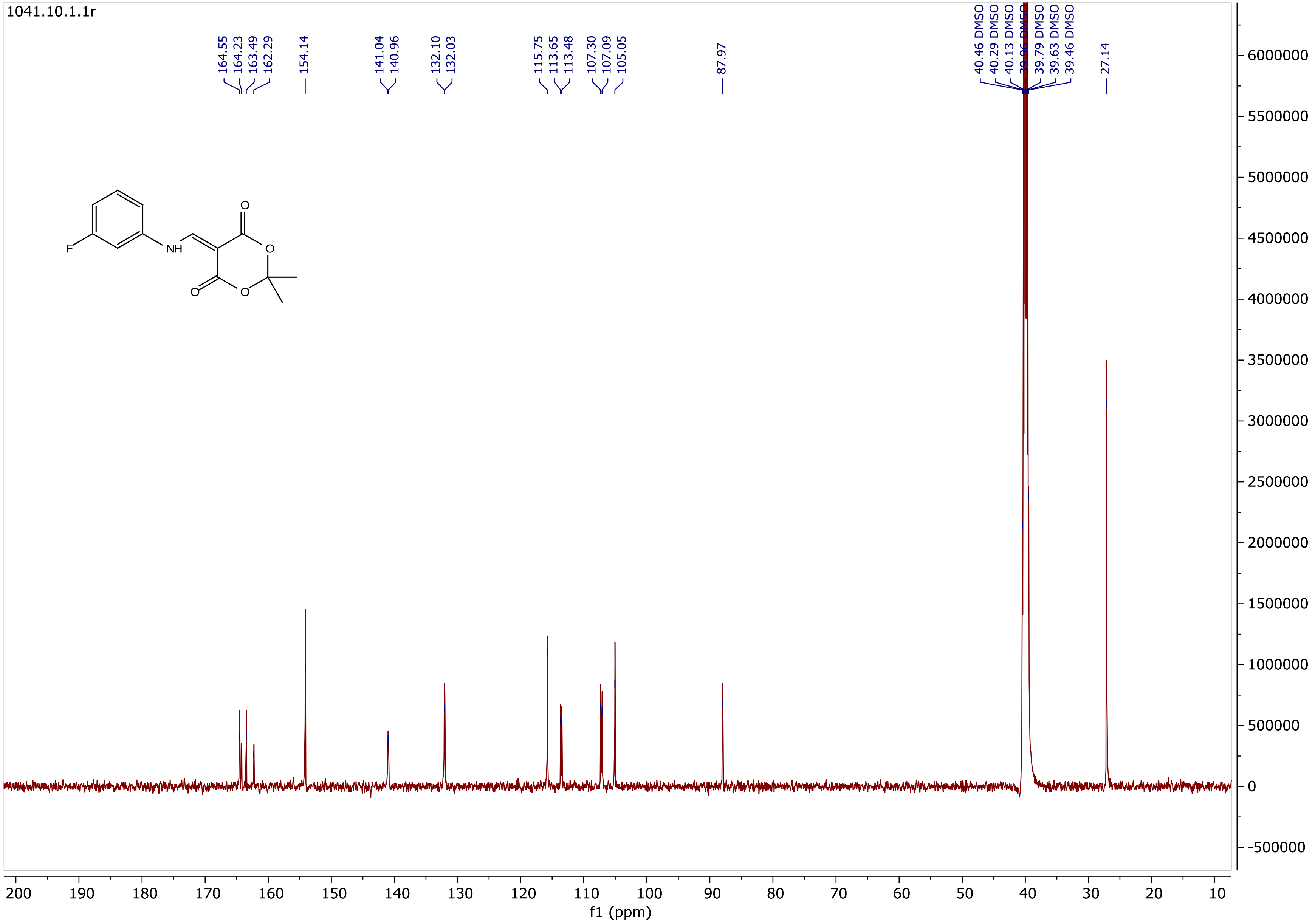

^{1}H NMR spectrum of 3-(((2,2-dimethyl-4,6-dioxo-1,3-dioxan-5-ylidene)methyl)amino)benzonitrile (22b)

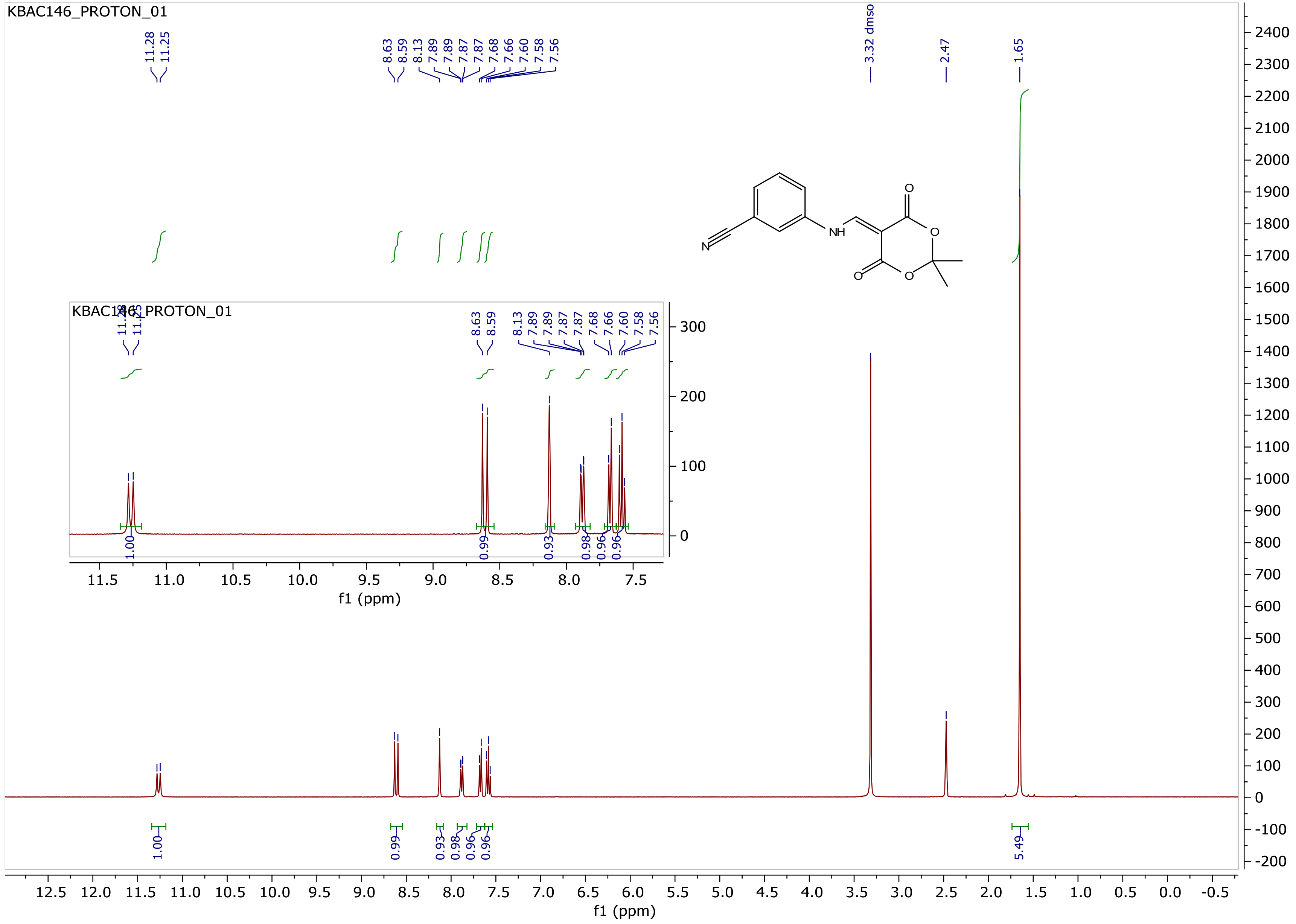

^{13}C NMR spectrum of 3-(((2,2-dimethyl-4,6-dioxo-1,3-dioxan-5-ylidene)methyl)amino)benzonitrile (22b)

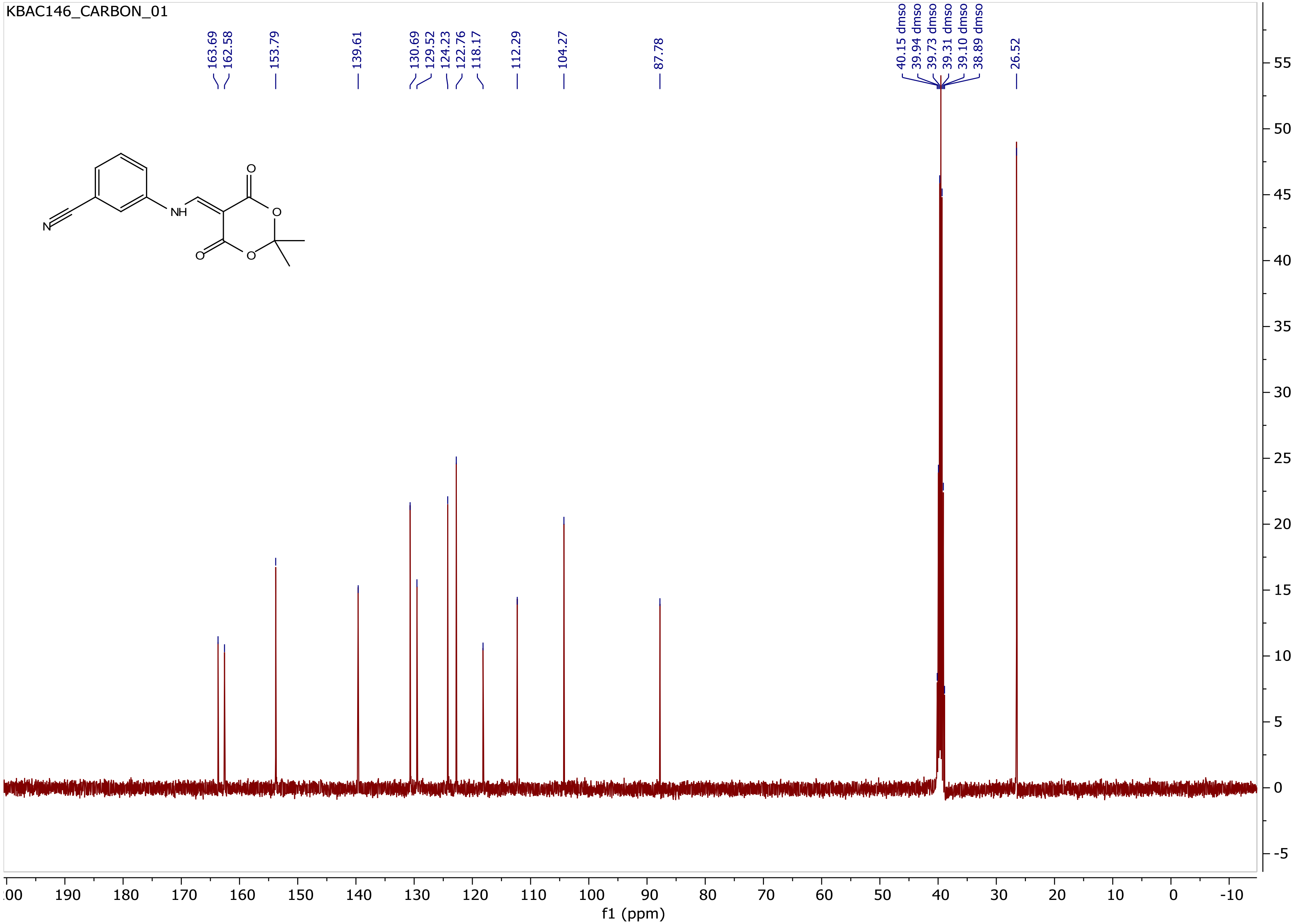

^{1}H NMR spectrum of 2,2-dimethyl-5-(((3-nitrophenyl)amino)methylene)-1,3-dioxane-4,6-dione (22c)

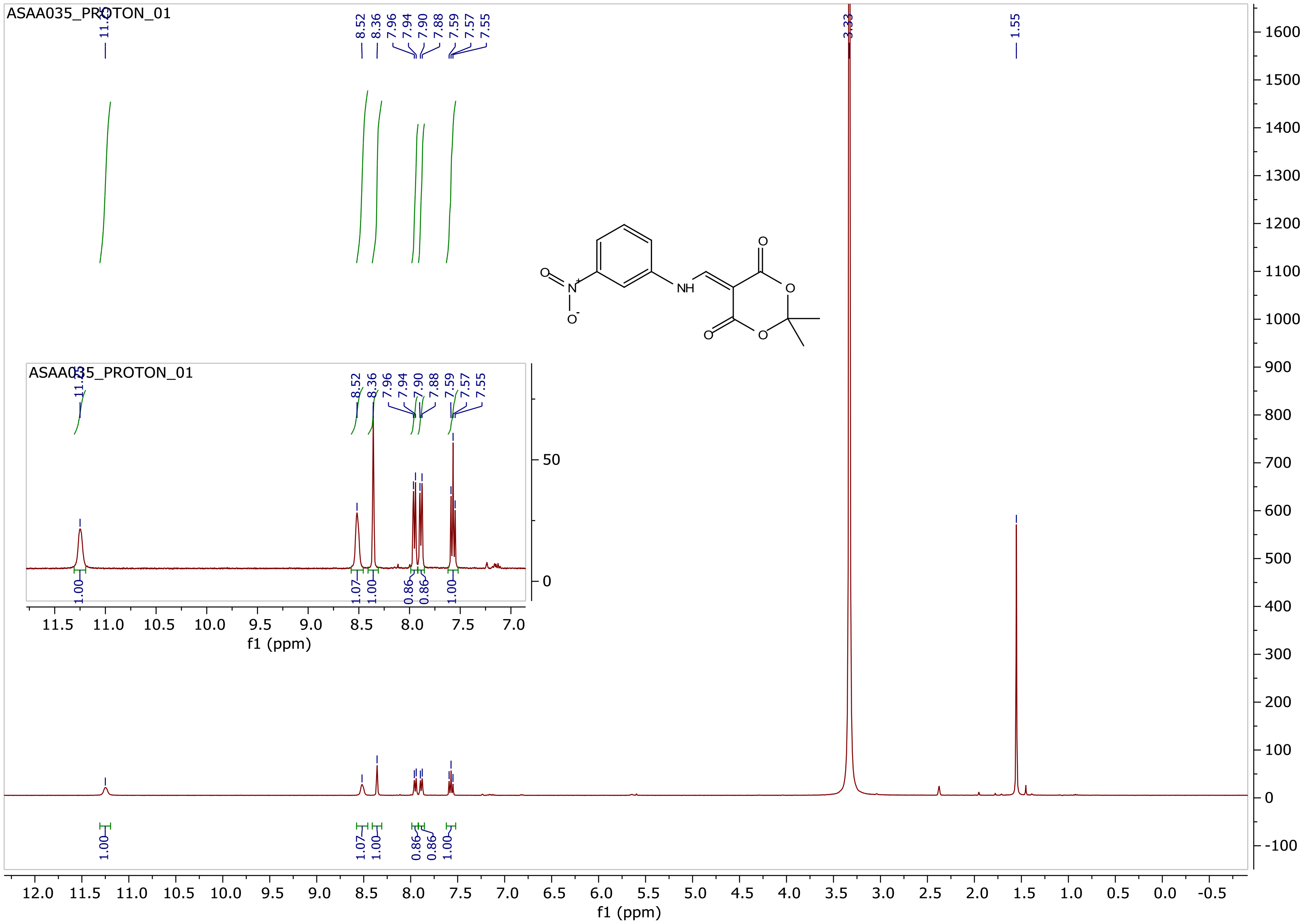

^{13}C NMR spectrum of 2,2-dimethyl-5-(((3-nitrophenyl)amino)methylene)-1,3-dioxane-4,6-dione (22c)

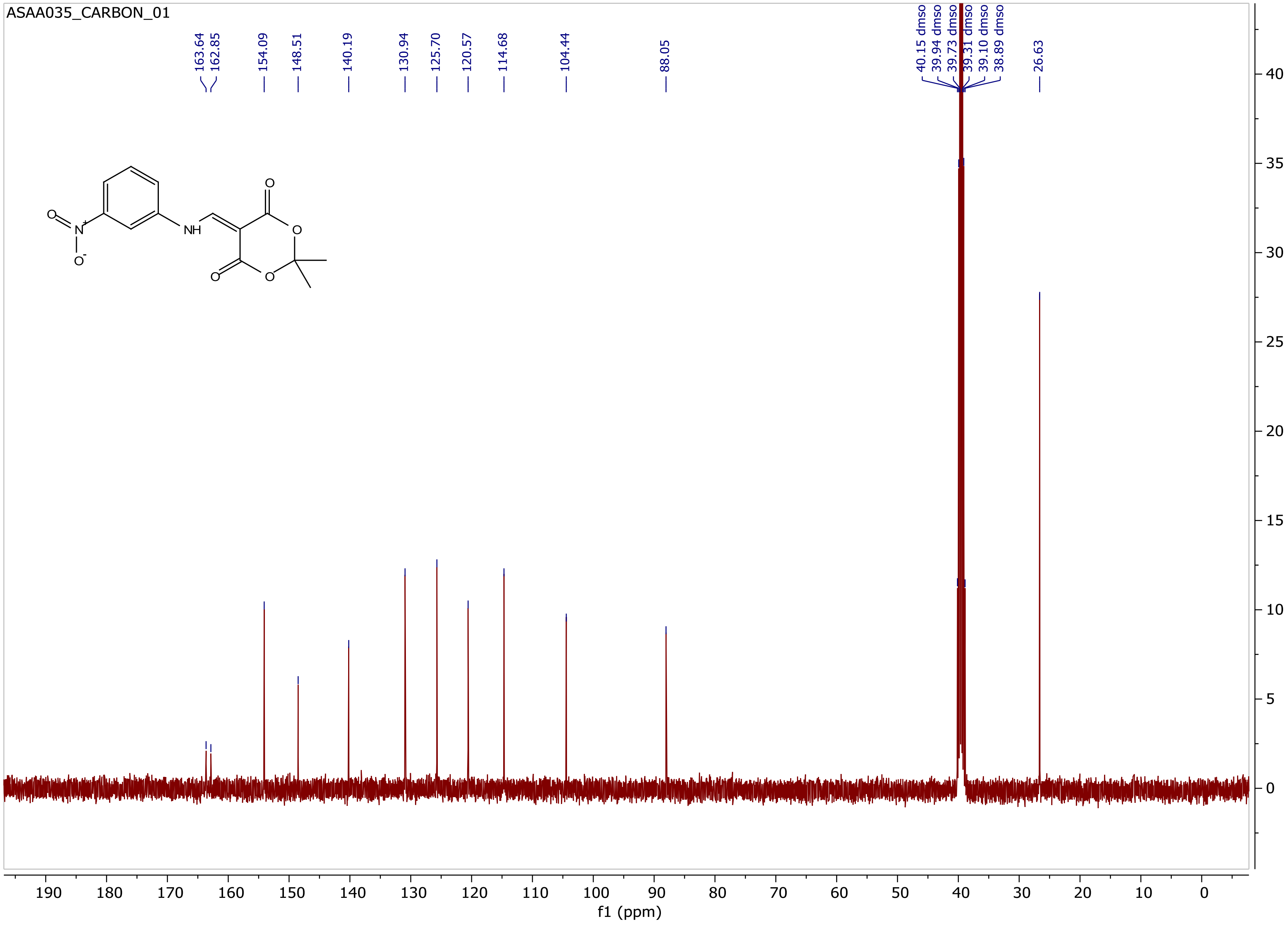

^{1}H NMR spectrum of 5-(((4-fluorophenyl)amino)methylene)-2,2-dimethyl-1,3-dioxane-4,6-dione (32a)

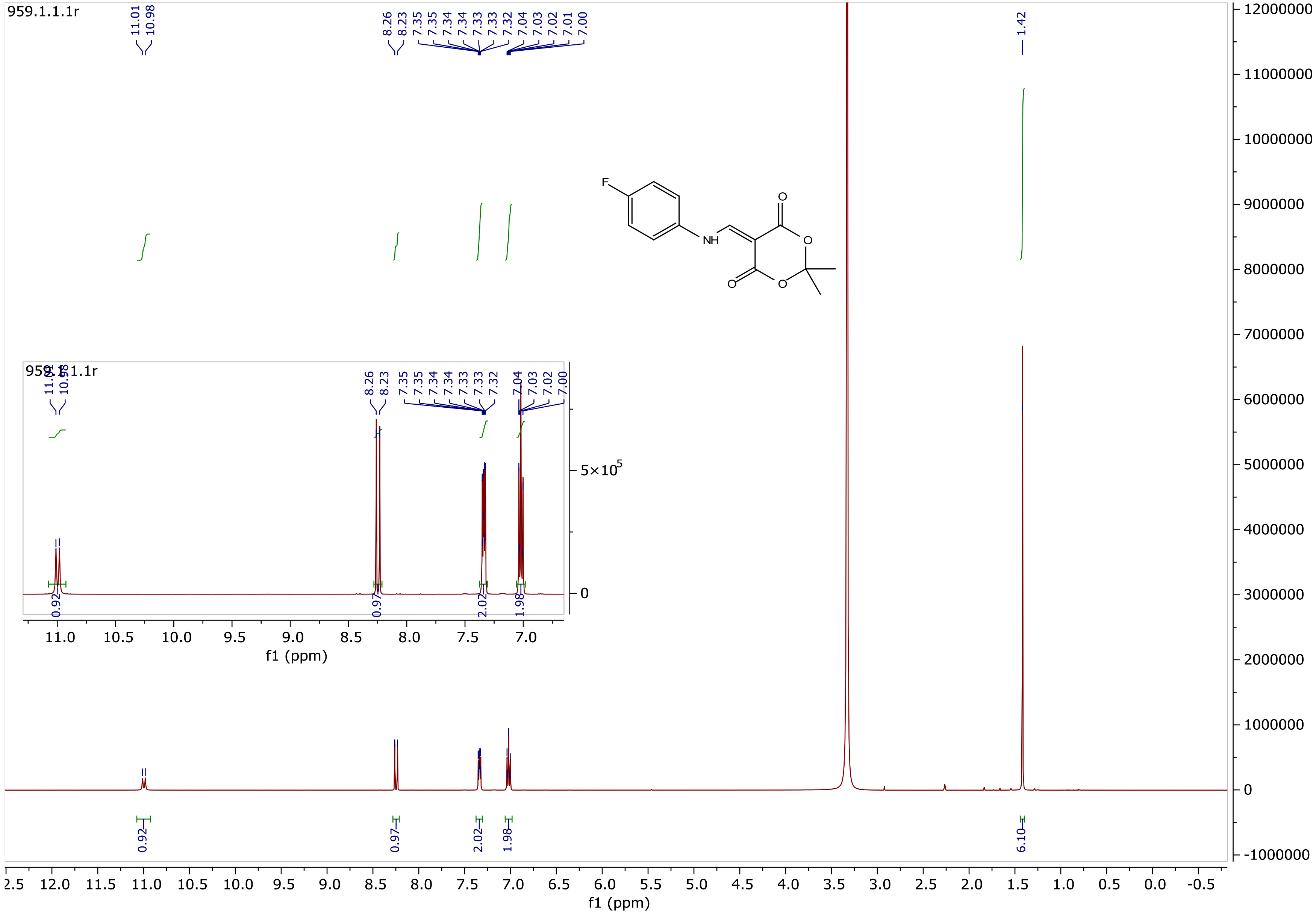

^{13}C NMR spectrum of 5-(((4-fluorophenyl)amino)methylene)-2,2-dimethyl-1,3-dioxane-4,6-dione (32a)

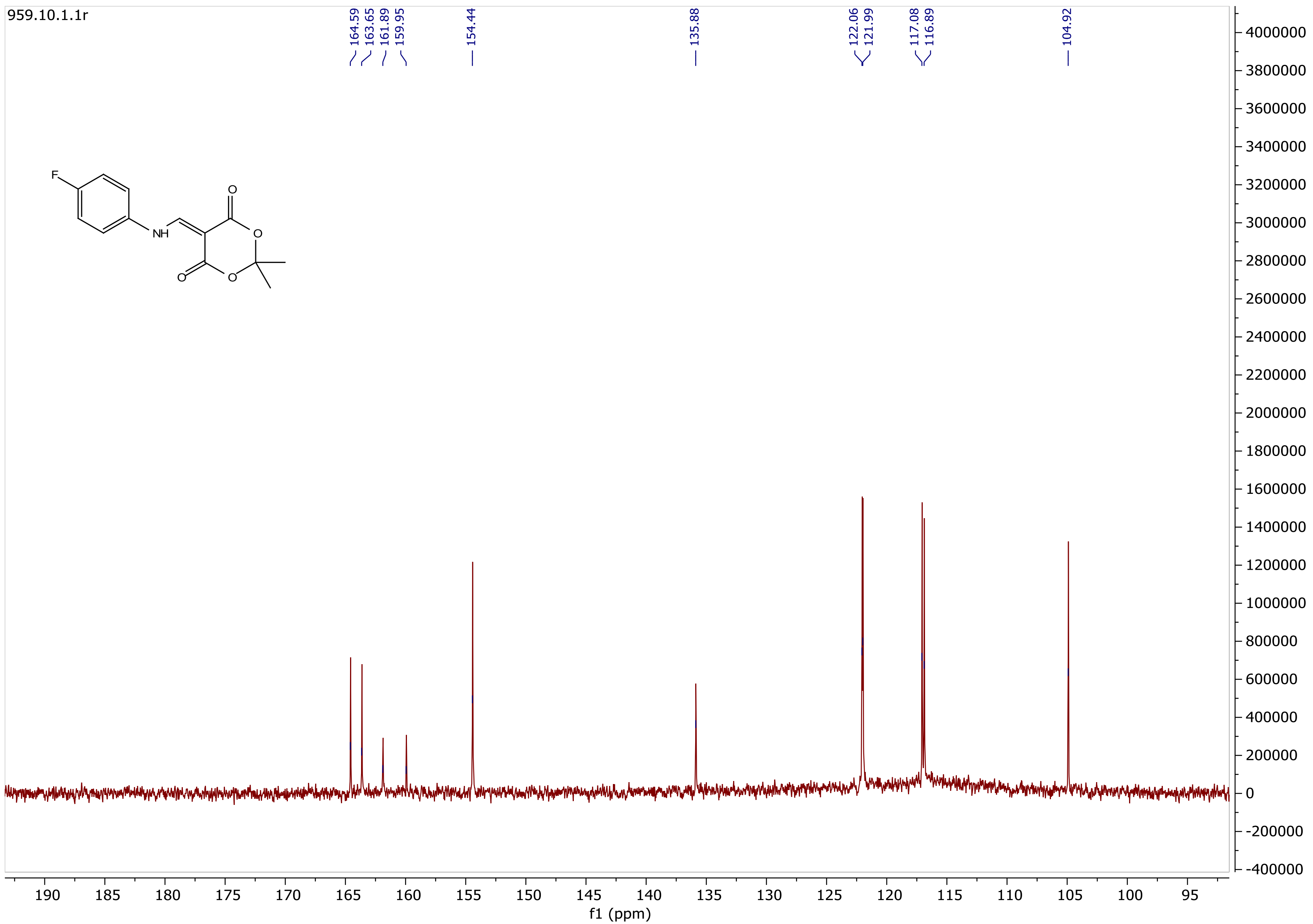

^{1}H NMR spectrum of 4-(((2,2-dimethyl-4,6-dioxo-1,3-dioxan-5-ylidene)methyl)amino)benzonitrile (32b)

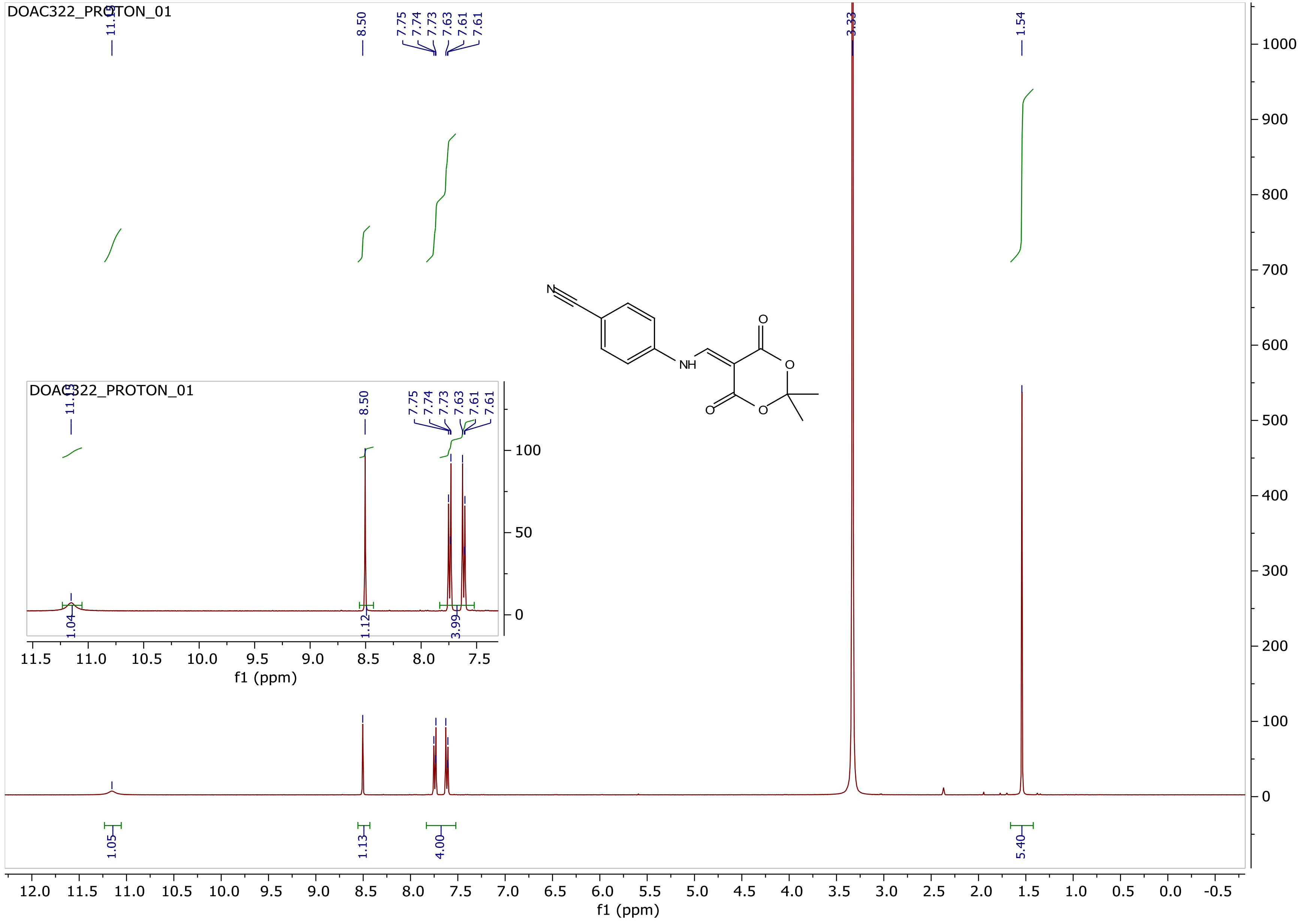

^{13}C NMR spectrum of 4-(((2,2-dimethyl-4,6-dioxo-1,3-dioxan-5-ylidene)methyl)amino)benzonitrile (32b)

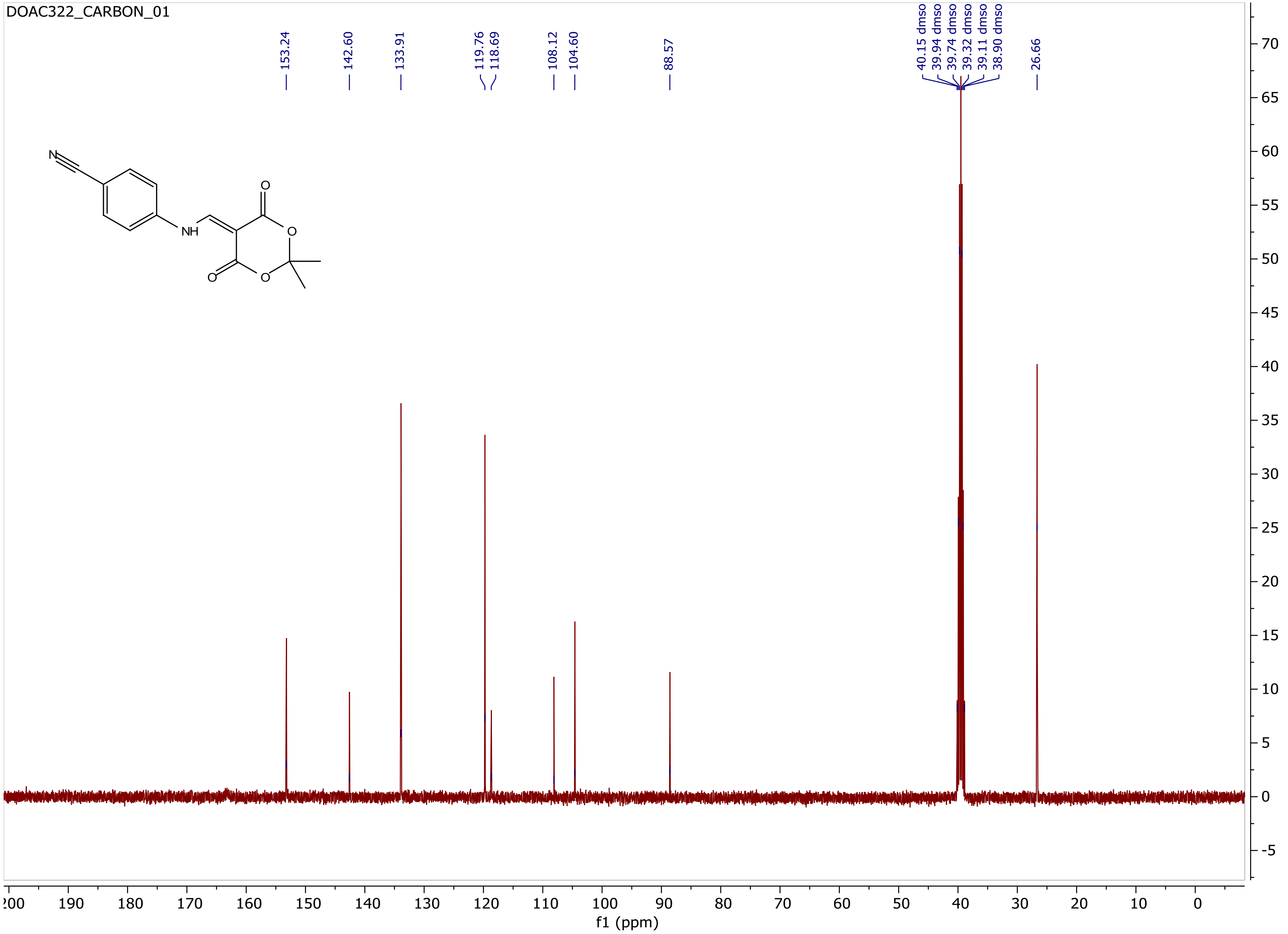

^{1}H NMR spectrum of mixture of 7-fluoro-and 5-fluoro-4-quinolone (23)

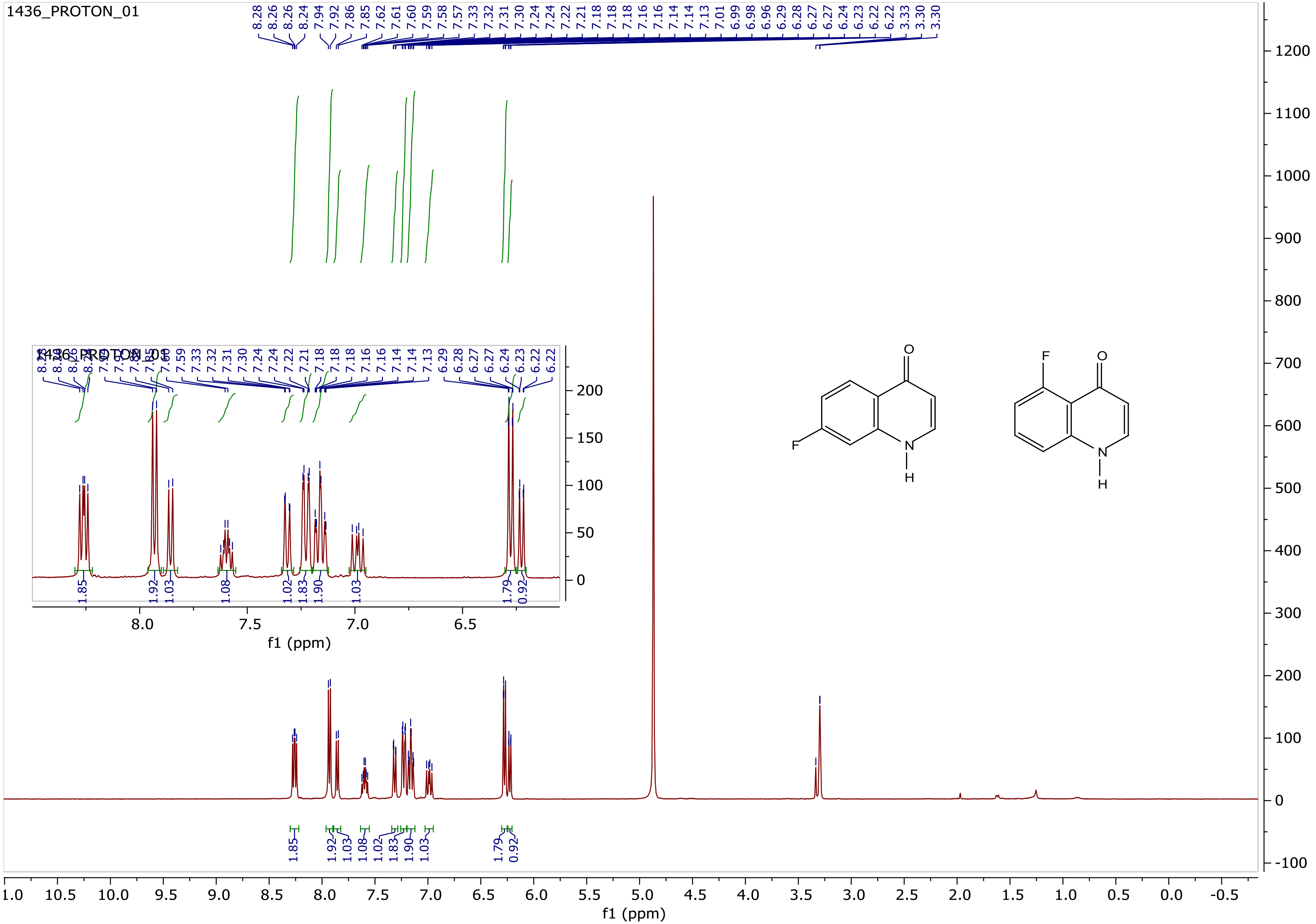

^{13}C NMR spectrum of mixture of 7-fluoro-and 5-fluoro-4-quinolone (23)

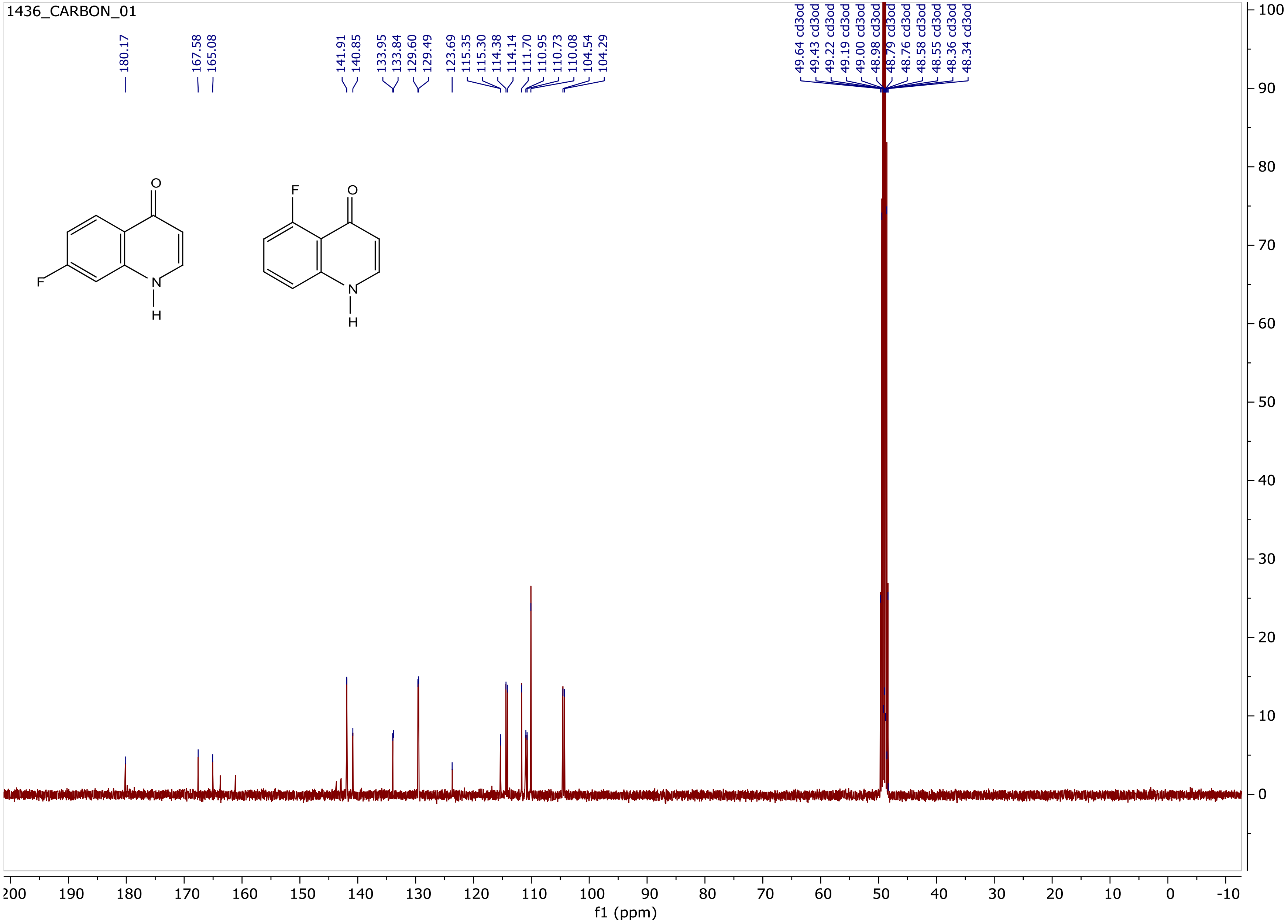

^{1}H NMR spectrum of mixture of 7-ciano-and 5-ciano-4-quinolone (24)

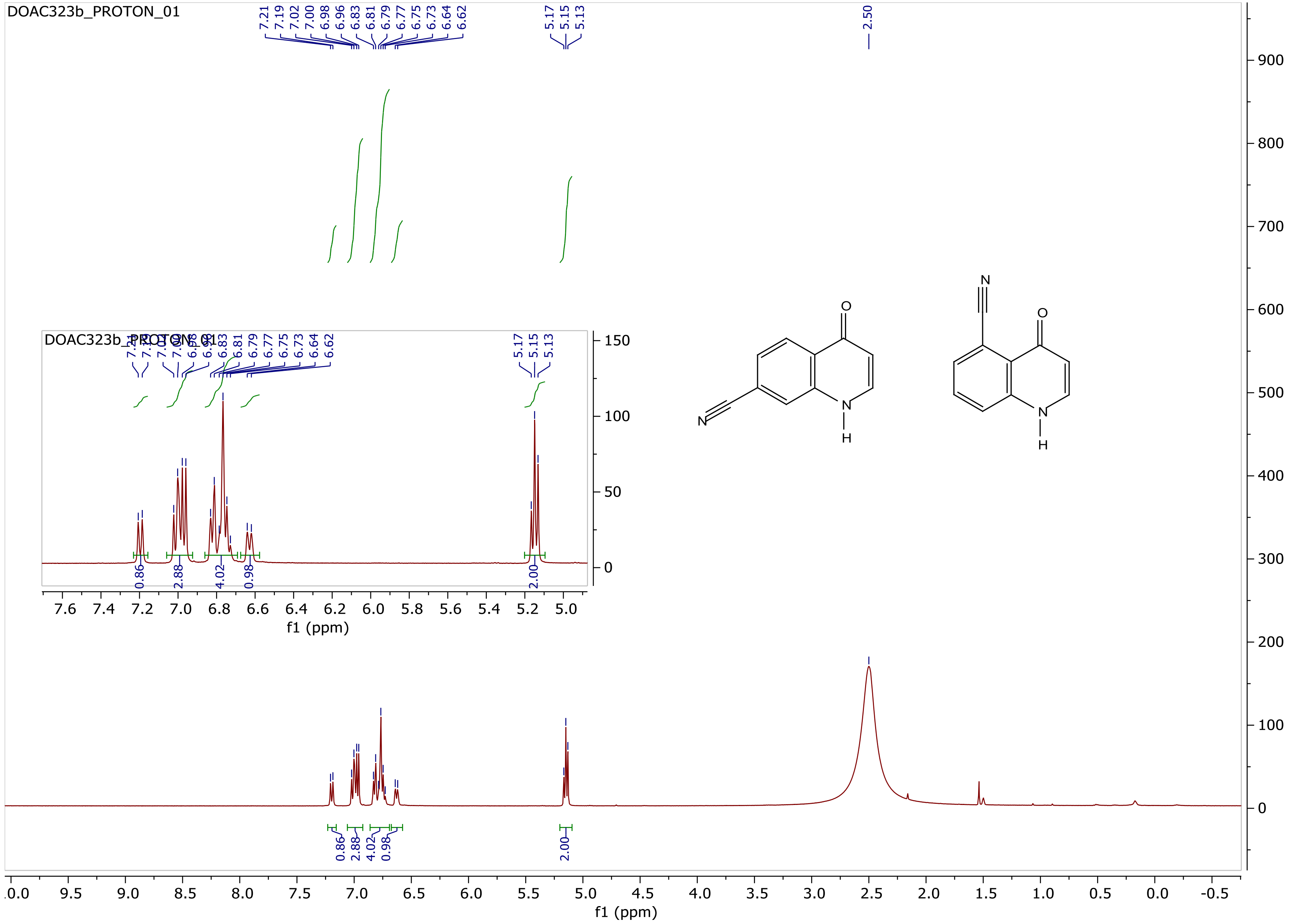

^{13}C NMR spectrum of mixture of 7-ciano-and 5-ciano-4-quinolone (24)

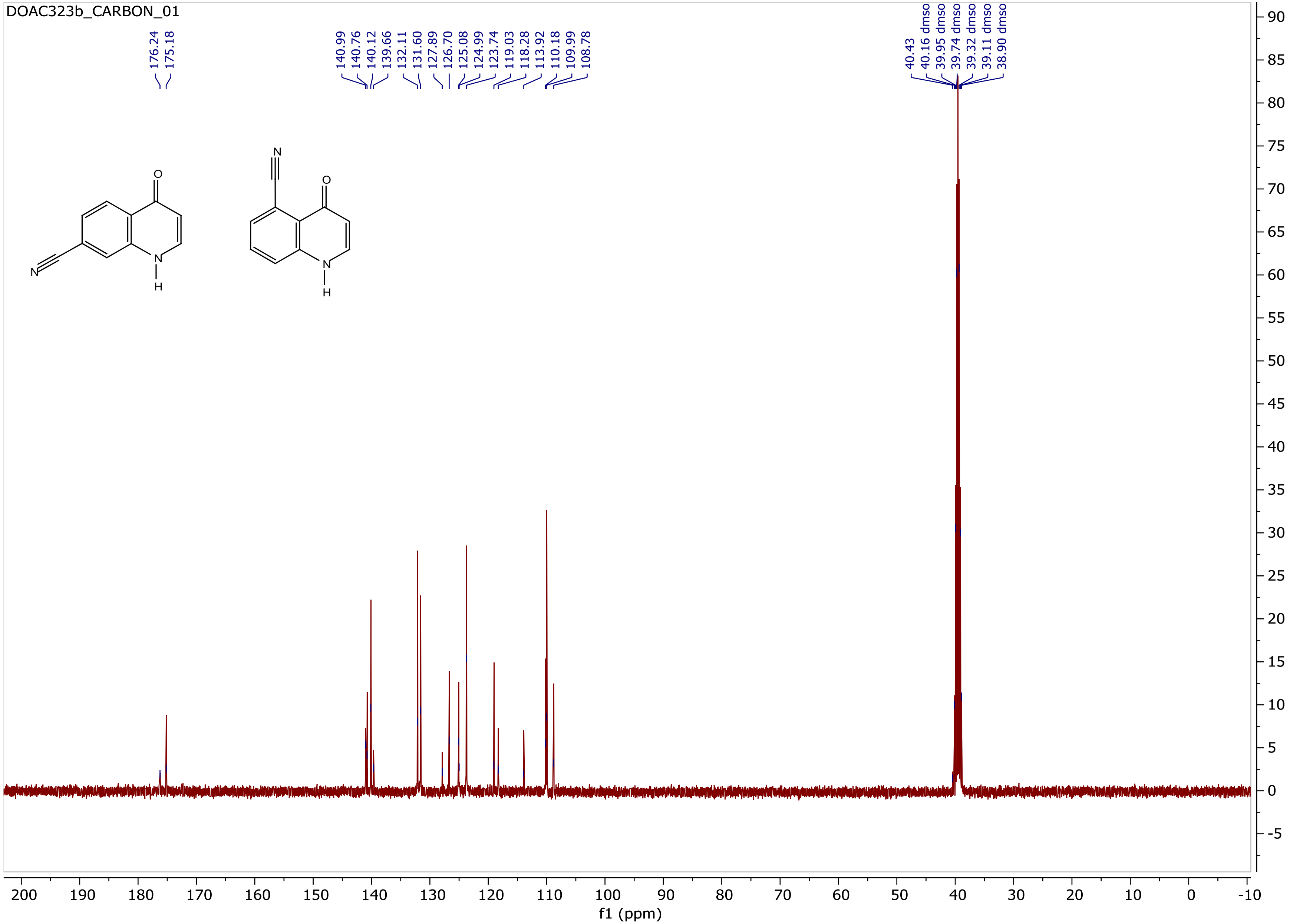

^{1}H NMR spectrum of mixture of 7-nitro-and 5-nitro-4-quinolone (25)

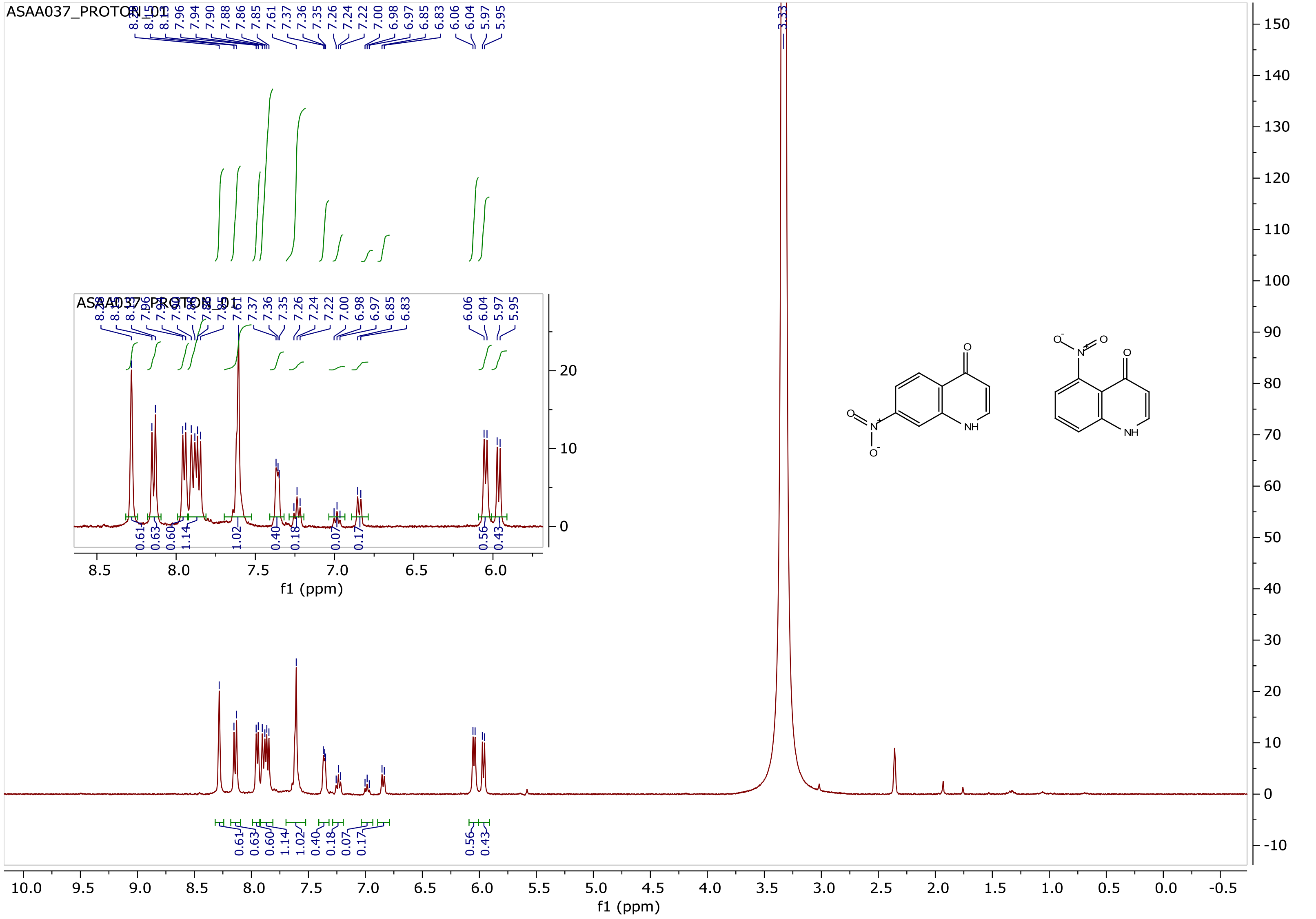

^{13}C NMR spectrum of mixture of 7-nitro-and 5-nitro-4-quinolone (AS12)

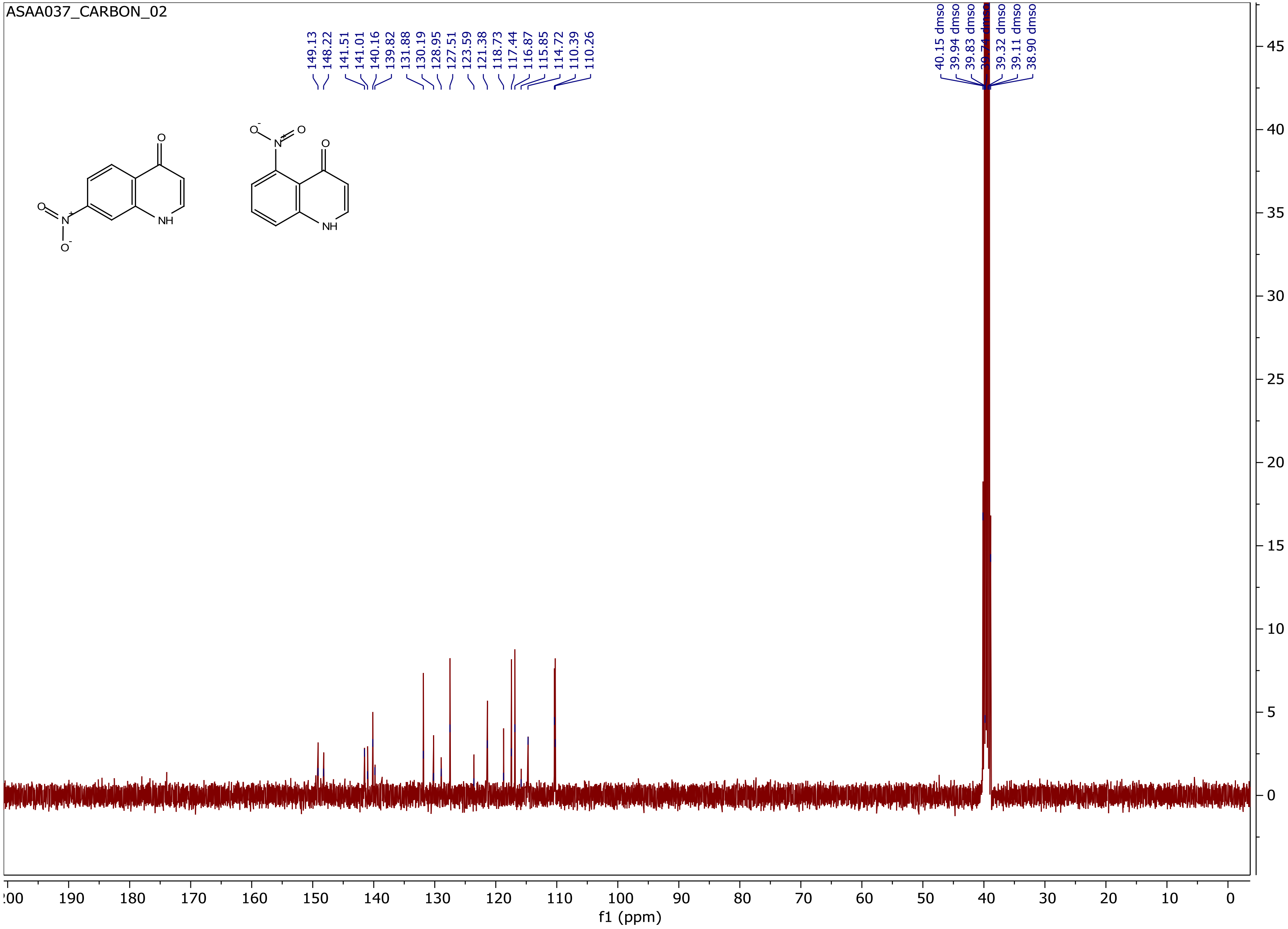

^{1}H NMR spectrum of 6-fluoro-4-quinolone (33a)

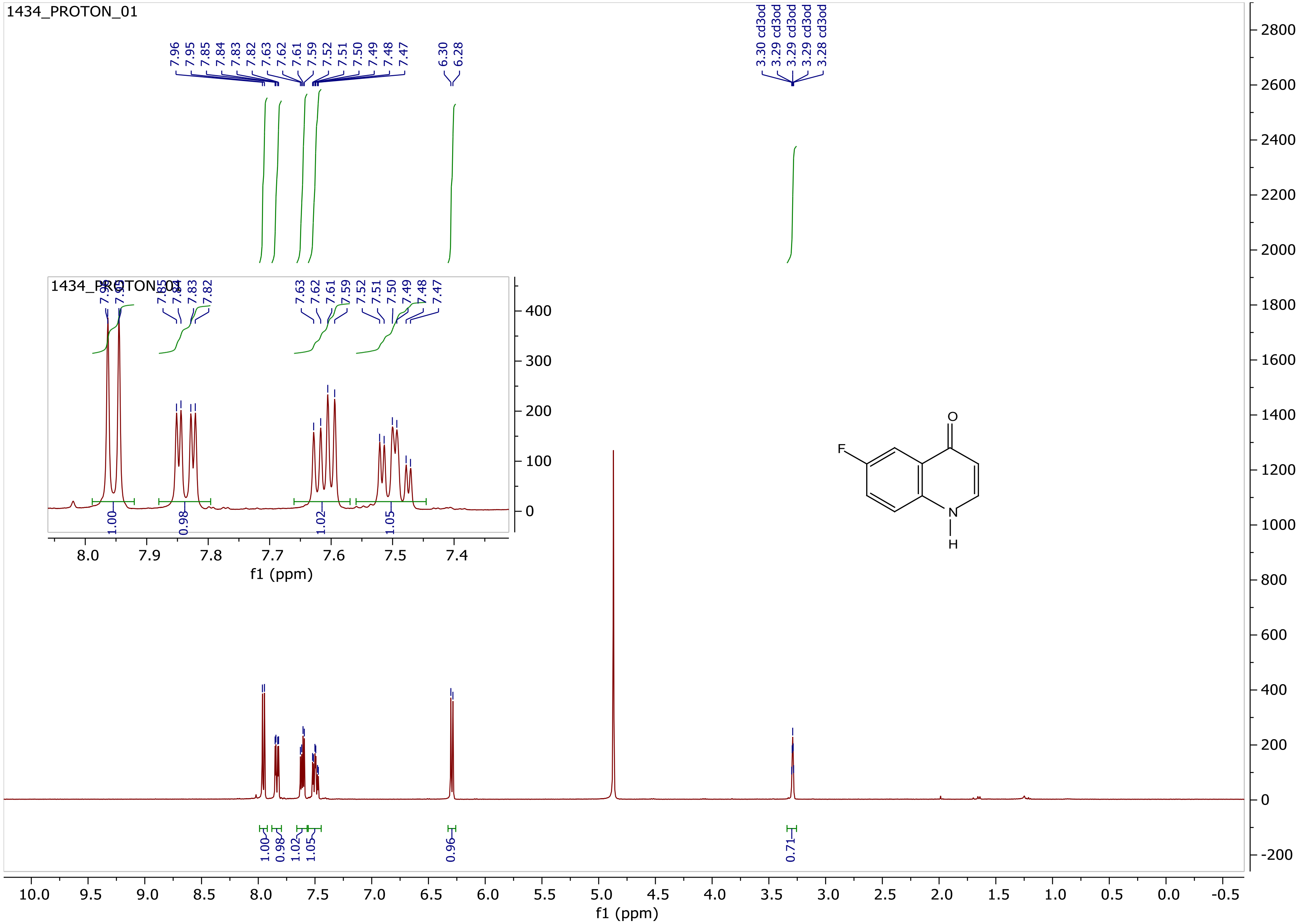

^{13}C NMR spectrum of 6-fluoro-4-quinolone (33a)

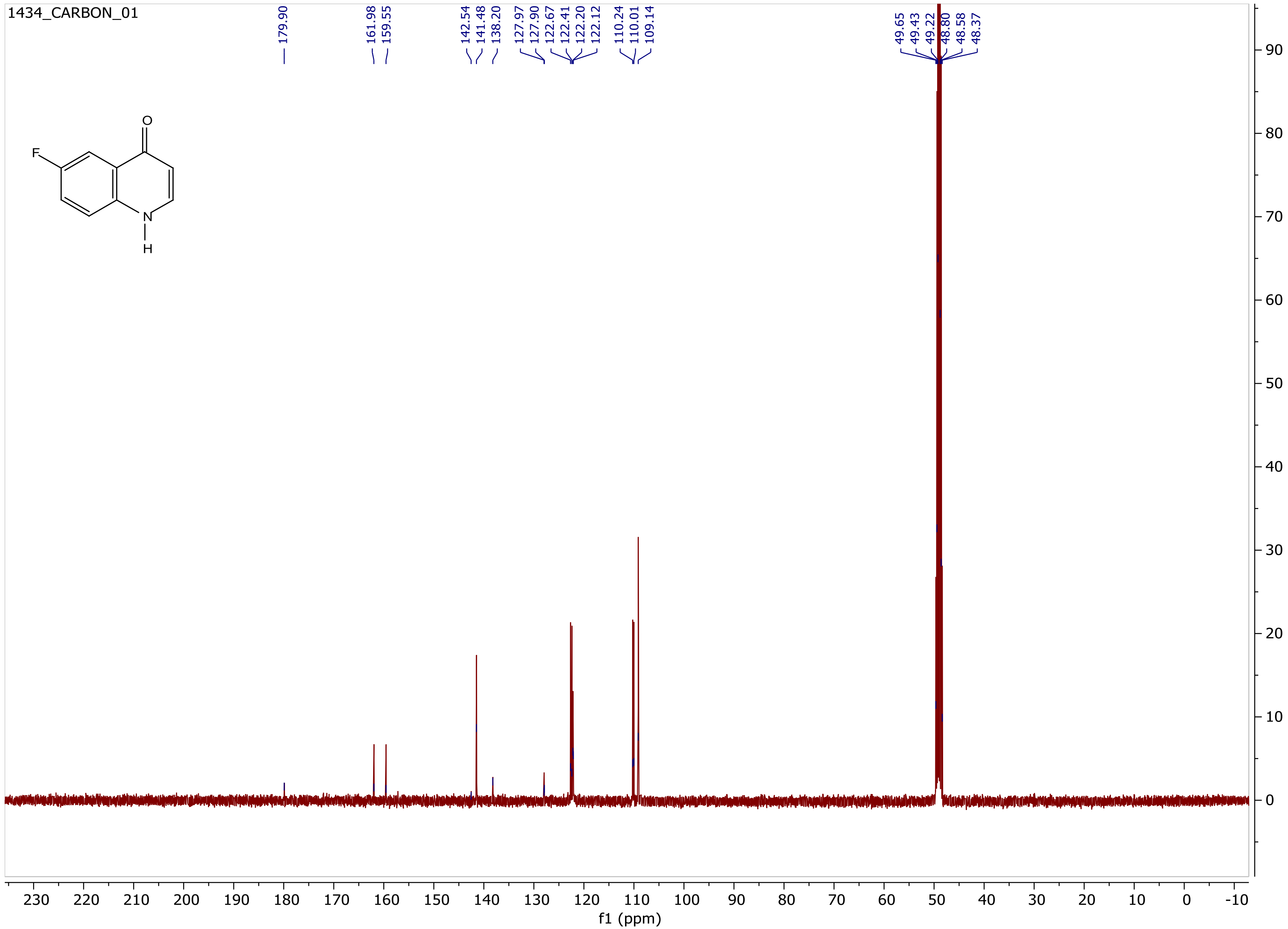

^{1}H NMR spectrum of 6-ciano-4-quinolone (33b)

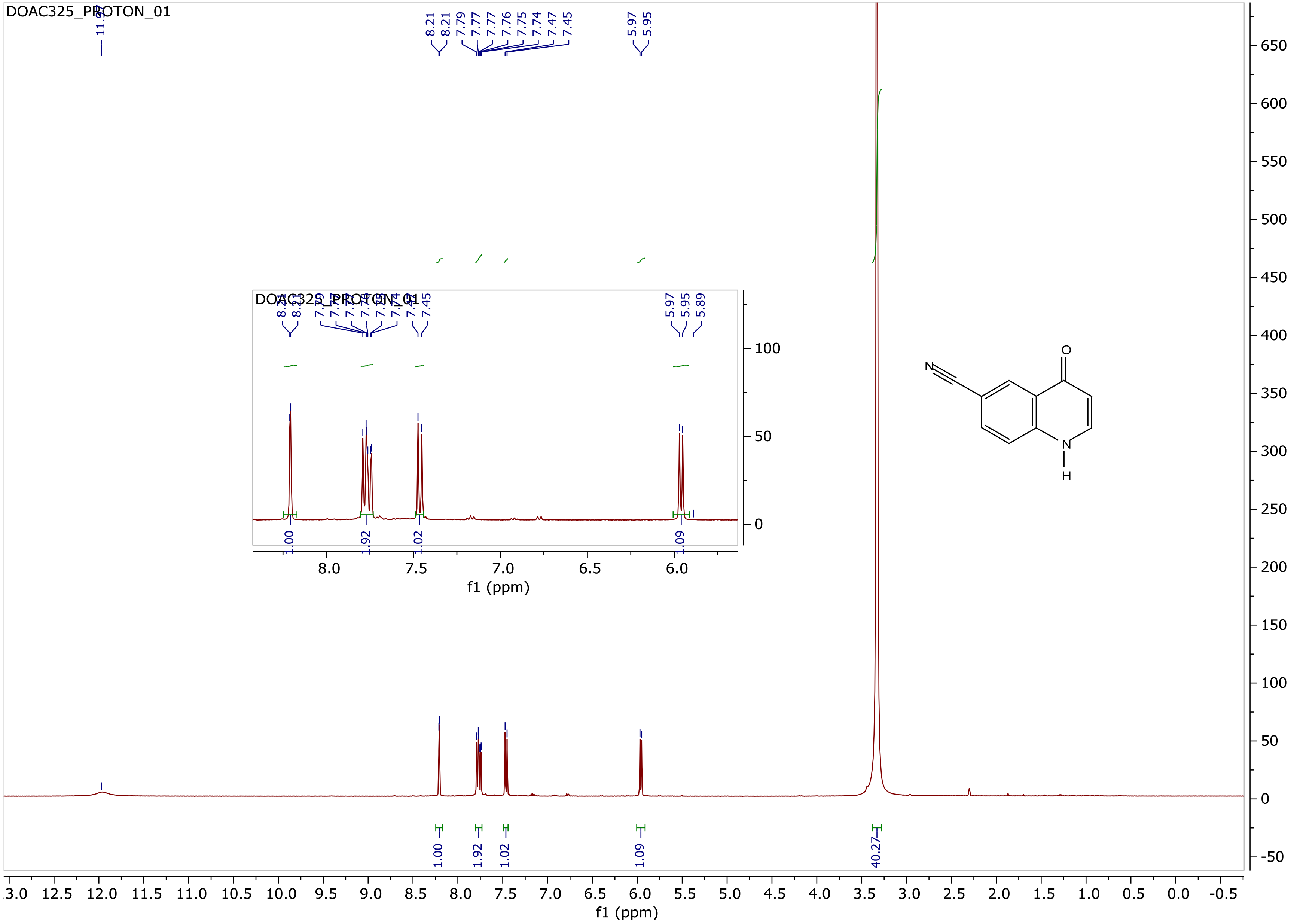

^{13}C NMR spectrum of 6-ciano-4-quinolone (33b)

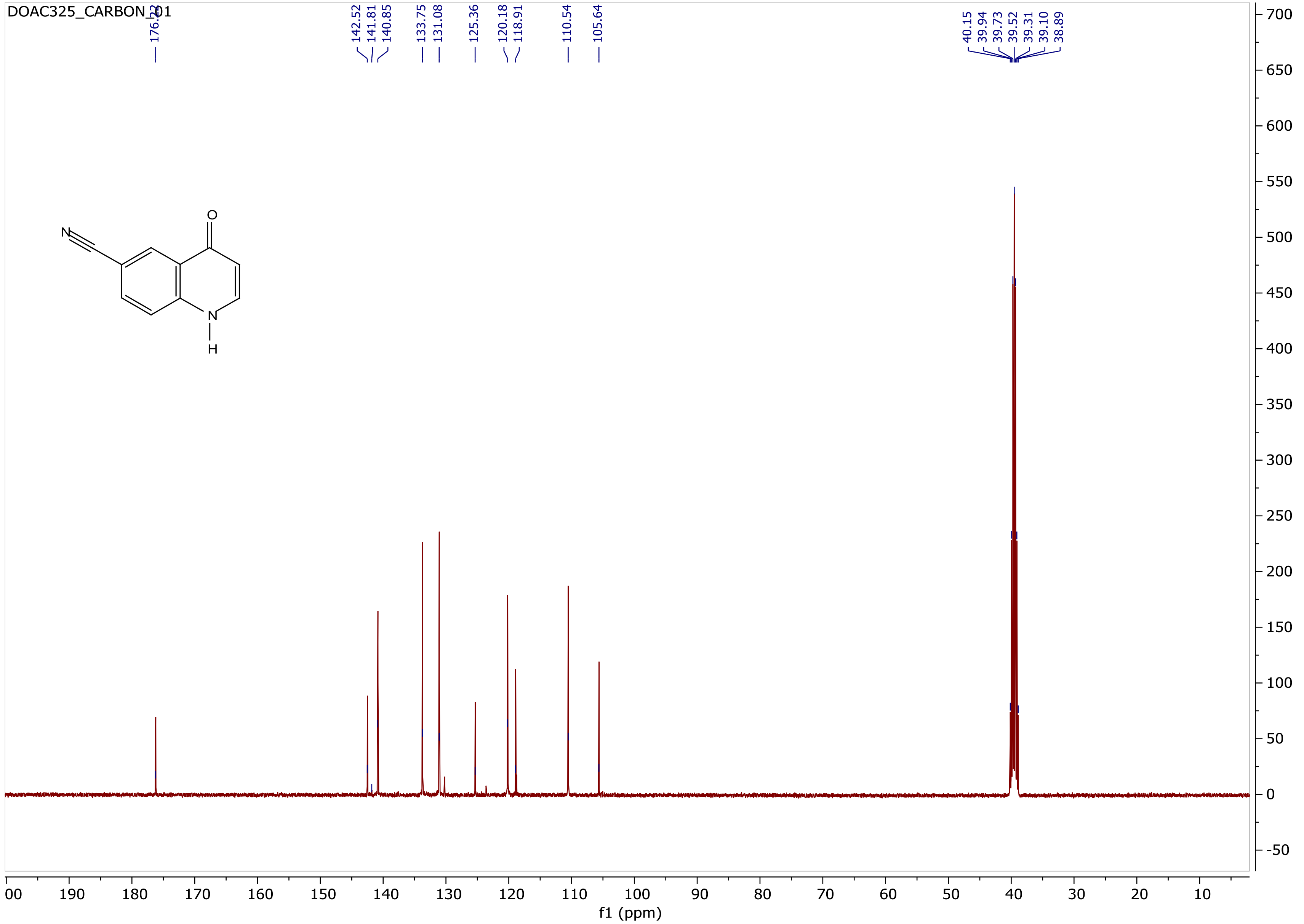

^{1}H NMR spectrum of 7-fluoro-4-chloroquinolines (26)

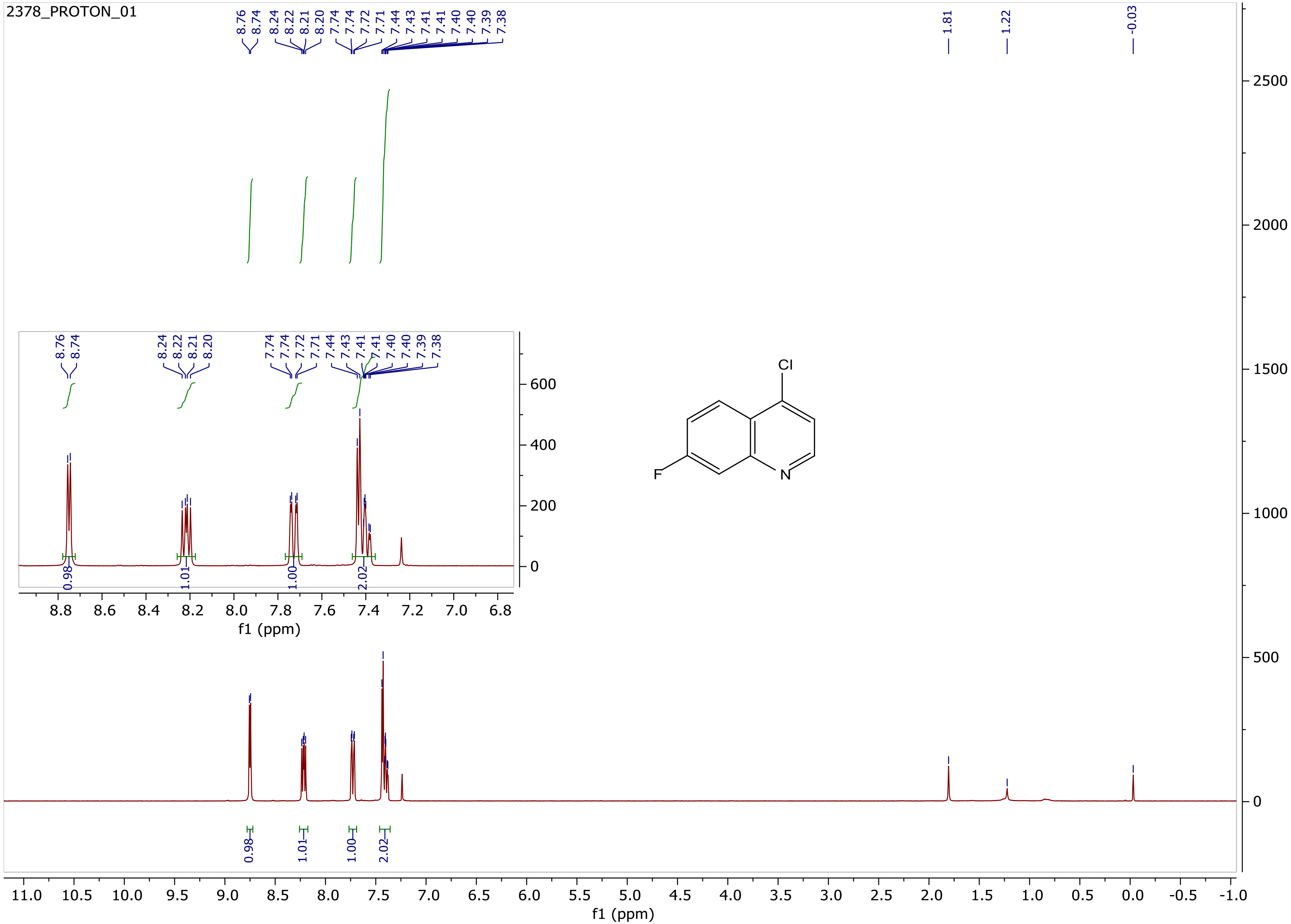

^{13}C NMR spectrum of 7-fluoro-4-chloroquinolines (26)

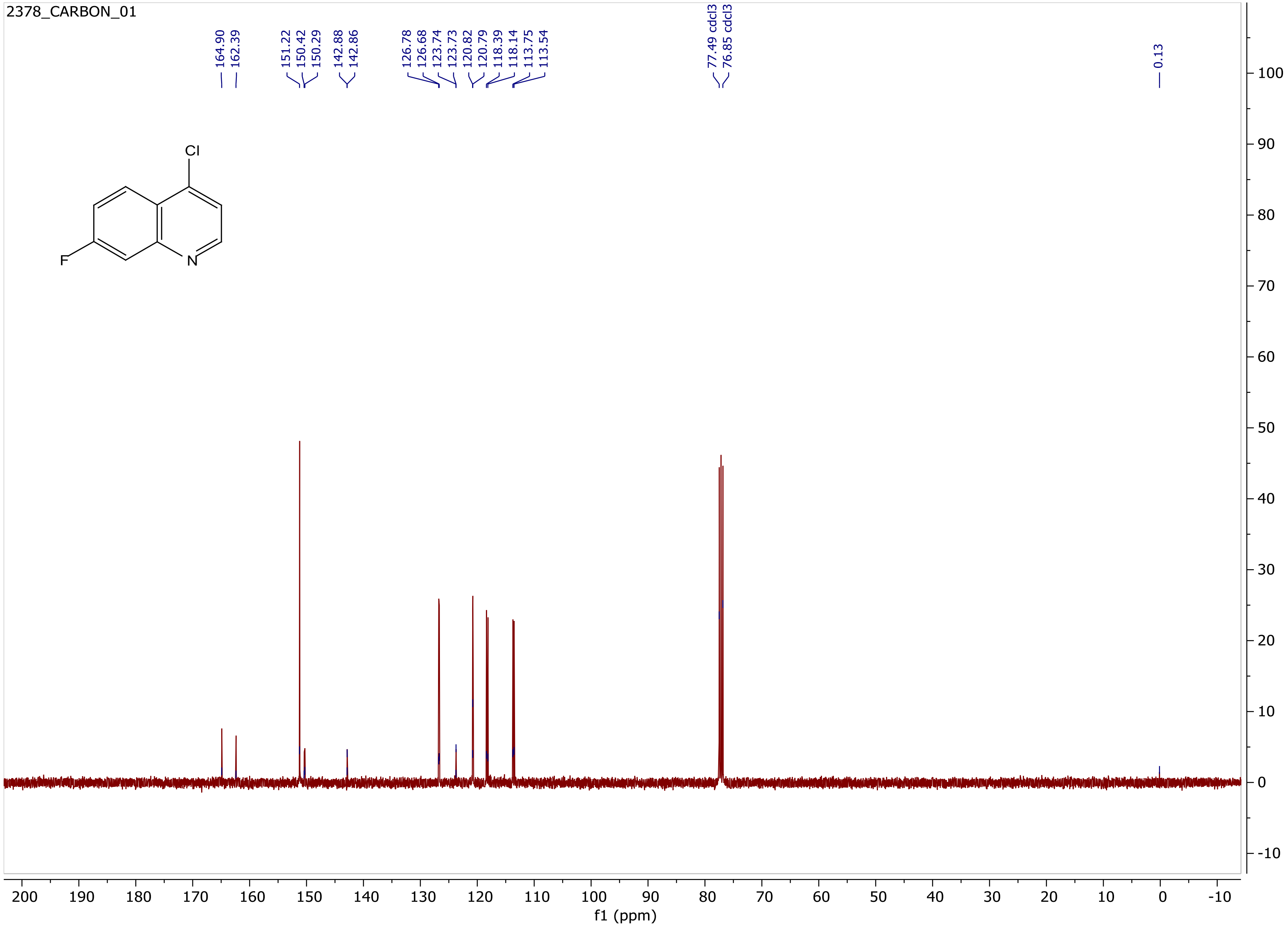

^{1}H NMR spectrum of 5-fluoro-4-chloroquinolines (27)

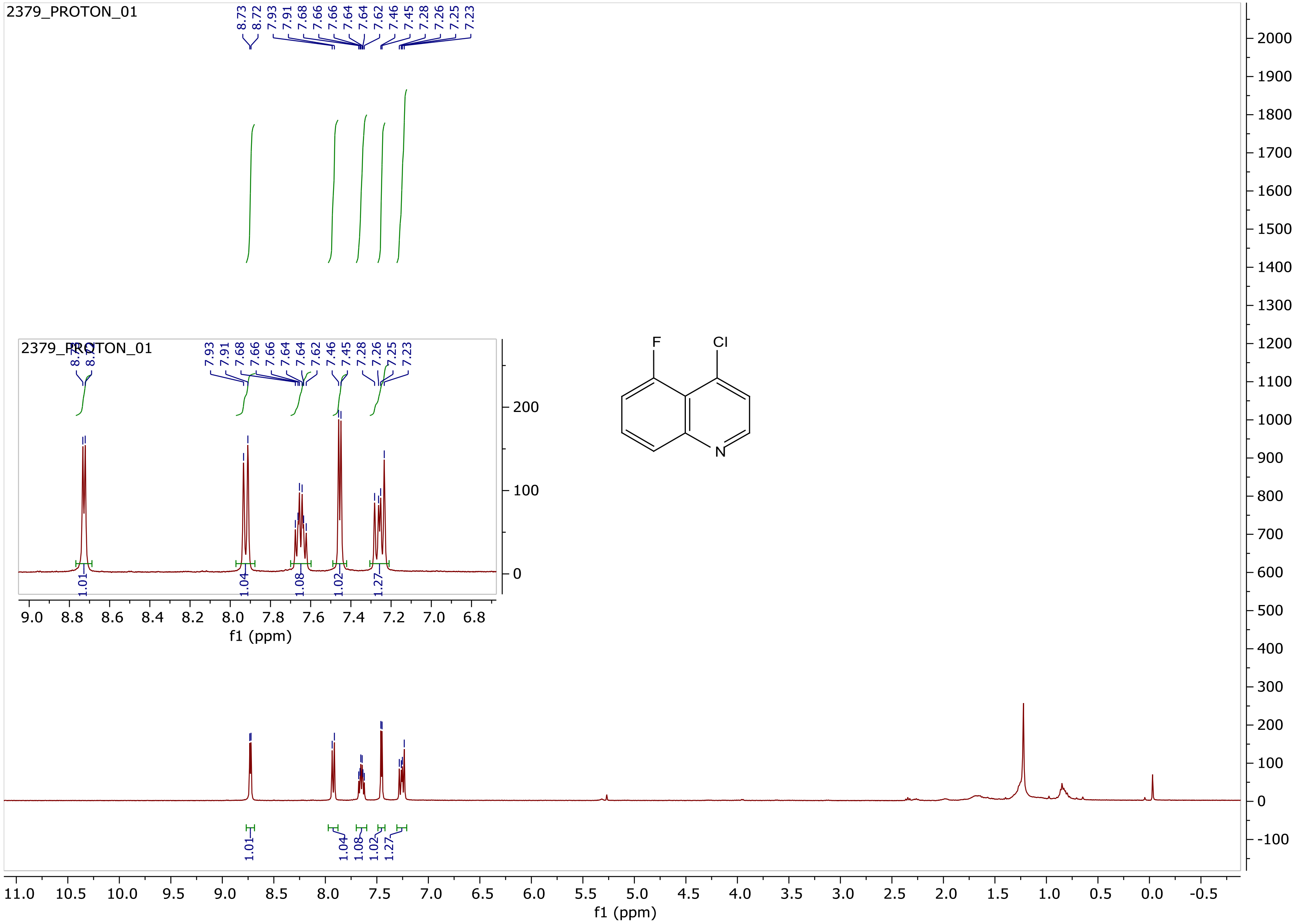

^{13}C NMR spectrum of 5-fluoro-4-chloroquinolines (27)

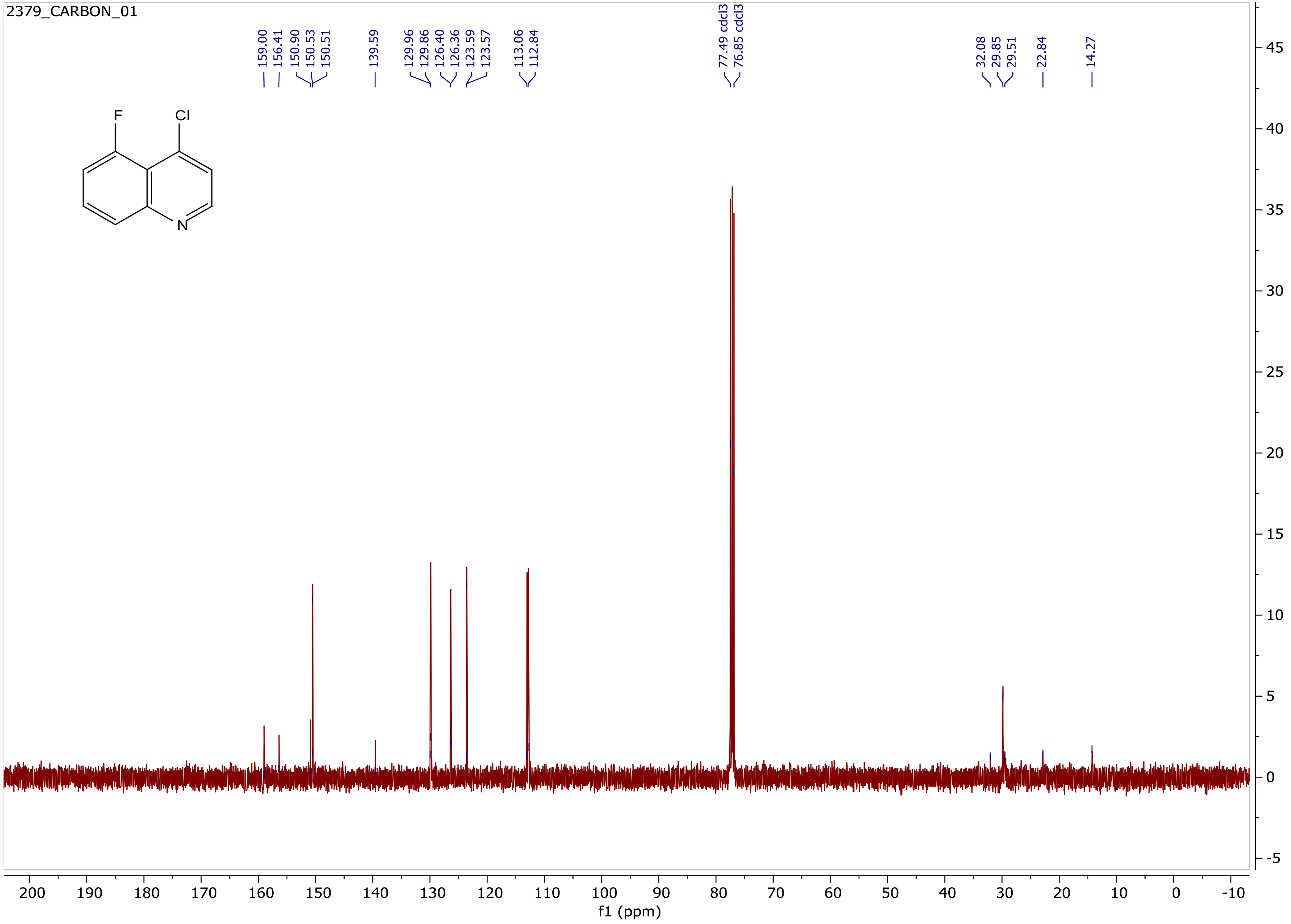

^{1}H NMR spectrum of 7-ciano-4-chloroquinolines (28)

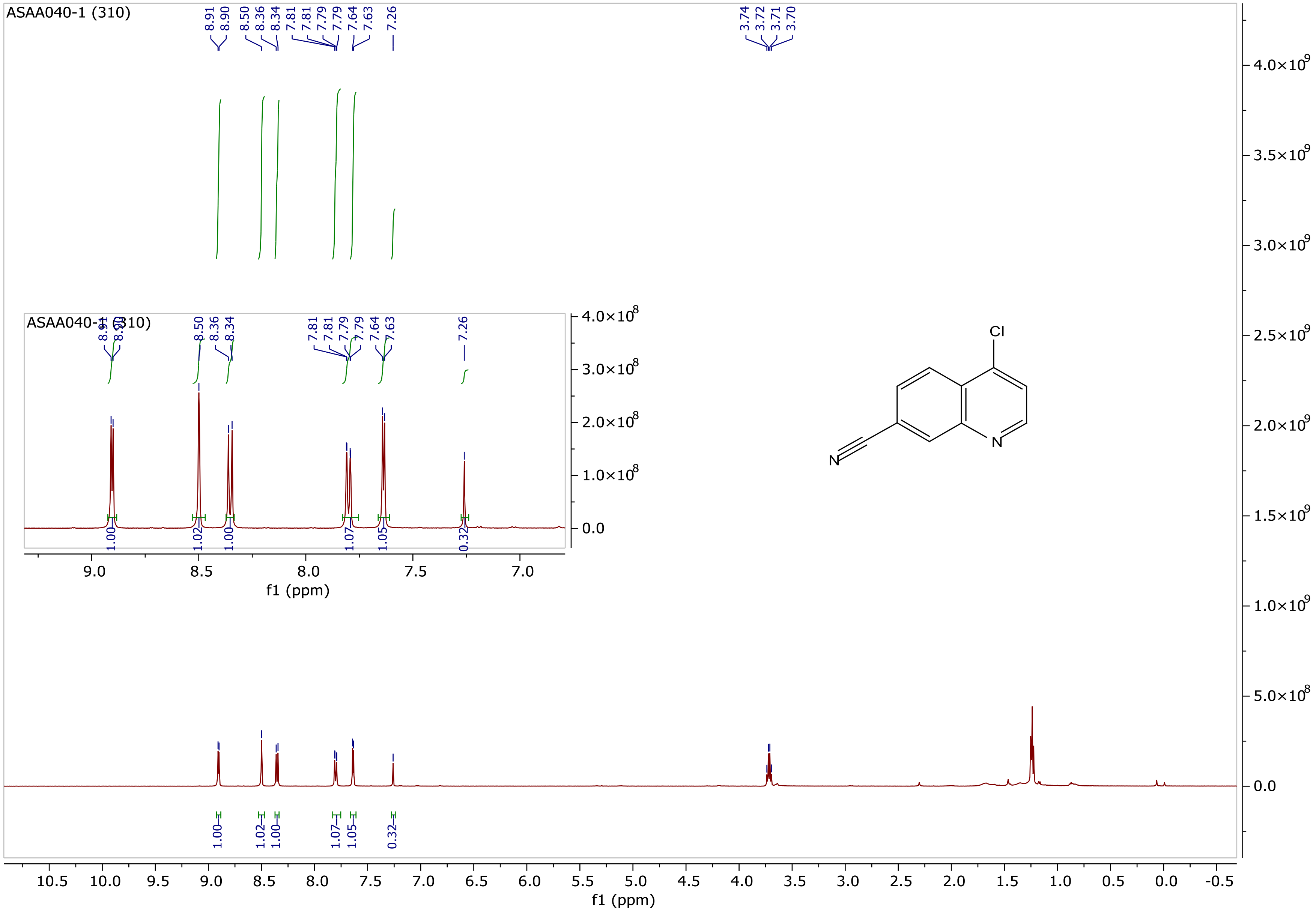

^{13}C NMR spectrum of 7-ciano-4-chloroquinolines (28)

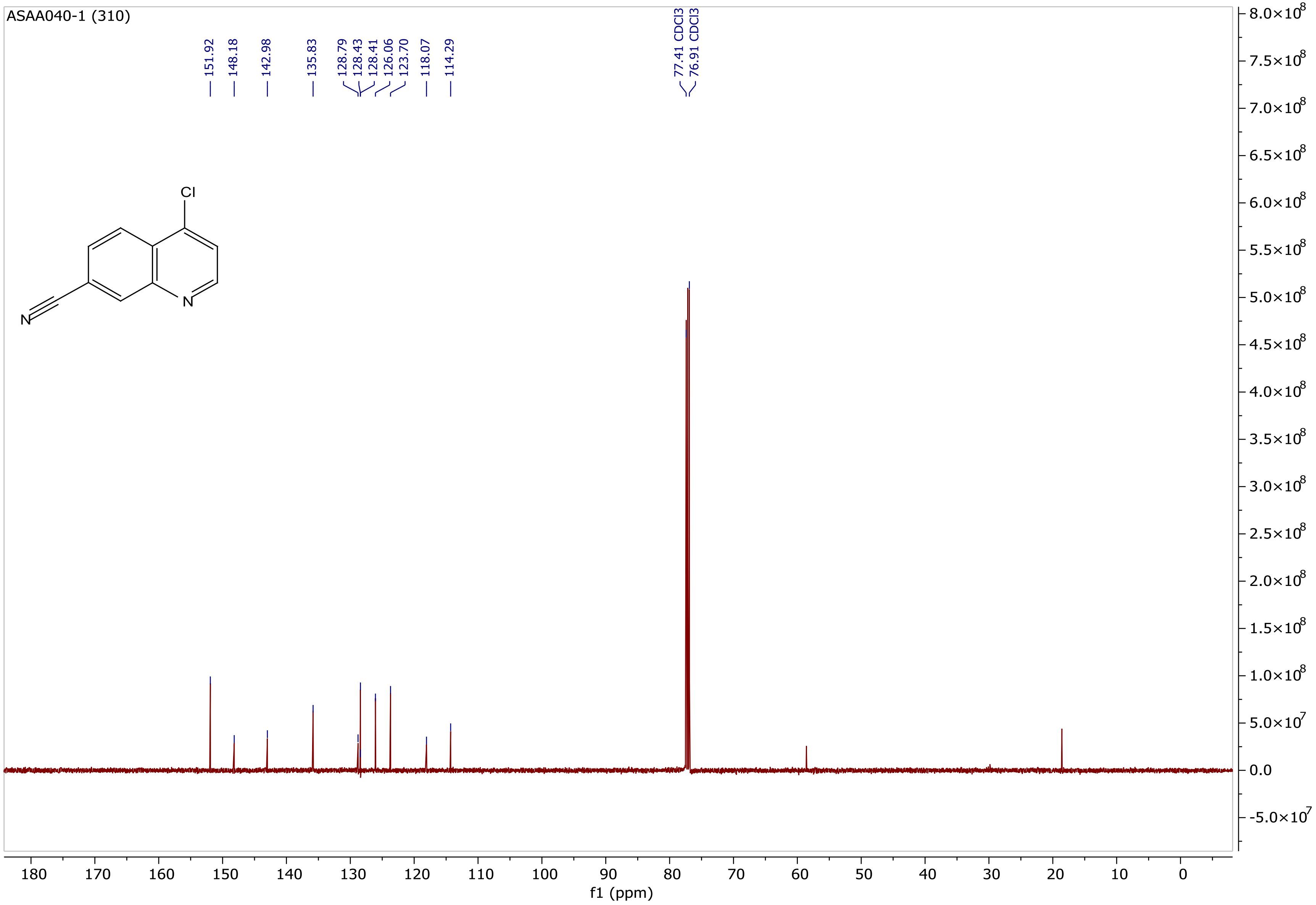

^{1}H NMR spectrum of 5-ciano-4-chloroquinolines (15)

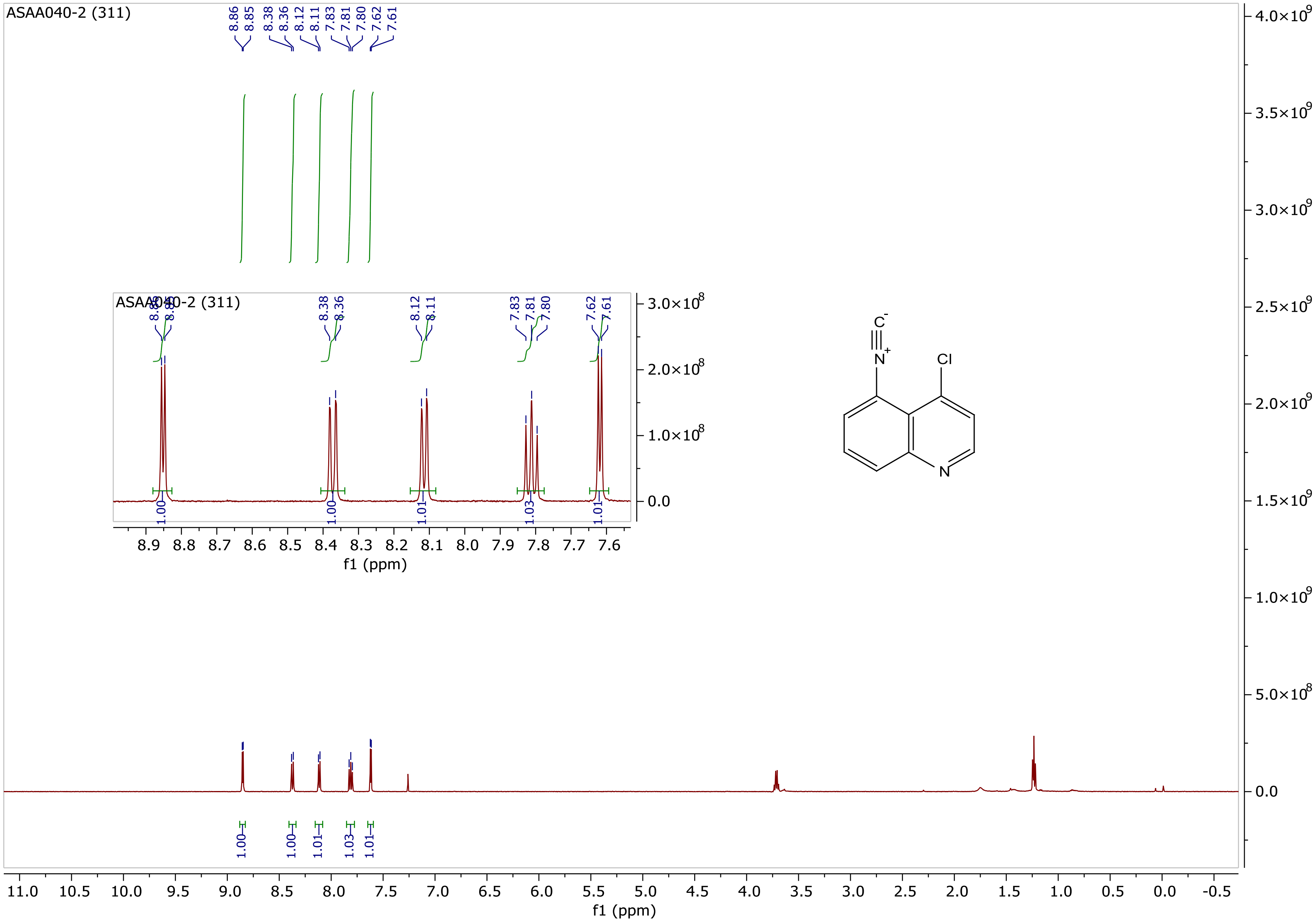

^{13}C NMR spectrum of 5-ciano-4-chloroquinolines (15)

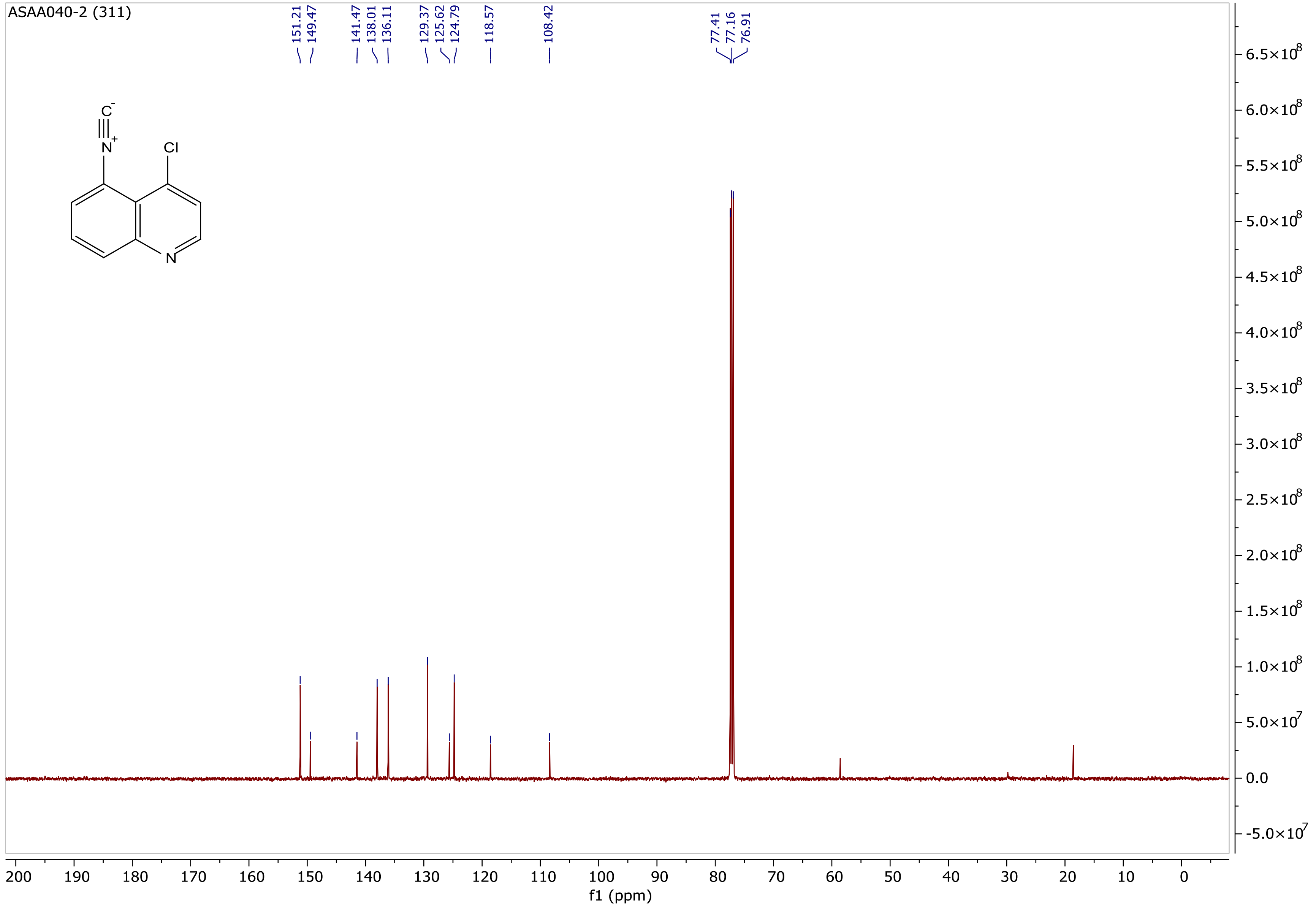

^{1}H NMR spectrum of 7-nitro-4-chloroquinoline (29)

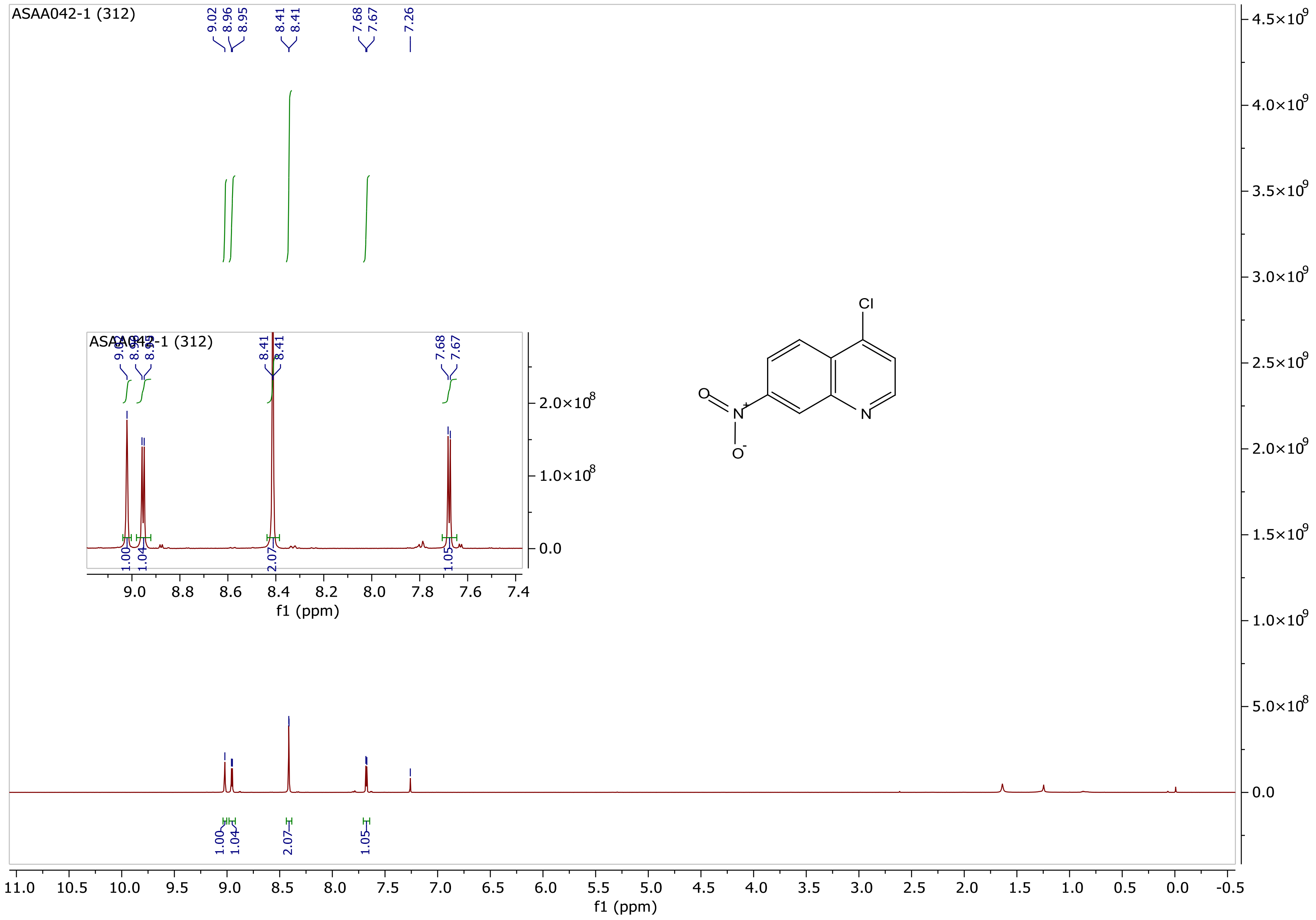

^{13}C NMR spectrum of 7-nitro-4-chloroquinoline (29)

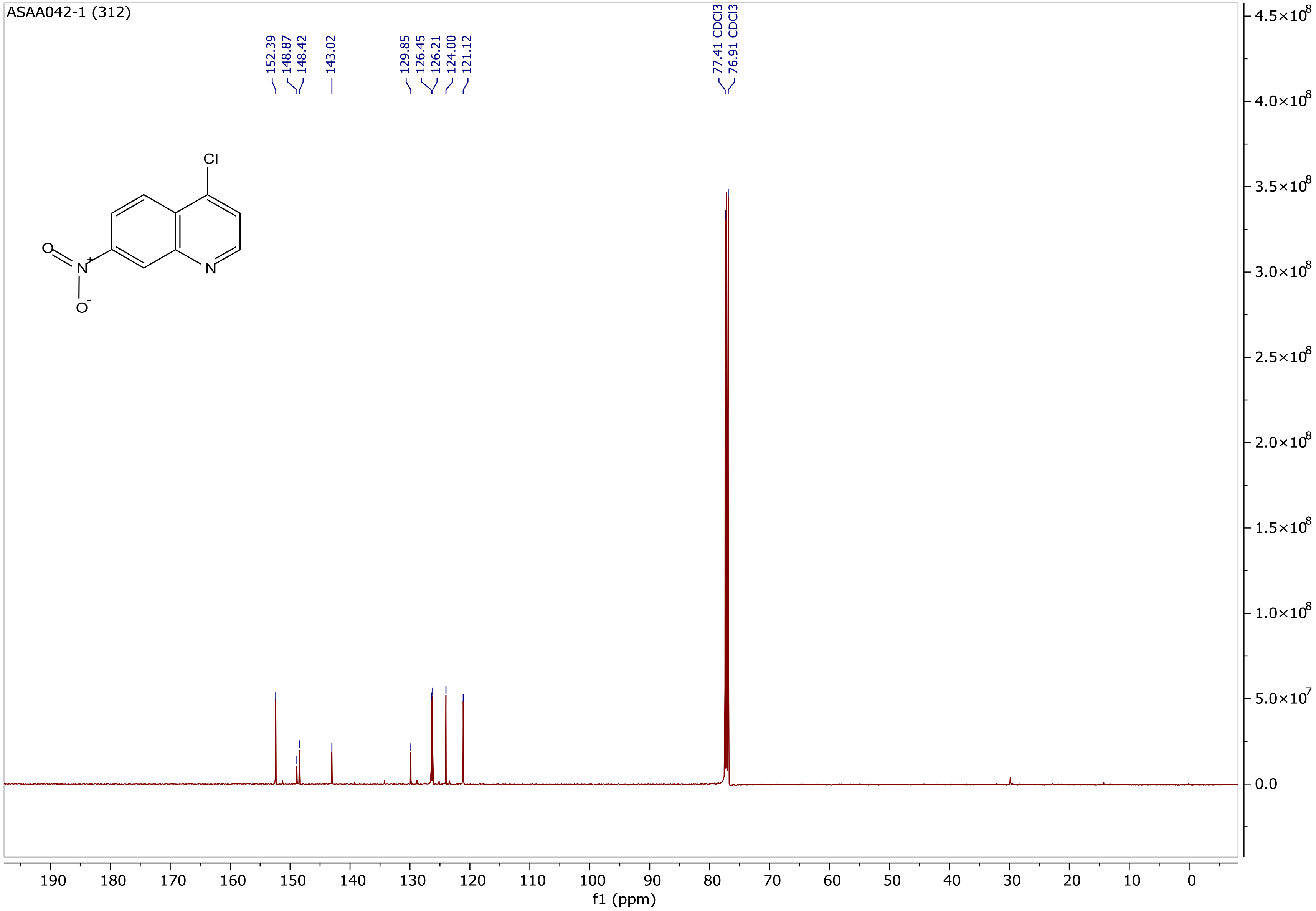

^{1}H NMR spectrum of 5-nitro-4-chloroquinoline (30)

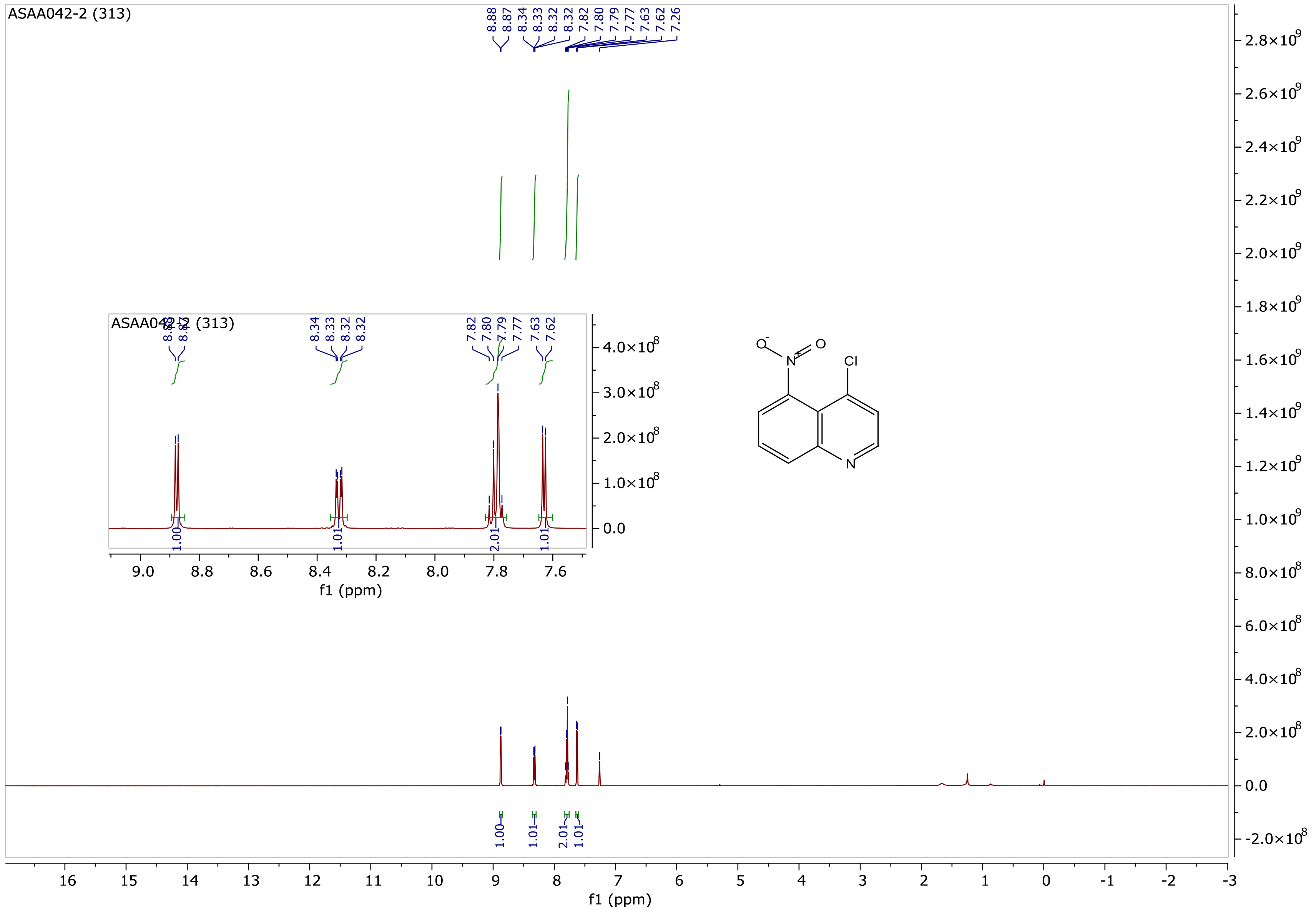

^{13}C NMR spectrum of 5-nitro-4-chloroquinoline (30)

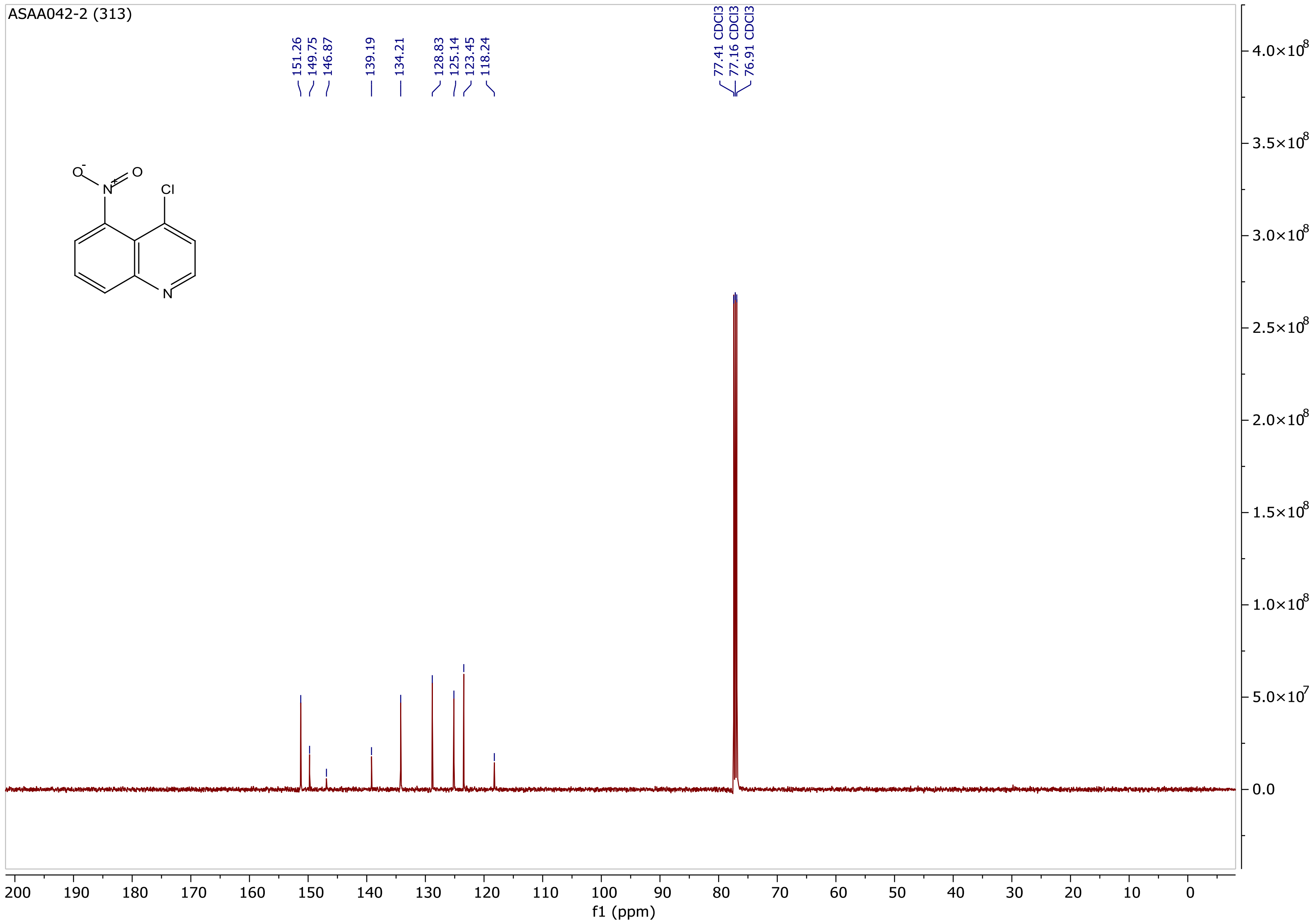

^{1}H NMR spectrum of 6-fluoro-4-chloroquinoline (34)

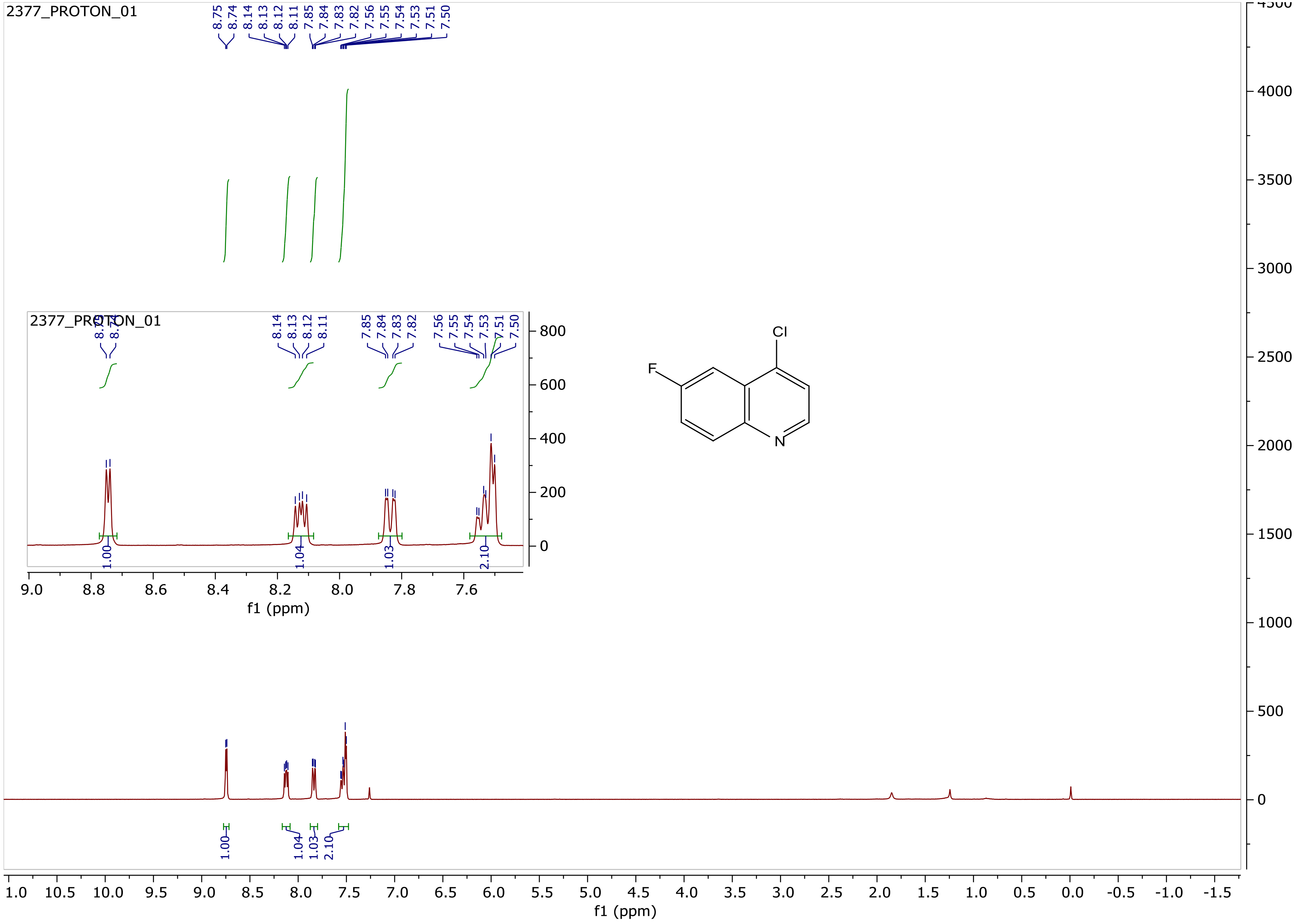

^{13}C NMR spectrum of 6-fluoro-4-chloroquinoline (34)

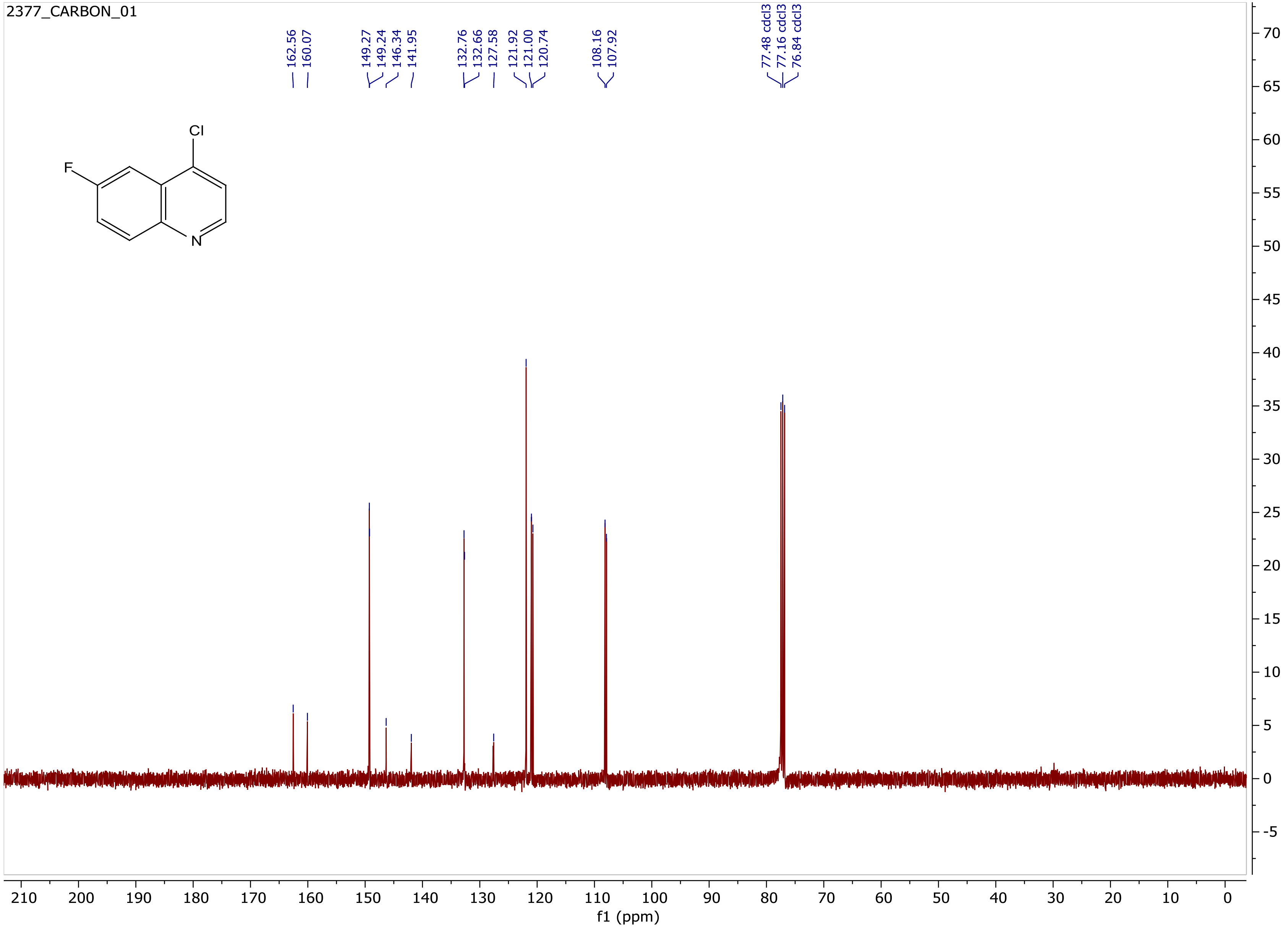

^{1}H NMR spectrum of 6-ciano-4-chloroquinoline (35)

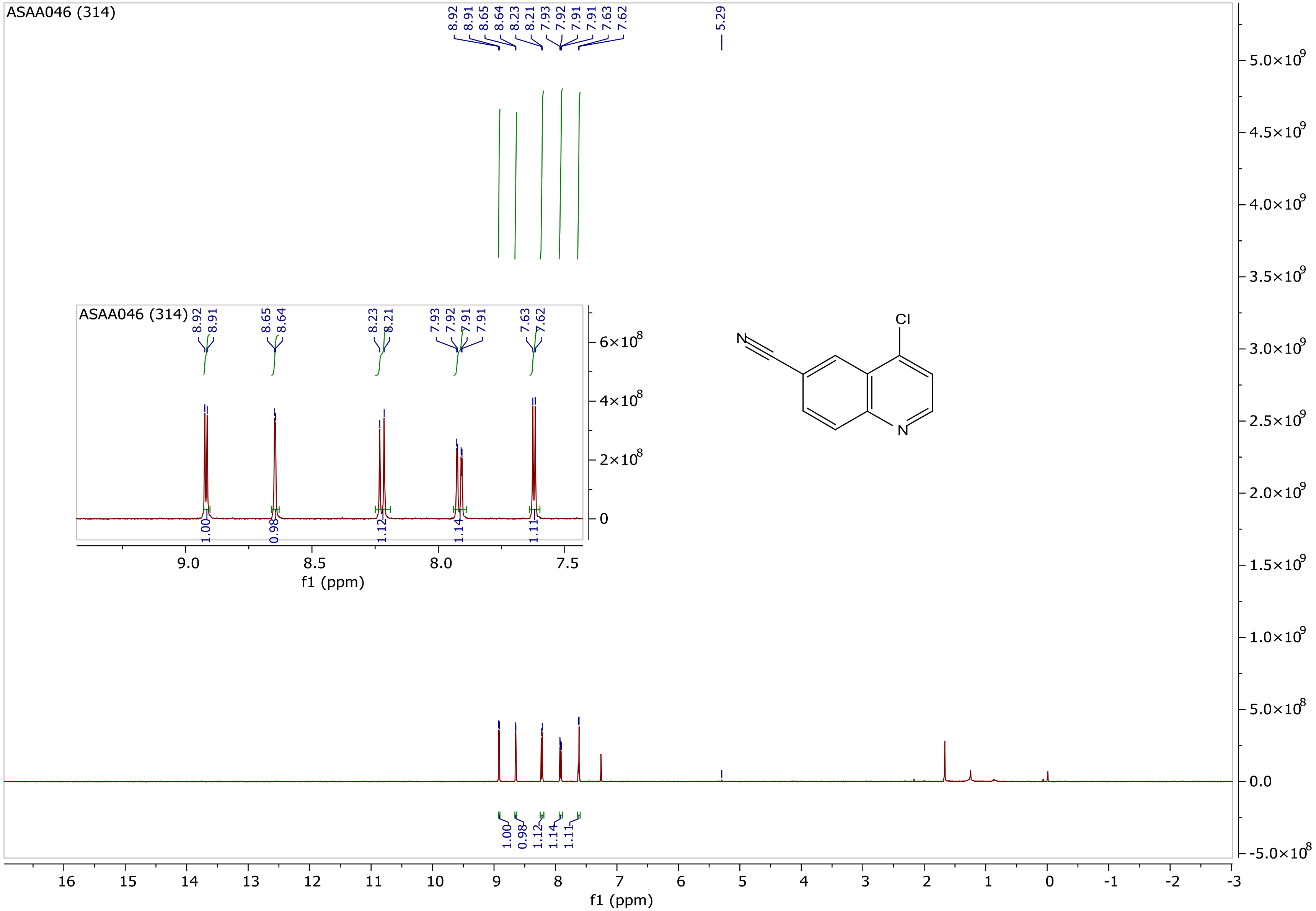

^{13}C NMR spectrum of 6-ciano-4-chloroquinoline (35)

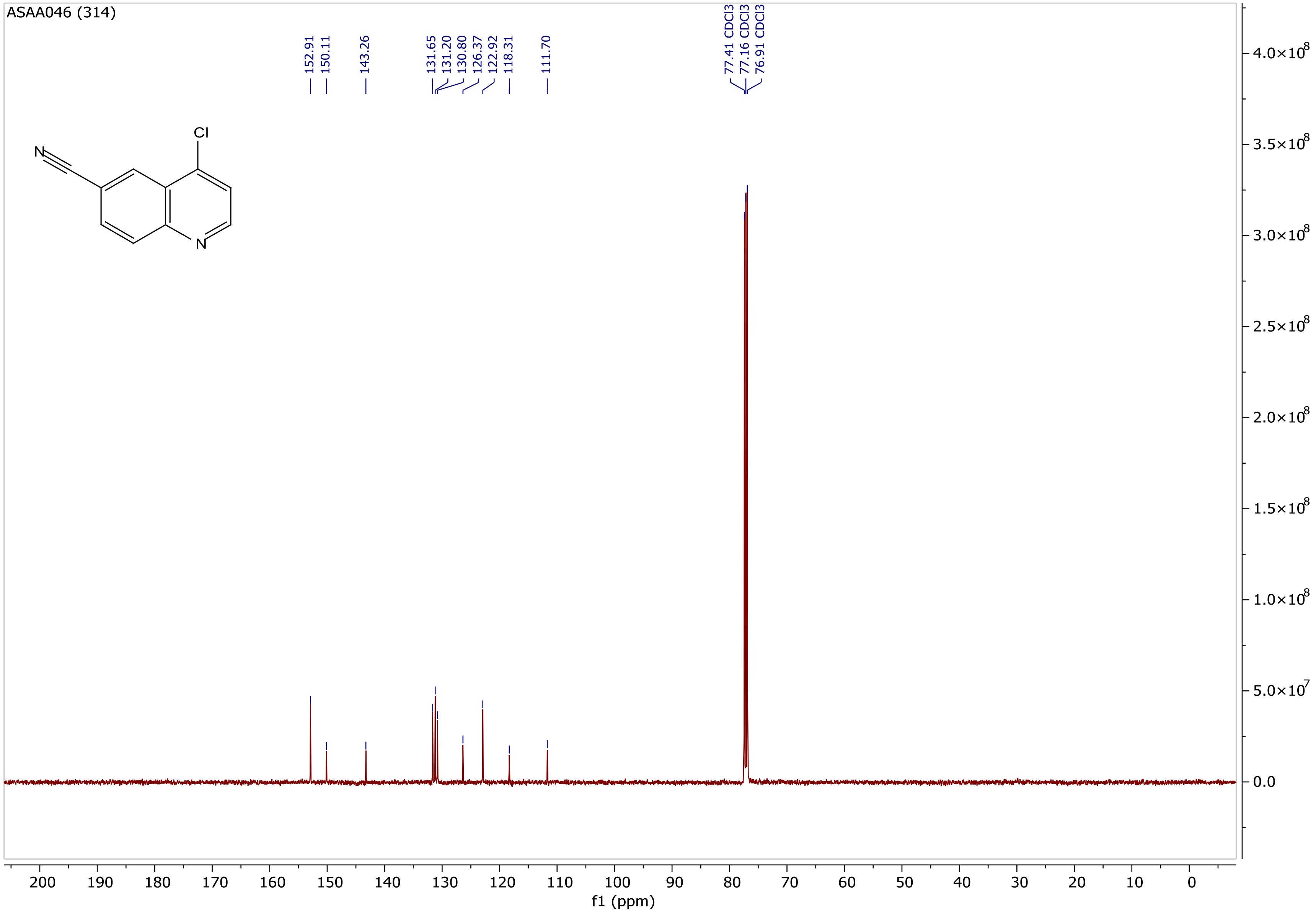

^{1}H NMR spectrum of 4-amino-2-((diethylamino)methyl)phenol (19a)

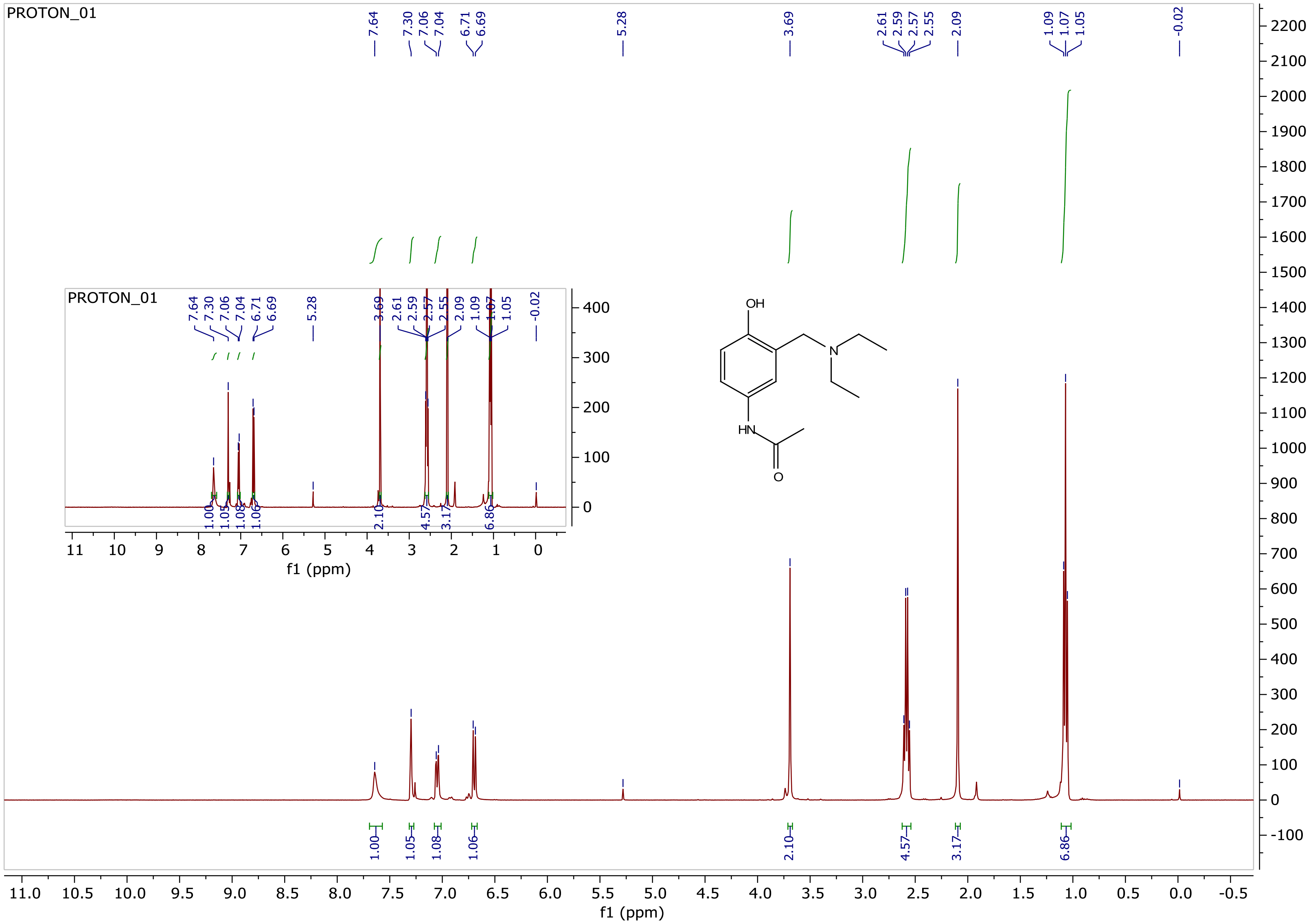

^{13}C NMR spectrum of 4-amino-2-((diethylamino)methyl)phenol (19a)

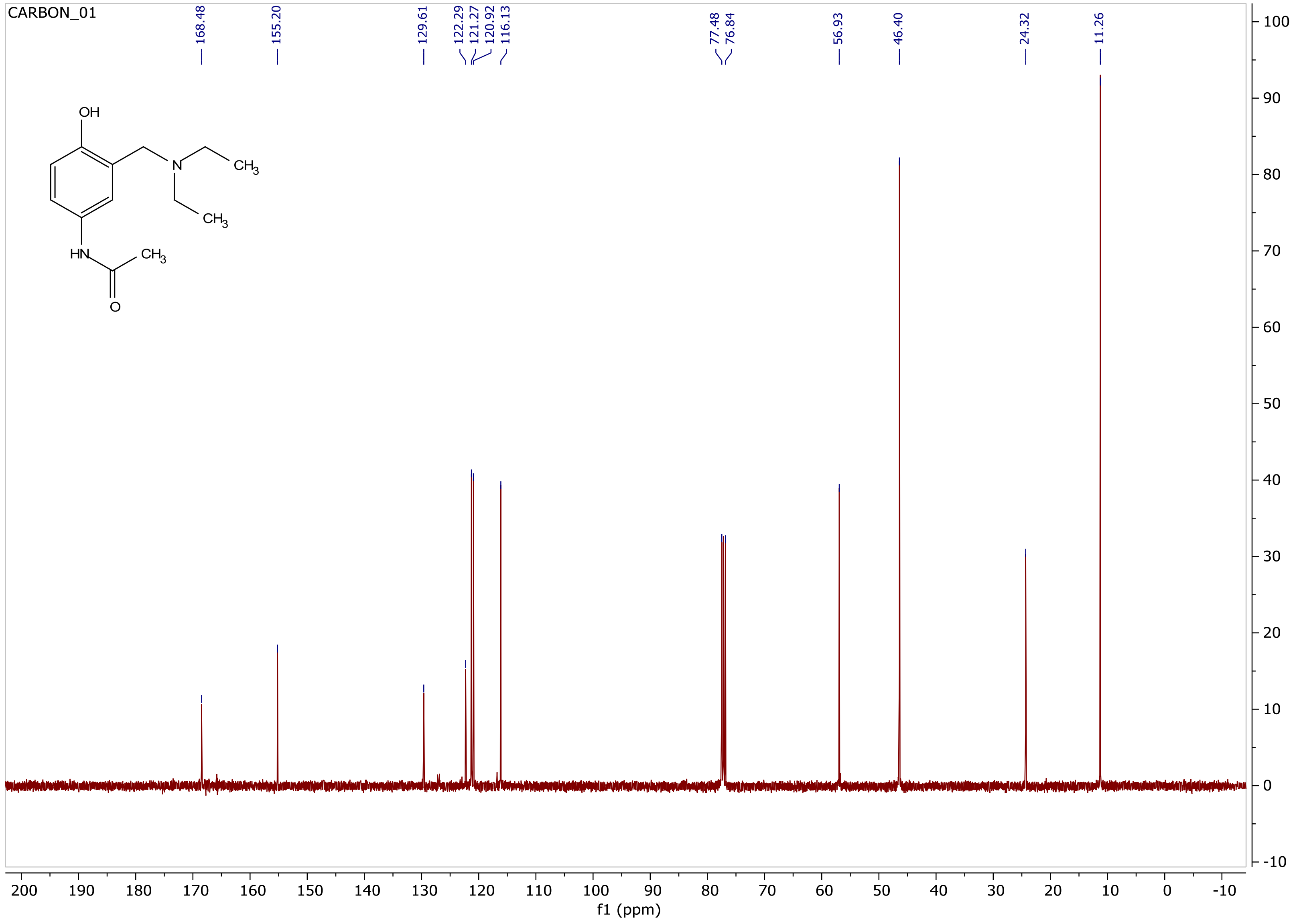

^{1}H NMR spectrum of N-(3-((ethyl(2-hydroxyethyl)amino)methyl)-4-hydroxyphenyl)acetamide (19b)

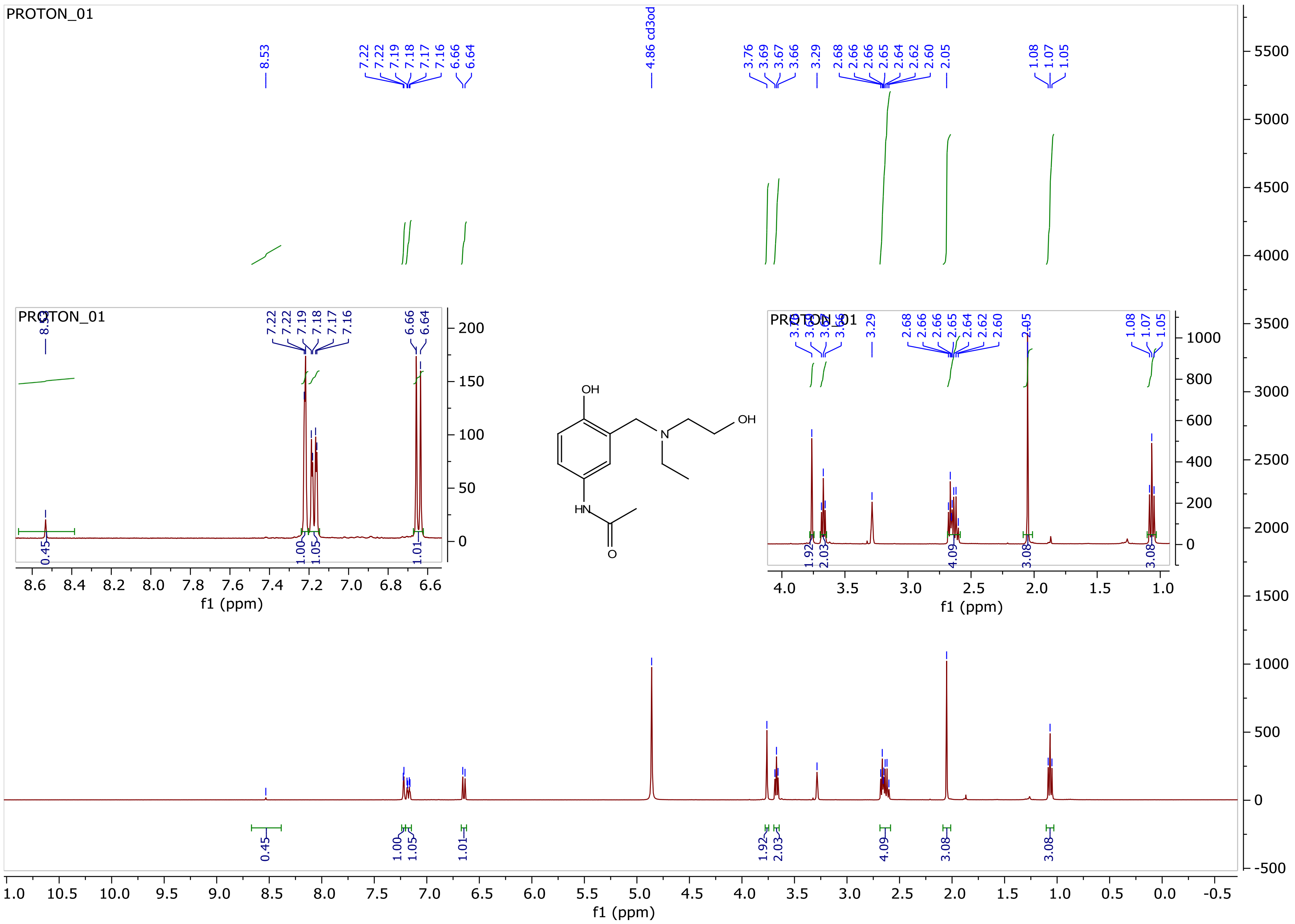

^{13}C NMR spectrum of N-(3-((ethyl(2-hydroxyethyl)amino)methyl)-4-hydroxyphenyl)acetamide (19b)

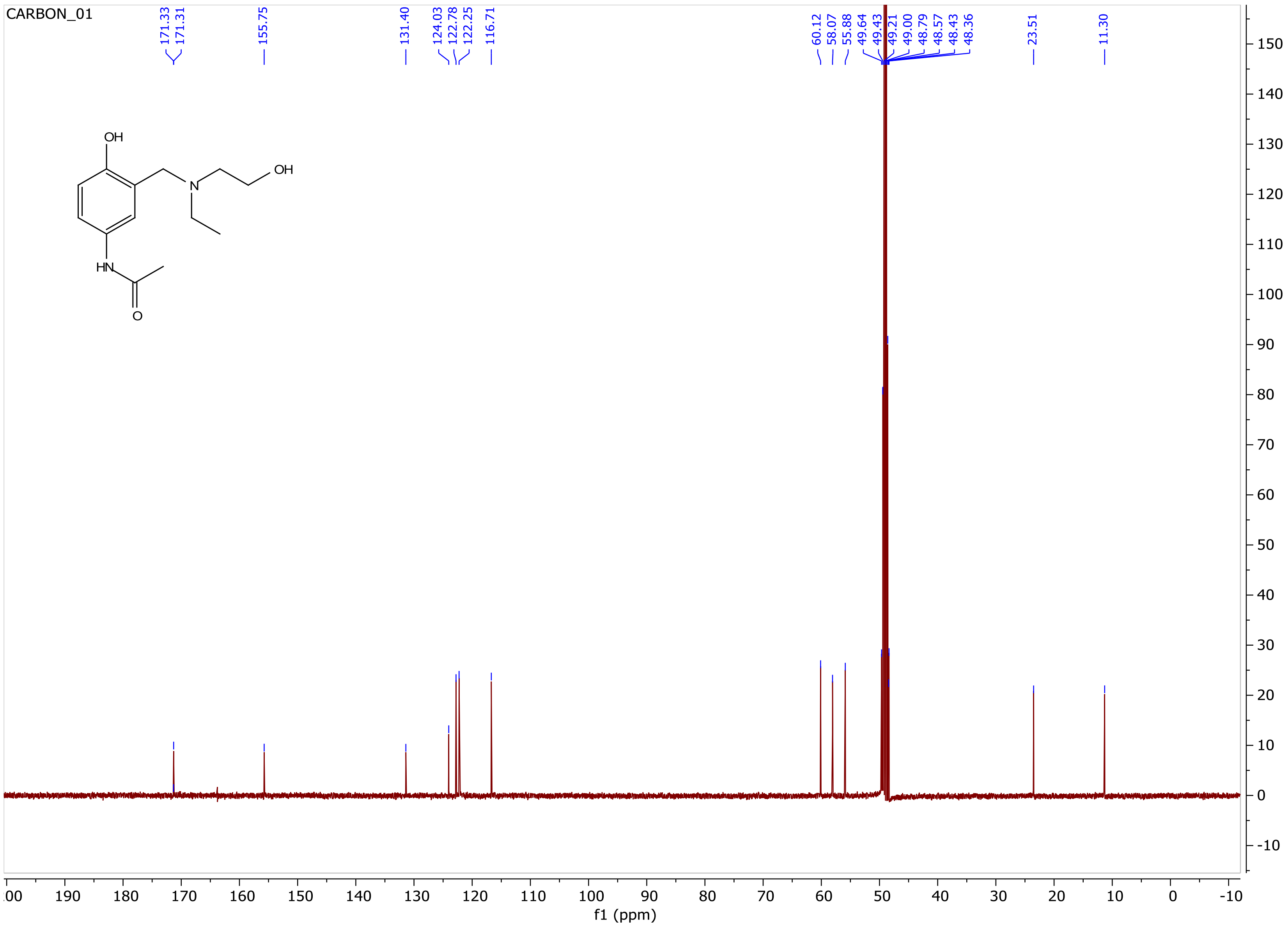

^{1}H NMR spectrum of 4-((7-chloroquinolin-4-yl)amino)-2-((diethylamino)methyl)phenol (AMQ)

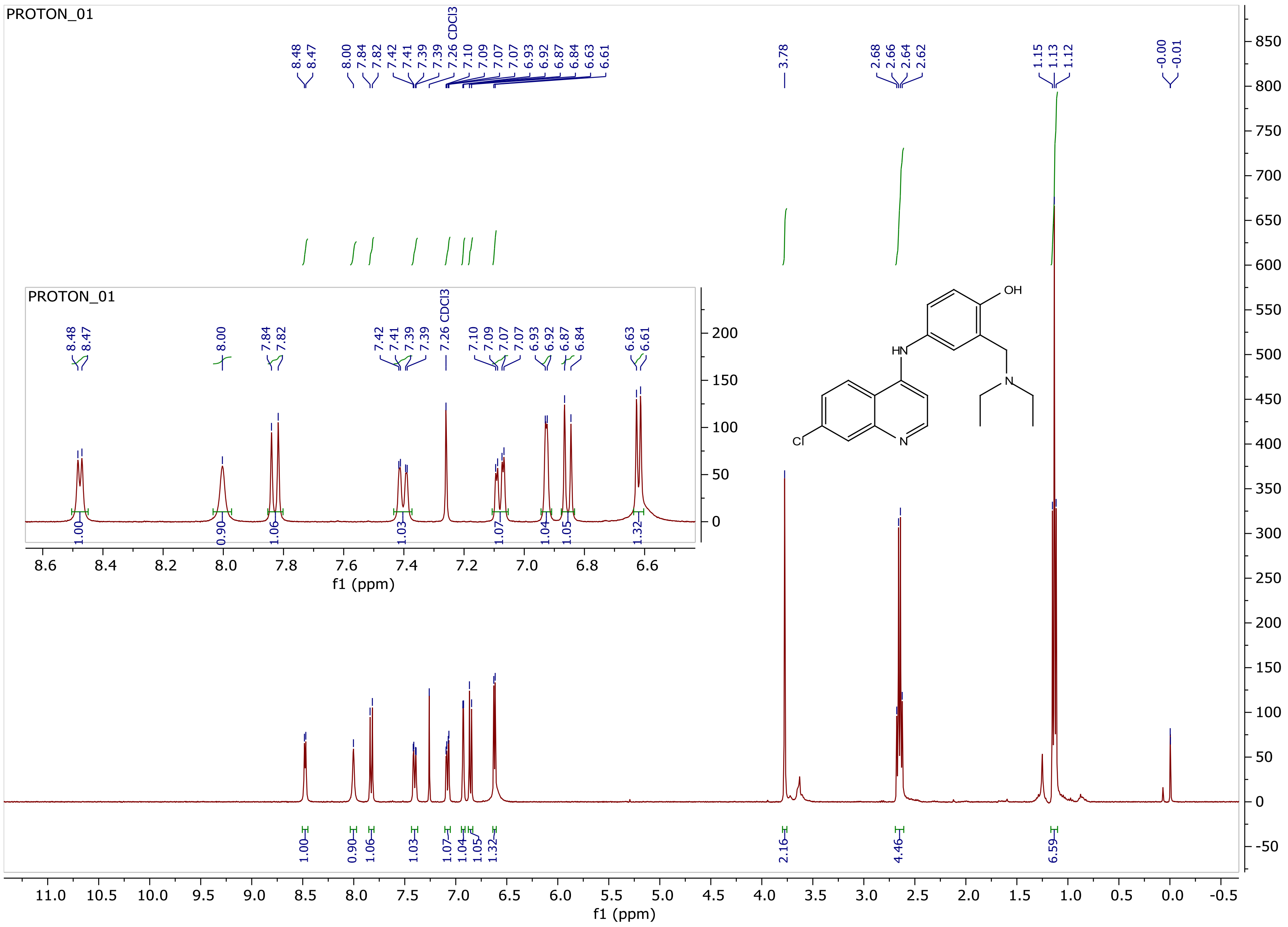

13C NMR spectrum of 4-((7-chloroquinolin-4-yl)amino)-2-((diethylamino)methyl)phenol (AMQ)

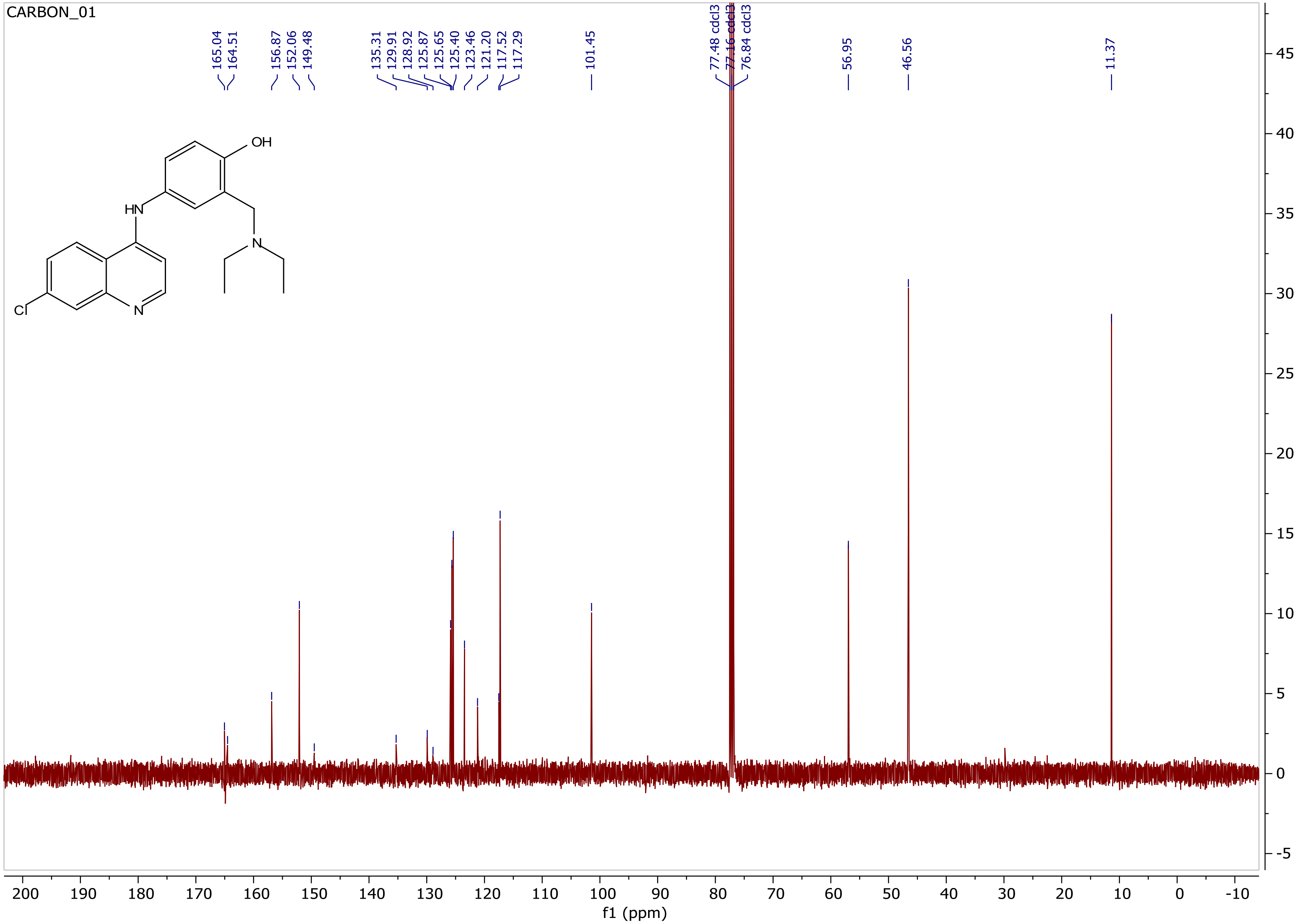

^{1}H NMR spectrum of 2-((diethylamino)methyl)-4-((7-fluoroquinolin-4-yl)amino)phenol (1)

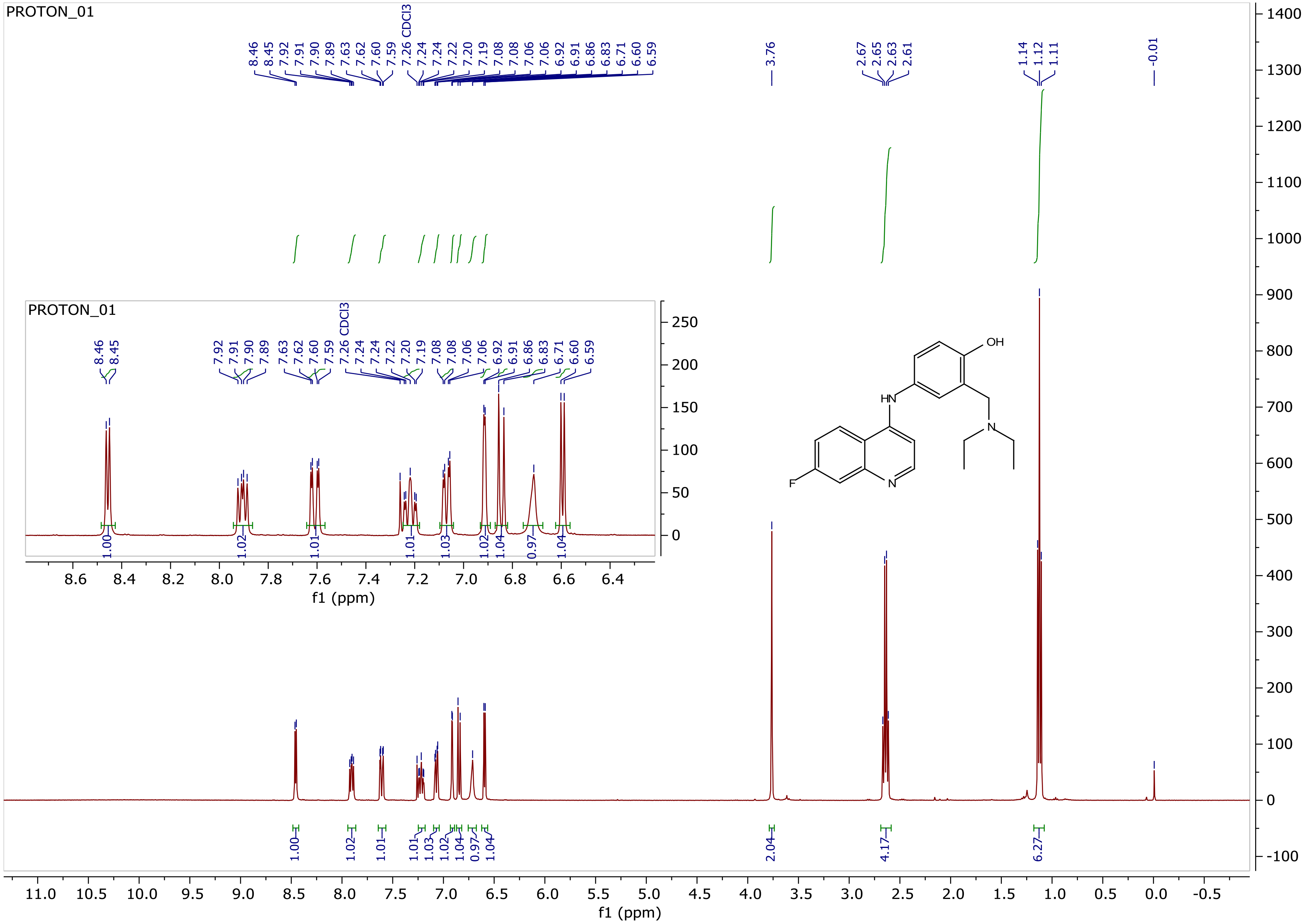

^{13}C NMR spectrum of 2-((diethylamino)methyl)-4-((7-fluoroquinolin-4-yl)amino)phenol (1)

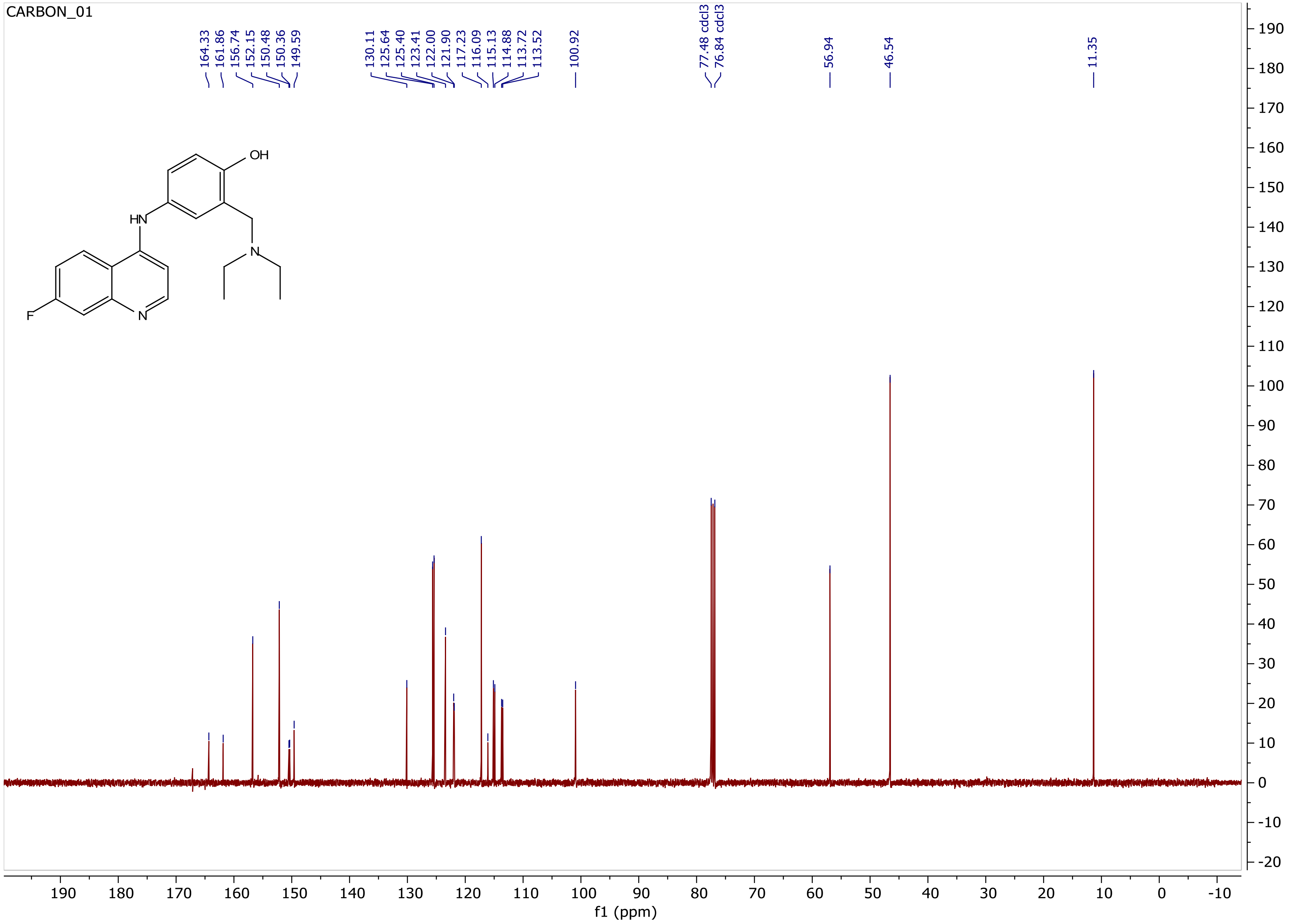

^{1}H NMR spectrum of 2-((diethylamino)methyl)-4-((7-(trifluoromethyl)quinolin-4-yl)amino)phenol (2)

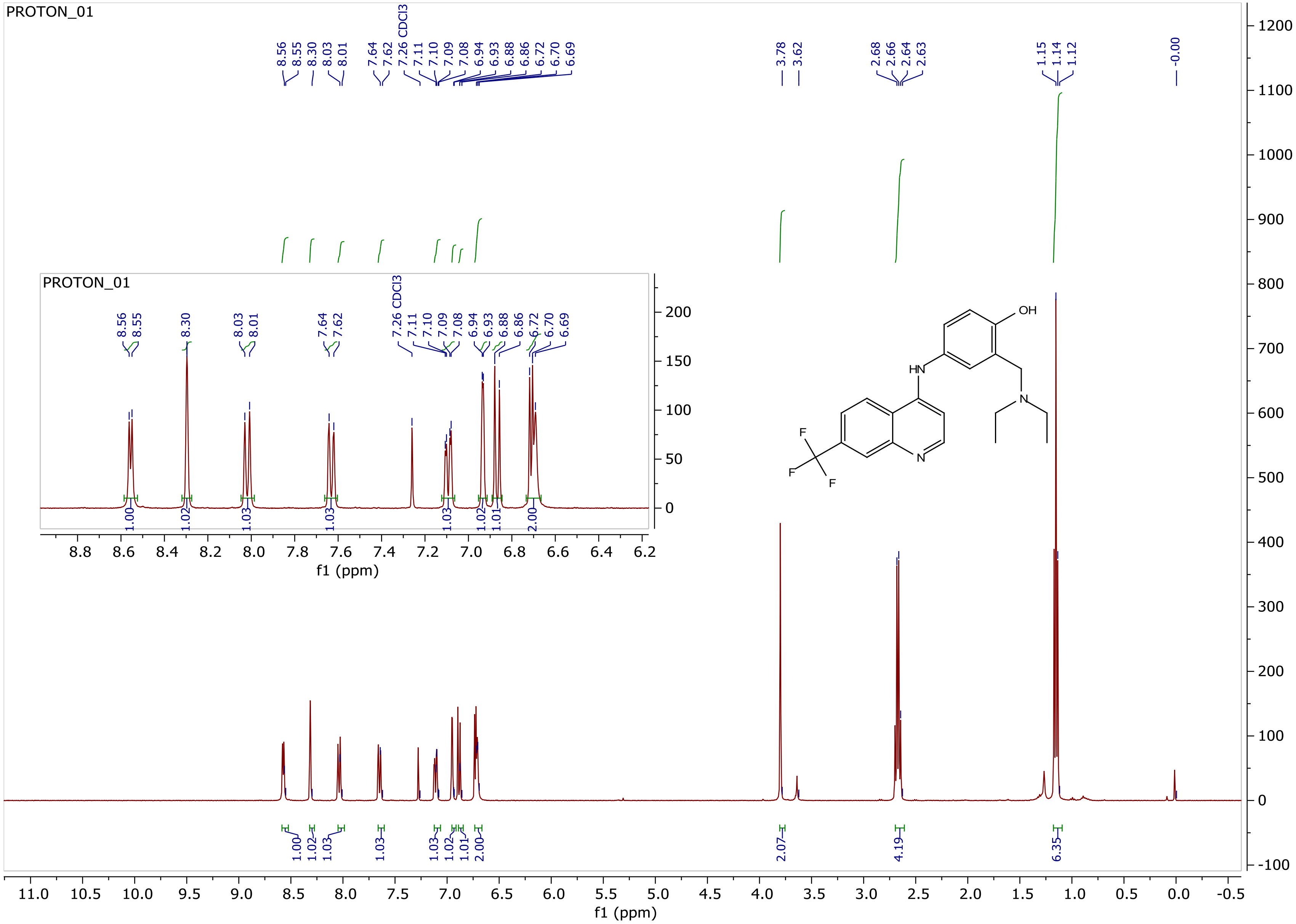

^{13}C NMR spectrum of 2-((diethylamino)methyl)-4-((7-(trifluoromethyl)quinolin-4-yl)amino)phenol (2)

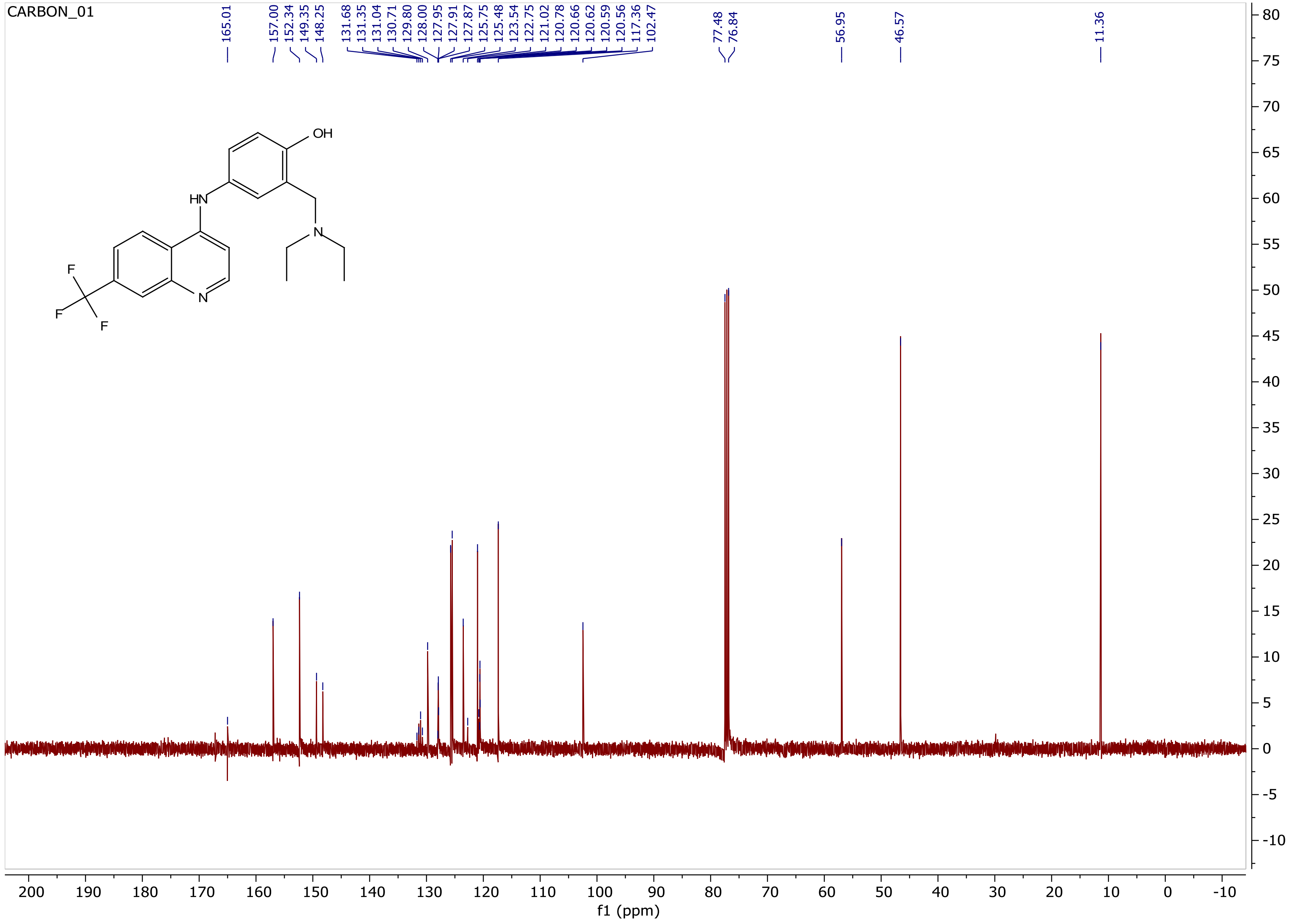

^{1}H NMR spectrum of 4-((3-((diethylamino)methyl)-4-hydroxyphenyl)amino)quinoline-7-carbonitrile (3)

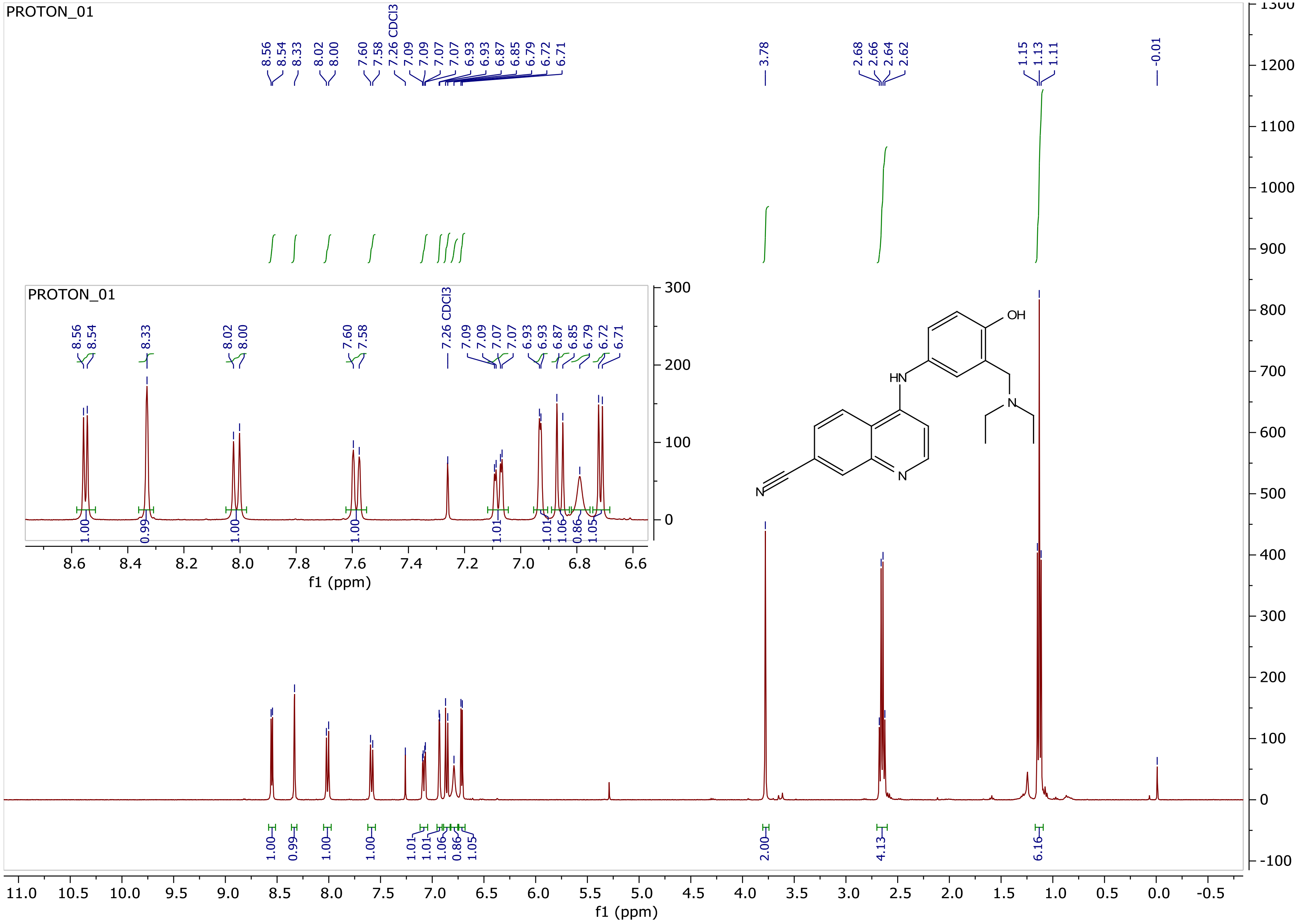

^{13}C NMR spectrum of 4-((3-((diethylamino)methyl)-4-hydroxyphenyl)amino)quinoline-7-carbonitrile (3)

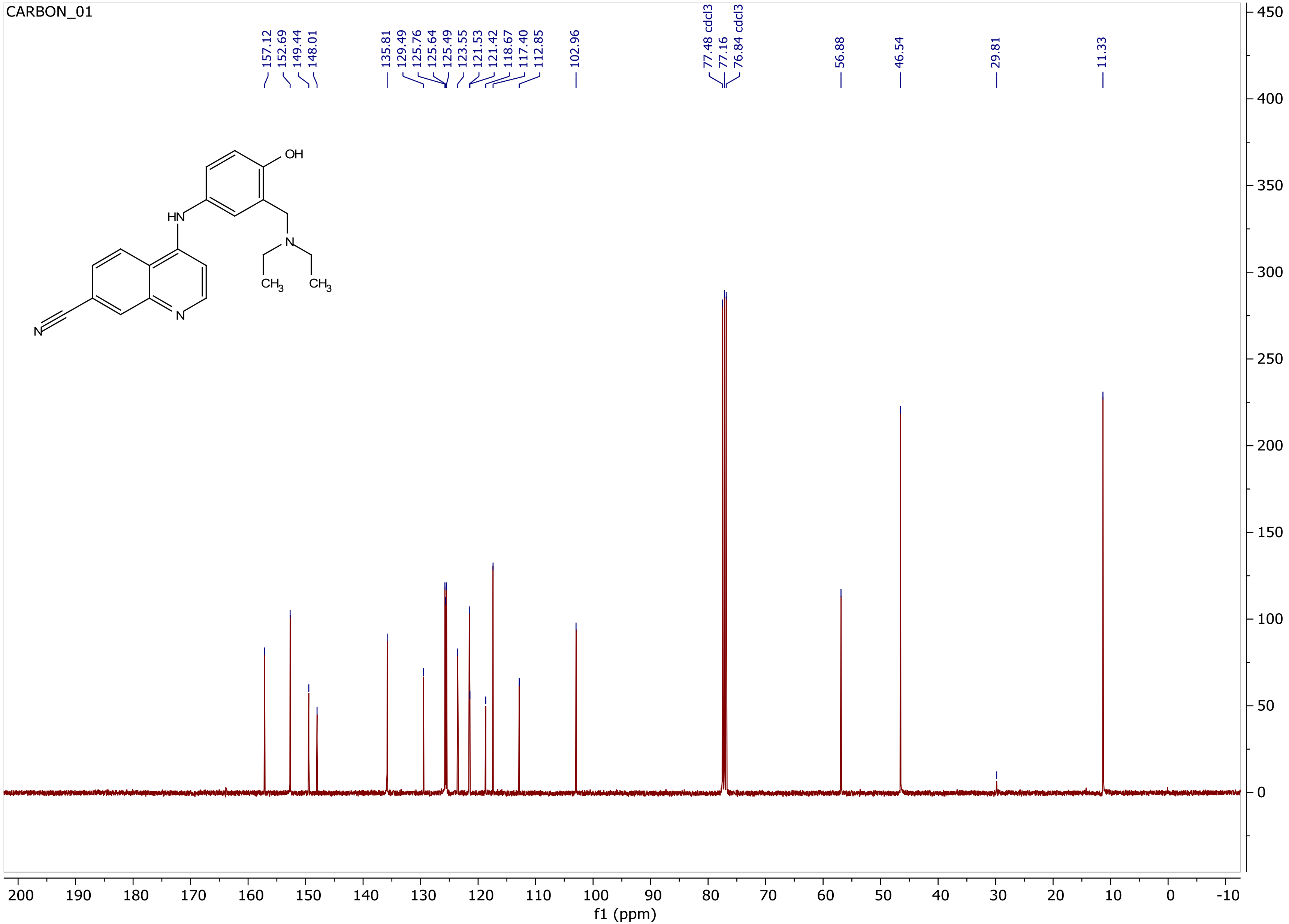

^{1}H NMR spectrum of 2-((diethylamino)methyl)-4-((7-nitroquinolin-4-yl)amino)phenol (4)

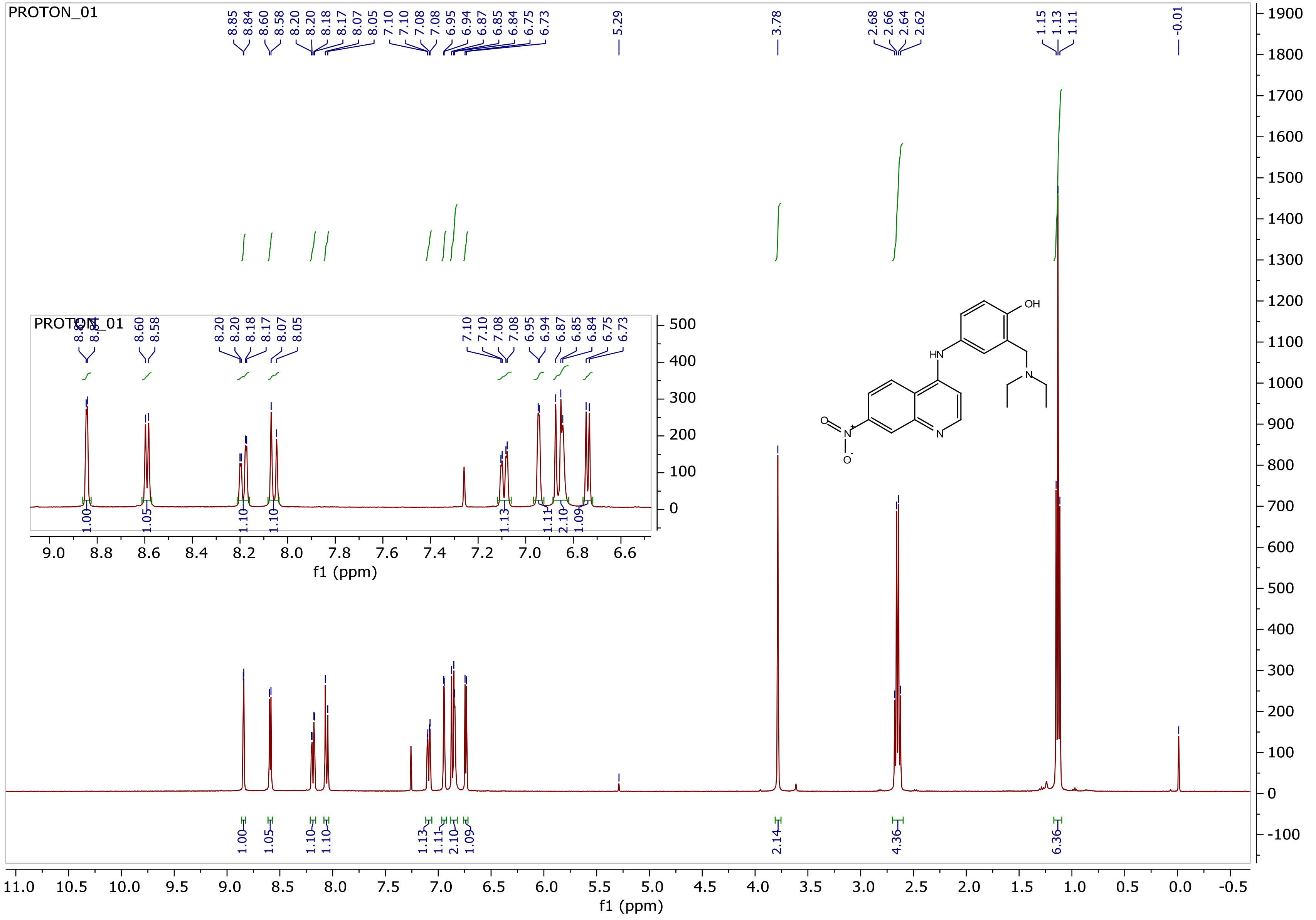

^{13}C NMR spectrum of 2-((diethylamino)methyl)-4-((7-nitroquinolin-4-yl)amino)phenol (4)

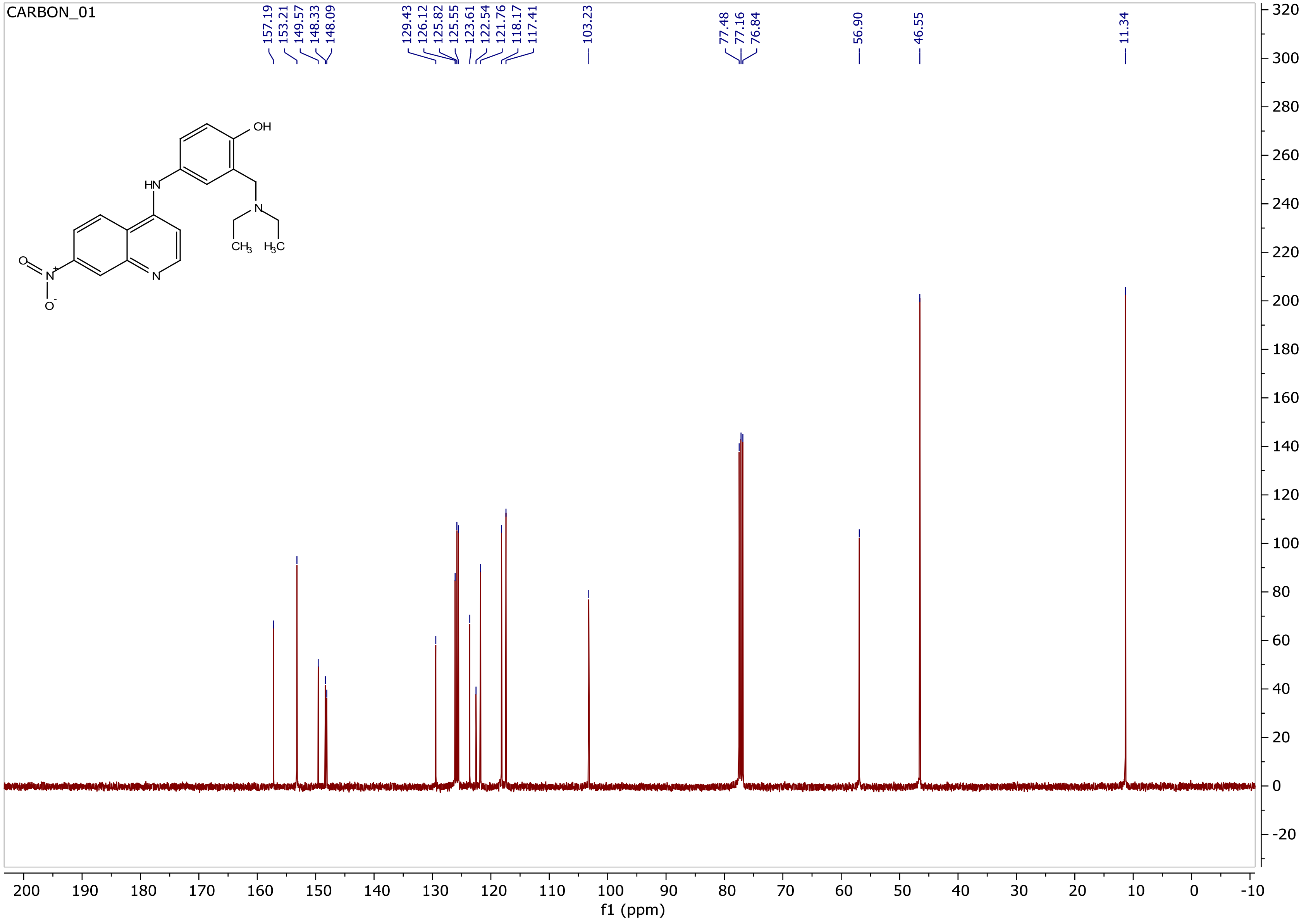

^{1}H NMR spectrum of 2-((diethylamino)methyl)-4-((7-methoxyquinolin-4-yl)amino)phenol (5)

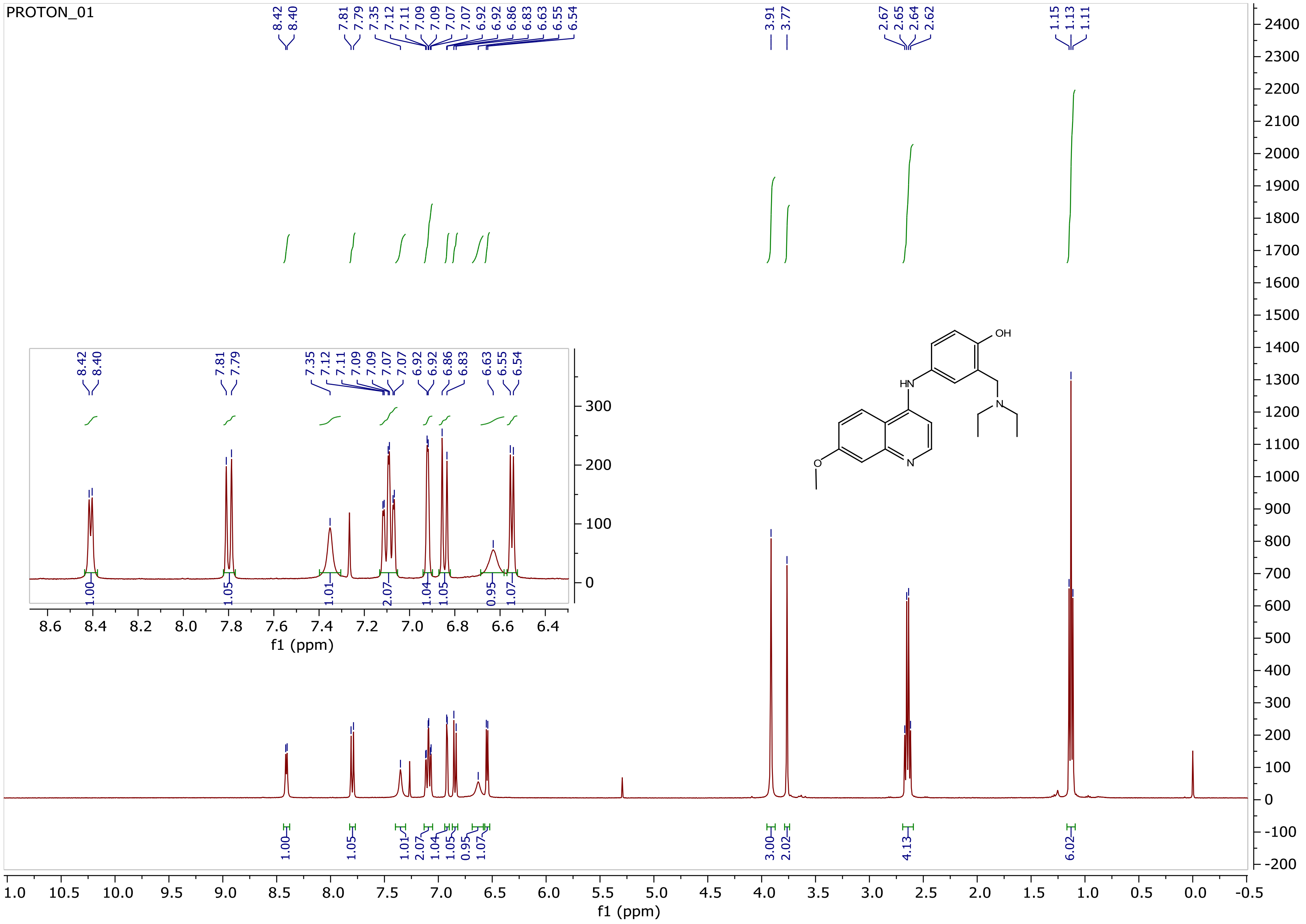

^{13}C NMR spectrum of 2-((diethylamino)methyl)-4-((7-methoxyquinolin-4-yl)amino)phenol (5)

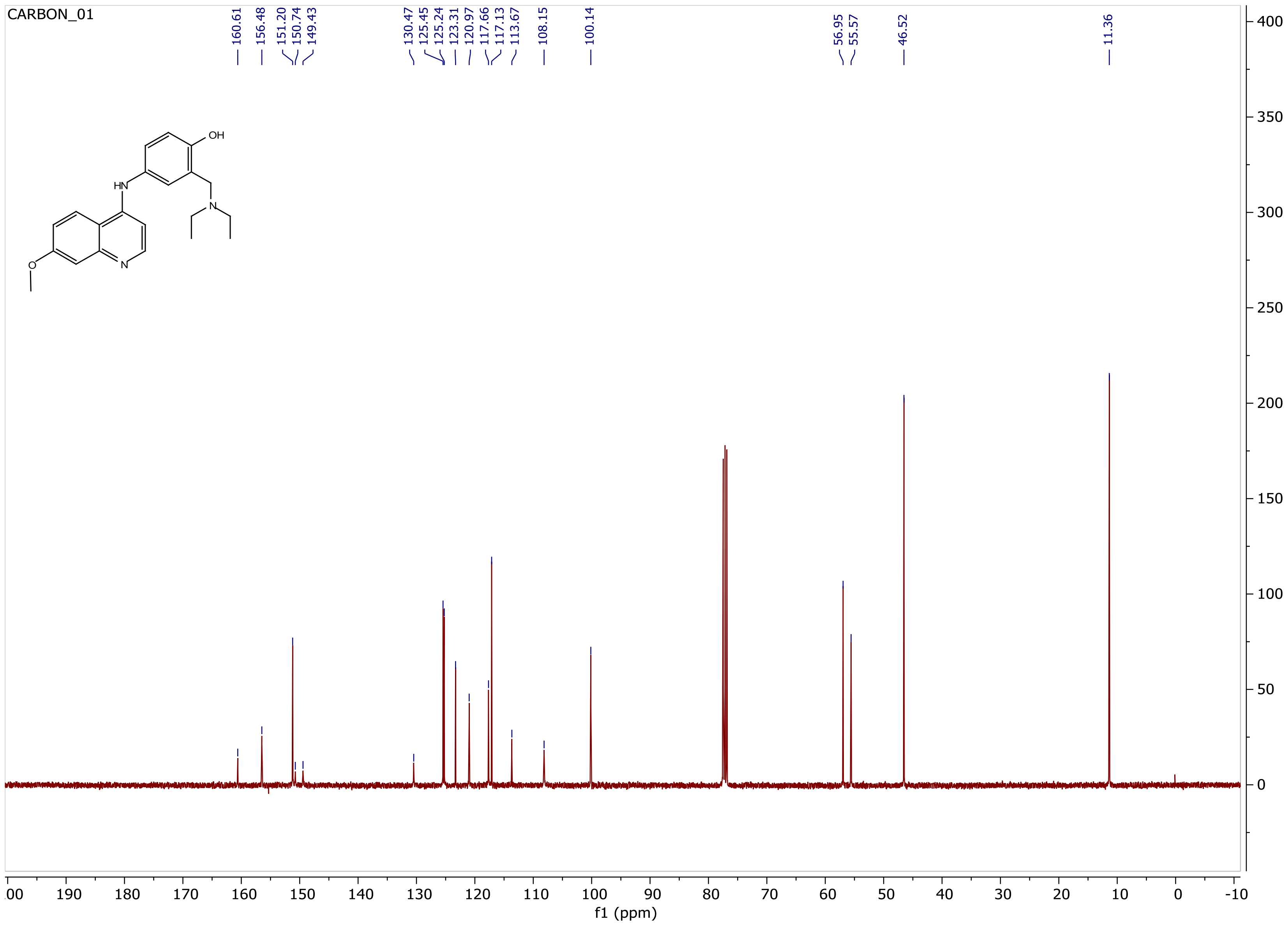

^{1}H NMR spectrum of 2-((diethylamino)methyl)-4-(quinolin-4-ylamino)phenol (6)

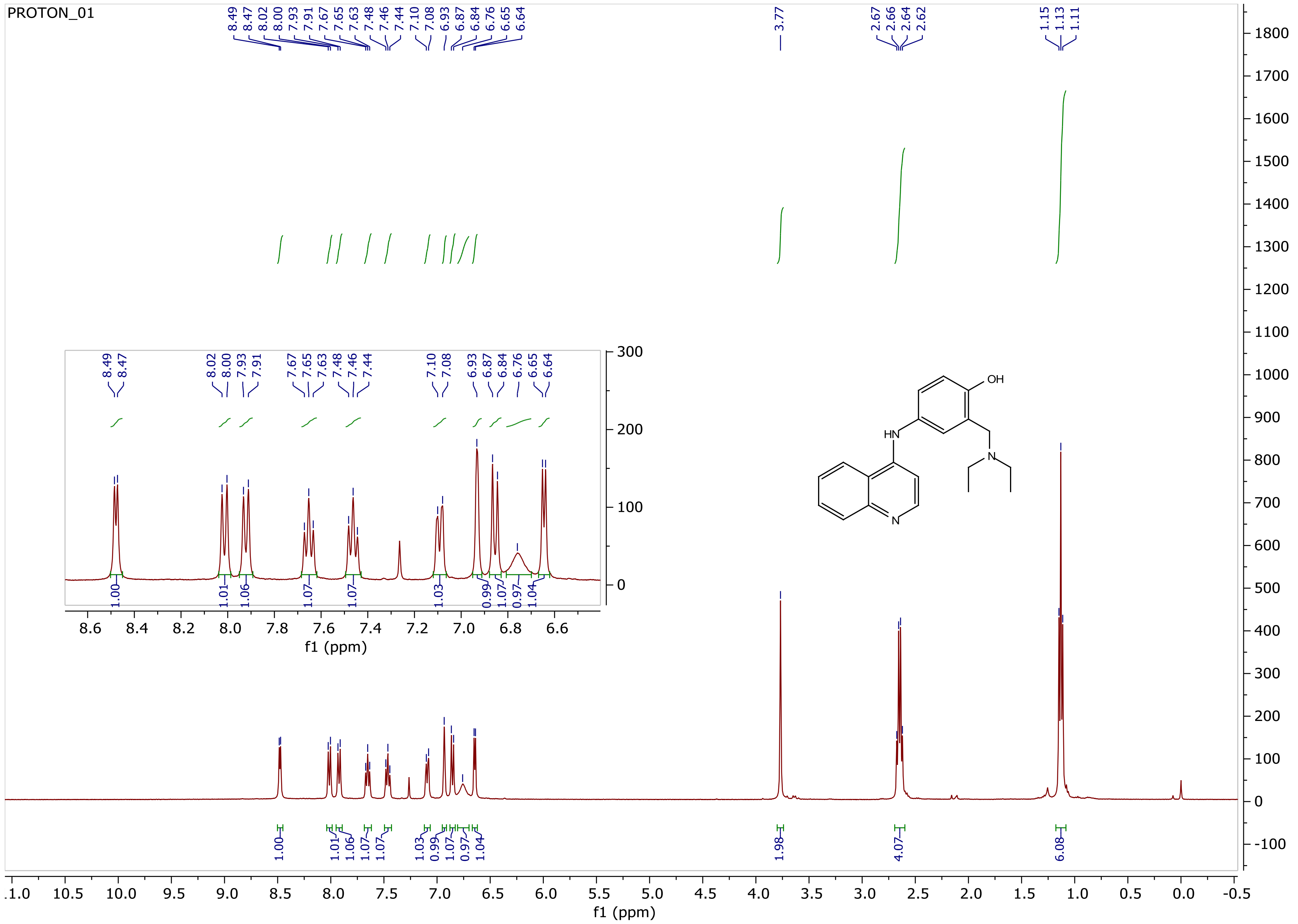

^{13}C NMR spectrum of 2-((diethylamino)methyl)-4-(quinolin-4-ylamino)phenol (6)

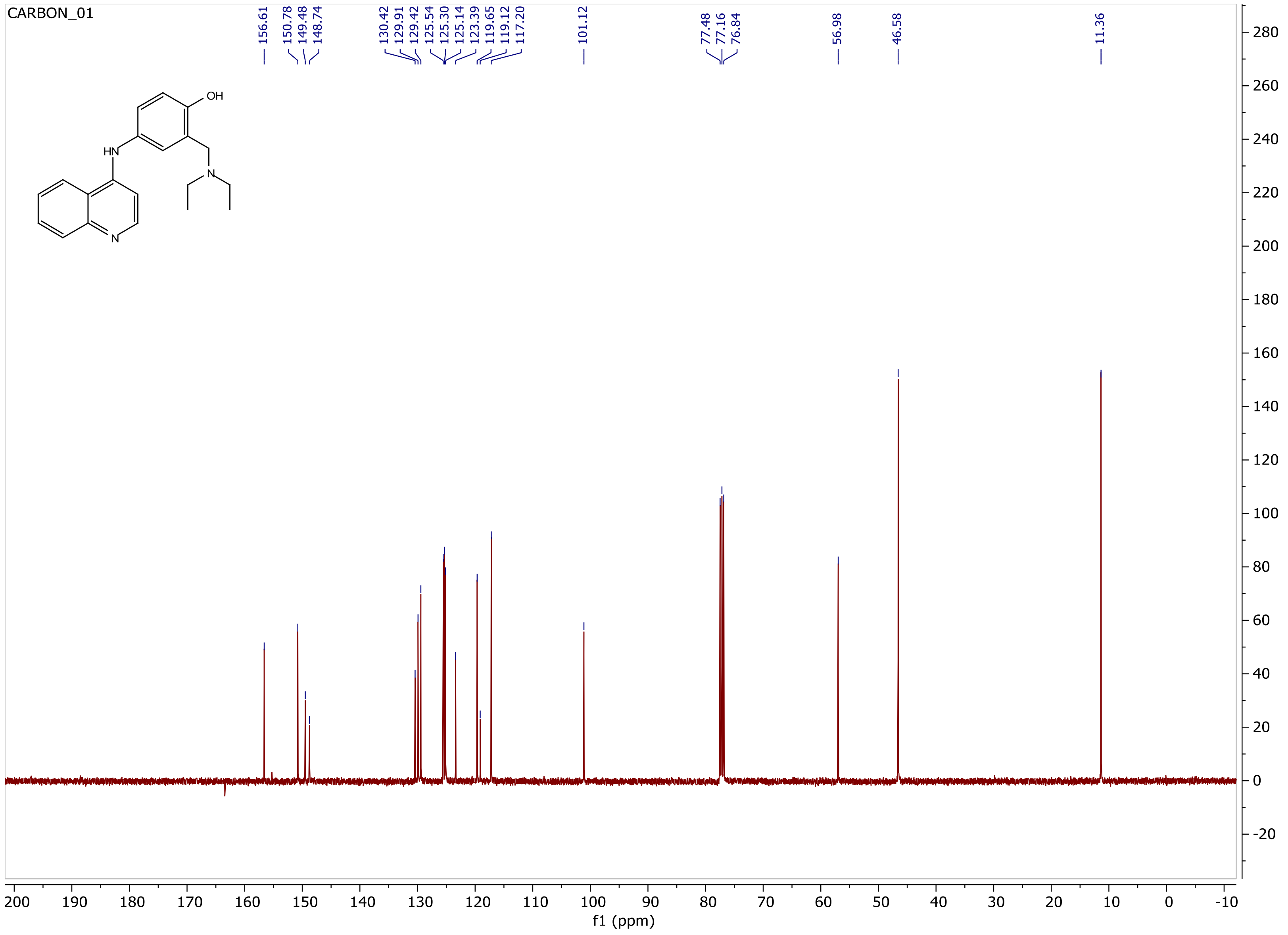

^{1}H NMR spectrum of 2-((diethylamino)methyl)-4-((6-fluoroquinolin-4-yl)amino)phenol (7)

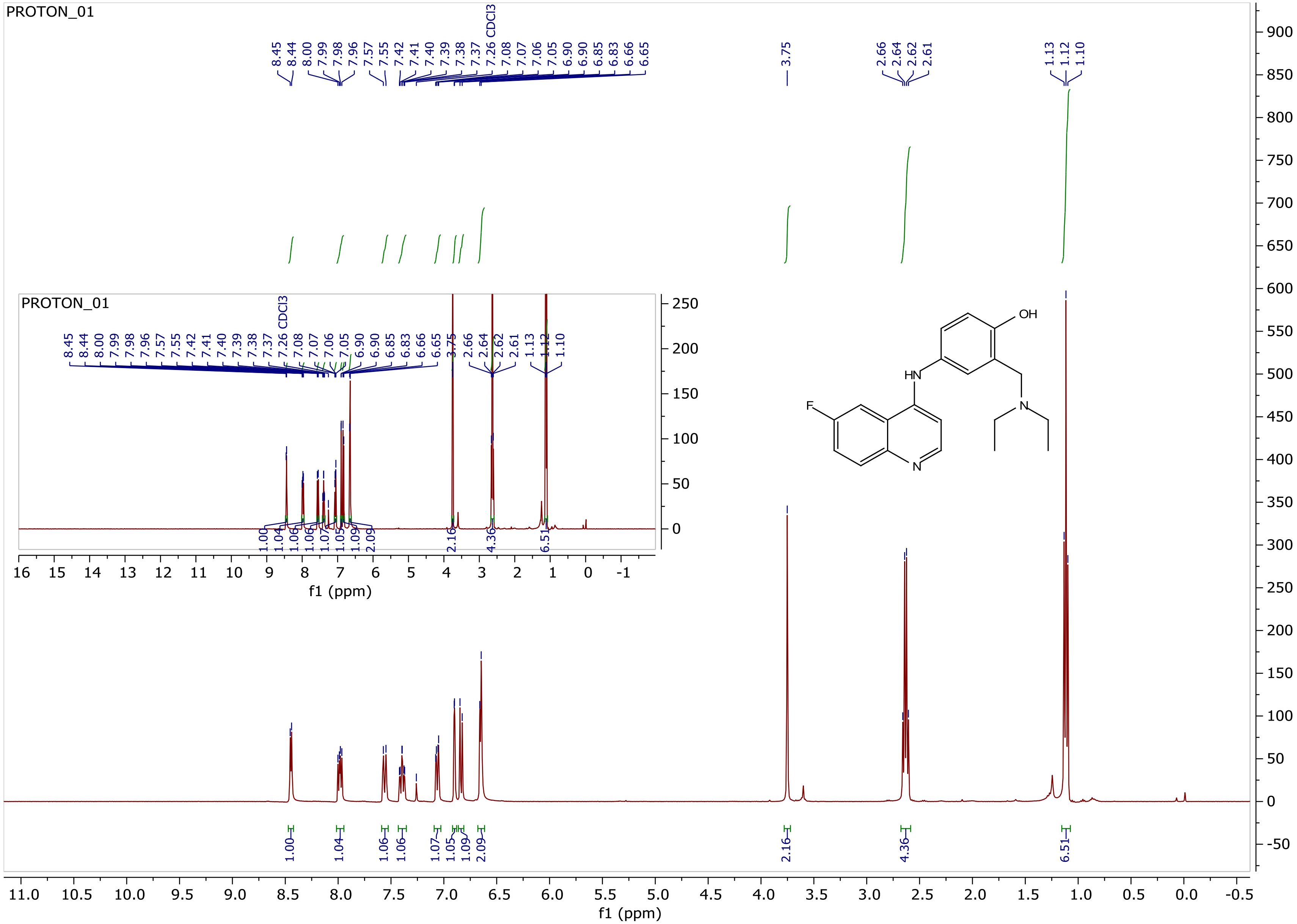

^{13}C NMR spectrum of 2-((diethylamino)methyl)-4-((6-fluoroquinolin-4-yl)amino)phenol (7)

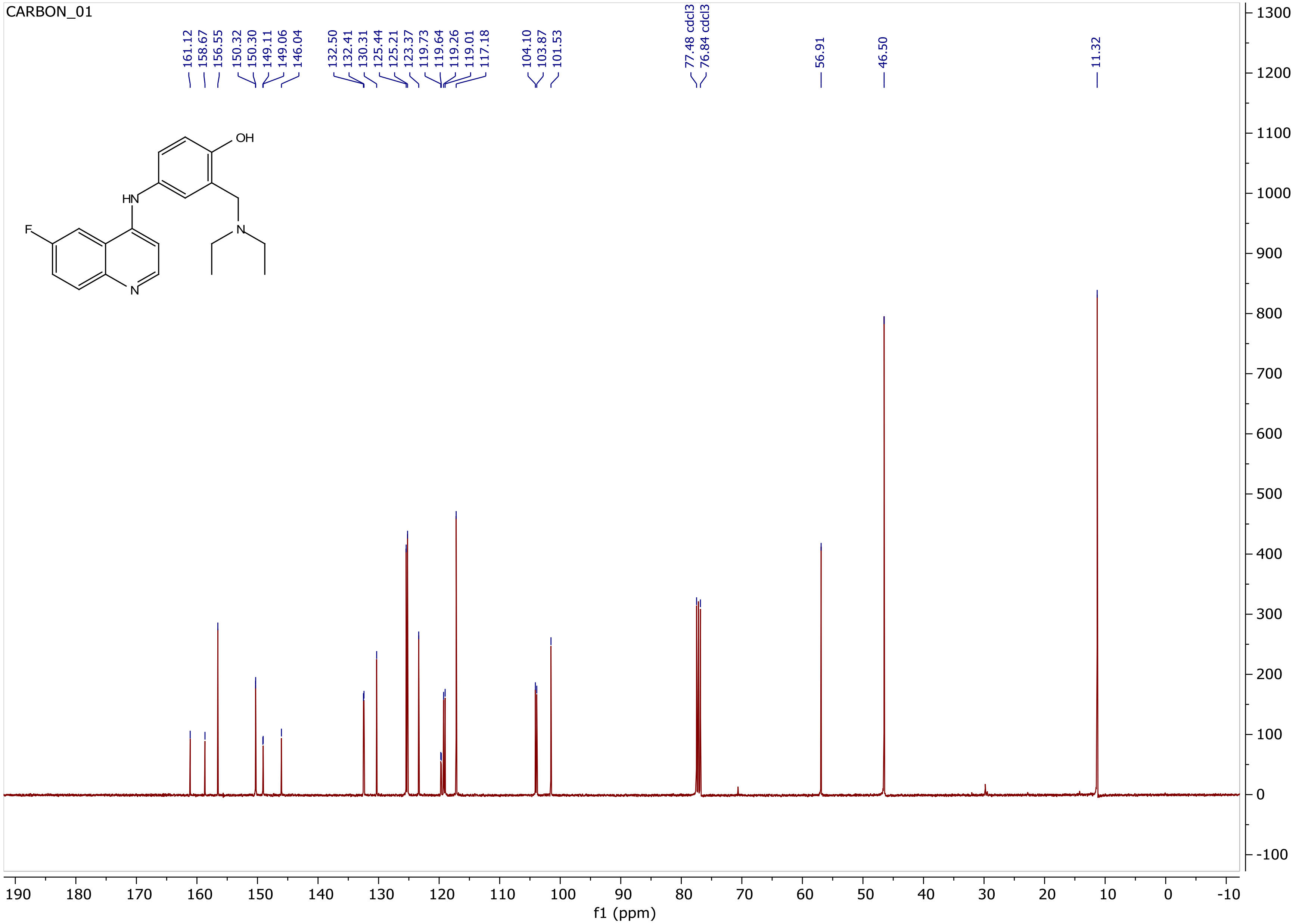

^{1}H NMR spectrum of 2-((diethylamino)methyl)-4-((8-fluoroquinolin-4-yl)amino)phenol (8)

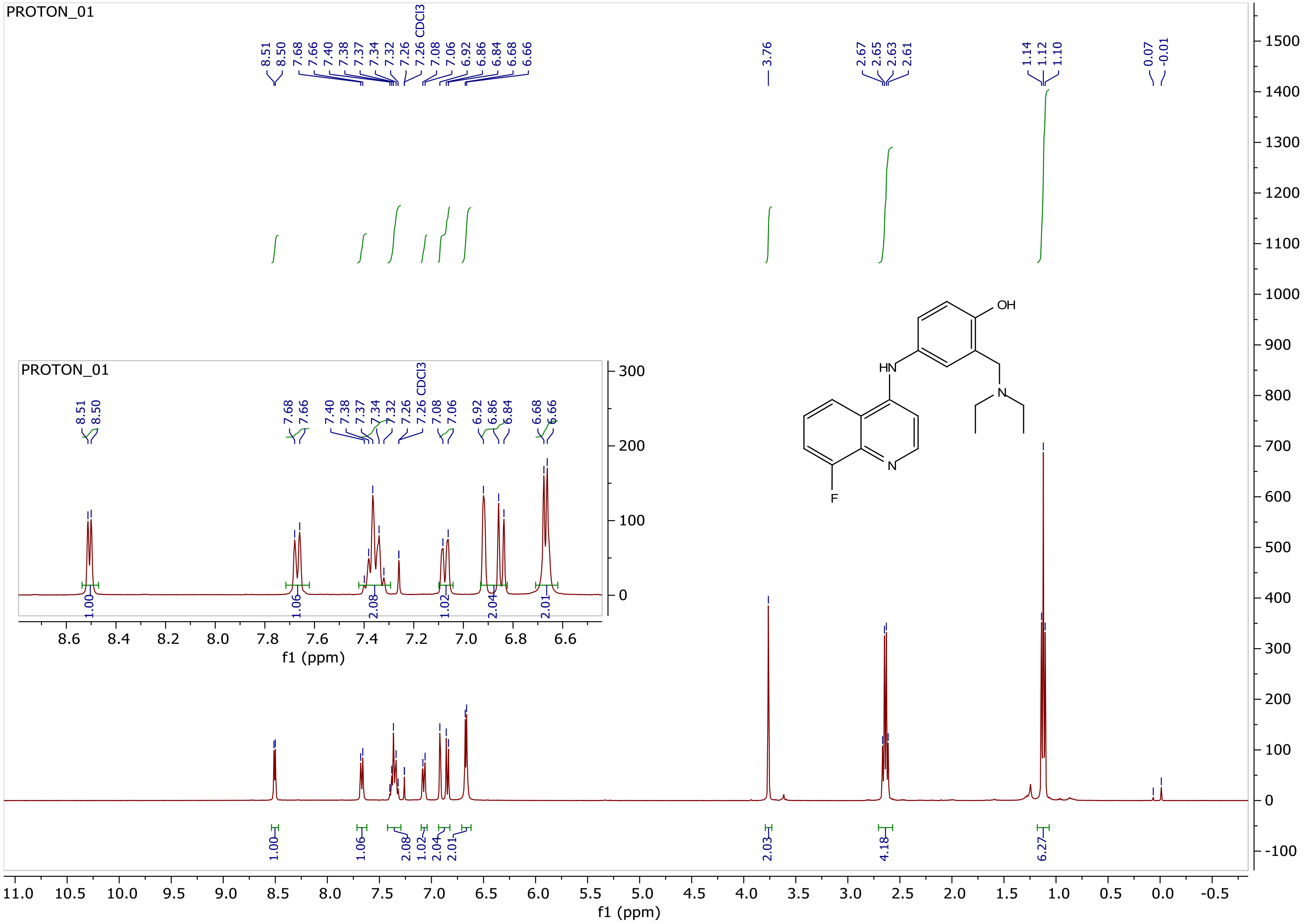

^{13}C NMR spectrum of 2-((diethylamino)methyl)-4-((8-fluoroquinolin-4-yl)amino)phenol (8)

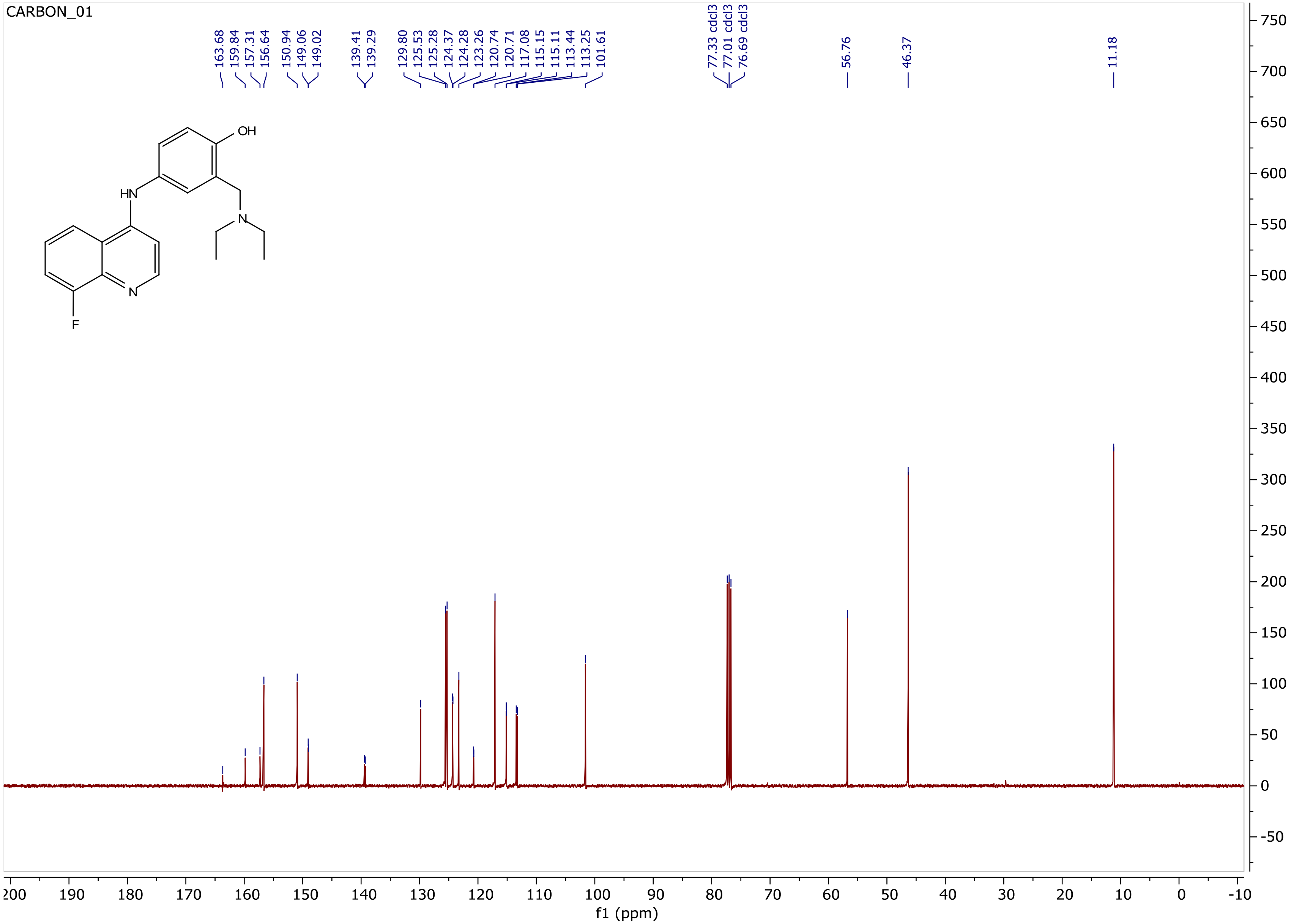

^{1}H NMR spectrum of 2-((diethylamino)methyl)-4-((6-(trifluoromethyl)quinolin-4-yl)amino)phenol (9)

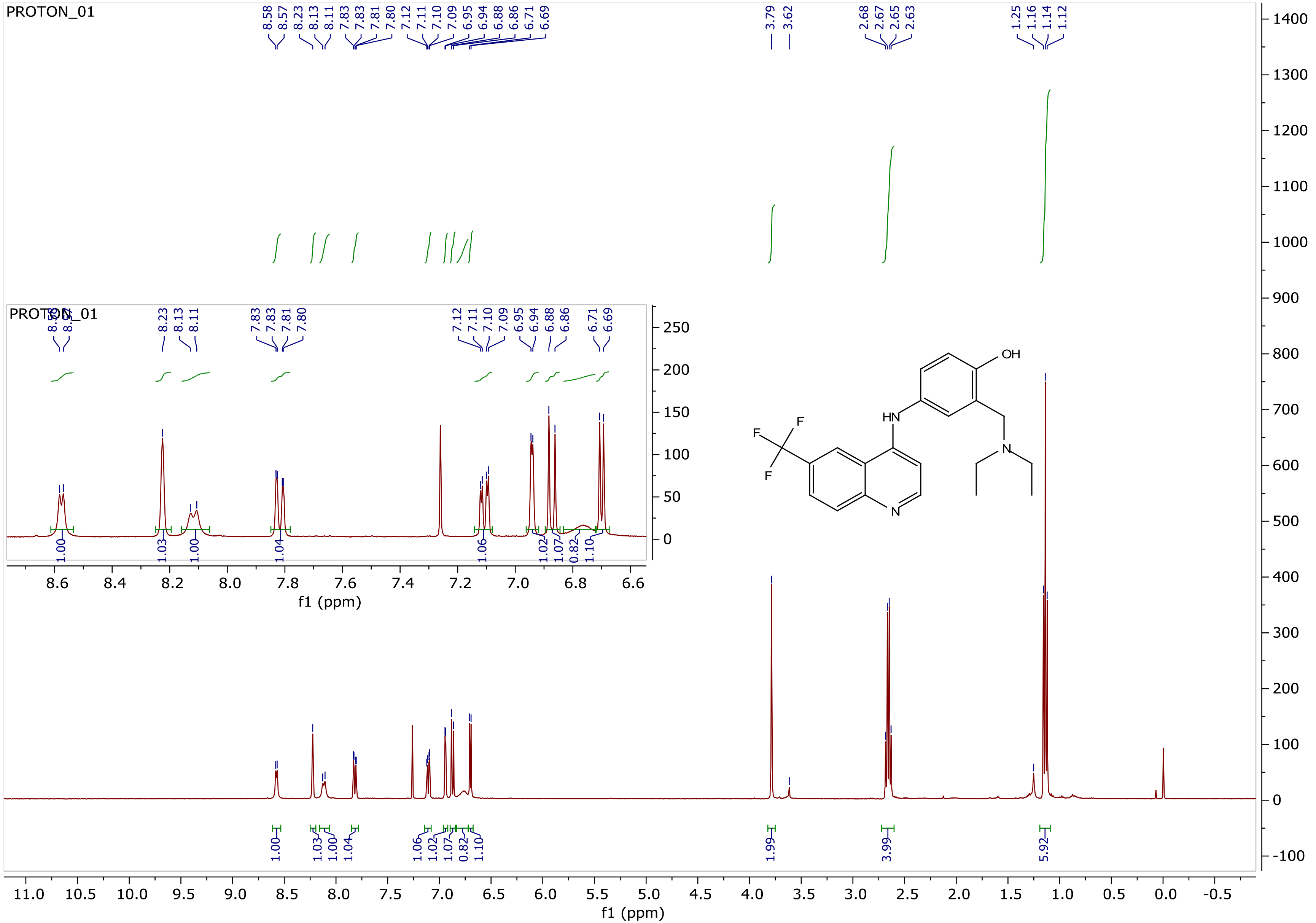

^{13}C NMR spectrum of 2-((diethylamino)methyl)-4-((6-(trifluoromethyl)quinolin-4-yl)amino)phenol (9)

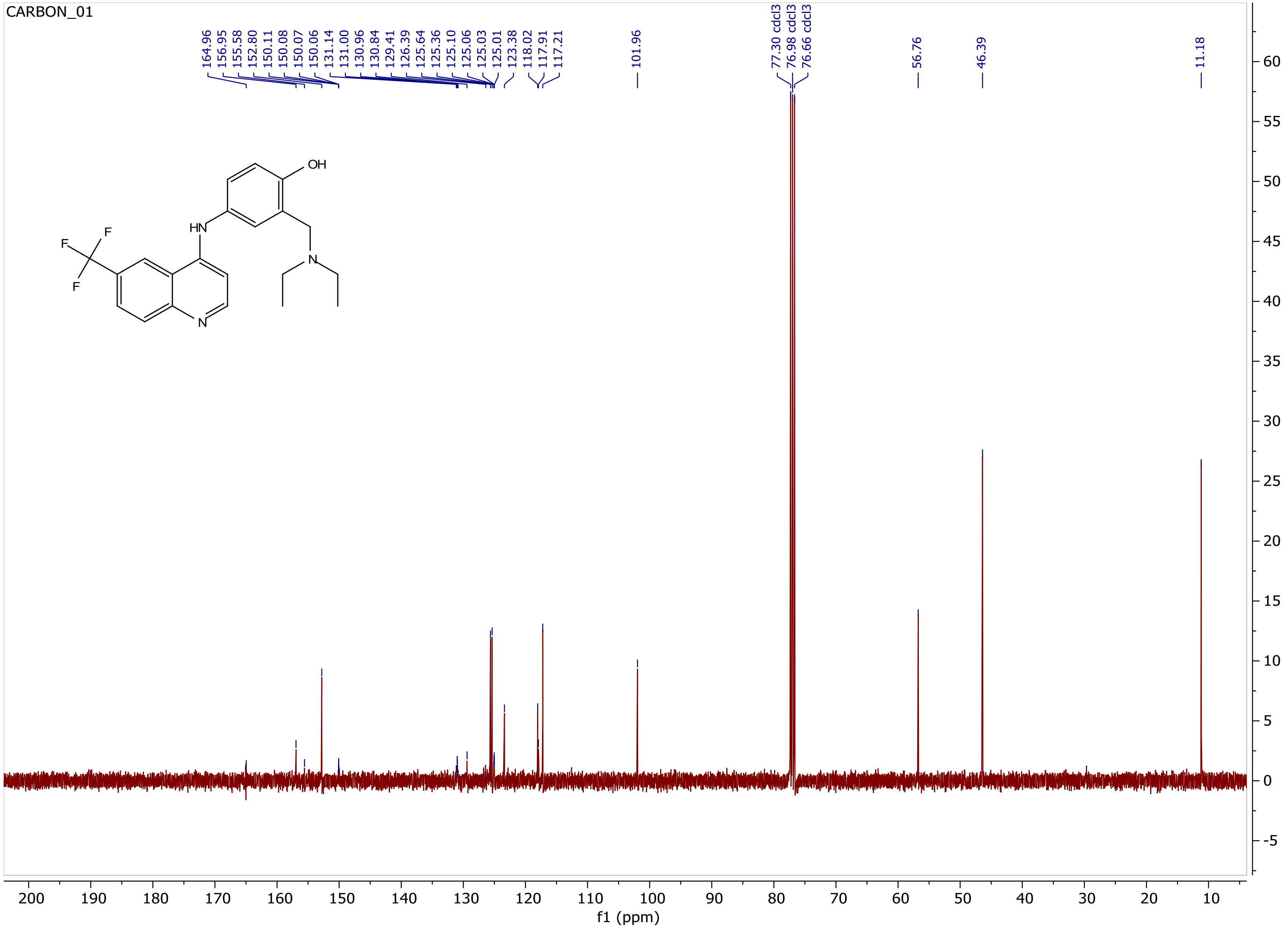

^{1}H NMR spectrum of 2-((diethylamino)methyl)-4-((8-(trifluoromethyl)quinolin-4-yl)amino)phenol (10)

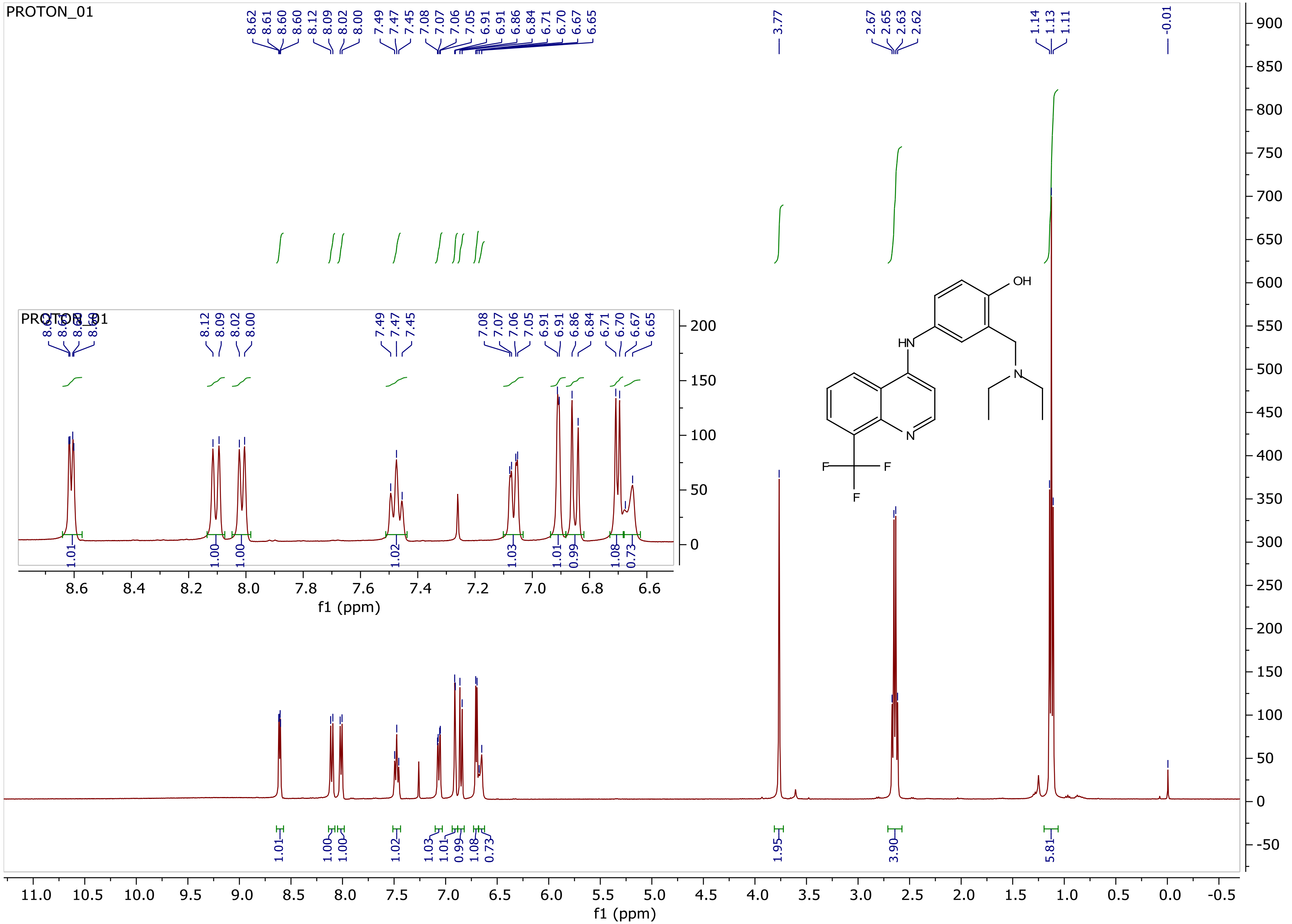

^{13}C NMR spectrum of 2-((diethylamino)methyl)-4-((8-(trifluoromethyl)quinolin-4-yl)amino)phenol (10)

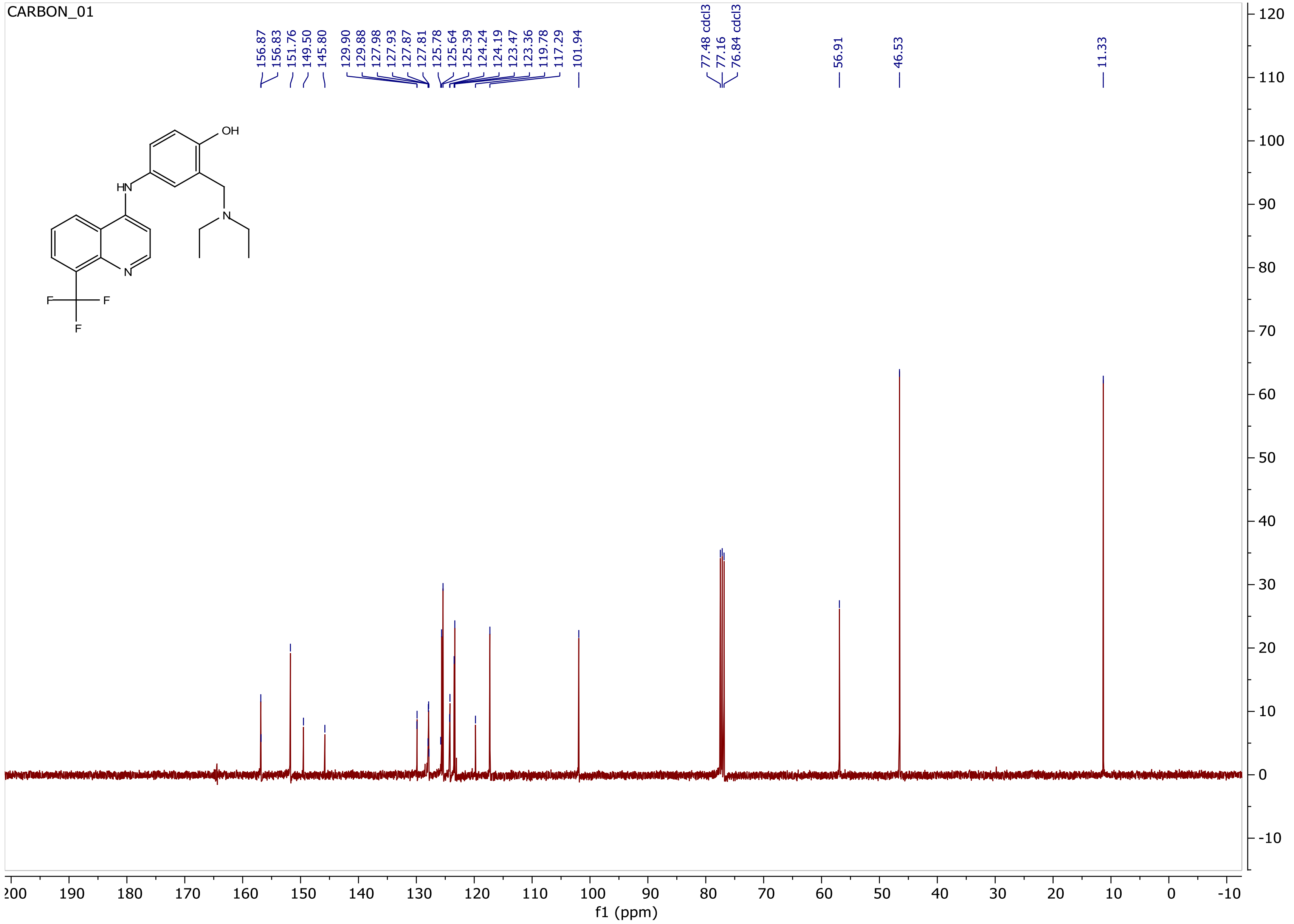

^{1}H NMR spectrum of 2-((diethylamino)methyl)-4-((2-(trifluoromethyl)quinolin-4-yl)amino)phenol (11)

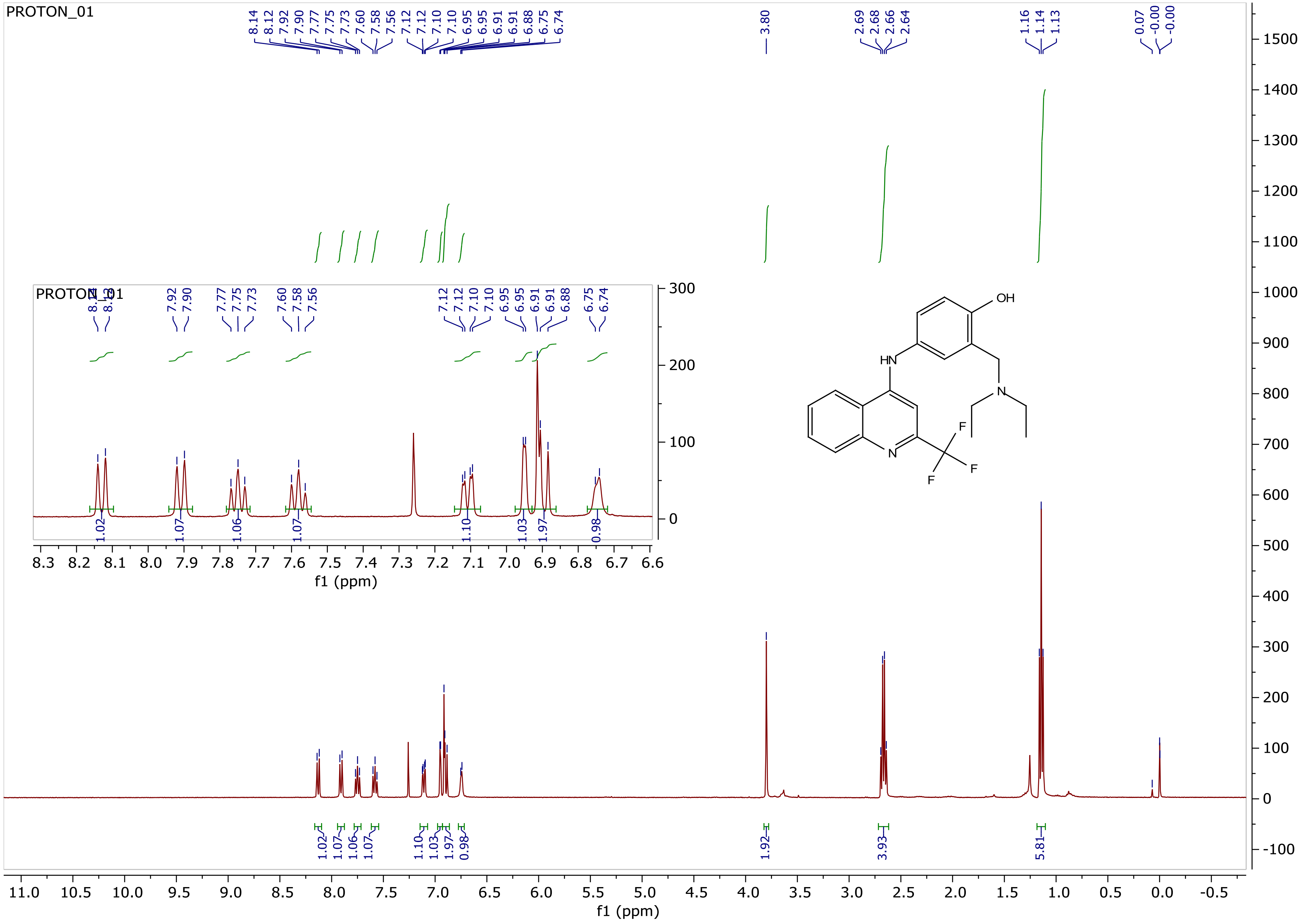

^{13}C NMR spectrum of 2-((diethylamino)methyl)-4-((2-(trifluoromethyl)quinolin-4-yl)amino)phenol (11)

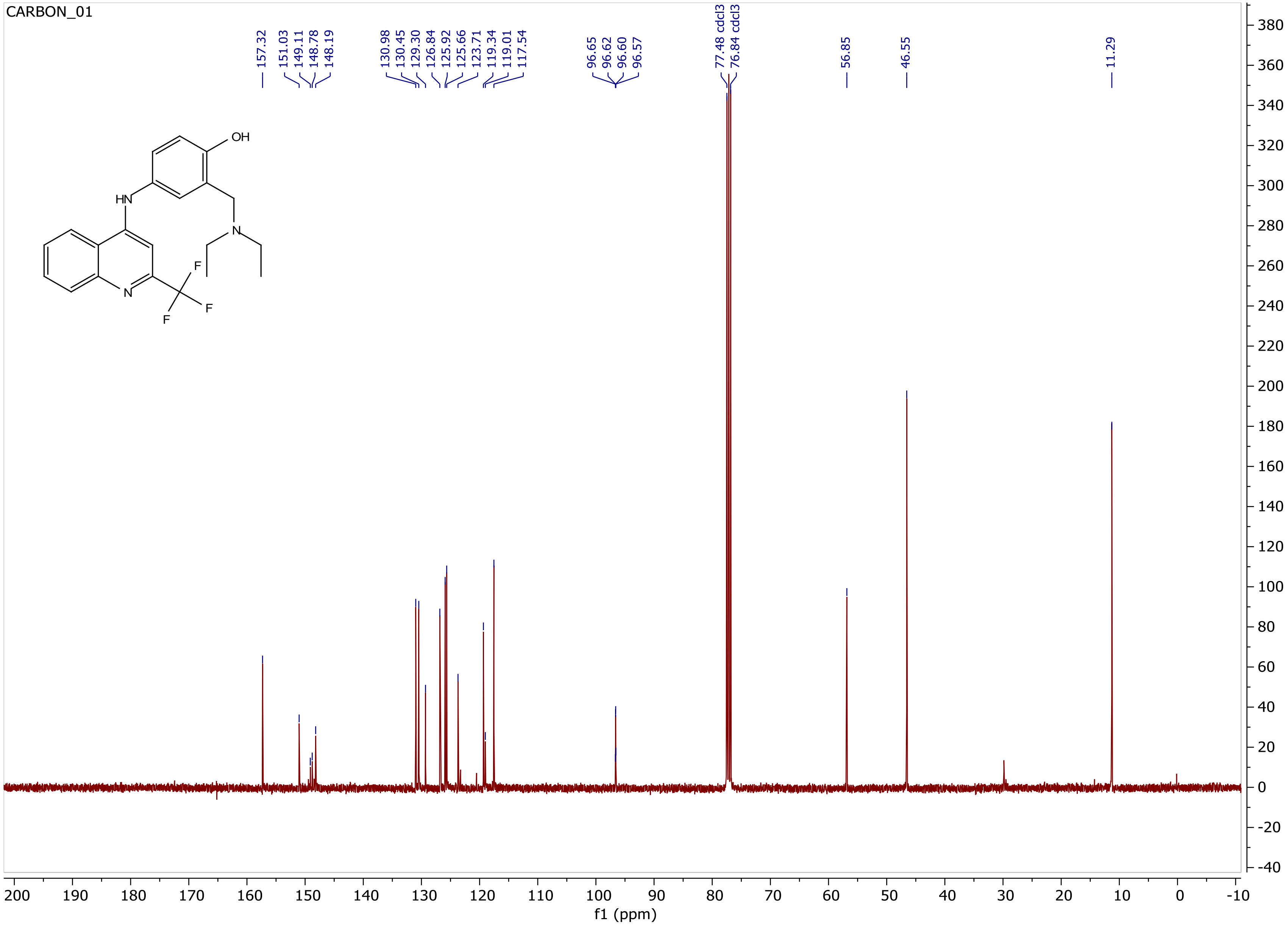

^{1}H NMR spectrum of 4-((3-((diethylamino)methyl)-4-hydroxyphenyl)amino)quinoline-6-carbonitrile (12)

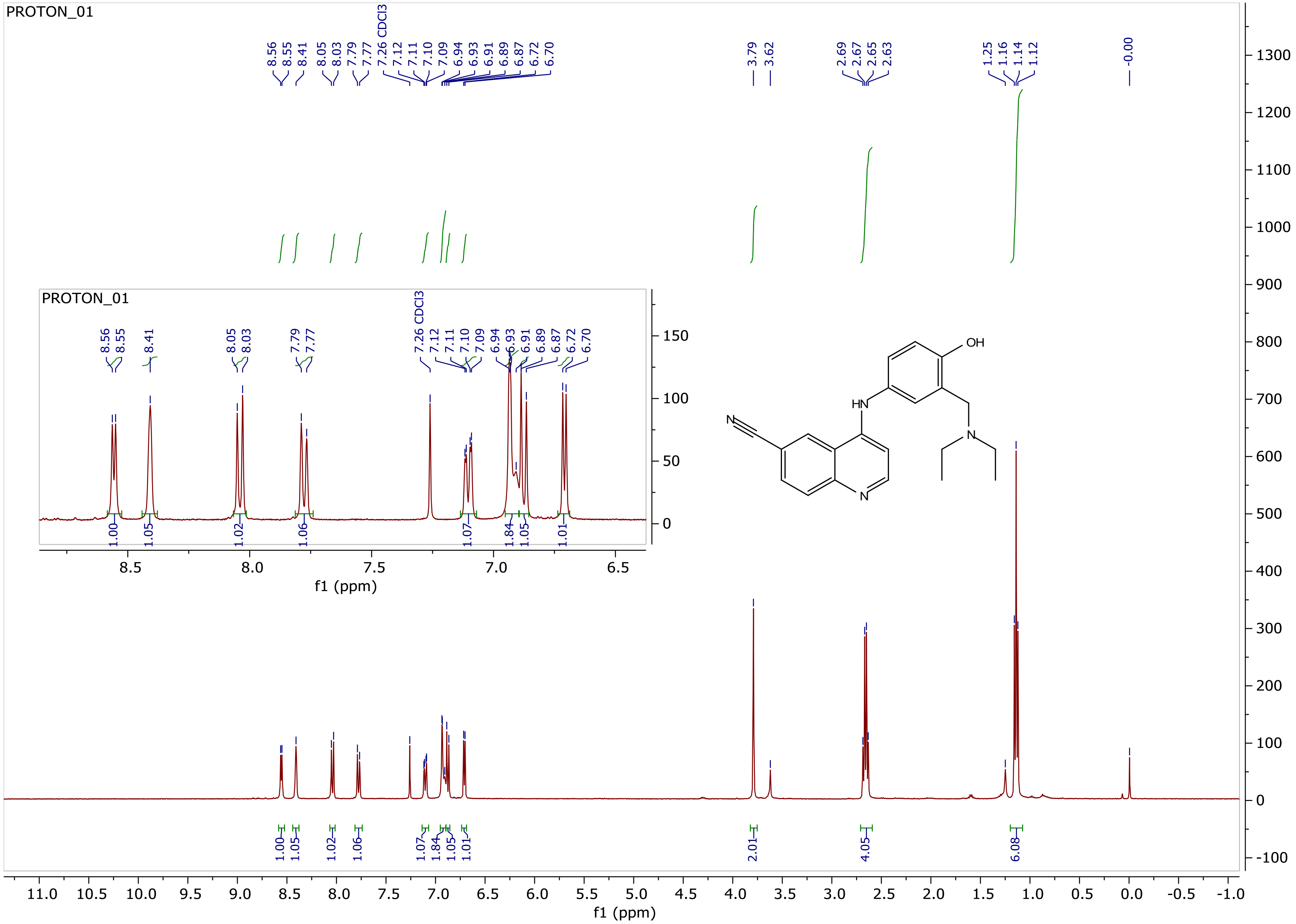

^{13}C NMR spectrum of 4-((3-((diethylamino)methyl)-4-hydroxyphenyl)amino)quinoline-6-carbonitrile (12)

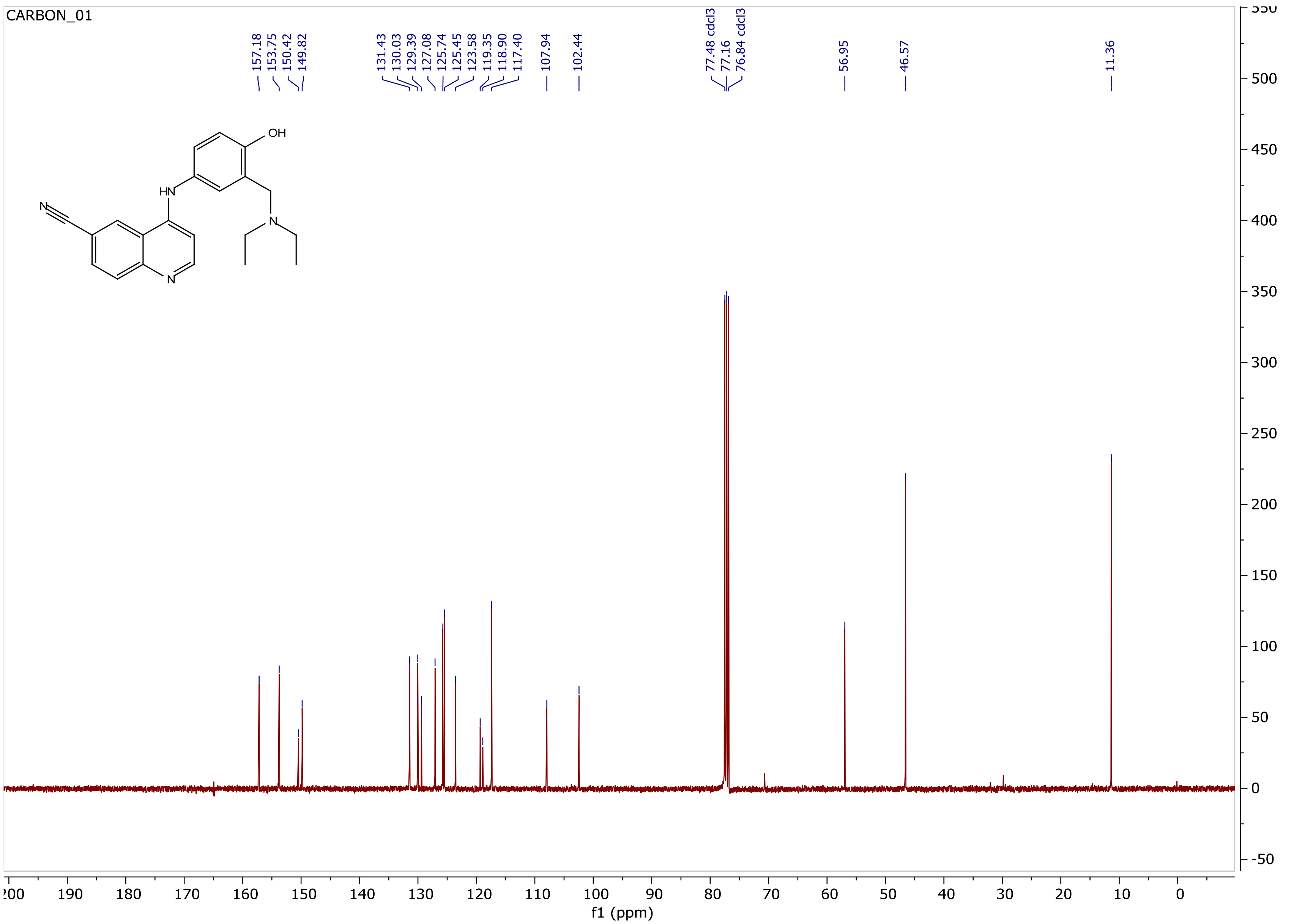

^{1}H NMR spectrum of 4-((3-((diethylamino)methyl)-4-hydroxyphenyl)amino)quinoline-5-carboxylic acid (13)

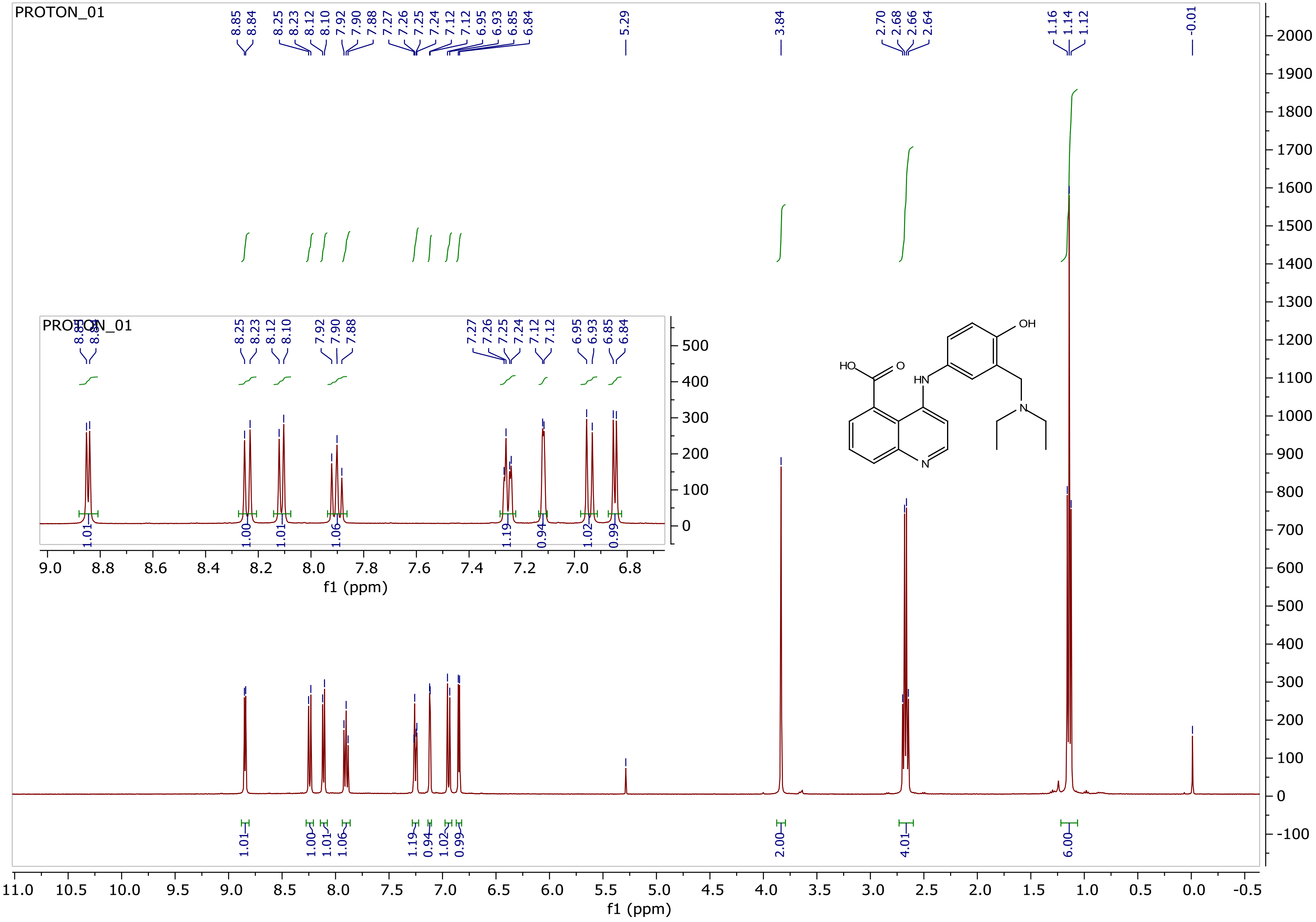

^{13}C NMR spectrum of 4-((3-((diethylamino)methyl)-4-hydroxyphenyl)amino)quinoline-5-carboxylic acid (13)

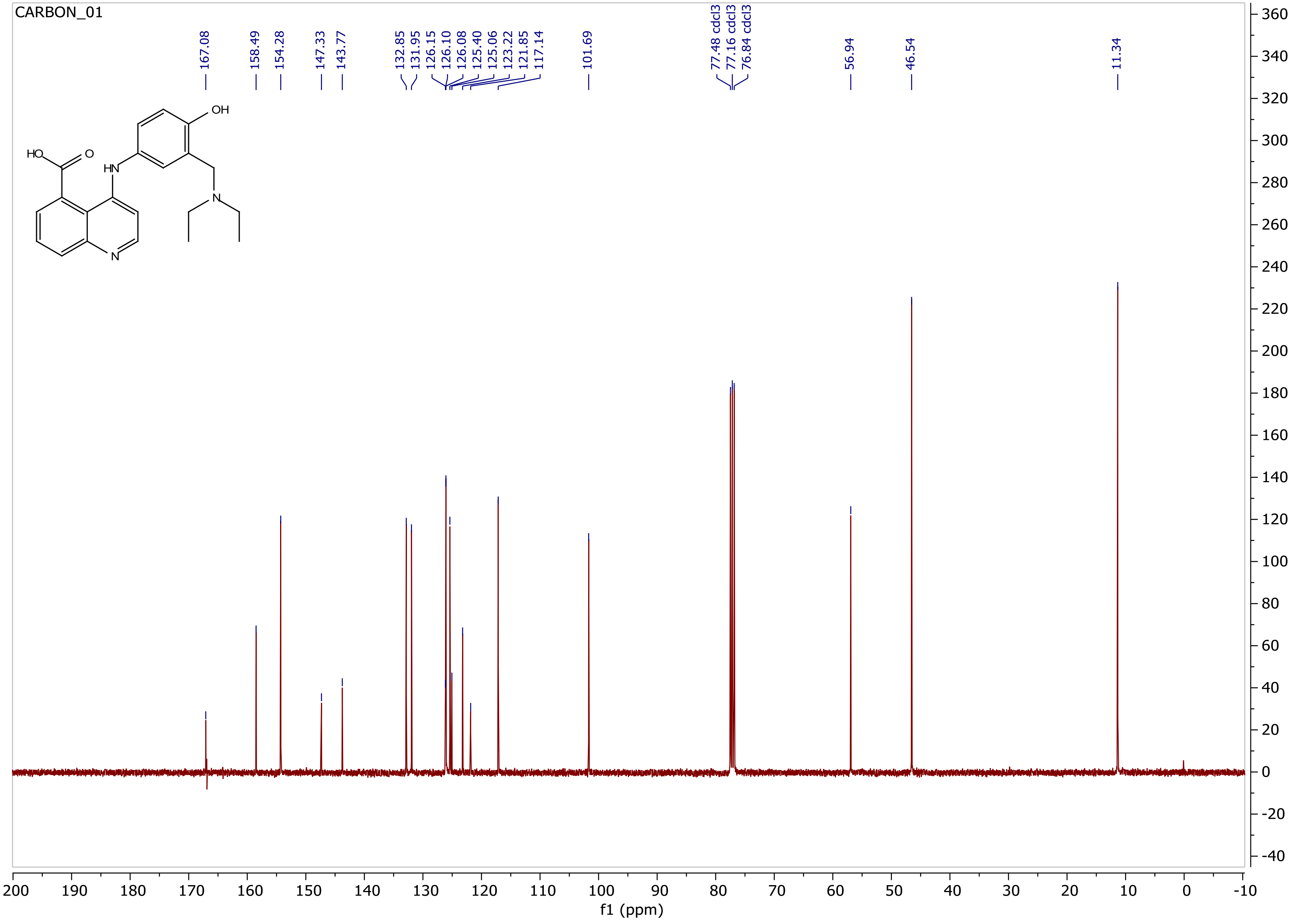

^{1}H NMR spectrum of 4-((7-chloroquinolin-4-yl)amino)-2-((ethyl(2-hydroxyethyl)amino)methyl)phenol (14)

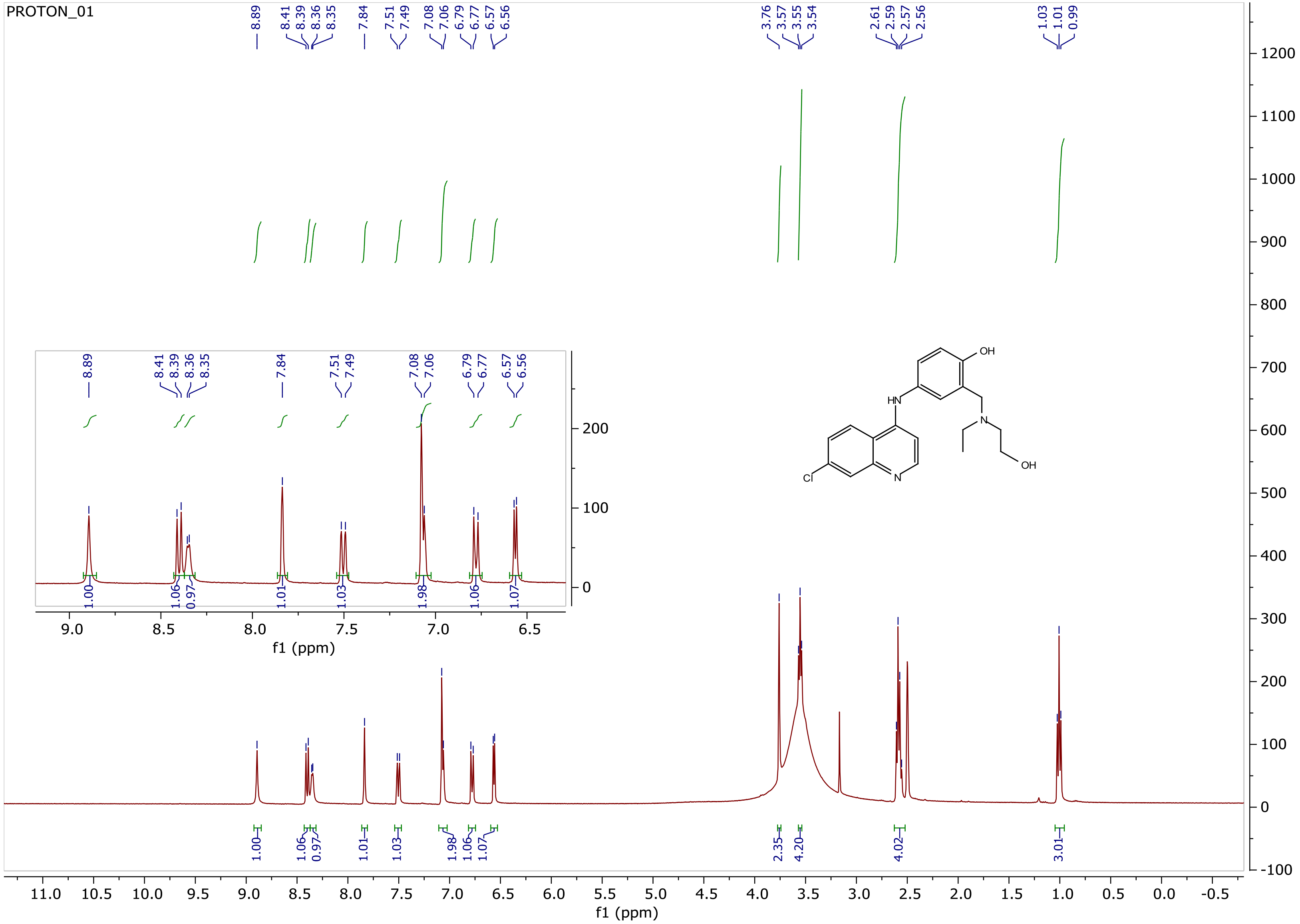

^{13}C NMR spectrum of 4-((7-chloroquinolin-4-yl)amino)-2-((ethyl(2-hydroxyethyl)amino)methyl)phenol (14)

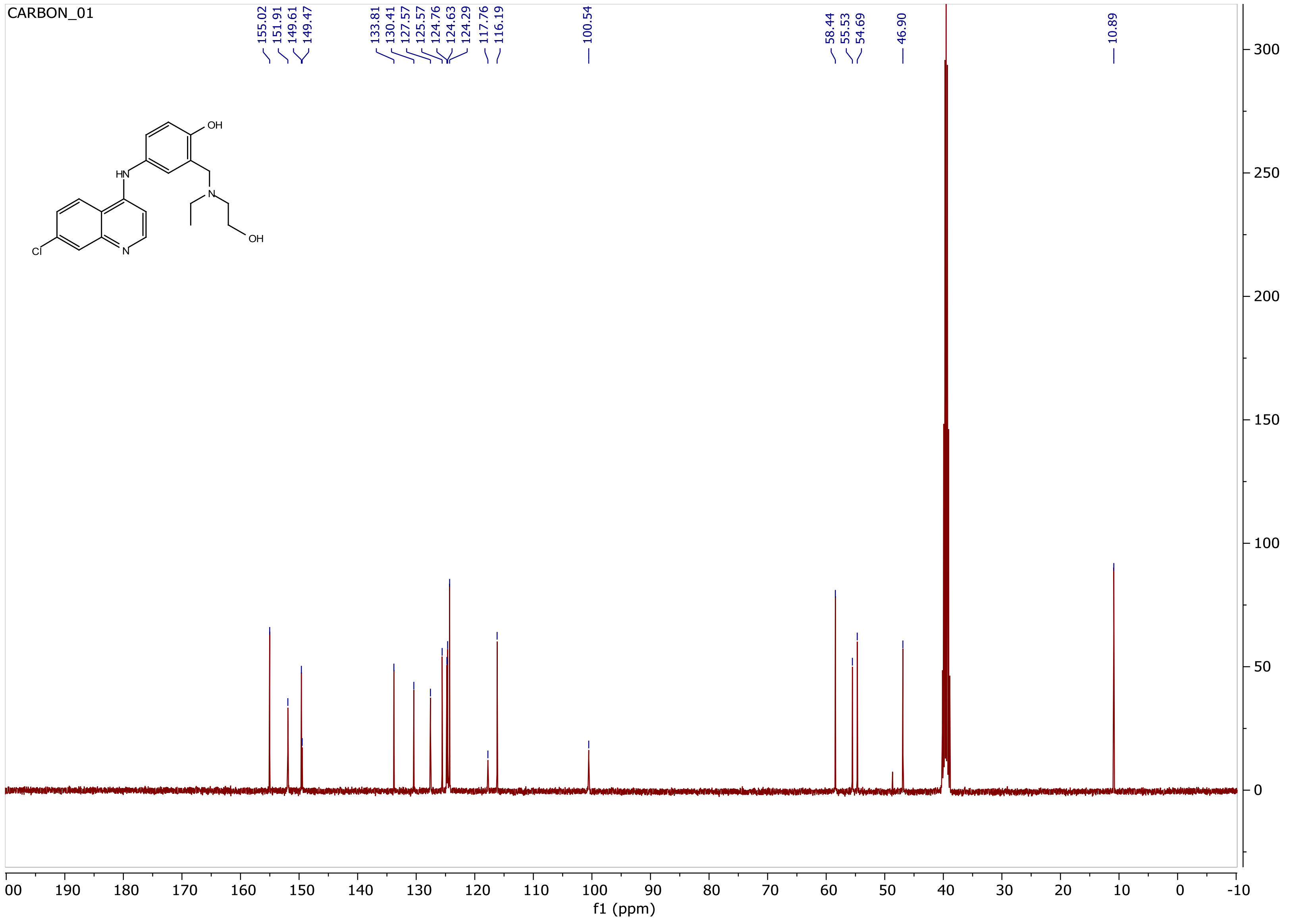

1.**2.4. HRMS spectra of the tested compounds**

Mass spectra were recorded on a 4800 Plus MALDI TOF/TOF Analyzer (Applied Biosystems, US instrument, Mundelein, USA) in positive ion mode (fixed laser intensity 4280) using CH_3CN/H_2O gradient with 0.2% HCOOH as the carrying solvent solution. Samples were dissolved in MeOH (HPLC grade purity) mixed with CHCA (α-Cyano-4-hydroxycinnamic acid) MALDI matrix (5 mg/mL in 50% acetonitrile (v/v)). Internal calibrants: Azithromycin and Azithromycin fragments (m/z 156-749).

HRMS data for tested compounds AMQ and 1 -1 4.

Specimen	Compound	Molecular formula	Ion	Calculated mass	Found mass	Error (ppm)
DO316	**AMQ**	$C_{20}H_{22}ClN_3O$	$[M+H]^+$	356.1530	356.1528	0.6
DO324	**1**	$C_{20}H_{22}FN_3O$	$[M+H]^+$	340.1825	340.1831	1.8
DO317	**2**	$C_{21}H_{22}F_3N_3O$	$[M+H]^+$	390.1793	390.1786	1.8
DO335	**3**	$C_{21}H_{22}N_4O$	$[M+H]^+$	347.1872	347.1871	0.3
AS36	**4**	$C_{20}H_{22}N_4O_3$	$[M+H]^+$	367.1770	367.1784	3.8
KBK33	**5**	$C_{21}H_{25}N_3O_2$	$[M+H]^+$	352.2025	352.2011	4.0
DB03	**6**	$C_{20}H_{23}N_3O$	$[M+H]^+$	322.1919	322.1923	1.2
DO323	**7**	$C_{20}H_{22}FN_3O$	$[M+H]^+$	340.1825	340.1823	0.6
DO334	**8**	$C_{20}H_{22}FN_3O$	$[M+H]^+$	340.1825	340.1828	0.9
DO320	**9**	$C_{21}H_{22}F_3N_3O$	$[M+H]^+$	390.1793	390.1798	1.3
DO321	**10**	$C_{21}H_{22}F_3N_3O$	$[M+H]^+$	390.1793	390.1797	1.0
DO322	**11**	$C_{21}H_{22}F_3N_3O$	$[M+H]^+$	390.1793	390.1797	1.0
DO325	**12**	$C_{21}H_{22}N_4O$	$[M+H]^+$	347.1872	347.1862	2.9
AS37	**13**	$C_{21}H_{23}N_3O_3$	$[M+H]^+$	366.1818	366.1815	0.8
KBK32	**14**	$C_{20}H_{22}ClN_3O_2$	$[M+H]^+$	372.1479	372.1486	1.9

AMQ

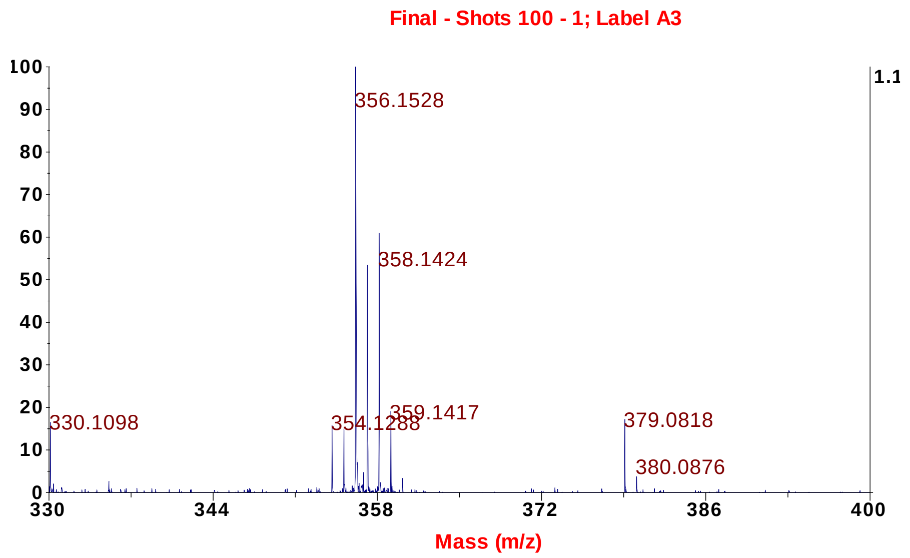

Final - Shots 100 - 1; Label A3
356.1528
358.1424
330.1098
354.1288
359.1417
379.0818
380.0876
1.1
Mass (m/z)

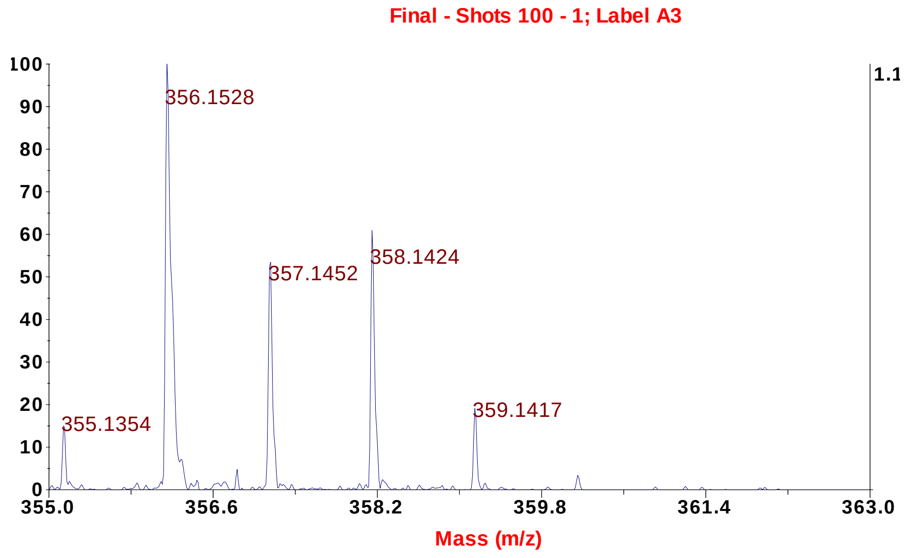

Final - Shots 100 - 1; Label A3
356.1528
357.1452
358.1424
355.1354
359.1417
1.1
Mass (m/z)

Compound 1

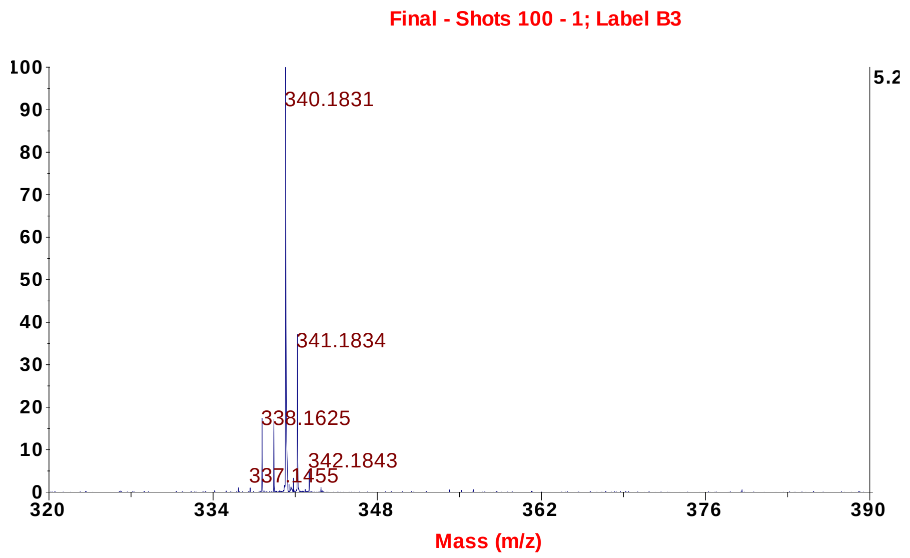
Final - Shots 100 - 1; Label B3
5.2
340.1831
341.1834
338.1625
342.1843
337.1455
Mass (m/z)

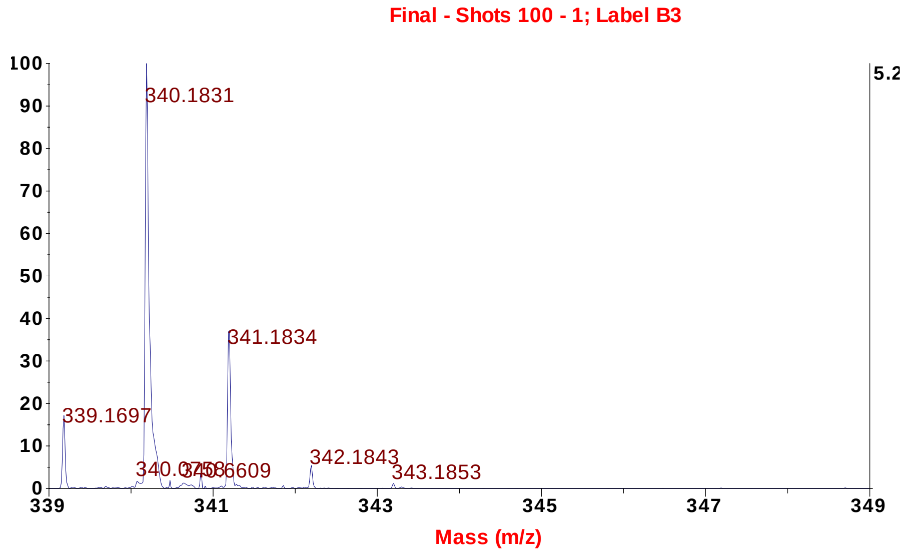
Final - Shots 100 - 1; Label B3
5.2
340.1831
341.1834
339.1697
342.1843
343.1853
Mass (m/z)

Compound 2

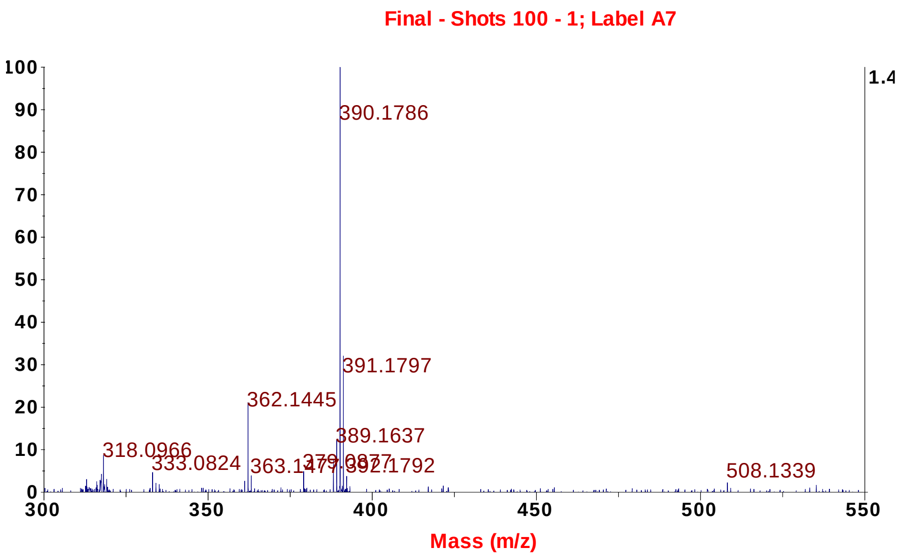
Final - Shots 100 - 1; Label A7
390.1786
391.1797
362.1445
389.1637
318.0966
333.0824
363.1477
379.0877
392.1792
508.1339
1.4
Mass (m/z)

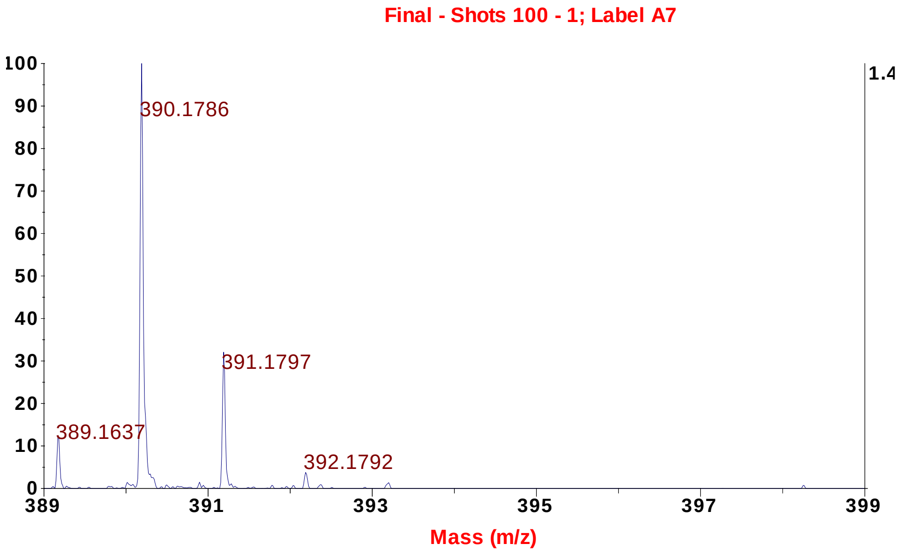
Final - Shots 100 - 1; Label A7
390.1786
391.1797
389.1637
392.1792
1.4
Mass (m/z)

Compound 3

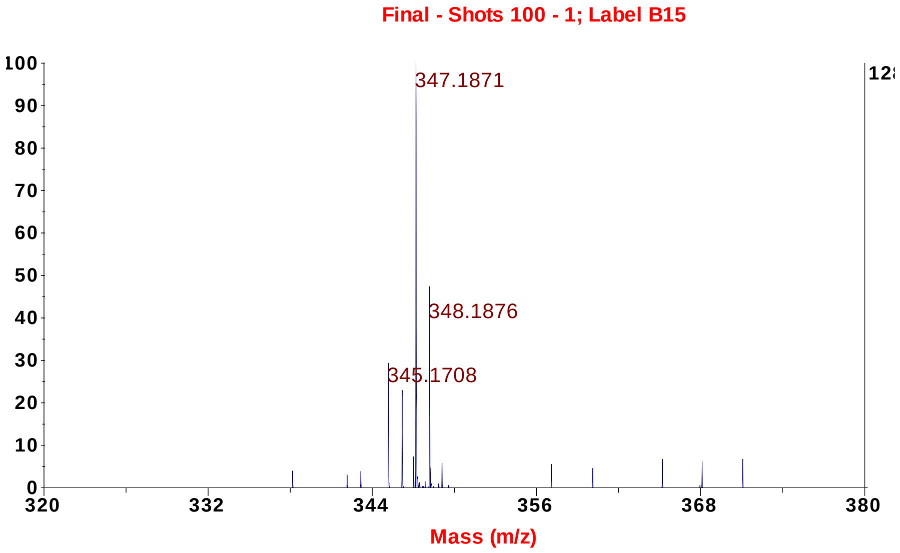
Final - Shots 100 - 1; Label B15
100
90
80
70
60
50
40
30
20
10
0
347.1871
348.1876
345.1708
320
332
344
356
368
380
Mass (m/z)

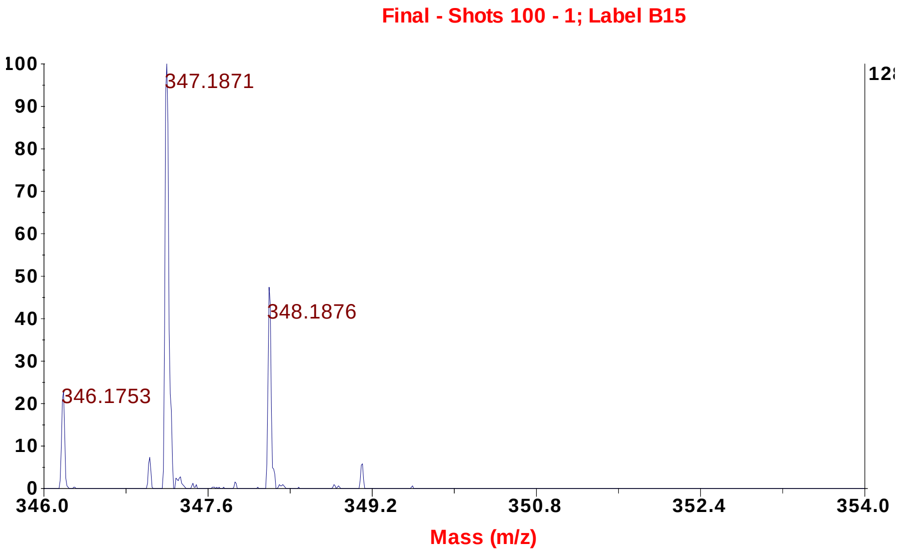
Final - Shots 100 - 1; Label B15
100
90
80
70
60
50
40
30
20
10
0
347.1871
348.1876
346.1753
346.0
347.6
349.2
350.8
352.4
354.0
Mass (m/z)

Compound 4

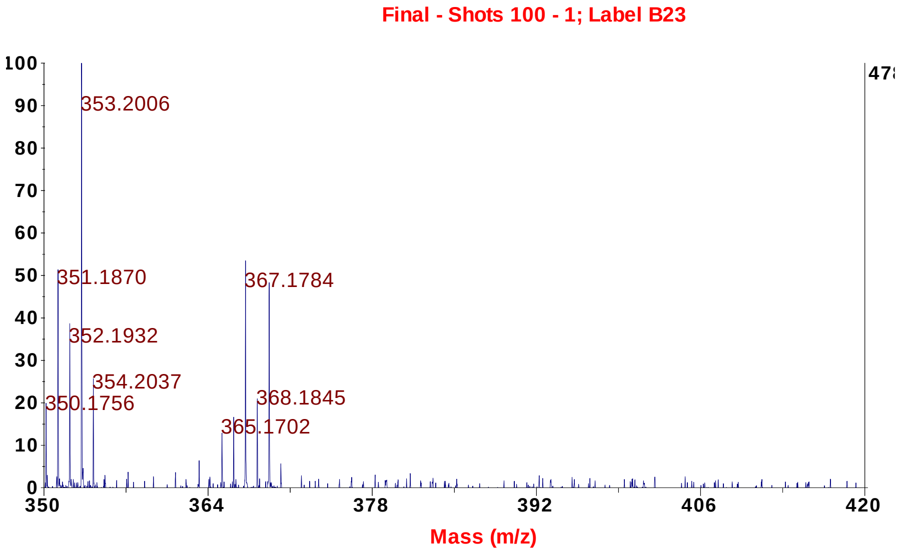
Final - Shots 100 - 1; Label B23
353.2006
351.1870
352.1932
354.2037
350.1756
367.1784
368.1845
365.1702
Mass (m/z)

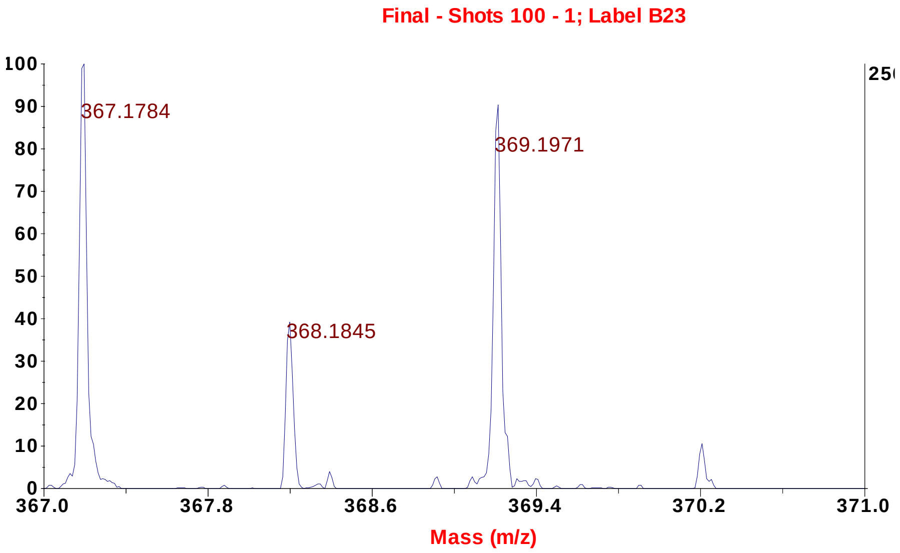
Final - Shots 100 - 1; Label B23
367.1784
369.1971
368.1845
Mass (m/z)

Compound 5

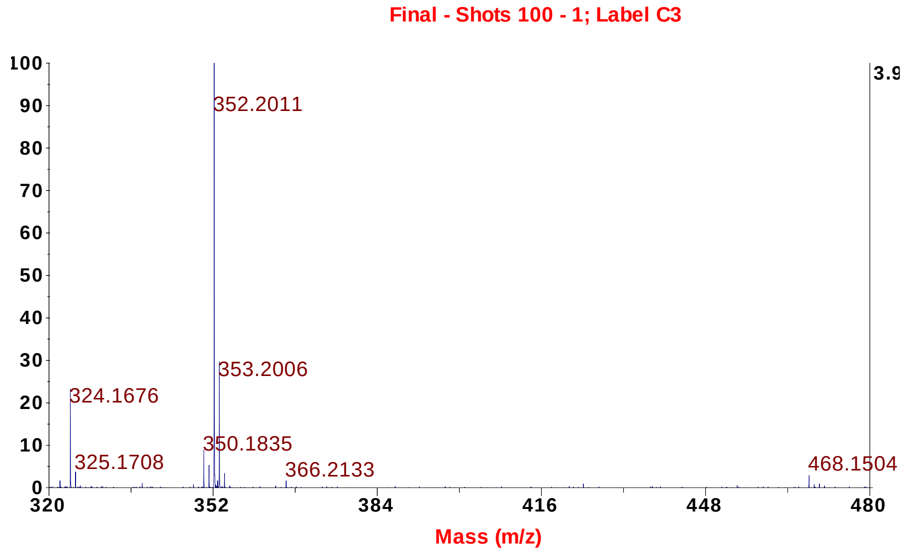

Final - Shots 100 - 1; Label C3
352.2011
353.2006
324.1676
350.1835
325.1708
366.2133
468.1504
3.9
Mass (m/z)

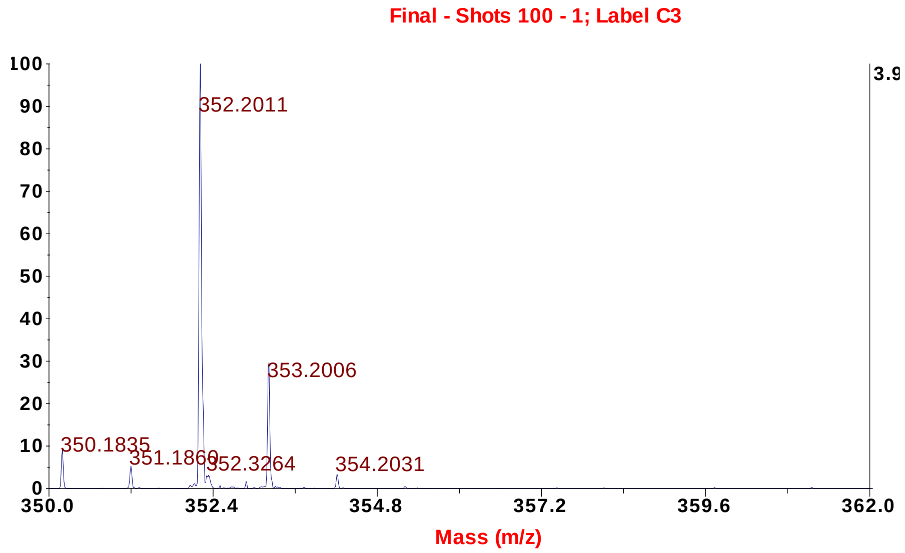

Final - Shots 100 - 1; Label C3
352.2011
353.2006
350.1835
351.1860
352.3264
354.2031
3.9
Mass (m/z)

Compound 6

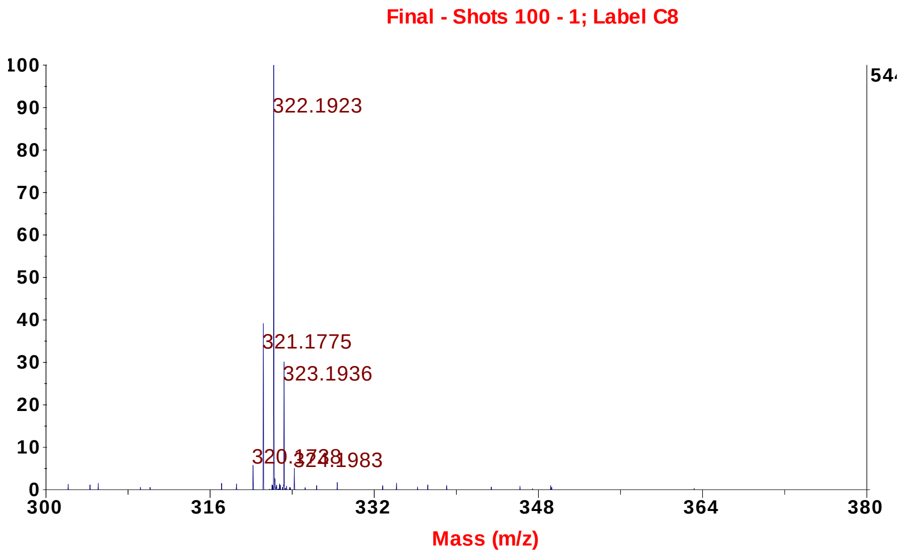

Final - Shots 100 - 1; Label C8
322.1923
321.1775
323.1936
320.1728
324.1983
Mass (m/z)

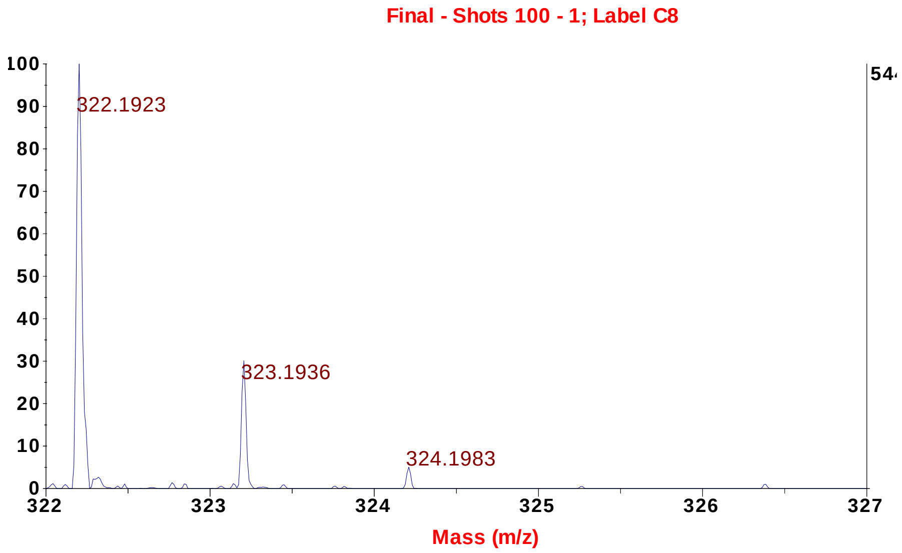

Final - Shots 100 - 1; Label C8
322.1923
323.1936
324.1983
Mass (m/z)

Compound 7

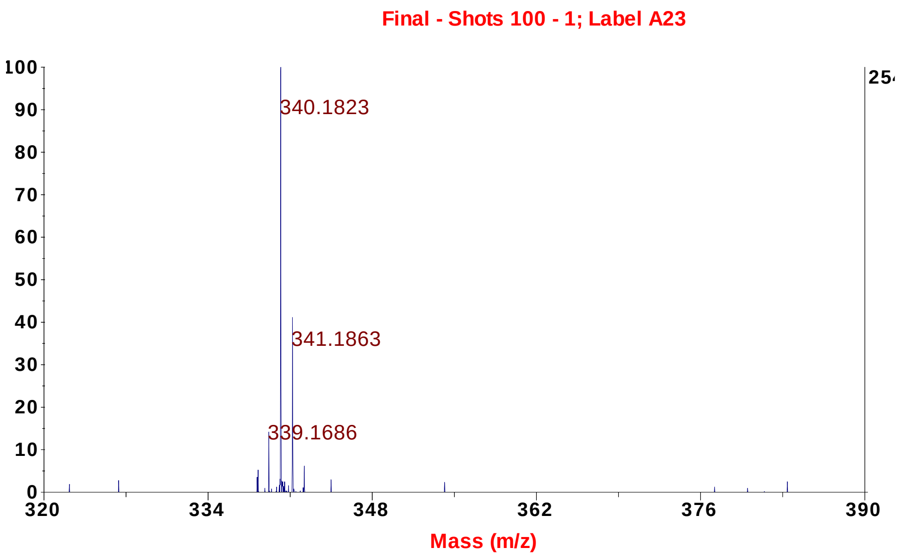

Final - Shots 100 - 1; Label A23
100
90
80
70
60
50
40
30
20
10
0
25
340.1823
341.1863
339.1686
320
334
348
362
376
390
Mass (m/z)

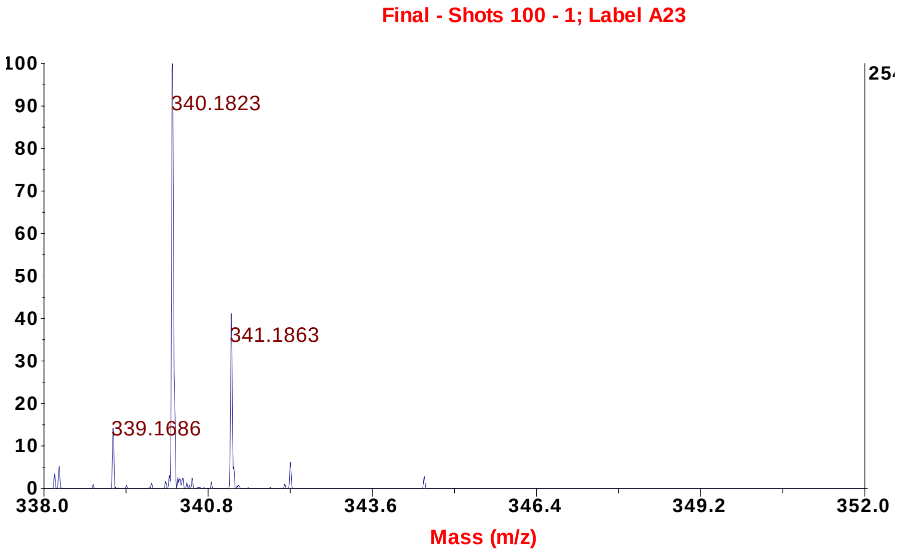

Final - Shots 100 - 1; Label A23
100
90
80
70
60
50
40
30
20
10
0
25
340.1823
341.1863
339.1686
338.0
340.8
343.6
346.4
349.2
352.0
Mass (m/z)

Compound 8

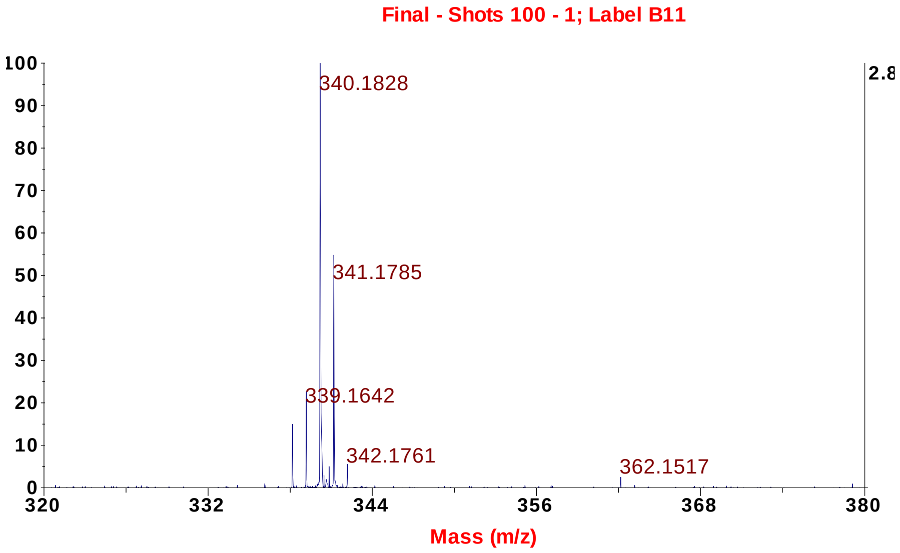
Final - Shots 100 - 1; Label B11
340.1828
341.1785
339.1642
342.1761
362.1517
Mass (m/z)

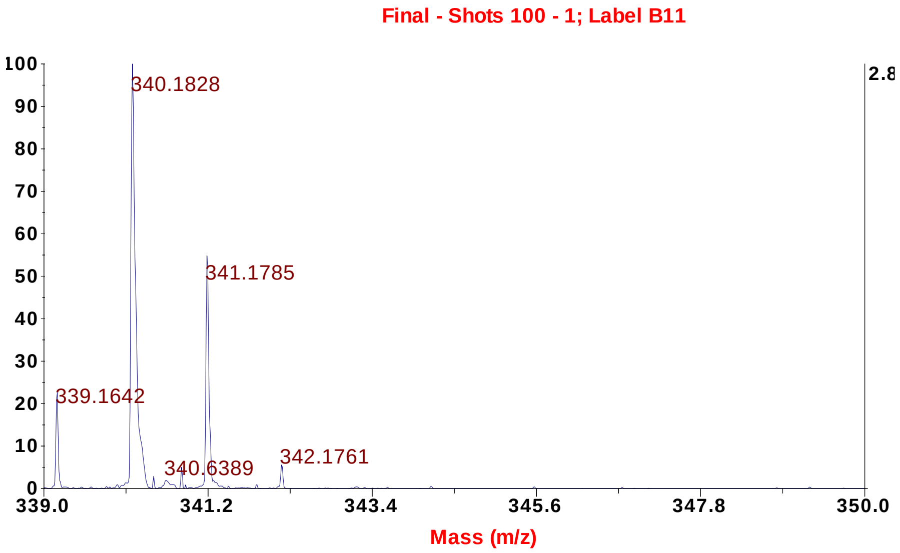
Final - Shots 100 - 1; Label B11
340.1828
341.1785
339.1642
340.6389
342.1761
Mass (m/z)

Compound 9

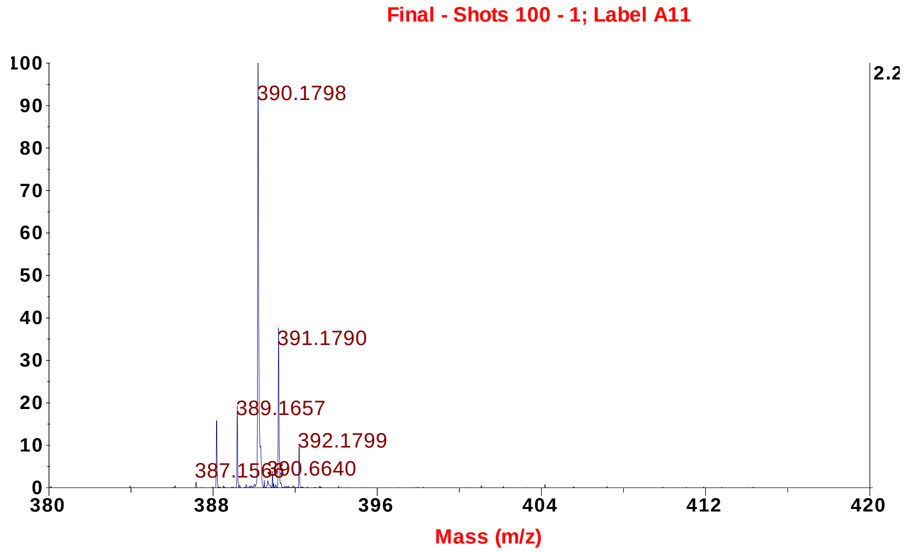

Final - Shots 100 - 1; Label A11
390.1798
391.1790
389.1657
392.1799
387.1568
390.6640
2.2
Mass (m/z)

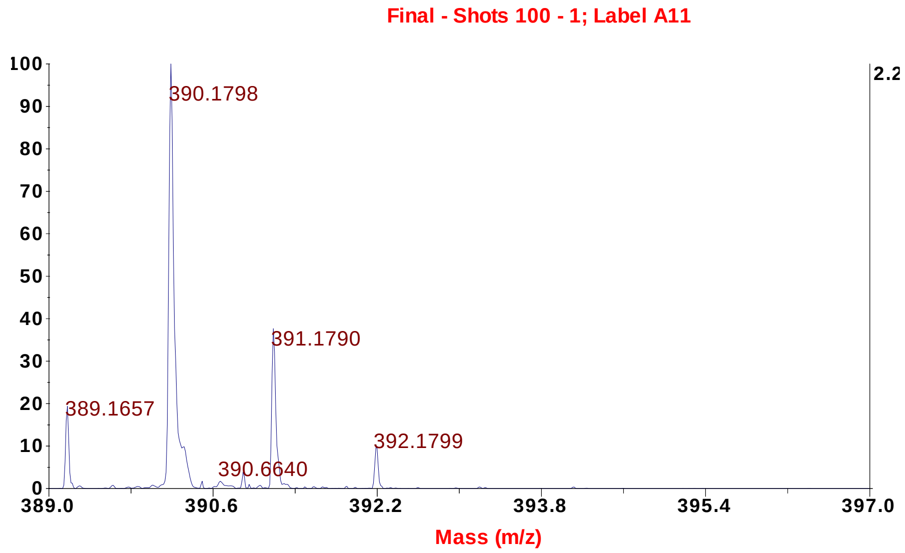

Final - Shots 100 - 1; Label A11
390.1798
391.1790
389.1657
392.1799
390.6640
2.2
Mass (m/z)

Compound 10

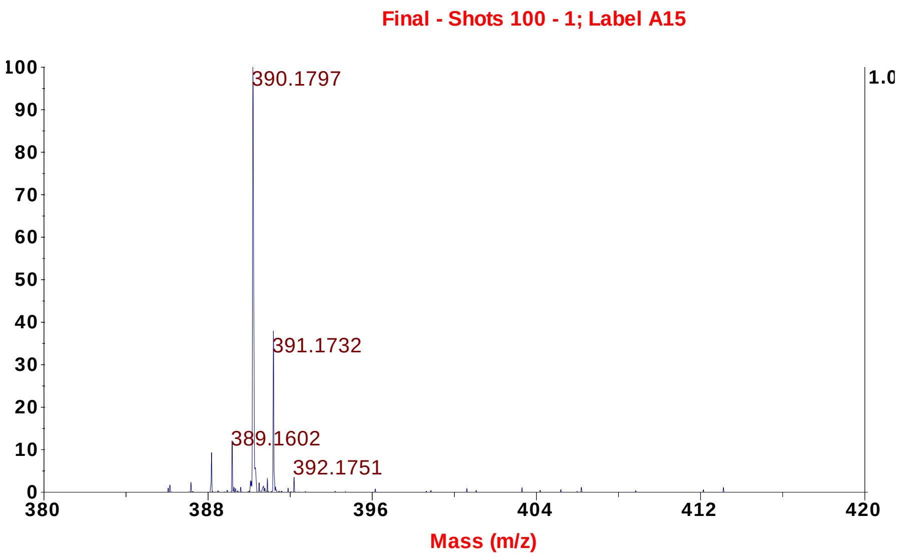
Final - Shots 100 - 1; Label A15
390.1797
391.1732
389.1602
392.1751
1.0
100
90
80
70
60
50
40
30
20
10
0
380
388
396
404
412
420
Mass (m/z)

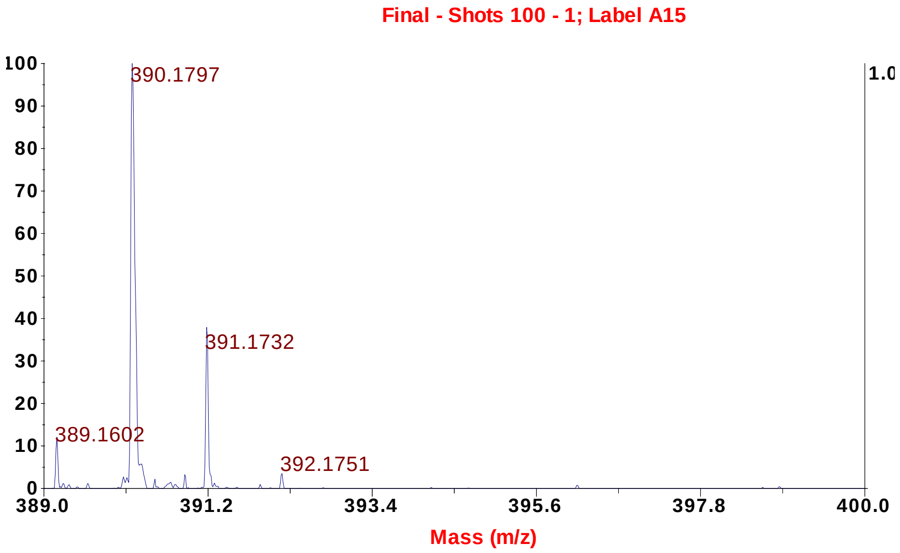
Final - Shots 100 - 1; Label A15
390.1797
391.1732
389.1602
392.1751
1.0
100
90
80
70
60
50
40
30
20
10
0
389.0
391.2
393.4
395.6
397.8
400.0
Mass (m/z)

Compound 11

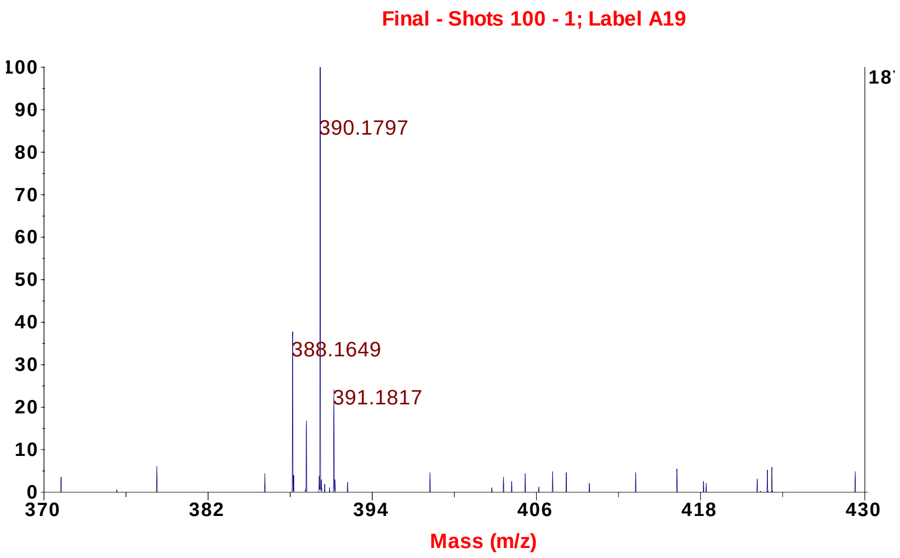

Final - Shots 100 - 1; Label A19
390.1797
388.1649
391.1817
Mass (m/z)

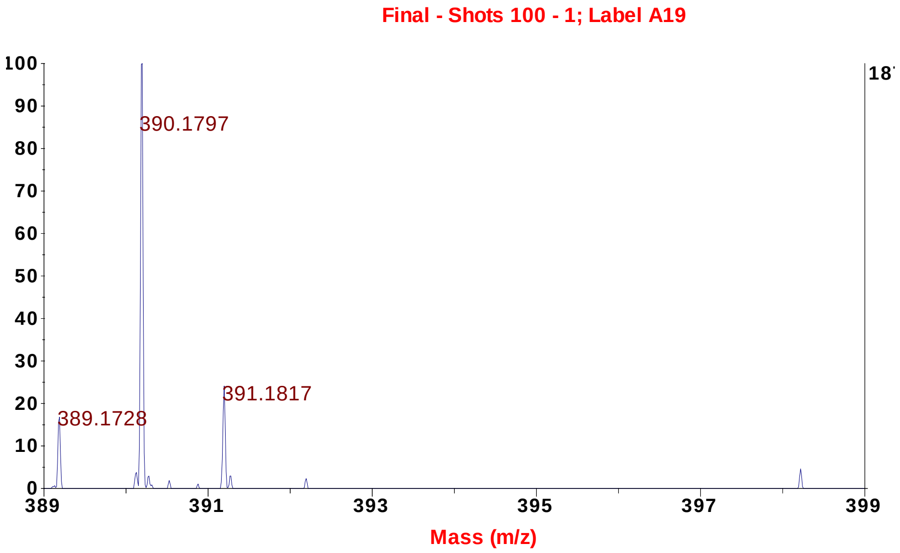

Final - Shots 100 - 1; Label A19
390.1797
391.1817
389.1728
Mass (m/z)

Compound 12

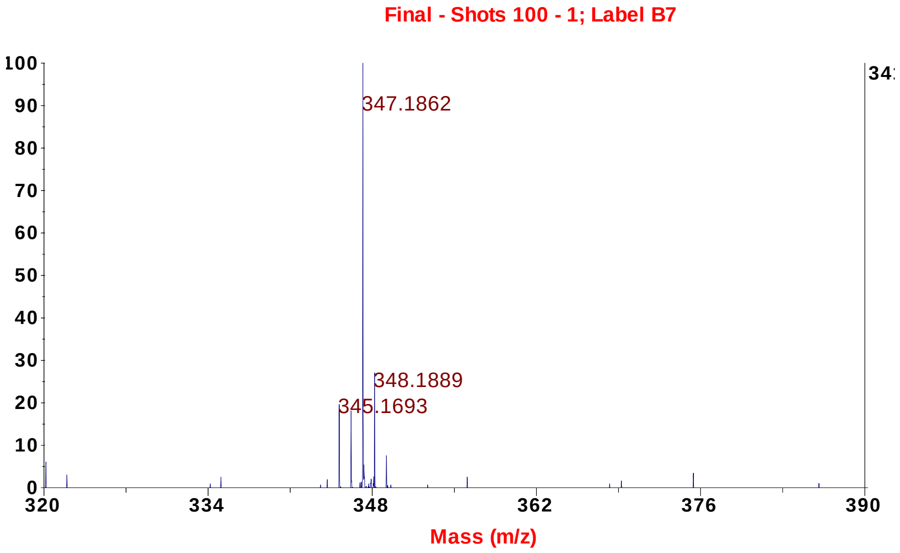
Final - Shots 100 - 1; Label B7
347.1862
348.1889
345.1693
Mass (m/z)

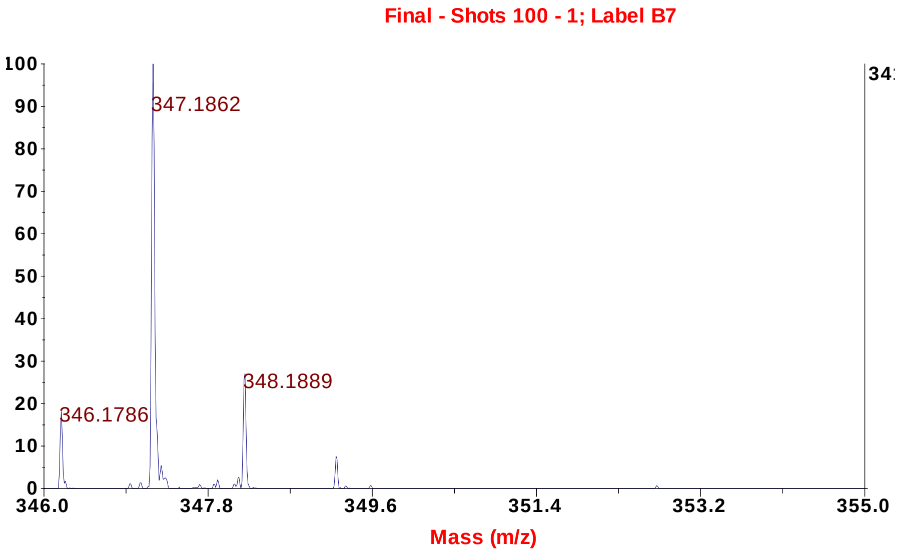
Final - Shots 100 - 1; Label B7
347.1862
348.1889
346.1786
Mass (m/z)

Compound 13

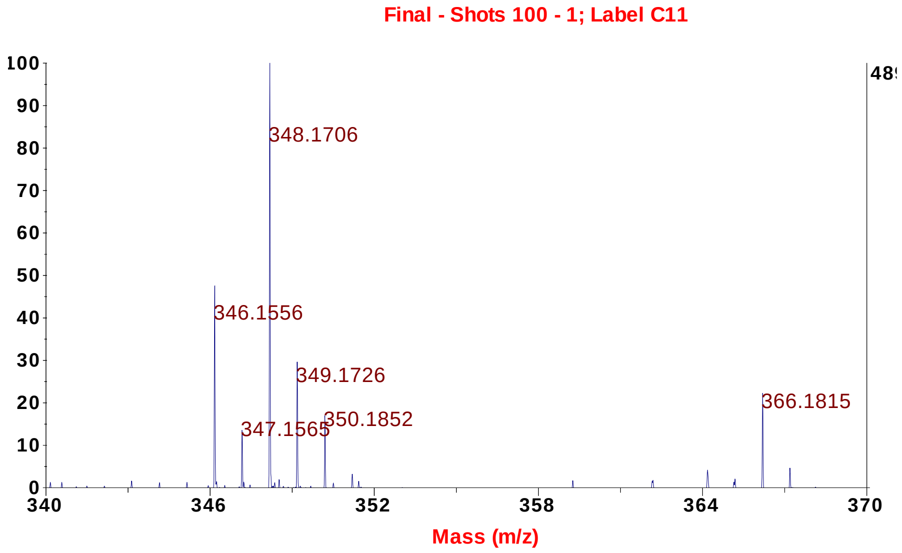

Final - Shots 100 - 1; Label C11
348.1706
346.1556
349.1726
366.1815
347.1565
350.1852
Mass (m/z)

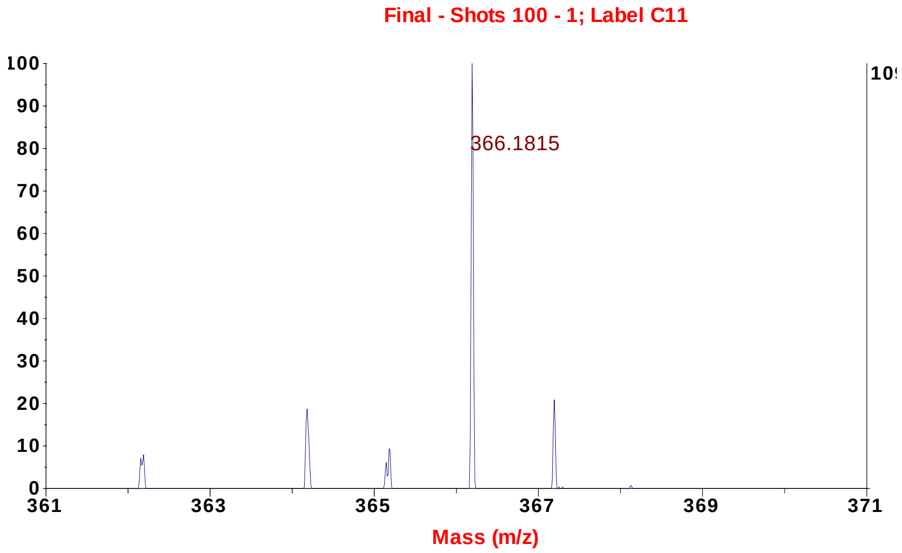

Final - Shots 100 - 1; Label C11
366.1815
Mass (m/z)

Compound 14

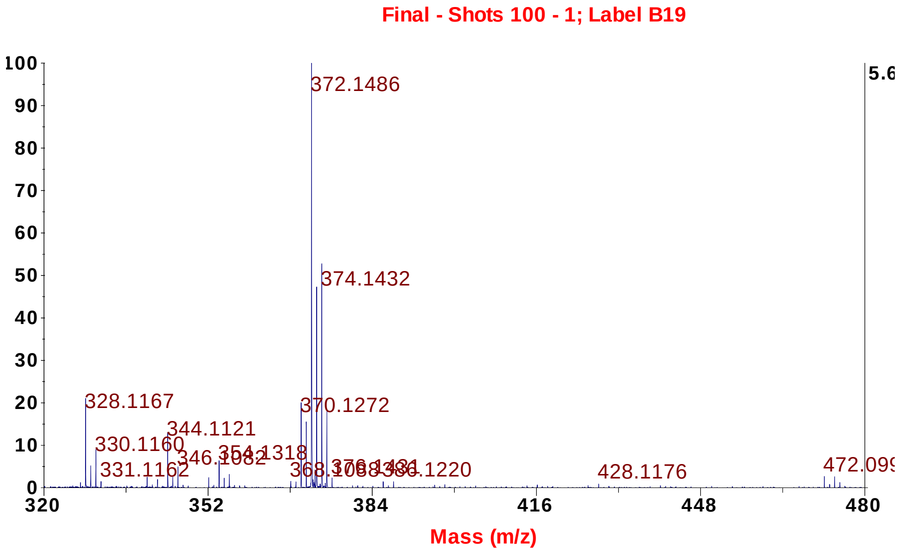

Final - Shots 100 - 1; Label B19
372.1486
374.1432
328.1167
370.1272
344.1121
330.1160
346.1082
354.1318
331.1162
368.1098
386.1220
428.1176
Mass (m/z)

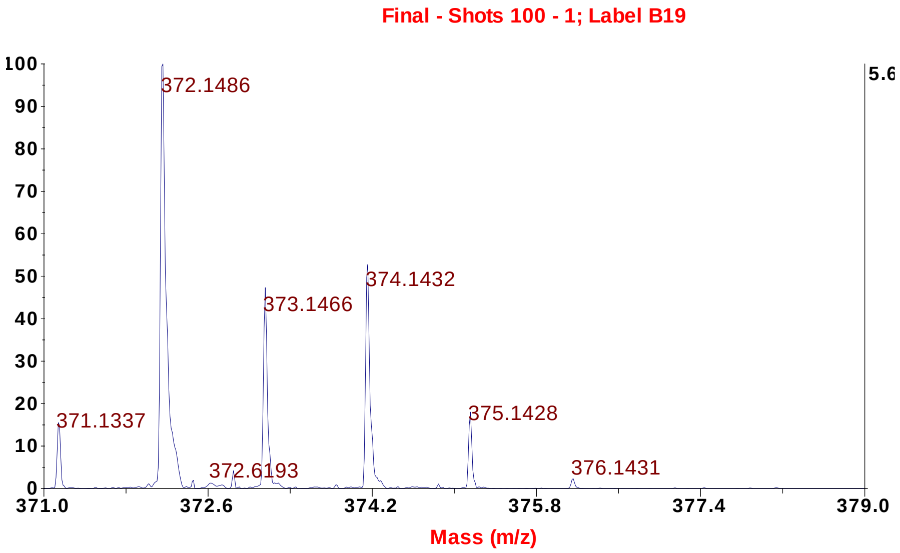

Final - Shots 100 - 1; Label B19
372.1486
374.1432
373.1466
371.1337
375.1428
372.6193
376.1431
Mass (m/z)

2. List of interactions between tested compounds and cholinesterases

2.1. List of interactions between derivatives **1** – **14** and AChE

Interactions between compound **AMQ** and AChE.

Amino acid	Non-bonding interactions
Glu202	H39, hydrogen bond; electrostatic-salt bridge; attractive charge
Ser203	O19, hydrogen bond-conventional hydrogen bond
Glu202	H40, hydrogen bond-carbon hydrogen bond
Tyr341	N7, electrostatic-π-cation
Trp86	N21, electrostatic-π-cation
Trp86	N21, electrostatic-π-cation
Asp74	**AMQ**, electrostatic-π-anion
Trp286	**AMQ**, hydrophobic-π-π stacked
Phe338	**AMQ**, hydrophobic-π-π stacked
Tyr341	**AMQ**, hydrophobic-π-π stacked
Trp286	**AMQ**, hydrophobic-π-alkyl
Trp286	**AMQ**, hydrophobic-π-alkyl

Interactions between compound **1** and AChE.

Amino acid	Non-bonding interactions
Glu202	N21, electrostatic-attractive charge
Ser203	O19, hydrogen bond-conventional hydrogen bond
Tyr341	N7, electrostatic-π-cation
Trp86	N21, electrostatic-π-cation
Trp86	N21, electrostatic-π-cation
Asp74	**1**, electrostatic-π-anion
Trp86	H41, hydrophobic-π-sigma
Trp286	**1**, hydrophobic-π-π stacked
Phe338	**1**, hydrophobic-π-π stacked
Tyr341	**1**, hydrophobic-π-π stacked
Tyr341	**1**, hydrophobic-π-π stacked

Interactions between compound **2** and AChE.

Amino acid	Non-bonding interactions
HOH752	F14, hydrogen bond; halogen–water hydrogen bonds; conventional hydrogen bond; halogen (fluorine)
HOH772	F14, hydrogen bond; halogen–water hydrogen bonds; conventional hydrogen bond; halogen (fluorine)
HOH809	H41, hydrogen bond-water hydrogen bond; carbon hydrogen bond
Ser203	H32, hydrogen bond-conventional hydrogen bond
Arg296	H48, hydrogen bond-carbon hydrogen bond
Gly120	F15, halogen-halogen (fluorine)
Gu202	F13, halogen-halogen (fluorine)
Gu202	F15, halogen-halogen (fluorine)
Tyr124	H42, hydrogen bond; electrostatic-π-cation; π-donor hydrogen bond
Tyr124	**2**, other-π-lone pair
Trp86	**2**, hydrophobic-π-π T-shaped
Trp86	**2**, hydrophobic-π-π T-shaped
His447	**2**, hydrophobic-π-π T-shaped
His447	**2**, hydrophobic-π-π T-shaped
Trp286	C12, hydrophobic-π-alkyl
Trp286	C12, hydrophobic-π-alkyl

Interactions between compound **3** and AChE.

Amino acid	Non-bonding interactions
Glu202	H39, hydrogen bond; electrostatic-salt bridge; attractive charge
Gly121	O20, hydrogen bond-conventional hydrogen bond
Ser203	O20, hydrogen bond-conventional hydrogen bond

Ser203	O20, hydrogen bond-carbon hydrogen bond
Glu202	H40, hydrogen bond-carbon hydrogen bond
Glu202	H46, hydrogen bond-carbon hydrogen bond
Tyr341	N7, electrostatic-π-cation
Trp86	N22, electrostatic-π-cation
Trp86	N22, electrostatic-π-cation
Trp286	**3**, hydrophobic-π-π stacked
Phe338	**3**, hydrophobic-π-π stacked

Interactions between compound **4** and AChE.

Amino acid	Non-bonding interactions
HOH771	N7, hydrogen bond-water hydrogen bond; conventional hydrogen bond
HOH883	N7, hydrogen bond-water hydrogen bond; conventional hydrogen bond
Asp74	H33, hydrogen bond-conventional hydrogen bond
Tyr337	H40, hydrogen bond-conventional hydrogen bond
Tyr337	H38, hydrogen bond-carbon hydrogen bond
Trp86	N20, electrostatic-π-cation
Tyr72	N25, electrostatic-π-cation
Trp286	N25, electrostatic-π-cation
Trp286	N25, electrostatic-π-cation
Trp286	O27, electrostatic-π-anion
Tyr124	**4**, other-π-lone pair
Trp286	**4**, hydrophobic-π-π stacked
Tyr341	**4**, hydrophobic-π-π stacked
Tyr124	**4**, hydrophobic-π-π T-shaped

Interactions between compound **5** and AChE.

Amino acid	Non-bonding interactions
Asp74	H30, hydrogen bond; electrostatic-salt bridge; attractive charge
Glu202	N20, electrostatic-attractive charge
Gly121	O18, hydrogen bond-conventional hydrogen bond
Gly122	O18, hydrogen bond-conventional hydrogen bond
Ser203	O18, hydrogen bond-conventional hydrogen bond
Ser203	O18, hydrogen bond-carbon hydrogen bond
Asp74	H31, hydrogen bond-carbon hydrogen bond
Glu202	H45, hydrogen bond-carbon hydrogen bond
Glu202	H46, hydrogen bond-carbon hydrogen bond
Tyr341	N7, electrostatic-π-cation
Trp86	N20, electrostatic-π-cation
Tyr124	**5**, other-π-lone pair
Trp286	C26, hydrophobic-π-alkyl

Interactions between compound **6** and AChE.

Amino acid	Non-bonding interactions
Asp74	H29, hydrogen bond; electrostatic-salt bridge; attractive charge
Glu202	N20, electrostatic-attractive charge
Gly121	O18, hydrogen bond-conventional hydrogen bond
His447	H36, hydrogen bond-conventional hydrogen bond
Asp74	H30, hydrogen bond-carbon hydrogen bond
Tyr341	N7, electrostatic-π-cation
Trp86	N20, electrostatic-π-cation
Trp86	N20, electrostatic-π-cation
Tyr124	**6**, other-π-lone pair
Tyr124	**6**, hydrophobic-π-π T-shaped

Interactions between compound **7** and AChE.

Amino acid	Non-bonding interactions
Asp74	H29, hydrogen bond; electrostatic-salt bridge; attractive charge
Glu202	N20, electrostatic-attractive charge

Gly121	O18, hydrogen bond-conventional hydrogen bond
Gly122	O18, hydrogen bond-conventional hydrogen bond
Ser203	H36, hydrogen bond-conventional hydrogen bond
Asp74	H30, hydrogen bond-carbon hydrogen bond
Glu202	H41, hydrogen bond-carbon hydrogen bond
Arg296	F25, halogen-halogen (fluorine)
Tyr341	N7, electrostatic-π-cation
Trp86	N20, electrostatic-π-cation
Trp86	N20, electrostatic-π-cation
Tyr124	**7**, other-π-lone pair
Tyr124	**7**, hydrophobic-π-π T-shaped
Phe338	**7**, hydrophobic-π-π T-shaped

Interactions between compound **8** and AChE.

Amino acid	Non-bonding interactions
Glu202	N20, electrostatic-attractive charge
HOH809	F25, hydrogen bond; halogen–water hydrogen bonds; conventional hydrogen bond; halogen (fluorine)
Trp86	N20, electrostatic-π-cation
Trp86	N20, electrostatic-π-cation
Trp86	H45, hydrophobic-π-sigma
Trp286	**8**, hydrophobic-π-π stacked
Phe338	**8**, hydrophobic-π-π stacked
Tyr341	**8**, hydrophobic-π-π stacked
Tyr341	**8**, hydrophobic-π-π stacked

Interactions between compound **9** and AChE.

Amino acid	Non-bonding interactions
Glu202	N20, electrostatic-attractive charge
HOH718	F26, hydrogen bond; halogen–water hydrogen bonds; conventional hydrogen bond; halogen (fluorine)
Gly121	O18, hydrogen bond-conventional hydrogen bond
Ser203	O18, hydrogen bond-conventional hydrogen bond
Trp286	F27, hydrogen bond-carbon hydrogen bond
Arg296	F28, halogen-halogen (fluorine)
Trp86	N20, electrostatic-π-cation
Trp86	N20, electrostatic-π-cation
Tyr341	H32, hydrogen bond; electrostatic-π-cation; π-donor hydrogen bond
Phe338	**9**, hydrophobic-π-π T-shaped
Tyr341	**9**, hydrophobic-π-π T-shaped
Tyr341	**9**, hydrophobic-π-π T-shaped
Tyr124	C25, hydrophobic-π-alkyl

Interactions between compound **10** and AChE.

Amino acid	Non-bonding interactions
Asp74	H35, hydrogen bond-conventional hydrogen bond
Trp286	N7, electrostatic-π-cation
Trp86	N20, electrostatic-π-cation
Tyr124	**10**, other-π-lone pair
Trp286	**10**, hydrophobic-π-π stacked
Trp286	**10**, hydrophobic-π-π stacked
Trp286	**10**, hydrophobic-π-π stacked
Trp286	**10**, hydrophobic-π-π stacked
Tyr341	**10**, hydrophobic-π-π stacked
Tyr124	**10**, hydrophobic-π-π T-shaped
Trp286	C25, hydrophobic-π-alkyl
Trp286	C25, hydrophobic-π-alkyl

Interactions between compound **11** and AChE.

Amino acid	Non-bonding interactions
Tyr337	O18, hydrogen bond-conventional hydrogen bond
Tyr124	H40, hydrogen bond-conventional hydrogen bond
Glu285	F27, halogen–halogen (fluorine)
Glu285	F28, halogen–halogen (fluorine)
Tyr341	O18, electrostatic-π-anion
Tyr124	**11**, other-π-lone pair
Tyr72	**11**, hydrophobic-π-π stacked
Tyr72	**11**, hydrophobic-π-π stacked
Trp286	**11**, hydrophobic-π-π stacked
Tyr72	C25, hydrophobic-π-alkyl
Tyr124	C25, hydrophobic-π-alkyl

Interactions between compound **12** and AChE.

Amino acid	Non-bonding interactions
Glu202	N20, electrostatic-attractive charge
Glu202	H40, hydrogen bond-conventional hydrogen bond
Glu202	H47, hydrogen bond-carbon hydrogen bond
Tyr341	N7, electrostatic-π-cation
Trp86	N20, electrostatic-π-cation
Tyr124	**12**, hydrogen bond-π-donor hydrogen bond
Trp286	**12**, hydrophobic-π-π stacked
Tyr341	**12**, hydrophobic-π-π stacked
Tyr124	**12**, hydrophobic-π-π T-shaped

Interactions between compound **13** and AChE.

Amino acid	Non-bonding interactions
Asp74	N7, electrostatic-attractive charge
HOH809	O26, hydrogen bond-water hydrogen bond; conventional hydrogen bond
HOH883	O27, hydrogen bond-water hydrogen bond; conventional hydrogen bond
HOH809	H34, hydrogen bond-water hydrogen bond; conventional hydrogen bond
Tyr337	O18, hydrogen bond-conventional hydrogen bond
Tyr124	H40, hydrogen bond-conventional hydrogen bond
Trp286	N7, electrostatic-π-cation
Trp286	N7, electrostatic-π-cation
Asp74	**13**, electrostatic-π-anion
Tyr124	**13**, other-π-lone pair
Trp286	**13**, hydrophobic-π-π stacked
Trp286	**13**, hydrophobic-π-π stacked
Trp286	**13**, hydrophobic-π-π stacked
Trp286	**13**, hydrophobic-π-π stacked
Tyr72	**13**, hydrophobic-π-π T-shaped
Tyr124	**13**, hydrophobic-π-π T-shaped

Interactions between compound **14** and AChE.

Amino acid	Non-bonding interactions
Asp74	N21, electrostatic-attractive charge
HOH809	H39, hydrogen bond-water hydrogen bond; carbon hydrogen bond
Tyr124	O19, hydrogen bond-conventional hydrogen bond
Asp74	H37, hydrogen bond-conventional hydrogen bond
Phe338	O26, hydrogen bond-carbon hydrogen bond
Tyr124	H38, hydrogen bond-carbon hydrogen bond
Tyr124	H41, hydrogen bond-carbon hydrogen bond
Tyr337	H47, hydrogen bond-carbon hydrogen bond
Tyr341	H50, hydrogen bond-π-donor hydrogen bond
Tyr72	**14**, hydrophobic-π-π stacked
Trp286	**14**, hydrophobic-π-π stacked
Trp286	**14**, hydrophobic-π-π stacked
Trp286	**14**, hydrophobic-π-π T-shaped

2.2. List of interactions between derivatives **1 – 14** and BChE

Interactions between compound **AMQ** and BChE.

Amino acid	Non-bonding interactions
Glu197	N21, electrostatic-attractive charge
HOH825	H36, hydrogen bond-water hydrogen bond; conventional hydrogen bond
Ser287	H29, hydrogen bond-conventional hydrogen bond
Ser287	H30, hydrogen bond-carbon hydrogen bond
Trp82	N21, electrostatic-π-cation
Trp82	H39, hydrogen bond; electrostatic-π-cation; π-donor hydrogen bond
Phe329	**AMQ**, hydrophobic-π-π T-shaped
Gly116;Gly117	**AMQ**, hydrophobic-amide-π stacked
Leu286	Cl12, hydrophobic-alkyl
Trp231	Cl12, hydrophobic-π-alkyl
Trp231	Cl12, hydrophobic-π-alkyl
Phe398	Cl12, hydrophobic-π-alkyl
Leu286	**AMQ**, hydrophobic-π-alkyl

Interactions between compound **1** and BChE.

Amino acid	Non-bonding interactions
Glu197	N21, electrostatic-attractive charge
HOH786	O19, hydrogen bond-water hydrogen bond; conventional hydrogen bond
HOH825	H36, hydrogen bond-water hydrogen bond; conventional hydrogen bond
Ser287	H29, hydrogen bond-conventional hydrogen bond
Gln119	H29, hydrogen bond-conventional hydrogen bond
Ser287	H30, hydrogen bond-carbon hydrogen bond
Trp82	N21, electrostatic-π-cation
Trp82	N21, electrostatic-π-cation
Trp231	**1**, hydrophobic-π-π T-shaped
Phe329	**1**, hydrophobic-π-π T-shaped
Leu286	**1**, hydrophobic-π-alkyl

Interactions between compound **2** and BChE.

Amino acid	Non-bonding interactions
HOH706	H43, hydrogen bond-water hydrogen bond; carbon hydrogen bond
Ser287	H39, hydrogen bond-conventional hydrogen bond
Gly117	O22, hydrogen bond-conventional hydrogen bond
Phe329	N7, electrostatic-π-cation
Trp231	N24, electrostatic-π-cation
Trp231	N24, electrostatic-π-cation
Phe329	**2**, hydrophobic-π-π stacked
Trp82	C12, hydrophobic-π-alkyl
Trp82	C12, hydrophobic-π-alkyl
Ala328	**2**, hydrophobic-π-alkyl

Interactions between compound **3** and BChE.

Amino acid	Non-bonding interactions
Glu197	N22, electrostatic-attractive charge
HOH786	O20, hydrogen bond-water hydrogen bond; conventional hydrogen bond
HOH831	O20, hydrogen bond-water hydrogen bond; conventional hydrogen bond
Ser287	H30, hydrogen bond-conventional hydrogen bond
Trp82	N22, electrostatic-π-cation
Trp82	N22, electrostatic-π-cation
Phe329	**3**, hydrophobic-π-π stacked
Phe329	**3**, hydrophobic-π-π T-shaped

Interactions between compound **4** and BChE.

Amino acid	Non-bonding interactions
Asp70	N20, electrostatic-attractive charge
HOH825	H38, hydrogen bond-water hydrogen bond; carbon hydrogen bond

HOH825	H40, hydrogen bond-water hydrogen bond; conventional hydrogen bond
Gln119	O26, hydrogen bond-conventional hydrogen bond
His438	H33, hydrogen bond-conventional hydrogen bond
Tyr332	N20, electrostatic-π-cation
Trp82	H37, hydrogen bond-π-donor hydrogen bond
Trp82	**4**, hydrophobic-π-π T-shaped
Trp82	**4**, hydrophobic-π-π T-shaped
Trp231	**4**, hydrophobic-π-π T-shaped
Trp231	**4**, hydrophobic-π-π T-shaped
Phe329	**4**, hydrophobic-π-π T-shaped
Phe329	**4**, hydrophobic-π-π T-shaped
Gly116;Gly117	**4**, hydrophobic-amide-π stacked
Gly116;Gly117	**4**, hydrophobic-amide-π stacked
Leu286	**4**, hydrophobic-π-alkyl

Interactions between compound **5** and BChE.

Amino acid	Non-bonding interactions
Glu197	N20, electrostatic-attractive charge
HOH786	O18, hydrogen bond-water hydrogen bond; conventional hydrogen bond
HOH831	O18, hydrogen bond-water hydrogen bond; conventional hydrogen bond
Ser287	H30, hydrogen bond-conventional hydrogen bond
Trp82	N20, electrostatic-π-cation
Trp82	N20, electrostatic-π-cation
Phe329	**5**, hydrophobic-π-π T-shaped
Leu286	C26, hydrophobic-alkyl
Trp231	C26, hydrophobic-π-alkyl
Trp231	C26, hydrophobic-π-alkyl
Phe398	C26, hydrophobic-π-alkyl

Interactions between compound **6** and BChE.

Amino acid	Non-bonding interactions
HOH825	H39, hydrogen bond-water hydrogen bond; carbon hydrogen bond
Gln119	H29, hydrogen bond-conventional hydrogen bond
Ser198	H32, hydrogen bond-conventional hydrogen bond
His438	H32, hydrogen bond-conventional hydrogen bond
Gln119	H30, hydrogen bond-carbon hydrogen bond
Trp82	O18, hydrogen bond-carbon hydrogen bond
Trp82	**6** hydrophobic-π-π T-shaped
Trp82	**6**, hydrophobic-π-π T-shaped
Trp231	**6**, hydrophobic-π-π T-shaped
Trp231	**6**, hydrophobic-π-π T-shaped
Phe329	**6**, hydrophobic-π-π T-shaped
His438	**6**, hydrophobic-π-π T-shaped
Gly116;Gly117	**6**, hydrophobic-amide-π stacked
Leu286	**6**, hydrophobic-π-alkyl

Interactions between compound **7** and BChE.

Amino acid	Non-bonding interactions
Glu197	N20, electrostatic-attractive charge
HOH786	O18, hydrogen bond-water hydrogen bond; conventional hydrogen bond
HOH825	H36, hydrogen bond-water hydrogen bond; conventional hydrogen bond
Ser287	H29, hydrogen bond-conventional hydrogen bond
Ser198	F25, hydrogen bond-carbon hydrogen bond
Ser287	H30, hydrogen bond-carbon hydrogen bond
Trp82	N20, electrostatic-π-cation
Trp82	H39, hydrogen bond; electrostatic-π-cation; π-donor hydrogen bond
Trp231	**7**, hydrophobic-π-π T-shaped
Trp231	**7**, hydrophobic-π-π T-shaped
Phe329	**7**, hydrophobic-π-π T-shaped
Gly116;Gly117	**7**, hydrophobic-amide-π stacked
Leu286	**7**, hydrophobic-π-alkyl

Interactions between compound **8** and BChE.

Amino acid	Non-bonding interactions
Glu197	N20, electrostatic-attractive charge
HOH709	O18, hydrogen bond-water hydrogen bond; conventional hydrogen bond
Ser287	H29, hydrogen bond-carbon hydrogen bond
Ser198	H44, hydrogen bond-conventional hydrogen bond
Glu197	H45, hydrogen bond-conventional hydrogen bond
Trp82	N20, electrostatic-π-cation
Trp231	**8**, hydrophobic-π-π T-shaped
Trp231	**8**, hydrophobic-π-π T-shaped
Leu286	**8**, hydrophobic-π-alkyl
Leu286	**8**, hydrophobic-π-alkyl

Interactions between compound **9** and BChE.

Amino acid	Non-bonding interactions
HOH786	O18, hydrogen bond-water hydrogen bond; conventional hydrogen bond
Ser198	F28, hydrogen bond; halogen-carbon hydrogen bond; halogen (fluorine)
Ser287	H32, hydrogen bond-conventional hydrogen bond
Pro285	H35, hydrogen bond-conventional hydrogen bond
His438	F28, halogen-halogen (fluorine)
Trp82	N20, electrostatic-π-cation
Trp82	N20, electrostatic-π-cation
Trp82	O18, electrostatic-π-anion
Trp82	O18, electrostatic-π-anion
Tyr332	**9**, hydrophobic-π-π T-shaped
Leu286	C25, hydrophobic-alkyl
Trp231	C25, hydrophobic-π-alkyl
Trp231	C25, hydrophobic-π-alkyl
Phe398	C25, hydrophobic-π-alkyl
Leu286	**9**, hydrophobic-π-alkyl

Interactions between compound **10** and BChE.

Amino acid	Non-bonding interactions
Asp70	N7, electrostatic-attractive charge
HOH806	F27, hydrogen bond; halogen–water hydrogen bonds; conventional hydrogen bond; halogen (fluorine)
HOH806	H32, hydrogen bond-water hydrogen bond; conventional hydrogen bond
Ser198	O18, hydrogen bond-conventional hydrogen bond
Asp70	H33, hydrogen bond-carbon hydrogen bond
HOH930	**10**, other-π-lone pair
Gly116;Gly117	**10**, hydrophobic-amide-π stacked

Interactions between compound **11** and BChE.

Amino acid	Non-bonding interactions
HOH771	O18, hydrogen bond-water hydrogen bond; conventional hydrogen bond
HOH930	F26, hydrogen bond; halogen–water hydrogen bonds; conventional hydrogen bond; halogen (fluorine)
HOH709	F28, hydrogen bond; halogen–water hydrogen bonds; conventional hydrogen bond; halogen (fluorine)
HOH825	H40, hydrogen bond-water hydrogen bond; conventional hydrogen bond
His438	H34, hydrogen bond-conventional hydrogen bond
Gln119	F27, halogen-halogen (fluorine)
Gln119	F28, halogen-halogen (fluorine)
Trp82	N20, electrostatic-π-cation
Glu197	**11**, electrostatic-π-anion
Trp82	**11**, hydrophobic-π-π T-shaped
Trp82	**11**, hydrophobic-π-π T-shaped
Trp231	**11**, hydrophobic-π-π T-shaped
Trp231	**11**, hydrophobic-π-π T-shaped
Phe329	**11**, hydrophobic-π-π T-shaped
Phe329	**11**, hydrophobic-π-π T-shaped
Gly116;Gly117	**11**, hydrophobic-amide-π stacked

Leu286	**11**, hydrophobic-π-alkyl

Interactions between compound **12** and BChE.

Amino acid	Non-bonding interactions
Glu197	N20, electrostatic-attractive charge
HOH709	N26, hydrogen bond-water hydrogen bond; conventional hydrogen bond
HOH825	H37, hydrogen bond-water hydrogen bond; conventional hydrogen bond
Thr120	N26, hydrogen bond-conventional hydrogen bond
Leu286	H30, hydrogen bond-conventional hydrogen bond
Trp231	N7, electrostatic-π-cation
Trp231	N7, electrostatic-π-cation
Trp82	N20, electrostatic-π-cation
Trp82	N20, electrostatic-π-cation
Trp231	**12**, hydrophobic-π-π T-shaped
Phe329	**12**, hydrophobic-π-π T-shaped
Gly116;Gly117	**12**, hydrophobic-amide-π stacked
Leu286	**12**, hydrophobic-π-alkyl

Interactions between compound **13** and BChE.

Amino acid	Non-bonding interactions
Glu197	N20, electrostatic-attractive charge
HOH736	O18, hydrogen bond-water hydrogen bond; conventional hydrogen bond
HOH706	O26, hydrogen bond-water hydrogen bond; conventional hydrogen bond
Ser198	O26, hydrogen bond-conventional hydrogen bond
Leu286	H31, hydrogen bond-conventional hydrogen bond
His438	O27, hydrogen bond-carbon hydrogen bond
Ser287	H32, hydrogen bond-carbon hydrogen bond
Glu197	H47, hydrogen bond-carbon hydrogen bond
Trp82	N20, electrostatic-π-cation
Trp82	N20, electrostatic-π-cation
Trp231	**13**, hydrophobic-π-π T-shaped
Trp231	**13**, hydrophobic-π-π T-shaped
Phe329	**13**, hydrophobic-π-π T-shaped
Phe329	**13**, hydrophobic-π-π T-shaped
Gly116;Gly117	**13**, hydrophobic-amide-π stacked
Leu286	**13**, hydrophobic-π-alkyl

Interactions between compound **14** and BChE.

Amino acid	Non-bonding interactions
HOH825	H38, hydrogen bond-water hydrogen bond; carbon hydrogen bond
HOH825	H40, hydrogen bond-water hydrogen bond; conventional hydrogen bond
HOH910	H50, hydrogen bond-water hydrogen bond; conventional hydrogen bond
Leu286	H30, hydrogen bond-conventional hydrogen bond
Ser198	H33, hydrogen bond-conventional hydrogen bond
His438	H33, hydrogen bond-conventional hydrogen bond
Asp70	H47, hydrogen bond-carbon hydrogen bond
Trp231	N7, electrostatic-π-cation
Trp231	N7, electrostatic-π-cation
Tyr332	N21, electrostatic-π-cation
Trp82	H37, hydrogen bond-π-donor hydrogen bond
His438	**14**, hydrophobic-π-π stacked
Trp231	**14**, hydrophobic-π-π T-shaped
Trp231	**14**, hydrophobic-π-π T-shaped
Phe329	**14**, hydrophobic-π-π T-shaped
Phe329	**14**, hydrophobic-π-π T-shaped
Gly116;Gly117	**14**, hydrophobic-amide-π stacked
Gly116;Gly117	**14**, hydrophobic-amide-π stacked
Leu286	**14**, hydrophobic-π-alkyl

3. Metal chelation study

3.1. UV/Vis spectra of compounds, metals and compounds–biometal mixtures

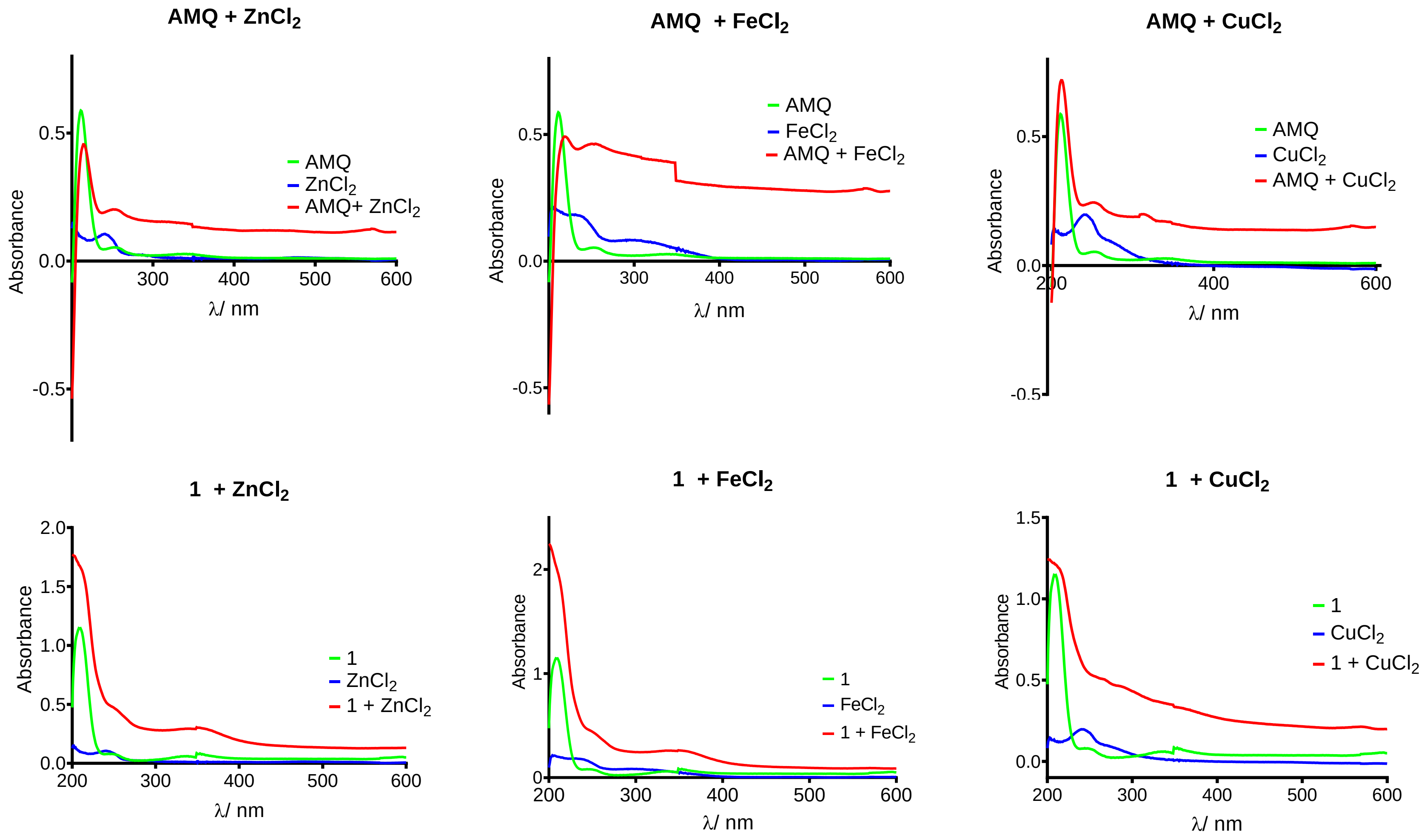

Figure S1. UV/Vis spectra of compounds (**AMQ-1**), metals (Zn^{2+}, Fe^{2+} and Cu^{2+}) and compound–biometal mixtures: compound is represented by green line, metal (Zn^{2+}, Fe^{2+} or Cu^{2+}) is represented by blue line, while the compound–biometal mixture is represented by red line.

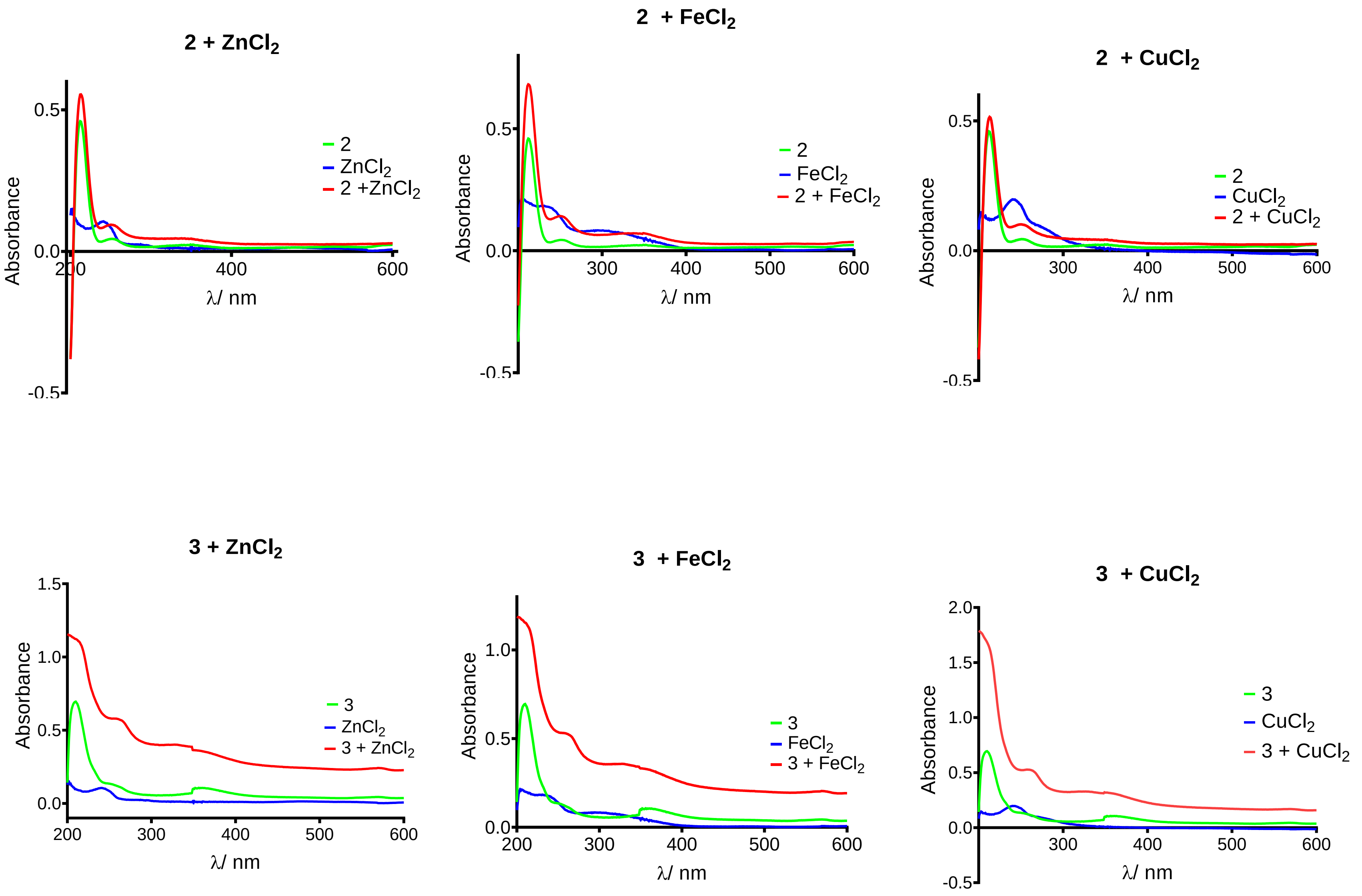

Figure S2. UV/Vis spectra of compounds (**2-3**), metals (Zn^{2+}, Fe^{2+} and Cu^{2+}) and compound–biometal mixtures: compound is represented by green line, metal (Zn^{2+}, Fe^{2+} or Cu^{2+}) is represented by blue line, while the compound–biometal mixture is represented by red line.

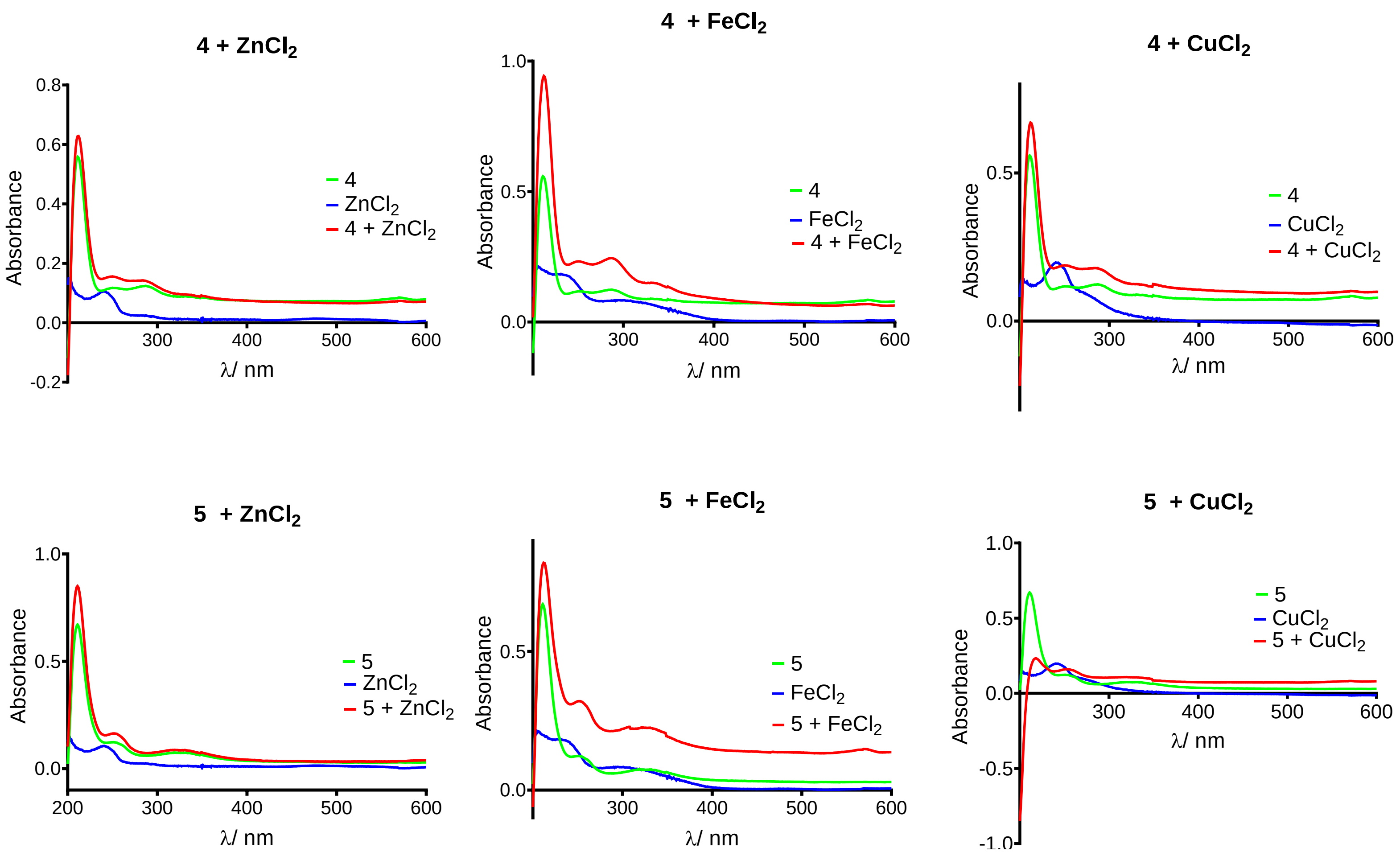

Figure S3. UV/Vis spectra of compounds (**4-5**), metals (Zn^{2+}, Fe^{2+} and Cu^{2+}) and compound–biometal mixtures: compound is represented by green line, metal (Zn^{2+}, Fe^{2+} or Cu^{2+}) is represented by blue line, while the compound–biometal mixture is represented by red line.

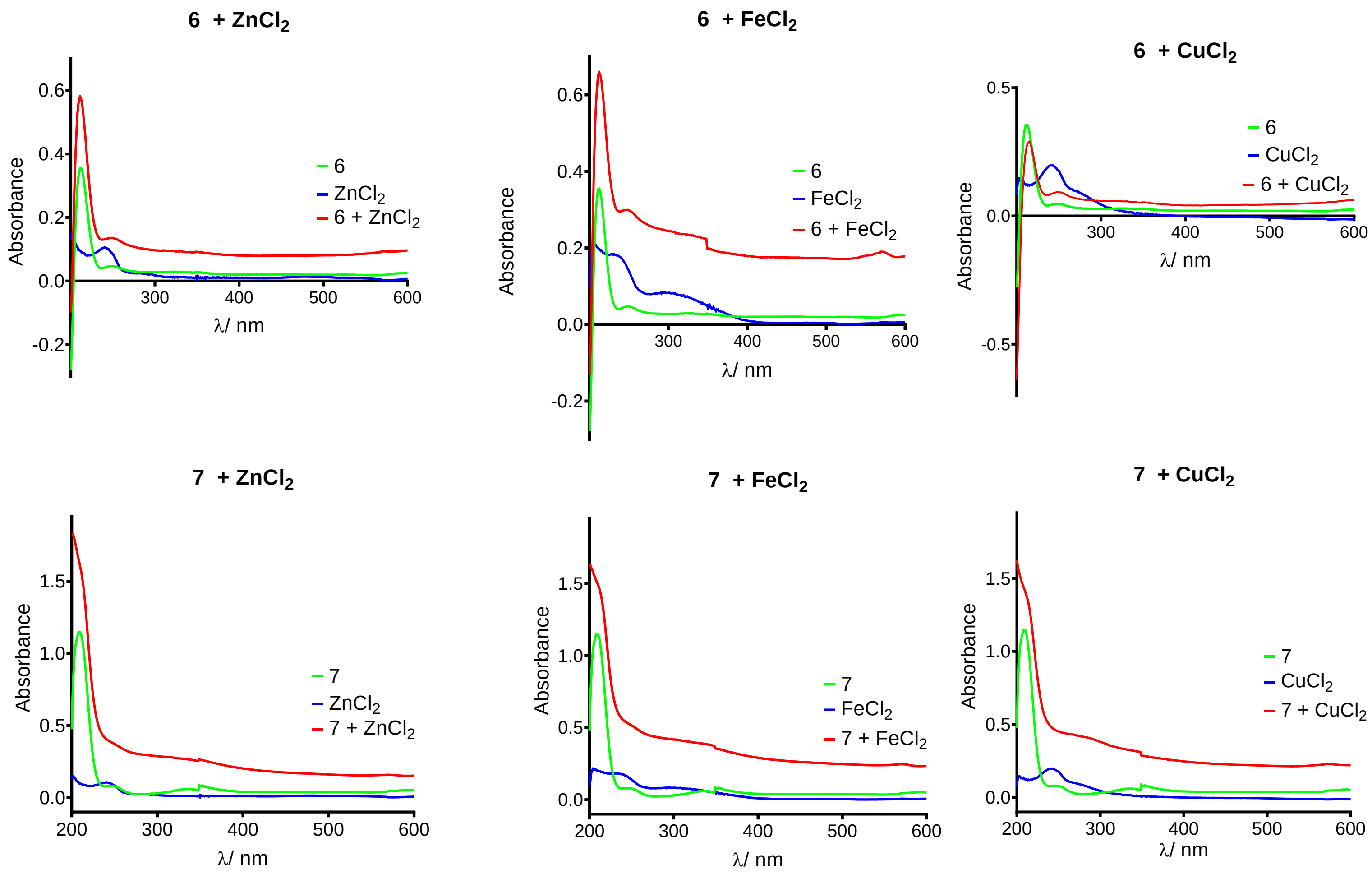

Figure S4. UV/Vis spectra of compounds (**6**-**7**), metals (Zn^{2+}, Fe^{2+} and Cu^{2+}) and compound–biometal mixtures: compound is represented by green line, metal (Zn^{2+}, Fe^{2+} or Cu^{2+}) is represented by blue line, while the compound–biometal mixture is represented by red line.

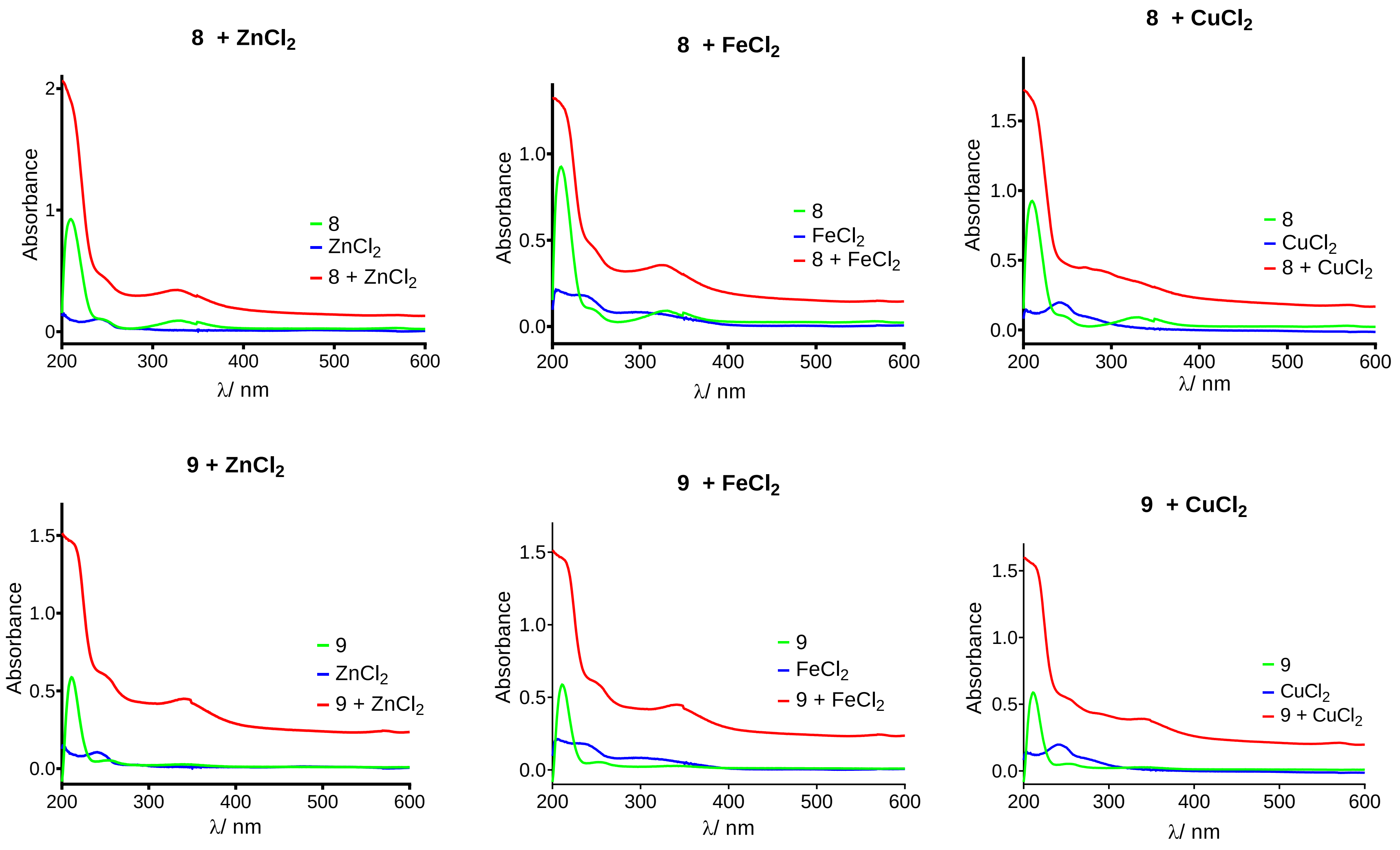

Figure S5. UV/Vis spectra of compounds (**8-9**), metals (Zn^{2+}, Fe^{2+} and Cu^{2+}) and compound–biometal mixtures: compound is represented by green line, metal (Zn^{2+}, Fe^{2+} or Cu^{2+}) is represented by blue line, while the compound–biometal mixture is represented by red line.

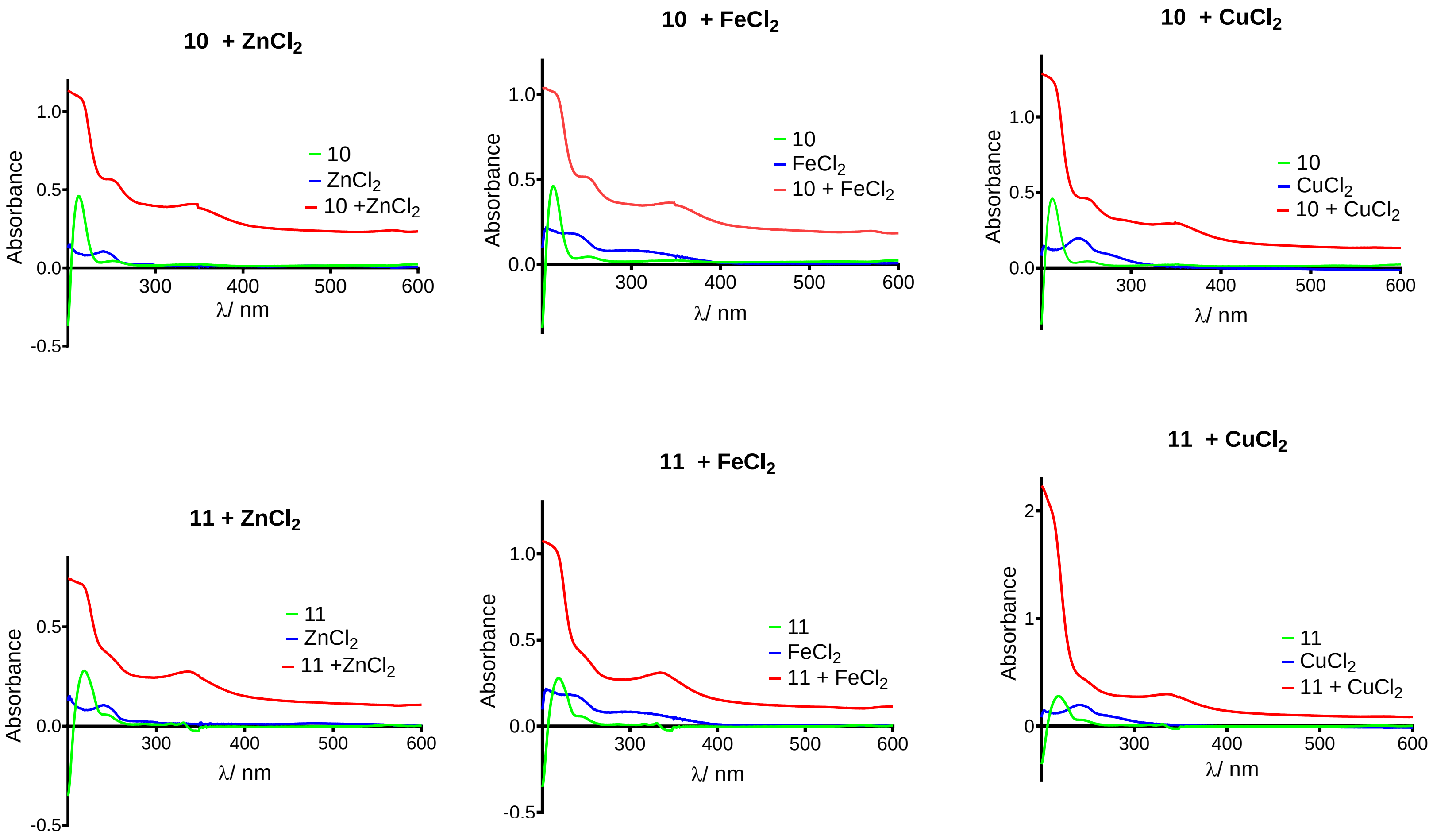

Figure S6. UV/Vis spectra of compounds (**10-11**), metals (Zn^{2+}, Fe^{2+} and Cu^{2+}) and compound–biometal mixtures: compound is represent by green line, metal (Zn^{2+}, Fe^{2+} or Cu^{2+}) is represented by blue line, while the compound–biometal mixture is represented by red line.

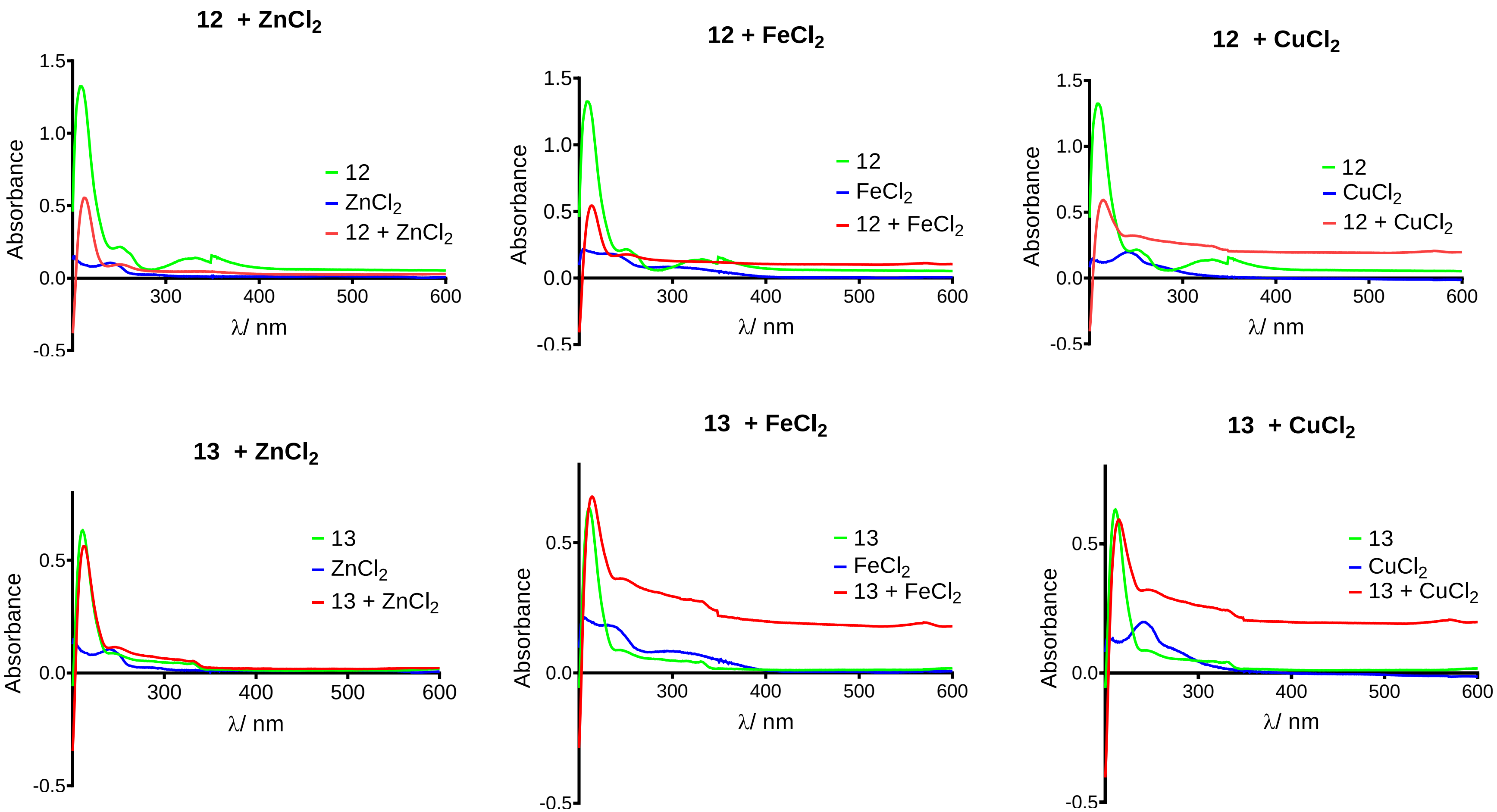

Figure S7. UV/Vis spectra of compounds (**12-13**), metals (Zn^{2+}, Fe^{2+} and Cu^{2+}) and compound–biometal mixtures: compound is represented by green line, metal (Zn^{2+}, Fe^{2+} or Cu^{2+}) is represented by blue line, while the compound–biometal mixture is represented by red line.

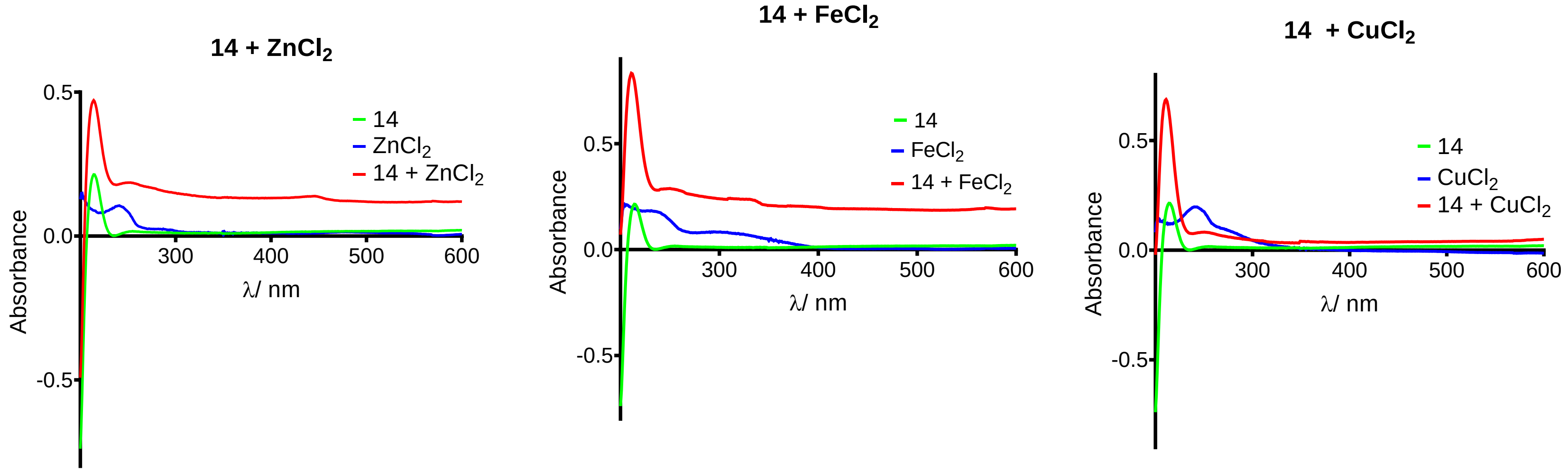

Figure S8. UV/Vis spectra of compound (**14**), metals (Zn^{2+}, Fe^{2+} and Cu^{2+}) and compound–biometal mixtures: compound is represented by green line, metal (Zn^{2+}, Fe^{2+} or Cu^{2+}) is represented by blue line, while the compound–biometal mixture is represented by red line.

3.2. Differential UV/VIS spectra of the compounds-biometal mixtures

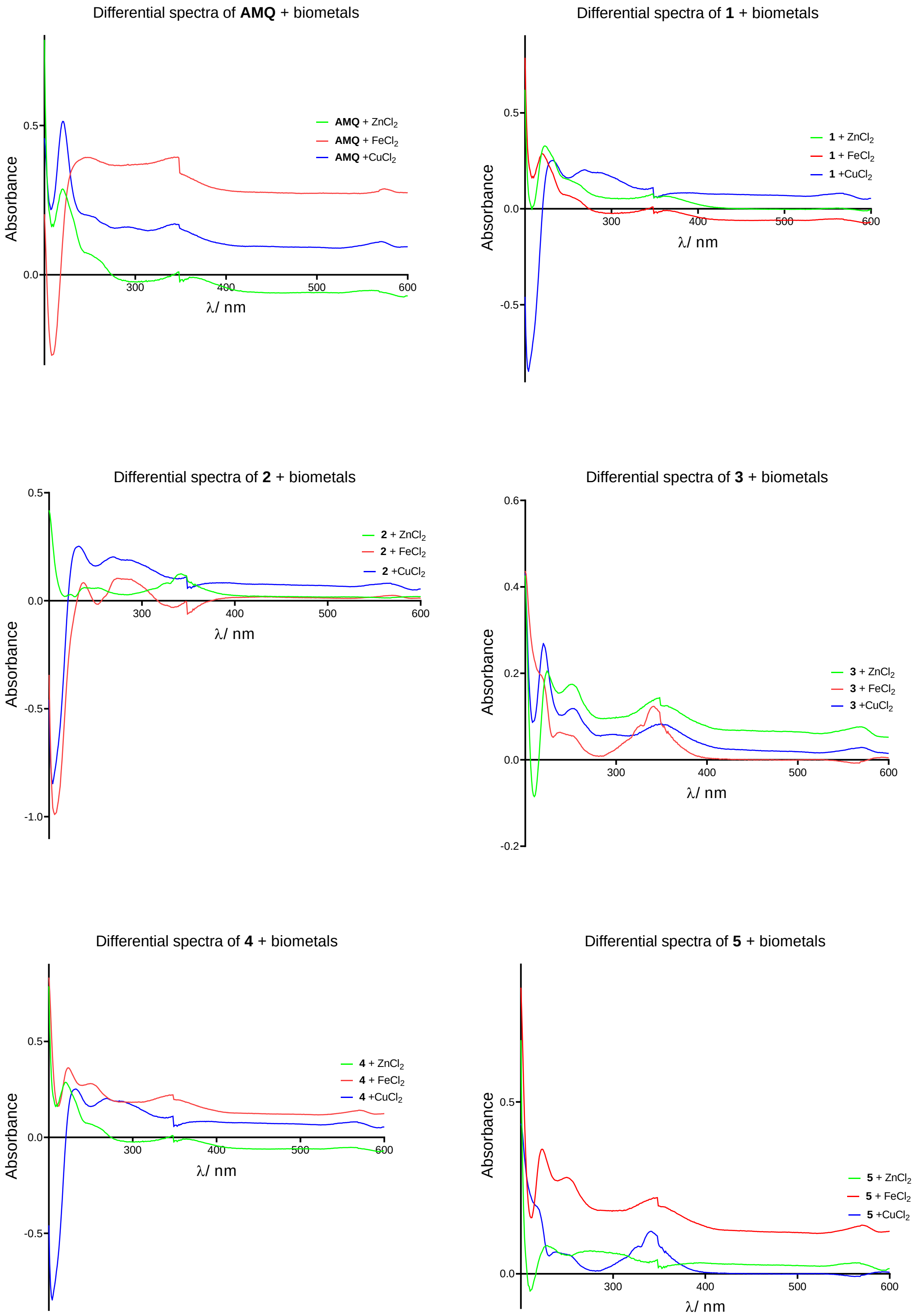

Figure S9. Differential UV/VIS spectra of the compound–biometal mixtures for compounds **AMQ-5.**

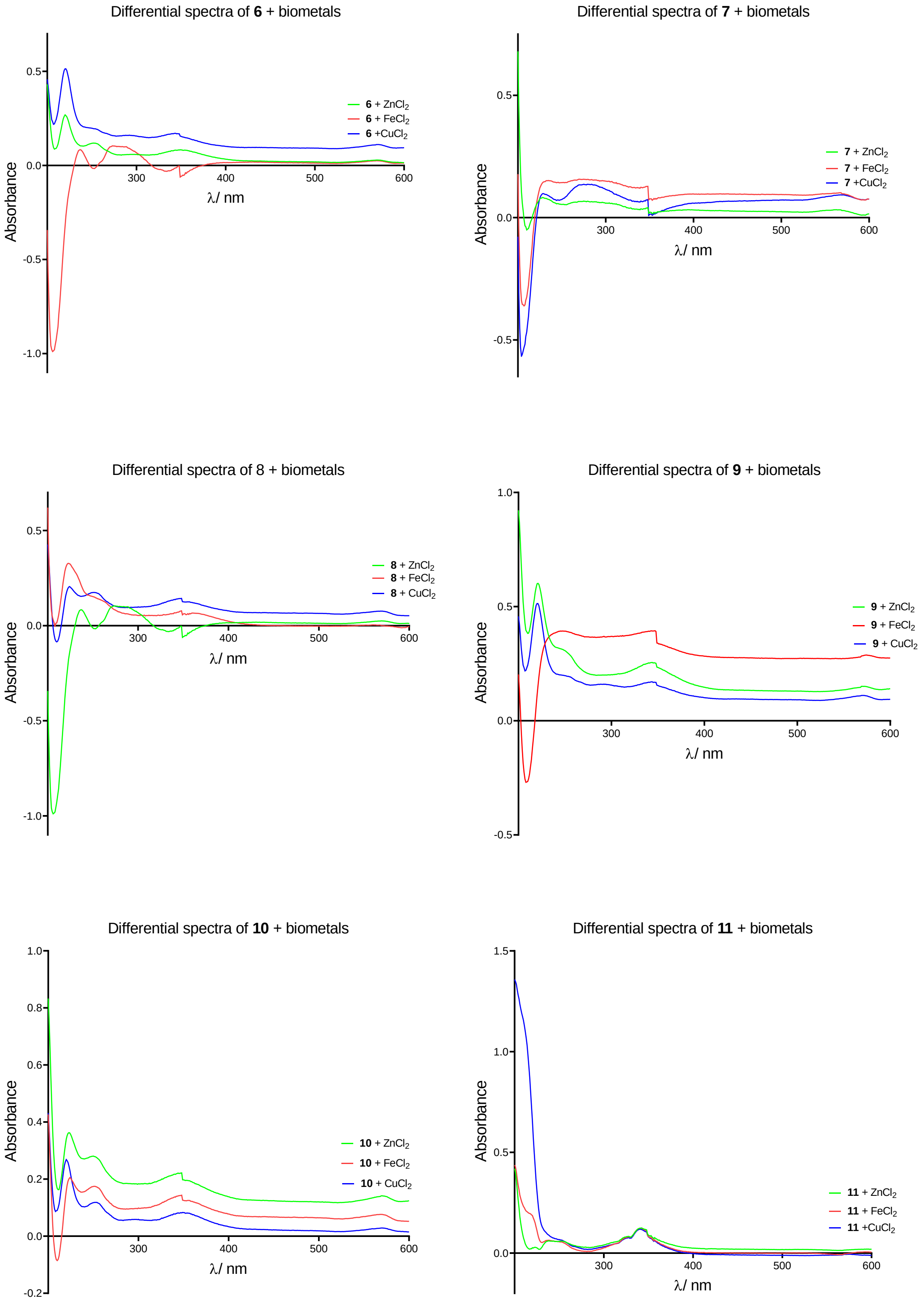

Figure S10. Differential UV/VIS spectra of the compound–biometal mixtures for compounds **6-11.**

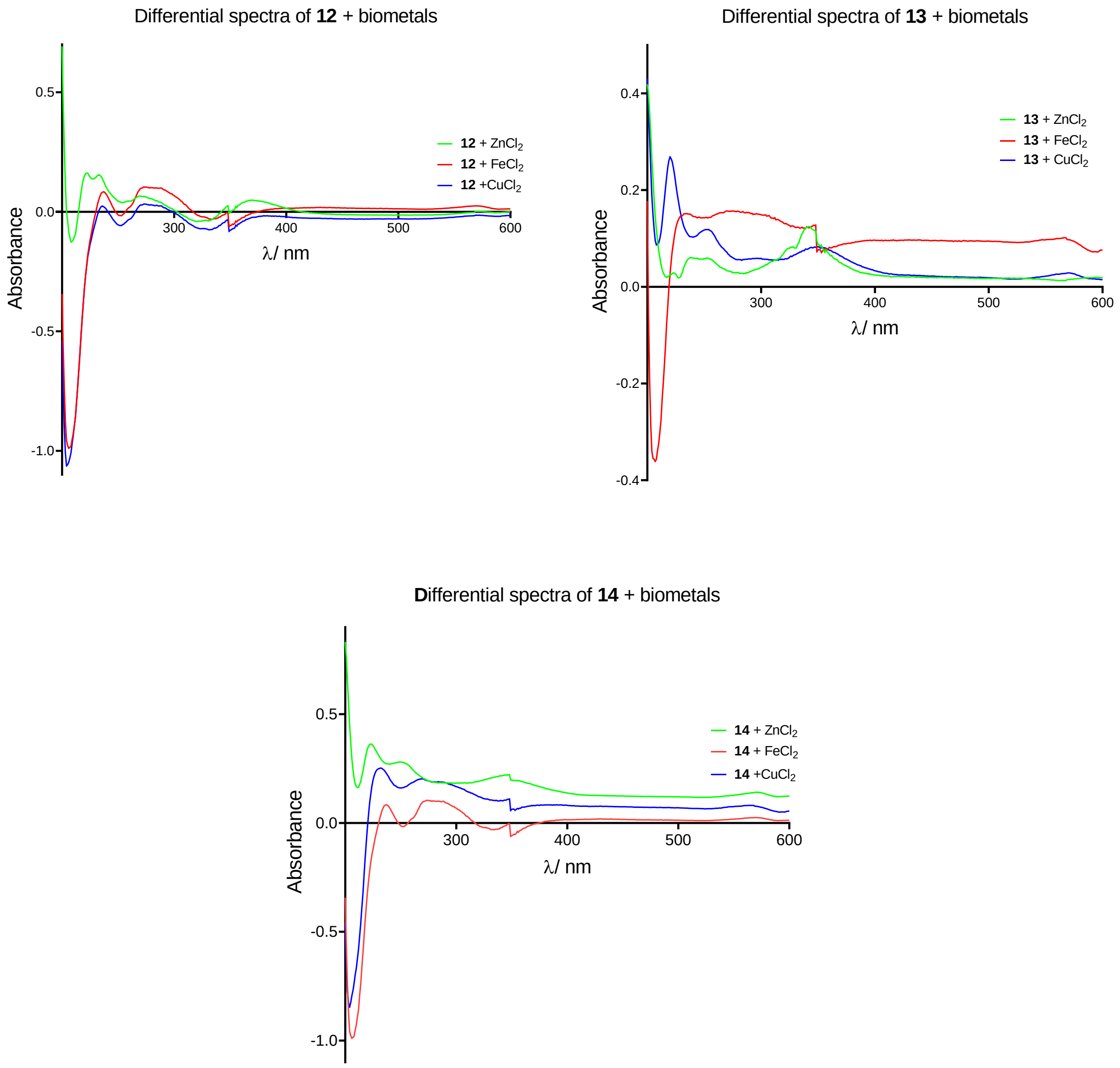

Figure S11. Differential UV/VIS spectra of the compound–biometal mixtures for compounds **12-14.**

4. In silico calculations

Table S1. Calculated physiochemical properties (molecular weight, MW; lipophilicity coefficient, logP; number of hydrogen bond donors, HBD; and number of hydrogen bond acceptors, HBA; rotatable bonds, RB; polar surface area, PSA) of aminoquinolines.

Compound	MW/10	logP	HBD	HBA	RB	PSA/10
**AMQ**	3.559	3.735	2	4	6	4.839
**1**	3.394	3.273	2	4	6	4.839
**2**	3.894	4.008	2	4	7	4.839
**3**	3.464	2.816	2	5	6	7.218
**4**	3.464	2.816	2	5	6	7.218
**5**	3.515	2.06	2	5	7	5.762
**6**	3.214	3.129	2	4	6	4.839
**7**	3.339	3.274	2	4	6	4.839
**8**	3.339	3.274	2	4	6	4.839
**9**	3.894	4.007	2	4	7	4.839
**10**	3.894	4.009	2	4	7	4.839
**11**	3.894	4.394	2	4	7	4.839
**12**	3.464	2.816	2	5	6	7.218
**13**	3.654	0.969	3	6	7	8.569
**14**	3.514	2.968	3	5	7	8.569
**Recommended values**	5	5	5	5	10	9

Table S2. In silico estimated % of compounds that will be absorbed through the human intestine.

Compound	Human intestinal absorption %
**AMQ**	99.4
**1**	99.2
**2**	99.4
**3**	97.4
**4**	99.7
**5**	98.6
**6**	99.2
**7**	99.2
**8**	99.2
**9**	99.5
**10**	99.5
**11**	99.6
**12**	97.3
**13**	92.8
**14**	98.5

References

1. Nsumiwa, S., Kuter, D., Wittlin, S., Chibale, K., Egan, T. J. Structure–activity relationships for ferriprotoporphyrin IX association and β-hematin inhibition by 4-aminoquinolines using experimental and ab initio methods. *Bioorgan Med Chem* **2013**, *21* (13), 3738-3748. doi: 10.1016/j.bmc.2013.04.040
